# Comparative global burden of ischemic heart disease and myocardial disease attributable to non-optimal temperatures, 1990–2021: an analysis based on GBD 2021

**DOI:** 10.3389/fpubh.2025.1605624

**Published:** 2025-09-23

**Authors:** Mengqi Guo, Zhexun Lian, Zongyi Xia, Lingbing Wang, Hui Xin, Fuhai Li

**Affiliations:** ^1^Department of Cardiology, The Affiliated Hospital of Qingdao University, Qingdao, Shandong, China; ^2^Clinical Medicine, Shandong Second Medical University, Weifang, Shandong, China; ^3^Department of Cardiology, Third People's Hospital of Huizhou, Guangzhou Medical University, Huizhou, Guangdong, China

**Keywords:** myocardial disease, ischemic heart disease, extreme temperatures, GBD, DALYs, mortality

## Abstract

**Objectives:**

To compare the global burden of myocardial disease (MD) and ischemic heart disease (IHD) attributable to high and low temperatures, and to examine demographic and socio-economic disparities over time.

**Methods:**

We analyzed disability-adjusted life years (DALYs) and mortality for MD and IHD attributable to high and low temperatures, stratified by sex, age, region, and socio-demographic index (SDI). Decomposition analysis quantified the contributions of population growth, aging, and epidemiological changes. Projections were generated using an age-period-cohort model.

**Results:**

Between 1990 and 2021, high temperature-related MD and IHD burdens increased [Estimated Annual Percent Change (EAPC): +1.26 and +1.68%, respectively], whereas low temperature burdens declined (EAPC: −1.87 and −1.73%) but remained considerably higher overall. MD disproportionately affected children under five and adults over 80, while IHD rarely appeared under 30 yet rose markedly from midlife onward. Heat-related MD and IHD burdens rose with SDI < 0.5 and declined above 0.5; cold-related burdens decreased consistently above SDI 0.75 but varied irregularly below this threshold. Central Asia exhibited the greatest heat- and cold-related burdens for both MD and IHD, whereas North Africa and the Middle East were particularly susceptible to heat. Population growth primarily fueled heat-related burdens, whereas cold-related burdens were more driven by aging and population change. Projections to 2040 indicate continuing increases in heat-related burdens, potentially exacerbating health disparities.

**Conclusions:**

Heat-attributable IHD is the fastest-growing threat, while MD remains critical for very young and older adult populations under extreme temperatures. Disparities across age, SDI, and geography highlight the urgency for targeted interventions.

## Introduction

Cardiovascular disease (CVD) remains the leading cause of death worldwide. In 2021, there were an estimated 612 million people living with CVD, accounting for 26.8% of all global deaths (1). Ischemic heart disease (IHD) has consistently been the top cause of age-standardized mortality, responsible for over 9 million deaths in 2019—nearly 16% of global mortality—and contributing significantly to disability-adjusted life years (DALYs) (2, 3). IHD primarily results from atherosclerosis or coronary spasms, restricting coronary blood flow and leading to myocardial infarction or chronic coronary syndromes.

Myocardial disease (MD), including cardiomyopathy and myocarditis, affects cardiac structure and function through mechanisms such as genetic mutations, infections, and autoimmune processes. In 2019, inflammatory cardiomyopathy and myocarditis caused ~340,349 deaths globally, with an age-standardized mortality rate of 4.40 per 100,000, affecting mainly older adults and men (4). While MD is less prevalent than IHD, it can cause severe morbidity and disproportionately impacts younger individuals, including children and young adults, particularly in less developed regions (5).

Environmental factors, particularly climate change, are increasingly implicated in the global CVD burden (6). Children born today may face lifetime temperatures 4°C above pre-industrial levels (7). Global temperatures over the past 7 years have been the highest on record, and each day of heat exposure increases monthly cardiovascular mortality by 0.12% among adults aged 20 and older (8). Simultaneously, Arctic ice melt and altered atmospheric patterns contribute to more frequent cold extremes, elevating CVD mortality in affected regions (9).

Both extreme high and low temperatures pose distinct risks for CVD patients. Heat exposure exacerbates dehydration, electrolyte imbalances, and increased blood viscosity, triggering arrhythmias, heart failure, or acute coronary events (10). In contrast, cold exposure raises blood pressure and systemic inflammation, straining cardiac function and increasing risks of myocardial infarction or heart failure (11, 12). Despite known associations, existing studies often focus on aggregate cardiovascular outcomes rather than disease-specific impacts, leaving geographic and socioeconomic differences underexplored (9, 13, 14). Our study fills these gaps by quantifying the global burdens attributable to non-optimal temperatures for IHD and MD separately and by providing detailed demographic and regional breakdowns to guide targeted interventions.

## Methods

### Data sources

The Global Burden of Diseases (GBD) 2021 study, conducted by the Institute for Health Metrics and Evaluation (IHME), provides comprehensive estimates for 369 diseases and 88 risk factors across 204 countries or regions. Study design and methods are detailed in prior GBD literature (2, 15). The University of Washington IRB waived informed consent for GBD data access (16). This study adhered to the Guidelines for Accurate and Transparent Health Estimates Reporting (GATHER) (17).

### Defining non-optimal temperatures

Temperature estimates were derived from the ERA5 reanalysis dataset, provided by the European Center for Medium-Range Weather Forecasts, which offers high-resolution (0.25° × 0.25°) temperature data from 1979 to the present. Non-optimal temperatures were defined as temperatures exceeding or falling below the theoretical minimum risk exposure level, which corresponds to daily temperatures associated with the lowest mortality rates for specific locations. To mitigate the influence of extreme temperatures and noise, data were truncated, preventing extrapolation of temperatures beyond the range of mortality data. Temperature ranges were truncated at the 1st and 99th percentiles of the mortality dataset, and daily mean temperatures were similarly truncated within each temperature range (13, 18).

### Estimated cardiovascular disease burden attributable to non-optimal temperature exposures

Non-optimal temperatures, including both extreme high and low temperatures, are identified as risk factors for myocarditis/cardiomyopathy (referred to as myocardial disease; MD) and ischemic heart disease (IHD) in the GBD database. Cause-specific relative risks of non-optimal temperature on daily mortality in the Global Burden of Disease Study were estimated using methods by Burkart et al. (19). Firstly, a Bayesian meta-regression framework with a two-dimensional spline model was applied to analyze the relative risks of 176 causes of death related to daily temperature. These relative risks were then applied globally, using daily temperature data combined with Global Burden of Disease Study data to calculate disease burden for each region.

### Data analysis

Burden was measured using mortality and disability-adjusted life years (DALYs), comprising years of life lost and years lived with disability (YLD). Age-standardized rates were calculated using the direct method with the GBD 2019 standard population as a reference, expressed per 100,000 population (ASMR for mortality, ASDR for DALYs). Age standardization was applied to eliminate the confounding effects of different population age structures across regions. Trends from 1990 to 2021 were assessed using Estimated Annual Percent Change (EAPC); Total Percentage Change (TPC) was used for regions with missing or negative values. Analyses were stratified by sex, 5-year age groups, and socio-demographic index (SDI) categories to capture burden patterns at global, regional, and national levels.

#### Decomposition analysis

To understand the contributions of population growth, aging, and epidemiological shifts, we conducted a decomposition analysis. This method involved comparing the observed burden in 2021 with hypothetical scenarios that isolated each of these three factors separately. Specifically, we recalculated DALYs and mortality assuming: (1) population size and age structure remained constant while disease rates changed (epidemiological changes), (2) age-specific rates and total population size were constant while age structure changed (aging effects), and (3) age-specific rates and age structure remained constant while the population size changed (population growth effects). The relative contributions of each factor were then quantified by comparing these hypothetical scenarios to the observed 2021 data (20).

#### Predicting trends

Future cases and incidence rates (2021–2040) were projected using a log-linear age-period-cohort model (21). Specifically, we utilized the NORDPRED package in R, applying age-period-cohort modeling to historical age-specific rates to estimate future incidence and mortality rates. Trends were projected by logarithmically extrapolating observed changes, with adjustments assuming gradually decreasing trends in increments of 25%, 50%, and 75% reductions applied sequentially across forecast intervals. Projections for the final year (2040) combined weighted averages of the preceding two forecast intervals, adjusted using United Nations national population forecasts for consistency (22). Analyses were conducted in R Studio using the NORDPRED package, with statistical significance set at *P* < 0.05.

### Statistical analysis

A positive 95% confidence interval indicated increasing trends, negative values indicated decreases, and intervals including zero suggested stability. All analyses were performed using R (version 4.2.1), with a two-sided *P*-value < 0.05 considered significant.

## Results

### Global assessment of cardiovascular burden from extreme temperatures (1990–2021)

**Figure 1:**
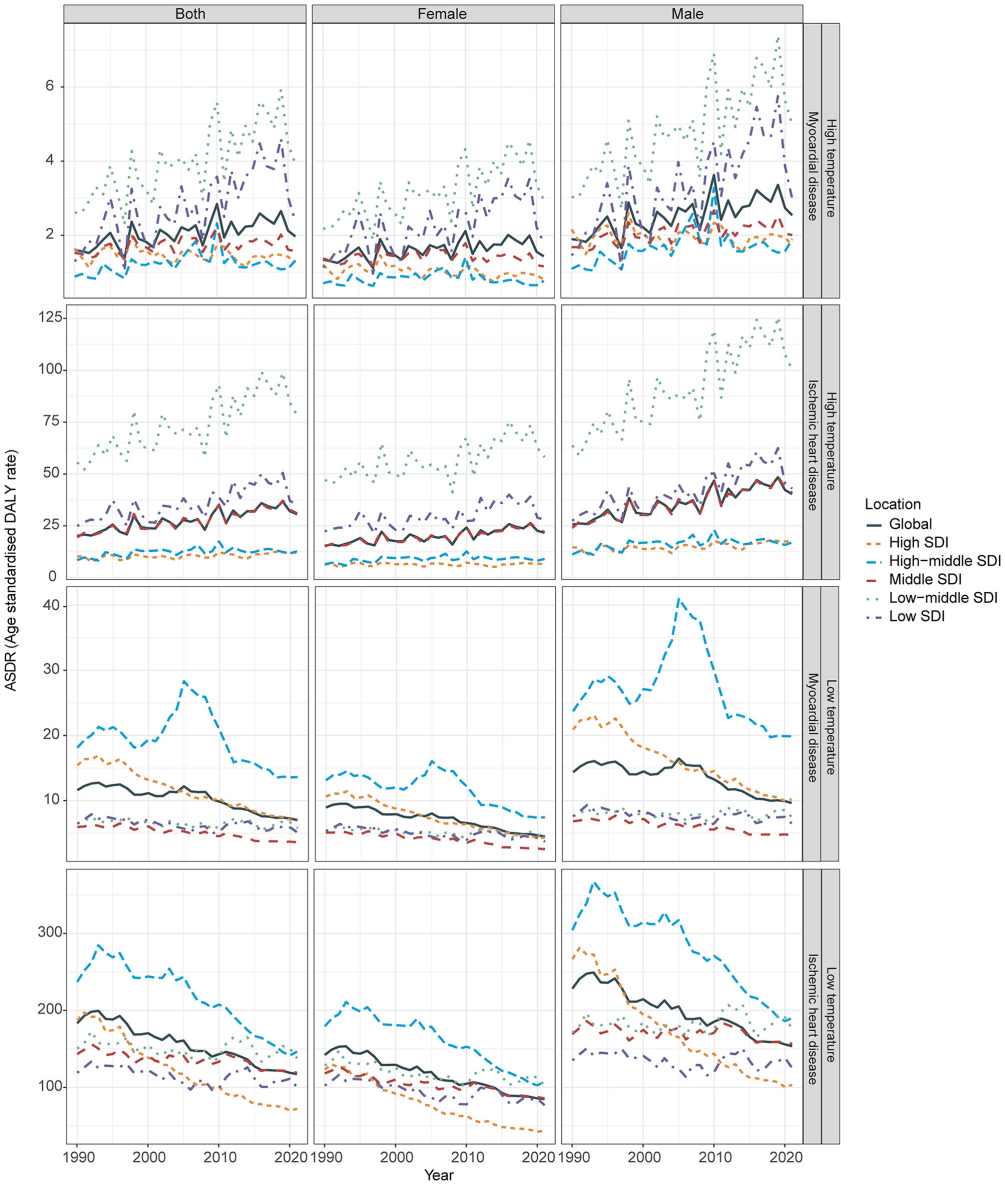
Age-standardized DALYs rate (ASDR) for myocardial disease, and ischemic heart disease attributable to high and low temperatures from 1990 to 2021, stratified by the sociodemographic index (SDI). DALYs, Disability-adjusted life years.

From 1990 to 2021, age-standardized mortality rates (ASMR) and age-standardized DALY rates (ASDR) attributable to high temperatures for myocardial disease (MD) and ischemic heart disease (IHD) generally increased, although with fluctuations. By 2021, the ASDR for heat-attributable MD was 1.97 per 100,000 (95% CI: −0.80 to 4.76), corresponding to 158,029 DALYs, while the ASMR reached 0.06 per 100,000 (95% CI: −0.02 to 0.15), with 5,224 deaths. For IHD, the ASDR was 30.57 per 100,000 (95% CI: 5.41 to 66.96), amounting to 2,623,940 DALYs, with an ASMR of 1.34 per 100,000 (95% CI: 0.20–3.07), corresponding to 112,390 deaths. Over 30 years, the estimated annual percent change (EAPC) in DALYs attributable to high temperatures was 1.26% (95% CI: 0.78% −1.75%) for MD, and 1.68% (95% CI: 1.33% −2.03%) for IHD. Mortality rates showed similar trends, with EAPCs of 1.35% (95% CI: 0.86% −1.85%) and 1.66% (95% CI: 1.31% −2%), respectively (Figure 1).

In contrast, ASMR and ASDR attributable to low temperatures, although historically higher, showed consistent reductions over time. By 2021, the ASDR for cold-attributable MD was 7.01 per 100,000 (95% CI: 5.35–9.03), corresponding to 578,936 DALYs, with an ASMR of 0.27 per 100,000 (95% CI: 0.21–0.34), reflecting 22,248 deaths. For IHD, the ASDR was 117.40 per 100,000 (95% CI: 101.19–144.86), accounting for 9,961,231 DALYs, and the ASMR was 6.14 per 100,000 (95% CI: 5.23–7.53), corresponding to 505,298 deaths. The EAPC in DALYs attributable to low temperatures was −1.87% (−2.17% −1.58%) for MD, and −1.73% (95% CI: −1.85% −1.6%) for IHD, with similar decreasing trends observed in ASMR (Figure 1 and Table 1).

**Table 1:** Global and SDI-region-specific DALYs and mortality rates (ASDR, ASMR), with EAPC values for myocardial disease, and ischemic heart disease attributable to high and low temperatures in 1990 vs. 2021.

**Location**	**Total ASDR (95% UI)**				**Female ASDR (95% UI)**				**Male ASDR (95% UI)**			
	**1990**	**2021**	**TPC**	**EAPC**	**1990**	**2021**	**TPC**	**EAPC**	**1990**	**2021**	**TPC**	**EAPC**
**Myocardial disease attributable to high temperature (1990 vs. 2021)**												
Global	1.60 (−0.75, 4.38)	1.97 (−0.80, 4.76)	0.23 (−0.30, 1.40)	1.26 (0.78–1.75)	1.34 (−0.50, 3.73)	1.43 (−0.50, 3.35)	0.07 (−0.64, 1.09)	0.93 (0.45–1.41)	1.90 (−0.93, 5.13)	2.54 (−1.09, 6.31)	0.34 (−0.25, 1.72)	1.47 (0.97–1.97)
High SDI	1.64 (−0.33, 3.95)	1.27 (−0.14, 2.74)	−0.23 (−0.78, 0.13)	−0.29 (−0.74–0.16)	1.20 (−0.24, 2.87)	0.81 (−0.08, 1.77)	−0.33 (−0.82, −0.05)	−0.74 (−1.21–−0.27)	2.16 (−0.46, 5.19)	1.73 (−0.19, 3.76)	−0.20 (−0.76, 0.15)	−0.15 (−0.61–0.3)
Middle-high SDI	0.89 (−0.46, 2.61)	1.32 (−0.94, 3.74)	0.49 (−0.35, 1.46)	0.97 (0.21–1.73)	0.71 (−0.32, 2.03)	0.77 (−0.48, 2.11)	0.09 (−0.61, 0.84)	0.01 (−0.66–0.7)	1.10 (−0.61, 3.29)	1.89 (−1.39, 5.45)	0.73 (−0.50, 1.85)	1.42 (0.62–2.22)
Middle SDI	1.53 (−0.40, 3.45)	1.57 (−0.31, 3.29)	0.03 (−0.32, 0.57)	0.54 (0.12–0.96)	1.38 (−0.28, 3.09)	1.16 (−0.20, 2.46)	−0.16 (−0.48, 0.45)	0.02 (−0.41–0.44)	1.68 (−0.50, 3.92)	2.01 (−0.42, 4.34)	0.19 (−0.28, 1.13)	0.94 (0.52–1.36)
Middle-low SDI	2.61 (−1.58, 7.50)	3.93 (−1.32, 9.57)	0.51 (−3.75, 4.51)	2 (1.37–2.63)	2.18 (−1.02, 6.87)	2.96 (−0.81, 7.16)	0.36 (−2.91, 8.00)	1.8 (1.18–2.43)	3.03 (−1.70, 9.08)	4.99 (−1.81, 12.56)	0.65 (−2.23, 4.90)	2.19 (1.56–2.83)
Low SDI	1.30 (−2.48, 7.35)	2.38 (−2.14, 7.88)	0.83 (−9.06, 7.65)	2.8 (1.79–3.81)	1.13 (−2.18, 6.40)	1.80 (−1.33, 5.87)	0.59 (−8.43, 4.82)	2.35 (1.38–3.32)	1.46 (−3.06, 8.18)	2.99 (−2.91, 10.33)	1.05 (−5.92, 11.14)	3.14 (2.1–4.2)
**Ischemic heart disease attributable to high temperature (1990 vs. 2021)**												
Global	20.18 (−0.12, 51.93)	30.57 (5.41, 66.96)	0.51 (−1.07, 3.9)	1.68 (1.33–2.03)	15.44 (−0.04, 39.55)	21.65 (3.91, 47.27)	0.40 (−0.87, 4.41)	1.4 (1.04–1.76)	25.6 (−0.2, 65.26)	40.34 (7.01, 89.25)	0.58 (−0.82, 3.94)	1.83 (1.48–2.18)
High SDI	10.29 (−1.15, 35.64)	12.05 (0.95, 31.71)	0.17 (−3.09, 6.06)	0.77 (0.4–1.15)	6.75 (−0.89, 24.01)	6.66 (0.4, 17.77)	−0.01 (−3.00, 4.47)	0.08 (−0.31–0.48)	14.67 (−1.55, 50.16)	17.59 (1.46, 45.87)	0.20 (−3.40, 5.70)	0.91 (0.53–1.29)
High-middle SDI	8.46 (−3.53, 33.02)	12.67 (−3.03, 43.09)	0.5 (−3.02, 4.49)	1.02 (0.5–1.55)	6.24 (−2.64, 24.68)	8.98 (−1.76, 31.54)	0.44 (−3.06, 4.70)	0.87 (0.33–1.42)	11.18 (−4.55, 43.36)	16.93 (−4.48, 58.32)	0.51 (−3.24, 3.86)	1.1 (0.59–1.61)
Middle SDI	19.46 (−2.17, 49.1)	31.05 (5.67, 68.28)	0.6 (−8.72, 5.24)	1.7 (1.34–2.05)	15.12 (−2.44, 39.63)	22.03 (4.09, 47.72)	0.46 (−8.49, 5.73)	1.44 (1.09–1.8)	23.98 (−1.83, 59.82)	41.03 (7.42, 89.32)	0.71 (−8.14, 7.02)	1.9 (1.54–2.26)
Low-middle SDI	55.56 (7.38, 106.82)	78.49 (21.27, 148.31)	0.41 (0.15, 2.01)	1.65 (1.23–2.07)	47.12 (5.43, 92.91)	58.14 (15.78, 107.87)	0.23 (−0.02, 1.52)	1.16 (0.72–1.6)	63.72 (9.29, 125.2)	100.33 (26.49, 192.02)	0.57 (0.27, 2.51)	2.03 (1.62–2.45)
Low SDI	24.9 (−5.09, 58.76)	35.29 (5.17, 70.32)	0.42 (−4.32, 6.66)	1.73 (1.22–2.25)	22.3 (−5.68, 53.08)	28.04 (3.77, 55.19)	0.26 (−5.58, 3.67)	1.32 (0.81–1.83)	27.4 (−5.22, 65.2)	42.96 (6.69, 85.62)	0.57 (−4.56, 6.29)	2.08 (1.55–2.6)
**Myocardial disease attributable to low temperature (1990 vs. 2021)**												
Global	11.61 (9.15, 14.37)	7.01 (5.35, 9.03)	−0.40 (−0.47, −0.31)	−1.87 (−2.17–1.58)	8.95 (6.89, 11.60)	4.49 (3.33, 5.99)	−0.50 (−0.57, −0.41)	−2.38 (−2.59–2.16)	14.37 (11.36, 17.45)	9.64 (7.36, 12.27)	−0.51 (−0.55, −0.48)	−1.58 (−1.92–1.24)
High SDI	15.44 (12.11, 17.61)	7.17 (5.71, 8.16)	−0.54 (−0.57, −0.50)	−2.92 (−3.07–2.76)	10.62 (8.25, 12.13)	4.31 (3.42, 4.94)	−0.59 (−0.63, −0.55)	−3.31 (−3.46–3.16)	20.92 (16.47, 23.80)	10.17 (8.14, 11.53)	−0.51 (−0.55, −0.48)	−2.8 (−2.96–2.64)
Middle-high SDI	18.14 (13.55, 20.96)	13.61 (10.52, 16.42)	−0.25 (−0.34, −0.14)	−1.21 (−1.87–0.54)	13.12 (9.61, 15.50)	7.45 (5.58, 9.29)	−0.43 (−0.52, −0.31)	−2.03 (−2.57–1.48)	23.72 (17.54, 27.51)	19.87 (15.29, 24.31)	−0.16 (−0.27, −0.04)	−0.89 (−1.61–0.17)
Middle SDI	5.97 (4.07, 8.44)	3.62 (2.40, 5.05)	−0.39 (−0.53, −0.26)	−1.88 (−2.1–1.66)	5.10 (3.33, 7.33)	2.55 (1.69, 3.59)	−0.50 (−0.63, −0.29)	−2.38 (−2.63–2.14)	6.83 (4.68, 9.66)	4.72 (3.06, 6.67)	−0.31 (−0.49, −0.15)	−1.51 (−1.72–1.3)
Middle-low SDI	6.52 (1.99, 12.15)	5.79 (2.06, 10.00)	−0.11 (−0.30, 0.22)	−0.34 (−0.6–0.07)	5.40 (1.48, 10.86)	4.18 (1.33, 7.54)	−0.23 (−0.41, 0.28)	−0.66 (−0.93–0.38)	7.61 (2.34, 14.87)	7.52 (2.78, 13.18)	−0.01 (−0.24, 0.34)	−0.07 (−0.34–0.2)
Low SDI	6.45 (2.70, 11.91)	5.08 (2.33, 8.98)	−0.21 (−0.41, 0.26)	−0.88 (−1.21–0.55)	5.29 (1.67, 10.63)	3.71 (1.50, 6.74)	−0.30 (−0.49, 0.29)	−1.18 (−1.53 to −0.84)	7.58 (2.97, 14.72)	6.51 (2.93, 12.15)	−0.14 (−0.37, 0.39)	−0.64 (−0.96 to −0.32)
**Ischemic heart disease attributable to low temperature (1990 vs. 2021)**												
Global	183.27 (161.52, 219.62)	117.39 (101.19, 144.86)	−0.36 (−0.4, −0.32)	−1.73 (−1.85–1.6)	142.15 (123.20, 170.66)	84.77 (72.13, 103.94)	−0.40 (−0.45, −0.36)	−1.98 (−2.1–1.86)	228.30 (201.97, 274.55)	153.52 (131.79, 186.38)	−0.33 (−0.38, −0.27)	−1.55 (−1.67–1.42)
High SDI	187.15 (168.11, 224.88)	72.05 (63.42, 86.78)	−0.61 (−0.63, −0.6)	−3.51 (−3.66–3.37)	123.38 (108.26, 148.94)	43.63 (36.54, 52.71)	−0.65 (−0.66, −0.63)	−3.85 (−3.99–3.7)	266.70 (243.18, 318.54)	103.33 (92.46, 123.28)	−0.61 (−0.63, −0.60)	−3.46 (−3.61–3.31)
Middle-high SDI	236.99 (213.64, 281.68)	146.37 (127.31, 178.50)	−0.38 (−0.43, −0.33)	−2.09 (−2.37–1.81)	179.37 (160.40, 213.64)	107.65 (90.19, 133.69)	−0.40 (−0.46, −0.33)	−2.19 (−2.47–1.92)	304.15 (272.95, 362.23)	190.13 (164.90, 232.67)	−0.37 (−0.44, −0.31)	−2.01 (−2.29–1.74)
Middle SDI	143.31 (118.77, 172.53)	119.00 (101.56, 142.01)	−0.17 (−0.26, −0.07)	−0.63 (−0.78–0.48)	118.26 (97.45, 143.45)	85.76 (71.70, 103.04)	−0.27 (−0.37, −0.16)	−1.06 (−1.21–0.92)	169.16 (138.84, 204.69)	156.58 (132.66, 187.73)	−0.07 (−0.20, 0.07)	−0.27 (−0.43–0.12)
Middle-low SDI	150.20 (111.81, 192.41)	137.92 (104.50, 178.71)	−0.08 (−0.17, 0.02)	−0.27 (−0.5–0.05)	128.43 (94.06, 168.00)	102.49 (76.62, 131.85)	−0.20 (−0.30, −0.10)	−0.73 (−0.96–0.5)	171.01 (123.67, 220.66)	175.87 (132.24, 231.11)	0.03 (−0.10, 0.18)	0.1 (−0.13–0.33)
Low SDI	119.02 (91.61, 151.98)	100.39 (78.76, 124.51)	−0.16 (−0.26, −0.04)	−0.64 (−0.91–0.37)	102.33 (75.38, 131.64)	76.89 (59.26, 95.72)	0.25 (−0.35, −0.12)	−1.02 (−1.31–0.73)	135.15 (102.21, 172.82)	125.23 (97.98, 157.59)	0.07 (−0.22, 0.09)	−0.32 (−0.58–0.06)
**Myocardial disease attributable to high temperature (1990 vs. 2021)**												
Global	0.05 (−0.03, 0.14)	0.06 (−0.02, 0.15)	0.25 (−0.68, 1.75)	1.35 (0.86–1.85)	0.04 (−0.02, 0.12)	0.05 (−0.02, 0.11)	0.12 (−0.84, 1.34)	1.1 (0.61–1.6)	0.06 (−0.03, 0.18)	0.08 (−0.03, 0.21)	0.33 (−0.78, 2.14)	1.48 (0.98–1.98)
High SDI	0.05 (−0.01, 0.13)	0.04 (−0.01, 0.09)	−0.28 (−0.75, 0.03)	−0.43 (−0.92–0.05)	0.04 (−0.01, 0.09)	0.03 (−0.00, 0.06)	−0.36 (−0.76, −0.07)	−0.73 (−1.23–−0.24)	0.07 (−0.02, 0.18)	0.05 (−0.01, 0.12)	−0.27 (−0.75, 0.08)	−0.42 (−0.9–0.06)
High-middle SDI	0.03 (−0.02, 0.10)	0.05 (−0.03, 0.12)	0.36 (−0.65, 1.26)	0.62 (−0.12–1.36)	0.03 (−0.02, 0.09)	0.03 (−0.02, 0.08)	0.09 (−0.69, 1.16)	−0.11 (−0.83–0.61)	0.04 (−0.03, 0.13)	0.06 (−0.04, 0.17)	0.52 (−0.99, 1.43)	0.99 (0.25–1.73)
Middle SDI	0.04 (−0.02, 0.13)	0.06 (−0.01, 0.12)	1.05 (−8.54, 13.22)	1.39 (0.94–1.85)	0.04 (−0.02, 0.09)	0.04 (−0.01, 0.10)	0.84 (−11.59, 6.60)	1.03 (0.57–1.5)	0.05 (−0.02, 0.13)	0.07 (−0.02, 0.16)	1.25 (−7.54, 9.36)	1.7 (1.26–2.15)
Low-middle SDI	0.08 (−0.06, 0.33)	0.14 (−0.05, 0.35)	0.62 (−3.36, 5.54)	2.28 (1.61–2.96)	0.07 (−0.04, 0.22)	0.10 (−0.03, 0.25)	0.52 (−6.41, 14.51)	2.23 (1.53–2.93)	0.10 (−0.06, 0.33)	0.18 (−0.07, 0.47)	0.73 (−2.98, 5.15)	2.4 (1.74–3.06)
Low SDI	0.05 (−0.10, 0.32)	0.10 (−0.08, 0.32)	0.33 (−2.08, 2.72)	3.4 (2.33–4.48)	0.04 (−0.08, 0.24)	0.08 (−0.05, 0.23)	0.15 (−1.97, 2.35)	3.03 (1.97–4.1)	0.06 (−0.12, 0.33)	0.12 (−0.11, 0.42)	0.49 (−1.59, 4.03)	3.71 (2.62–4.83)
**Ischemic heart disease attributable to high temperature (1990 vs. 2021)**												
Global	0.90 (−0.03, 2.46)	1.34 (0.20, 3.07)	0.48 (−1.96, 4.76)	1.66 (1.31–2)	0.72 (−0.04, 1.99)	1.00 (0.15, 2.32)	0.39 (−2.29, 4.59)	1.43 (1.08–1.79)	1.14 (−0.02, 3.01)	1.74 (0.26, 4.01)	0.52 (−1.59, 4.71)	1.78 (1.44–2.13)
High SDI	0.53 (−0.08, 1.89)	0.45 (−0.00, 1.31)	−0.14 (−2.91, 3.65)	−0.25 (−0.67–0.16)	0.39 (−0.06, 1.43)	0.28 (−0.01, 0.85)	−0.28 (−2.21, 2.69)	−0.86 (−1.3 to −0.42)	0.72 (−0.10, 2.55)	0.64 (0.00, 1.83)	−0.11 (−2.46, 4.00)	−0.1 (−0.51–0.31)
High-middle SDI	0.44 (−0.18, 1.76)	0.70 (−0.18, 2.45)	0.61 (−2.81, 4.69)	1.3 (0.77–1.83)	0.35 (−0.14, 1.45)	0.56 (−0.12, 2.01)	0.57 (−3.31, 5.22)	1.15 (0.6–1.7)	0.55 (−0.22, 2.18)	0.89 (−0.26, 3.15)	0.62 (−2.43, 4.25)	1.41 (0.91–1.92)
Middle SDI	0.92 (−0.13, 2.40)	1.51 (0.24, 3.47)	0.64 (−6.96, 5.7)	1.88 (1.53–2.23)	0.76 (−0.15, 2.07)	1.15 (0.18, 2.61)	0.51 (−7.16, 5.63)	1.66 (1.31–2.01)	1.11 (−0.11, 2.82)	1.96 (0.29, 4.43)	0.77 (−9.91, 4.89)	2.1 (1.75–2.44)
Low-middle SDI	2.42 (0.29, 4.71)	3.49 (0.96, 6.57)	0.44 (0.19, 2.04)	1.77 (1.35–2.19)	2.12 (0.22, 4.23)	2.71 (0.74, 5.02)	0.28 (0.03, 1.61)	1.32 (0.88–1.76)	2.72 (0.35, 5.37)	4.37 (1.18, 8.38)	0.61 (0.31, 2.48)	2.18 (1.76–2.6)
Low SDI	1.13 (−0.26, 2.68)	1.71 (0.26, 3.36)	0.51 (−5.61, 8.01)	2.15 (1.61–2.7)	1.04 (−0.31, 2.52)	1.39 (0.19, 2.74)	0.33 (−4.42, 5.73)	1.7 (1.15–2.25)	1.22 (−0.24, 2.93)	2.06 (0.35, 4.09)	0.69 (−5.08, 7.56)	2.56 (2–3.12)
**Myocardial disease attributable to low temperature (1990 vs. 2021)**												
Global	0.52 (0.40, 0.61)	0.27 (0.21, 0.34)	−0.47 (−0.53, −0.41)	−2.34 (−2.52–2.15)	0.44 (0.34, 0.52)	0.19 (0.15, 0.24)	−0.56 (−0.62, −0.50)	−2.84 (−2.99–2.7)	0.60 (0.48, 0.71)	0.36 (0.28, 0.44)	−0.40 (−0.46, −0.33)	−1.96 (−2.17–1.74)
High SDI	0.65 (0.51, 0.73)	0.30 (0.23, 0.34)	−0.46 (−0.52, −0.37)	−2.93 (−3.08–2.78)	0.49 (0.39, 0.56)	0.20 (0.15, 0.23)	−0.57 (−0.63, −0.49)	−3.27 (−3.43–3.12)	0.84 (0.66, 0.95)	0.41 (0.32, 0.46)	−0.39 (−0.46, −0.30)	−2.8 (−2.95–2.64)
High-middle SDI	0.87 (0.63, 1.01)	0.47 (0.36, 0.56)	−0.54 (−0.57, −0.51)	−2.22 (−2.66–1.79)	0.72 (0.52, 0.85)	0.31 (0.23, 0.38)	−0.59 (−0.62, −0.55)	−2.97 (−3.3–2.63)	1.05 (0.78, 1.23)	0.64 (0.50, 0.77)	−0.51 (−0.55, −0.48)	−1.85 (−2.34–1.36)
Middle SDI	0.20 (0.13, 0.29)	0.13 (0.09, 0.19)	−0.06 (−0.25, 0.29)	−1.52 (−1.78–1.26)	0.17 (0.11, 0.25)	0.10 (0.06, 0.15)	−0.16 (−0.37, 0.33)	−1.93 (−2.23–1.63)	0.22 (0.15, 0.33)	0.17 (0.11, 0.24)	0.04 (−0.20, 0.43)	−1.15 (−1.38–0.92)
Low-middle SDI	0.23 (0.07, 0.45)	0.22 (0.07, 0.37)	−0.12 (−0.33, 0.37)	−0.17 (−0.45–0.11)	0.19 (0.05, 0.38)	0.16 (0.05, 0.28)	−0.18 (−0.41, 0.52)	−0.43 (−0.71–0.16)	0.27 (0.08, 0.54)	0.28 (0.10, 0.51)	−0.07 (−0.30, 0.43)	0.09 (−0.2–0.37)
Low SDI	0.23 (0.09, 0.45)	0.20 (0.09, 0.36)	−0.32 (−0.51, −0.15)	−0.38 (−0.77–0)	0.19 (0.06, 0.40)	0.15 (0.06, 0.29)	−0.41 (−0.60, −0.19)	−0.55 (−0.97–0.14)	0.27 (0.11, 0.55)	0.25 (0.11, 0.49)	−0.23 (−0.43, −0.05)	−0.22 (−0.58–0.15)
**Ischemic heart disease attributable to low temperature (1990 vs. 2021)**												
Global	9.74 (8.52, 11.60)	6.14 (5.23, 7.53)	−0.37 (−0.4, −0.33)	−1.76 (−1.88–1.64)	8.28 (7.10, 9.86)	4.85 (4.05, 5.97)	−0.41 (−0.46, −0.37)	−2.03 (−2.15–1.92)	11.45 (10.10, 13.75)	7.71 (6.60, 9.29)	−0.33 (−0.38, −0.27)	−1.5 (−1.62–1.38)
High SDI	10.27 (9.00, 12.35)	3.84 (3.21, 4.60)	−0.63 (−0.64, −0.61)	−3.66 (−3.8–3.51)	7.74 (6.56, 9.32)	2.67 (2.12, 3.25)	−0.65 (−0.68, −0.64)	−3.95 (−4.1–3.8)	13.84 (12.40, 16.58)	5.25 (4.56, 6.24)	−0.62 (−0.64, −0.61)	−3.59 (−3.74–3.44)
High-middle SDI	13.03 (11.64, 15.52)	8.42 (7.17, 10.32)	−0.35 (−0.4, −0.3)	−1.85 (−2.08–1.62)	11.11 (9.85, 13.25)	6.97 (5.70, 8.68)	−0.37 (−0.43, −0.31)	−1.98 (−2.21–1.75)	15.50 (13.88, 18.37)	10.25 (8.85, 12.46)	−0.34 (−0.41, −0.28)	−1.71 (−1.94–1.48)
Middle SDI	7.22 (5.96, 8.63)	6.44 (5.45, 7.62)	−0.11 (−0.2, 0)	−0.32 (−0.49–0.15)	6.32 (5.16, 7.64)	4.97 (4.10, 5.94)	−0.21 (−0.32, −0.09)	−0.72 (−0.88–0.56)	8.22 (6.79, 9.95)	8.27 (6.97, 9.88)	0.01 (−0.13, 0.17)	0.07 (−0.1–0.24)
Low-middle SDI	6.75 (4.99, 8.67)	6.24 (4.79, 8.06)	−0.08 (−0.16, 0.02)	−0.23 (−0.46–0)	6.08 (4.50, 7.80)	4.90 (3.68, 6.29)	−0.19 (−0.28, −0.10)	−0.67 (−0.9–0.44)	7.40 (5.37, 9.60)	7.75 (5.86, 10.25)	0.05 (−0.07, 0.21)	0.18 (−0.06–0.42)
Low SDI	5.29 (4.03, 6.77)	4.79 (3.74, 5.92)	−0.1 (−0.19, 0.03)	−0.3 (−0.61–0.01)	4.68 (3.47, 5.96)	3.78 (2.91, 4.73)	0.19 (−0.30, −0.05)	−0.71 (−1.04–0.39)	5.90 (4.44, 7.63)	5.90 (4.60, 7.40)	0.00 (−0.16, 0.17)	0.07 (−0.23–0.37)

DALYs, Disability-adjusted life years; ASDR, Age-Standardized Death Rate; ASMR, Age-Standardized Mortality Rate; TPC, Total Percent Change; SDI, Sociodemographic Index.

### Gender differences in cardiovascular burden from extreme temperatures

Over the past three decades, men consistently exhibited a higher burden from extreme temperatures than women. For heat-attributable MD, the ASDR increased more rapidly in men (EAPC: 1.47%, 95% CI: 0.97% −1.97%) than in women (EAPC: 0.93%, 95% CI: 0.45% −1.41%), reaching 2.53 vs. 1.42 per 100,000 in 2021, respectively. A similar pattern was observed for heat-attributable IHD, with ASDR EAPCs of 1.83% (95% CI: 1.48% −2.18%) in men and 1.40% (95% CI: 1.04% −1.76%) in women, and 2021 ASDRs of 40.34 vs. 21.65 per 100,000.

In contrast, for cold-attributable burden, both sexes showed decreasing trends, but women experienced steeper declines. For MD, the ASDR EAPC was −2.38% (95% CI: −2.59% to −2.16%) in women vs. −1.58% (95% CI: −1.92% to −1.24%) in men; corresponding 2021 ASDRs were 4.47 in women and 9.62 in men. For IHD, EAPCs were −1.98% (95% CI: −2.10% to −1.86%) in women and −1.55% (95% CI: −1.67% to −1.42%) in men, with ASDRs of 84.78 vs. 153.52 per 100,000, respectively (Figure 1).

### Regional differences in cardiovascular burden from extreme temperatures (1990–2021)

From 1990 to 2021, the heat-attributable CVD burden increased across most socio-demographic index (SDI) tiers, particularly in low-SDI regions. For MD, high-SDI regions showed a slight decline (ASDR EAPC −0.29%), while low-SDI regions experienced significant rises (ASDR EAPC 2.8%). IHD burdens also grew more sharply in lower SDI areas. By 2021, low-middle SDI regions recorded the highest ASDRs for both MD (3.93 per 100,000) and IHD (78.49 per 100,000), contributing 64,292 DALYs for MD and 1,169,778 DALYs for IHD attributable to heat (Figure 1, Table 1).

For low temperatures, burdens declined across all SDI tiers, with the steepest reductions in high-SDI regions (MD: ASDR EAPC −2.93%; IHD: ASDR EAPC −3.66%). Declines were less pronounced in low and low-middle SDI regions. By 2021, middle-high SDI regions had the highest ASDRs for both MD and IHD, contributing ~2,833,786 DALYs for IHD and 234,094 DALYs for MD, highlighting persistent cold-related risks even in relatively developed areas. ASMR trends followed similar patterns across SDI levels (Table 1).

**Figure 2:**
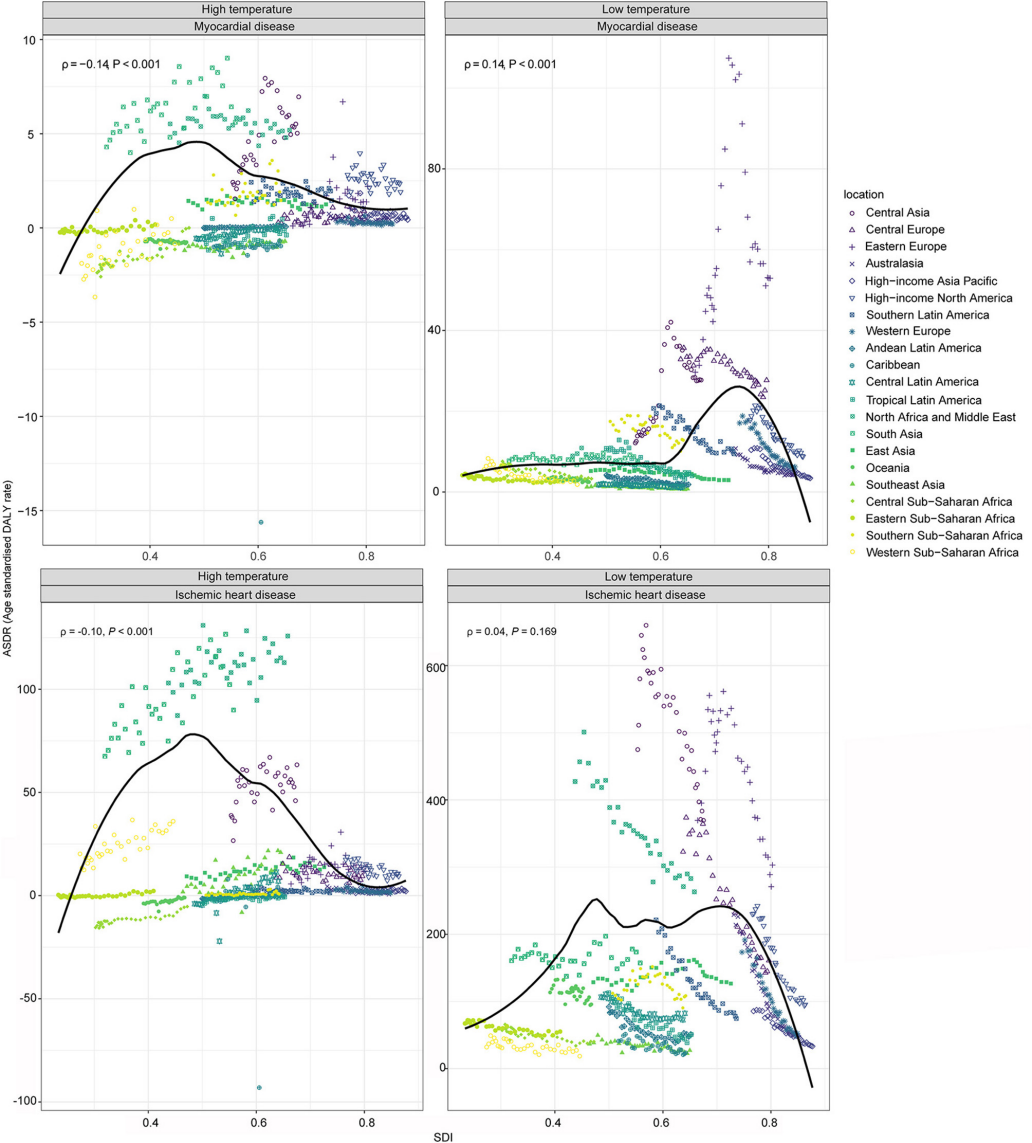
Age-standardized DALYs rate (ASDR) for myocardial disease, and ischemic heart disease attributable to high and low temperatures in 2021, plotted against the sociodemographic index (SDI) for 21 GBD regions. Each dot represents a country, with colors indicating different GBD regions. The dark line was an adaptive association fitted with adaptive Loess regression based on all data points. DALYs, Disability-adjusted life years.

Significant regional variation were noted. For heat-attributable MD, Eastern Europe saw the largest increase (ASDR TPC 20.01%), and for IHD, Southeast Asia had the largest rise (ASDR TPC 7.59%). For cold-attributable MD, only Central Asia and Eastern Europe exhibited slight increases, while other regions declined. By 2021, Central Asia, South Asia, and North Africa/Middle East bore the highest heat-attributable burdens, while Eastern Europe and Central Asia had the highest cold-attributable ASDRs (Figure 2 and Table 2).

**Table 2:** Comprehensive data on DALYs and mortality rates (ASDR, ASMR), and TPC values for myocardial disease, and ischemic heart disease attributable to high and low temperatures across 21 GBD regions in 1990 vs. 2021.

**Region**	**1990 ASDR (95% UI)**	**2021 ASDR (95% UI)**	**TPC of ASDR (95% UI)**	**1990 ASMR (95% UI)**	**2021 ASMR (95% UI)**	**TPC of ASMR (95% UI)**
**Myocardial disease attributable to high temperature**						
Southeast Asia	−1.20 (−1.87, −0.59)	−0.71 (−1.25, −0.02)	−1.2 (−1.87, −0.59)	−0.05 (−0.02, −0.08)	−0.03 (0.01, −0.05)	−0.49 (−1.30, −0.07)
East Asia	1.43 (−0.01, 3.01)	1.25 (−0.01, 2.57)	1.43 (−0.01, 3.01)	0.04 (0.09, −0.00)	0.05 (0.10, −0.00)	0.22 (−0.41, 0.74)
Oceania	−0.72 (−1.42, −0.39)	−0.76 (−1.36, −0.44)	−0.72 (−1.42, −0.39)	−0.02 (−0.01, −0.05)	−0.02 (−0.01, −0.04)	−0.05 (−0.34, 0.36)
Central Asia	2.42 (0.26, 4.63)	6.96 (−0.05, 14.92)	2.42 (0.26, 4.63)	0.07 (0.14, 0.01)	0.23 (0.49, −0.01)	2.12 (−0.25, 2.99)
Central Europe	0.28 (−1.09, 1.96)	0.90 (−1.03, 3.33)	2.2 (−18.75, 9.08)	0.01 (0.10, −0.06)	0.04 (0.15, −0.05)	1.77 (−14.48, 7.92)
Eastern Europe	0.10 (−0.98, 1.19)	2.20 (−4.41, 9.38)	20.01 (−36.74, 87.57)	0.00 (0.04, −0.03)	0.06 (0.25, −0.12)	15.40 (−29.82, 46.80)
High-income Asia Pacific	1.72 (0.04, 3.71)	0.43 (−0.07, 0.99)	−0.75 (−0.98, −0.69)	0.08 (0.17, 0.01)	0.02 (0.04, −0.00)	−0.77 (−0.93, −0.72)
Australasia	0.74 (−0.35, 2.09)	0.22 (−0.13, 0.65)	−0.7 (−1.02, −0.43)	0.03 (0.08, −0.01)	0.01 (0.03, −0.01)	−0.70 (−1.01, −0.41)
Western Europe	0.30 (−0.27, 1.10)	0.21 (−0.04, 0.52)	−0.31 (−2.26, 0.87)	0.02 (0.06, −0.02)	0.01 (0.03, −0.00)	−0.41 (−2.04, 0.66)
Southern Latin America	2.42 (−0.09, 5.15)	1.45 (−0.12, 3.08)	−0.4 (−0.56, −0.22)	0.11 (0.23, −0.00)	0.07 (0.15, −0.01)	−0.36 (−0.53, −0.17)
High-income North America	2.82 (−0.77, 7.00)	1.90 (−0.25, 4.42)	−0.32 (−0.91, 0.04)	0.10 (0.25, −0.03)	0.07 (0.16, −0.01)	−0.32 (−0.83, 0.02)
Caribbean	−1.01 (−2.01, −0.33)	−0.96 (−1.95, −0.04)	−0.05 (−0.83, 0.65)	−0.03 (−0.01, −0.05)	−0.02 (0.01, −0.05)	−0.20 (−1.40, 0.47)
Andean Latin America	0.00 (−0.15, 0.10)	0.06 (−0.03, 0.16)	12.22 (−17.89, 12.38)	−0.00 (0.00, −0.00)	0.00 (0.00, −0.00)	−20.41 (−12.72, 19.29)
Central Latin America	−0.56 (−0.85, −0.39)	−0.01 (−0.35, 0.31)	−0.98 (−1.64, −0.28)	−0.02 (−0.01, −0.03)	−0.00 (0.01, −0.01)	−0.85 (−1.33, −0.28)
Tropical Latin America	−0.45 (−2.46, 2.15)	−0.38 (−1.20, 0.66)	−0.16 (−2.29, 1.53)	−0.01 (0.10, −0.09)	−0.01 (0.03, −0.04)	0.07 (−2.12, 1.04)
North Africa and Middle East	5.52 (−0.16, 11.90)	5.19 (0.31, 10.53)	−0.06 (−0.43, 0.59)	0.16 (0.35, −0.00)	0.16 (0.33, 0.01)	0.01 (−0.33, 0.73)
South Asia	4.29 (−1.01, 10.49)	5.61 (−0.71, 12.39)	0.31 (−0.48, 1.27)	0.15 (0.39, −0.03)	0.20 (0.46, −0.03)	0.30 (−0.19, 1.19)
Central Sub-Saharan Africa	−2.38 (−11.60, 0.16)	0.00 (−2.84, 2.41)	−1.0 (−10.64, 7.16)	−0.09 (0.01, −0.40)	0.00 (0.10, −0.11)	−1.01 (−10.05, 8.47)
Eastern Sub-Saharan Africa	−0.23 (−1.03, 0.72)	0.18 (−0.83, 1.39)	−1.78 (−9.14, 12.14)	−0.01 (0.02, −0.03)	0.01 (0.04, −0.02)	−2.10 (−14.72, 11.49)
Southern Sub-Saharan Africa	1.35 (−0.59, 3.49)	1.46 (−0.52, 3.72)	0.08 (−0.22, 0.59)	0.06 (0.15, −0.03)	0.06 (0.15, −0.02)	0.01 (−0.31, 0.50)
Western Sub-Saharan Africa	−1.88 (−8.96, 9.20)	−0.85 (−6.25, 7.08)	−0.55 (−4.96, 3.31)	−0.08 (0.34, −0.36)	−0.03 (0.27, −0.24)	−0.57 (−5.56, 2.99)
**Ischemic heart disease attributable to high temperature**						
Southeast Asia	1.79 (12.10, −16.45)	15.37 (24.22, 7.73)	7.59 (−24.80, 14.62)	0.09 (0.59, −0.69)	0.78 (1.26, 0.36)	7.29 (−27.84, 12.55)
East Asia	9.34 (35.46, −7.01)	13.75 (49.90, −5.48)	0.47 (−3.42, 2.73)	0.52 (1.96, −0.39)	0.85 (3.08, −0.33)	0.63 (−3.89, 2.87)
Oceania	−3.82 (−0.62, −14.97)	−0.94 (1.09, −6.44)	−0.75 (−2.23, −0.10)	−0.17 (−0.03, −0.64)	−0.04 (0.05, −0.28)	−0.76 (−2.09, −0.07)
Central Asia	38.82 (130.92, −1.73)	63.34 (178.90, 9.67)	0.63 (−3.96, 7.47)	2.03 (6.88, −0.09)	3.44 (9.65, 0.51)	0.69 (−4.07, 7.66)
Central Europe	8.65 (37.12, −2.62)	9.40 (36.14, −0.72)	0.09 (−4.58, 5.48)	0.46 (2.00, −0.14)	0.54 (2.06, −0.04)	0.17 (−4.9, 5.66)
Eastern Europe	5.62 (23.90, −1.32)	14.08 (52.05, 0.48)	1.50 (−15.00, 26.14)	0.31 (1.33, −0.07)	0.78 (2.88, 0.03)	1.49 (−11.86, 20.58)
High-income Asia Pacific	5.42 (20.55, −2.09)	2.08 (7.80, −0.64)	−0.62 (−0.83, −0.46)	0.32 (1.20, −0.12)	0.11 (0.42, −0.03)	−0.65 (−0.83, −0.53)
Australasia	4.43 (17.99, −2.08)	0.63 (3.07, −0.88)	−0.86 (−1.93, 0.30)	0.25 (1.01, −0.11)	0.04 (0.19, −0.05)	−0.85 (−2.13, 0.25)
Western Europe	2.73 (12.69, −0.86)	1.27 (5.66, −0.33)	−0.53 (−1.20, 0.58)	0.15 (0.71, −0.04)	0.08 (0.34, −0.02)	−0.5 (−1.06, 0.63)
Southern Latin America	4.22 (17.80, −4.12)	1.34 (6.68, −2.23)	−0.68 (−1.49, 0.98)	0.22 (0.95, −0.22)	0.07 (0.35, −0.12)	−0.69 (−1.48, 0.95)
High-income North America	17.40 (65.05, −3.28)	9.41 (34.40, −2.44)	−0.46 (−1.07, 0.36)	0.93 (3.51, −0.17)	0.50 (1.84, −0.13)	−0.46 (−1.12, 0.36)
Caribbean	−0.53 (4.45, −11.31)	1.31 (4.06, −1.69)	−3.45 (−5.94, 4.78)	0.02 (0.24, −0.46)	0.09 (0.22, −0.04)	3.64 (−6.03, 3.23)
Andean Latin America	−1.50 (−0.37, −3.55)	−0.02 (1.15, −1.12)	−0.98 (−3.24, −0.39)	−0.08 (−0.02, −0.18)	−0.00 (0.06, −0.06)	−0.96 (−3.04, −0.38)
Central Latin America	−3.99 (3.24, −13.60)	6.98 (14.58, −0.19)	−2.75 (−25.08, 17.33)	−0.21 (0.16, −0.70)	0.35 (0.73, −0.01)	−2.64 (−21.64, 13.9)
Tropical Latin America	−3.87 (8.56, −13.95)	−0.62 (4.98, −4.84)	−0.84 (−2.20, 1.30)	−0.18 (0.42, −0.66)	−0.02 (0.23, −0.22)	−0.86 (−2.03, 0.97)
North Africa and Middle East	103.00 (240.92, 7.94)	125.79 (274.00, 23.92)	0.22 (0.04, 1.13)	4.87 (11.48, 0.33)	5.97 (13.07, 1.09)	0.23 (0.06, 1.28)
South Asia	67.51 (124.51, 11.81)	89.91 (160.49, 25.58)	0.33 (0.12, 1.50)	2.91 (5.41, 0.47)	3.98 (7.06, 1.16)	0.37 (0.16, 1.53)
Central Sub-Saharan Africa	−15.47 (−2.39, −71.15)	−5.35 (0.99, −18.16)	−0.65 (−1.27, 0.55)	−0.74 (−0.12, −3.42)	−0.26 (0.04, −0.90)	−0.64 (−1.22, 0.58)
Eastern Sub-Saharan Africa	0.33 (5.20, −6.17)	2.24 (8.99, −2.73)	5.87 (−9.26, 10.08)	0.01 (0.23, −0.30)	0.10 (0.41, −0.12)	11.06 (−12.27, 17.17)
Southern Sub-Saharan Africa	0.25 (5.18, −3.53)	0.75 (7.01, −4.27)	2.04 (−3.66, 2.83)	0.01 (0.25, −0.17)	0.04 (0.34, −0.21)	1.9 (−2.76, 2.79)
Western Sub-Saharan Africa	20.19 (55.24, −21.05)	36.01 (65.21, 8.49)	0.78 (−8.20, 11.38)	1.01 (2.71, −1.02)	1.89 (3.42, 0.48)	0.87 (−8.49, 12.39)
**Myocardial disease attributable to low temperature**						
Southeast Asia	1.22 (2.14, 0.46)	0.75 (1.29, 0.24)	−0.39 (−0.71, −0.06)	0.06 (0.10, 0.02)	0.04 (0.06, 0.01)	−0.40 (−0.68, −0.04)
East Asia	5.19 (7.18, 3.88)	2.94 (3.94, 1.53)	−0.43 (−0.69, −0.21)	0.13 (0.20, 0.10)	0.11 (0.15, 0.05)	−0.19 (−0.58, 0.15)
Oceania	3.13 (4.92, 0.58)	2.47 (4.36, −0.40)	−0.21 (−0.94, 0.22)	0.09 (0.14, 0.02)	0.07 (0.12, −0.01)	−0.24 (−0.92, 0.21)
Central Asia	12.13 (15.03, 9.75)	27.71 (34.84, 20.94)	1.28 (0.80, 1.86)	0.39 (0.48, 0.31)	0.94 (1.17, 0.73)	1.40 (0.91, 2.01)
Central Europe	32.83 (37.39, 26.94)	27.66 (32.33, 21.64)	−0.16 (−0.26, −0.03)	1.69 (1.92, 1.38)	1.27 (1.46, 1.02)	−0.25 (−0.32, −0.15)
Eastern Europe	29.65 (34.61, 21.56)	52.94 (65.91, 38.44)	0.79 (0.50, 1.16)	0.91 (1.06, 0.67)	1.48 (1.83, 1.09)	0.63 (0.36, 0.99)
High-income Asia Pacific	10.41 (12.11, 7.56)	3.42 (3.94, 2.49)	−0.67 (−0.71, −0.64)	0.45 (0.53, 0.33)	0.14 (0.16, 0.10)	−0.69 (−0.72, −0.66)
Australasia	10.32 (14.18, 0.87)	4.21 (5.69, 1.47)	−0.59 (−0.69, −0.27)	0.38 (0.53, 0.02)	0.16 (0.21, 0.05)	−0.58 (−0.69, −0.25)
Western Europe	17.07 (19.87, 13.16)	6.21 (7.22, 4.53)	−0.64 (−0.68, −0.60)	0.91 (1.07, 0.70)	0.30 (0.35, 0.22)	−0.68 (−0.71, −0.65)
Southern Latin America	20.68 (28.21, 12.09)	9.44 (11.31, 5.98)	−0.54 (−0.64, −0.41)	0.89 (1.24, 0.52)	0.44 (0.52, 0.28)	−0.51 (−0.61, −0.37)
High-income North America	17.79 (21.28, 12.49)	8.77 (10.51, 6.52)	−0.51 (−0.55, −0.46)	0.64 (0.77, 0.45)	0.33 (0.39, 0.24)	−0.48 (−0.53, −0.42)
Caribbean	1.85 (2.79, 1.01)	1.75 (2.52, 1.06)	−0.06 (−0.24, 0.20)	0.06 (0.08, 0.03)	0.06 (0.08, 0.04)	0.06 (−0.12, 0.32)
Andean Latin America	4.19 (5.30, 2.24)	1.84 (2.43, 0.90)	−0.56 (−0.68, −0.40)	0.12 (0.15, 0.06)	0.06 (0.08, 0.03)	−0.53 (−0.66, −0.37)
Central Latin America	1.79 (2.52, 0.89)	1.29 (1.87, −0.01)	−0.28 (−0.91, 0.04)	0.06 (0.08, 0.03)	0.04 (0.06, −0.00)	−0.33 (−0.95, −0.04)
Tropical Latin America	10.79 (16.50, 5.32)	4.77 (7.41, 1.71)	−0.56 (−0.70, −0.46)	0.44 (0.68, 0.22)	0.18 (0.29, 0.06)	−0.59 (−0.73, −0.49)
North Africa and Middle East	8.92 (15.50, 4.46)	3.98 (6.66, 2.28)	−0.55 (−0.69, −0.35)	0.26 (0.45, 0.13)	0.14 (0.23, 0.08)	−0.48 (−0.64, −0.27)
South Asia	7.72 (15.41, 1.94)	7.57 (13.60, 2.32)	−0.02 (−0.22, 0.44)	0.27 (0.56, 0.07)	0.28 (0.51, 0.09)	0.02 (−0.18, 0.53)
Central Sub-Saharan Africa	6.10 (12.03, 0.30)	3.28 (6.55, −0.15)	−0.46 (−0.73, 0.08)	0.23 (0.47, 0.01)	0.13 (0.26, −0.01)	−0.44 (−0.73, 0.11)
Eastern Sub-Saharan Africa	4.13 (6.98, −1.85)	2.64 (4.31, −0.47)	−0.36 (−0.79, 0.10)	0.12 (0.20, −0.06)	0.08 (0.13, −0.01)	−0.33 (−0.78, 0.18)
Southern Sub-Saharan Africa	17.46 (23.97, 9.05)	12.82 (17.25, 5.47)	−0.27 (−0.47, −0.07)	0.78 (1.11, 0.41)	0.55 (0.74, 0.24)	−0.30 (−0.51, −0.04)
Western Sub-Saharan Africa	5.53 (11.73, 1.23)	1.85 (3.88, 0.38)	−0.67 (−0.78, −0.50)	0.22 (0.46, 0.05)	0.07 (0.15, 0.01)	−0.68 (−0.79, −0.52)
**Ischemic heart disease attributable to low temperature**						
Southeast Asia	36.57 (47.36, 28.07)	27.22 (34.00, 21.09)	−0.26 (−0.37, −0.10)	1.69 (2.19, 1.28)	1.34 (1.68, 1.02)	−0.21 (−0.32, −0.04)
East Asia	128.67 (153.61, 106.07)	126.70 (155.49, 103.38)	−0.02 (−0.19, 0.22)	6.97 (8.30, 5.74)	7.73 (9.44, 6.32)	0.11 (−0.09, 0.36)
Oceania	113.36 (148.65, 86.71)	95.73 (126.55, 71.51)	−0.16 (−0.37, 0.15)	4.69 (6.17, 3.70)	4.04 (5.32, 3.05)	−0.14 (−0.34, 0.14)
Central Asia	474.93 (587.27, 417.21)	369.53 (457.30, 320.13)	−0.22 (−0.29, −0.15)	24.80 (30.93, 21.58)	20.34 (25.08, 17.53)	−0.18 (−0.25, −0.11)
Central Europe	322.86 (389.68, 282.78)	160.96 (202.94, 139.80)	−0.50 (−0.54, −0.47)	17.24 (20.94, 15.03)	9.40 (11.86, 8.10)	−0.45 (−0.49, −0.42)
Eastern Europe	369.56 (447.59, 337.96)	303.08 (385.85, 259.67)	−0.18 (−0.27, −0.09)	20.16 (24.44, 18.07)	16.55 (21.11, 14.14)	−0.18 (−0.27, −0.09)
High-income Asia Pacific	85.55 (95.94, 75.86)	33.25 (37.41, 28.70)	−0.61 (−0.63, −0.59)	5.10 (5.74, 4.38)	1.83 (2.10, 1.49)	−0.64 (−0.66, −0.62)
Australasia	229.23 (266.92, 207.62)	52.79 (61.92, 46.04)	−0.77 (−0.79, −0.76)	12.73 (14.87, 11.32)	3.18 (3.78, 2.69)	−0.75 (−0.77, −0.73)
Western Europe	173.43 (206.66, 153.18)	52.42 (61.12, 45.15)	−0.70 (−0.72, −0.68)	9.56 (11.41, 8.27)	3.10 (3.65, 2.57)	−0.68 (−0.7, −0.66)
Southern Latin America	221.26 (249.58, 205.66)	74.54 (81.60, 68.16)	−0.66 (−0.69, −0.64)	11.85 (13.43, 10.78)	3.89 (4.28, 3.48)	−0.67 (−0.7, −0.65)
High-income North America	230.18 (279.34, 205.25)	95.43 (113.16, 84.48)	−0.59 (−0.60, −0.57)	12.36 (15.01, 10.65)	5.06 (6.04, 4.24)	−0.59 (−0.61, −0.57)
Caribbean	41.03 (51.50, 33.00)	23.63 (28.90, 19.16)	−0.42 (−0.51, −0.31)	2.14 (2.67, 1.73)	1.15 (1.41, 0.94)	−0.46 (−0.53, −0.37)
Andean Latin America	91.97 (105.45, 74.41)	51.29 (64.45, 40.21)	−0.44 (−0.54, −0.31)	4.70 (5.35, 3.81)	2.70 (3.37, 2.15)	−0.42 (−0.52, −0.29)
Central Latin America	106.57 (122.20, 94.03)	82.31 (98.63, 71.58)	−0.23 (−0.32, −0.11)	5.62 (6.44, 4.95)	4.36 (5.22, 3.77)	−0.22 (−0.31, −0.1)
Tropical Latin America	112.87 (135.37, 92.02)	50.86 (60.69, 41.78)	−0.55 (−0.57, −0.51)	5.34 (6.41, 4.28)	2.31 (2.77, 1.89)	−0.57 (−0.59, −0.53)
North Africa and Middle East	427.36 (535.84, 347.58)	263.22 (331.05, 221.31)	−0.38 (−0.45, −0.30)	20.81 (26.06, 16.87)	13.71 (17.08, 11.52)	−0.34 (−0.4, −0.26)
South Asia	160.64 (212.70, 110.26)	157.44 (208.52, 105.90)	−0.02 (−0.14, 0.11)	6.86 (9.18, 4.71)	6.97 (9.19, 4.66)	0.01 (−0.11, 0.15)
Central Sub-Saharan Africa	57.07 (77.66, 40.31)	40.15 (52.74, 29.22)	−0.30 (−0.47, −0.02)	2.77 (3.69, 1.96)	2.00 (2.65, 1.46)	−0.28 (−0.45, −0.01)
Eastern Sub-Saharan Africa	67.17 (82.03, 52.65)	55.21 (68.16, 44.94)	−0.18 (−0.34, 0.01)	3.04 (3.73, 2.41)	2.69 (3.29, 2.16)	−0.12 (−0.27, 0.07)
Southern Sub-Saharan Africa	112.08 (130.59, 92.72)	108.45 (123.45, 95.74)	−0.03 (−0.13, 0.11)	5.38 (6.31, 4.42)	5.53 (6.30, 4.86)	0.03 (−0.08, 0.19)
Western Sub-Saharan Africa	32.03 (47.54, 18.52)	18.49 (25.92, 11.94)	−0.42 (−0.52, −0.27)	1.63 (2.40, 0.97)	0.97 (1.35, 0.64)	−0.41 (−0.5, −0.27)

DALYs, disability-adjusted life years; ASDR, age-standardized death rate; ASMR, age-standardized mortality rate; TPC, total percent change; GBD, global burden of disease.

Temperature-related burdens followed SDI patterns: high-temperature burdens increased in regions with SDI below 0.5, but declined above this threshold. For cold-attributable IHD, burdens increased sharply between SDI 0.6 and 0.75 and then declining at higher SDI levels. For cold-attributable MD, burdens increased below SDI 0.45, fluctuated between 0.45 and 0.75, and declined beyond 0.75 (Figure 2).

### National differences in cardiovascular burden from extreme temperatures

**Figure 3:**
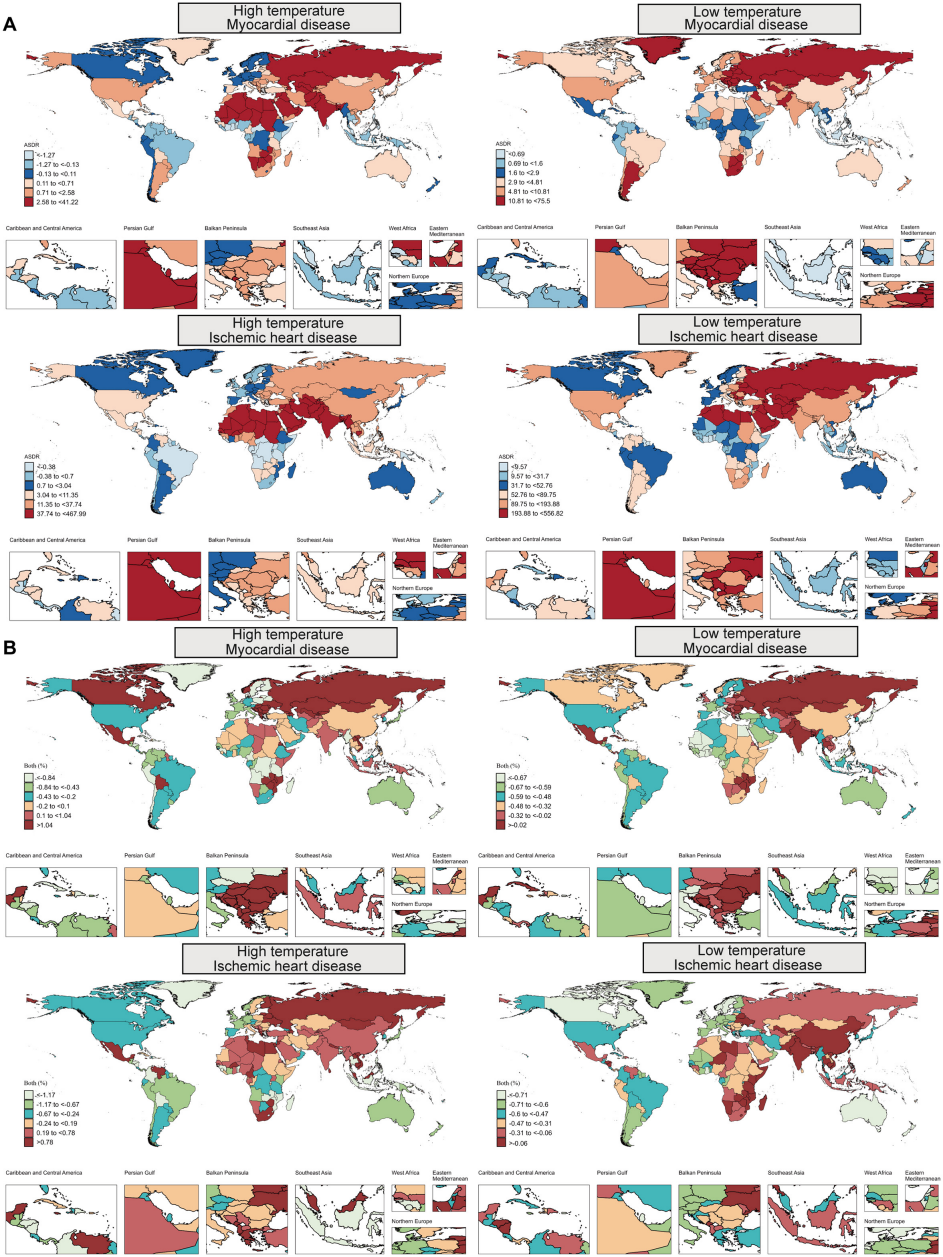
**(A)** Age-standardized DALY rates (ASDR) and **(B)** total percentage change (TPC) for myocardial disease, as well as ischemic heart disease, attributable to high and low temperatures across countries in 2021. DALYs, Disability-adjusted life years.

Between 1990 and 2021, heat-attributable cardiovascular trends varied widely by country. MD burden rose in 38% of countries, led by Kazakhstan (total percentage change, TPC = +45.84%), whereas Bangladesh showed the largest decline (−104.11%). For IHD, 39% of countries registered increases, headed by Jamaica (+33.78%), with Zambia recording the greatest reduction (−44.36%). In 2021, Turkmenistan had the highest heat-attributable MD burden (ASDR 41.22; ASMR 1.22 per 100,000) and Iraq the highest IHD burden (ASDR 467.99; ASMR 24.70 per 100,000; Figure 3; Supplementary Tables S1, S2).

For cold-attributable burden, most countries improved: only ~15% saw increases. Kazakhstan again recorded the largest MD rise (+17.59%), whereas Italy showed the steepest fall (−0.87%). In 2021 Kazakhstan also had the highest cold-attributable MD burden (ASDR 75.50; ASMR 2.65 per 100,000). For IHD, Kiribati posted the greatest increase (+7.97%), while the Maldives showed the largest drop (−0.88%). Turkmenistan remained highest for cold-attributable IHD (ASDR 556.82; ASMR 29.72 per 100,000). The Cook Islands and Marshall Islands had the lowest burdens for both diseases.

### Age and sex patterns in 2021

Heat-attributable burden: MD ASDR rose steadily with age—from 3.93 (60–64 years) to 7.40 (80–84 years), peaking at 18.26 per 100 000 beyond 95 years; children < 5 year were also vulnerable (3.45). Heat-attributable IHD rose sharply from mid-life, reaching 320.06 per 100,000 beyond 95 years.

**Figure 4:**
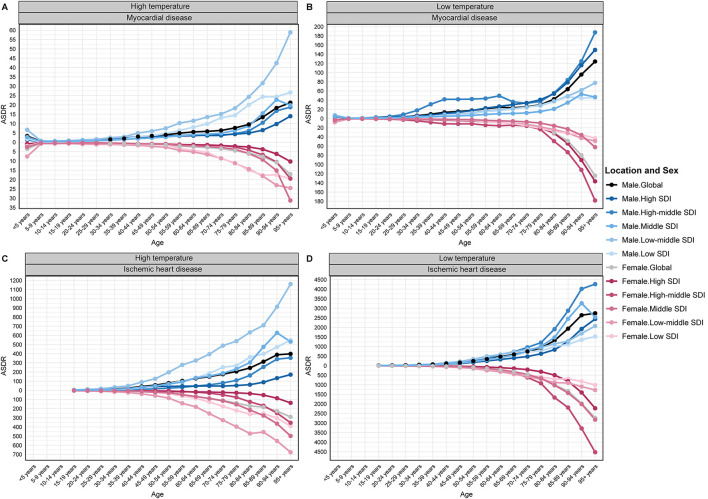
Global and SDI-stratified distribution of ASDR by 5-year age groups in 2021 for temperature-attributable myocardial and ischemic heart disease: **(A)** myocardial disease–high temperatures; **(B)** myocardial disease–low temperatures; **(C)** ischemic heart disease–high temperatures; **(D)** ischemic heart disease–low temperatures. ASDR, age-standardized DALY rate; SDI, Socio-demographic Index.

Cold-attributable burden: MD ASDR climbed from 16.58 (60–64 years) to 35.69 (80–84 years) and 124.39 beyond 95 year, with children < 5 year again relatively affected (5.23). Cold-attributable IHD accelerated after 80 years, rising from 340.03 (60–64 years) to 1 130.74 (80–84 years) and 2 734.64 per 100,000 beyond 95 years (Figure 4; Supplementary Tables S3–S10).

### Potential risk factors

**Figure 5:**
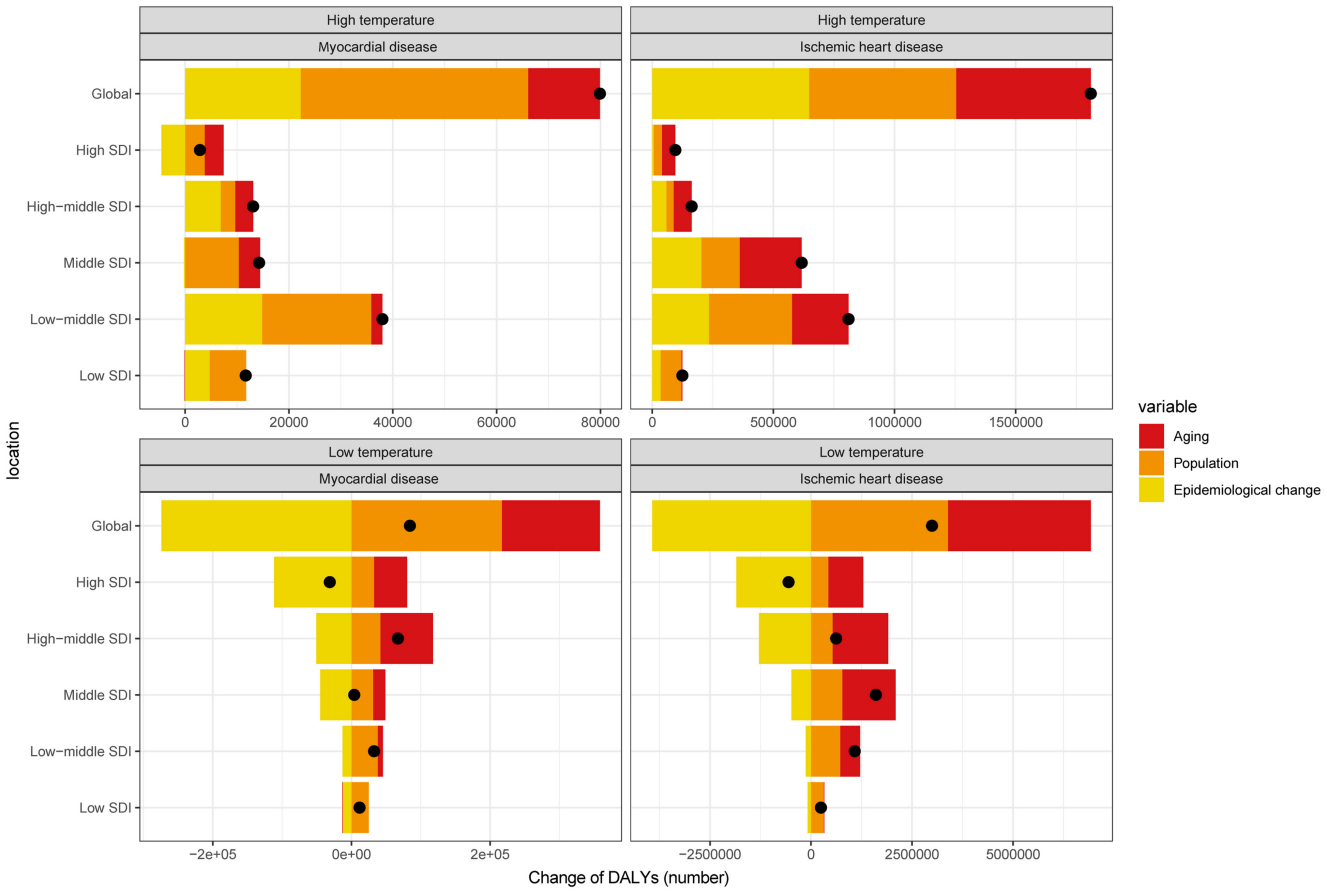
Changes in DALYs for myocardial disease, and ischemic heart disease attributable to high and low temperatures, driven by population-level determinants: aging, population growth, and epidemiological change, from 1990 to 2021, globally and across SDI quintiles. The black dot represents the overall change contributed by all three components combined. Positive values indicate an increase in DALYs attributable to the respective component, while negative values indicate a decrease.

The three main contributors to the increased global burden of MD and IHD attributable to non-optimal temperatures were aging, population change, and epidemiological progress. For heat-attributable MD, population change contributed 54.79% to the DALY increase, with epidemiological shifts accounting for 27.87% and aging for 17.34%. For heat-attributable IHD, aging accounted for 30.70%, population growth for 33.53%, and epidemiological change for 35.77%. Cold-attributable diseases, aging and population change were the dominant factors, especially in low-SDI regions where population growth was the primary driver (Figure 5 and Supplementary Table S11).

### Predicted trends

**Figure 6:**
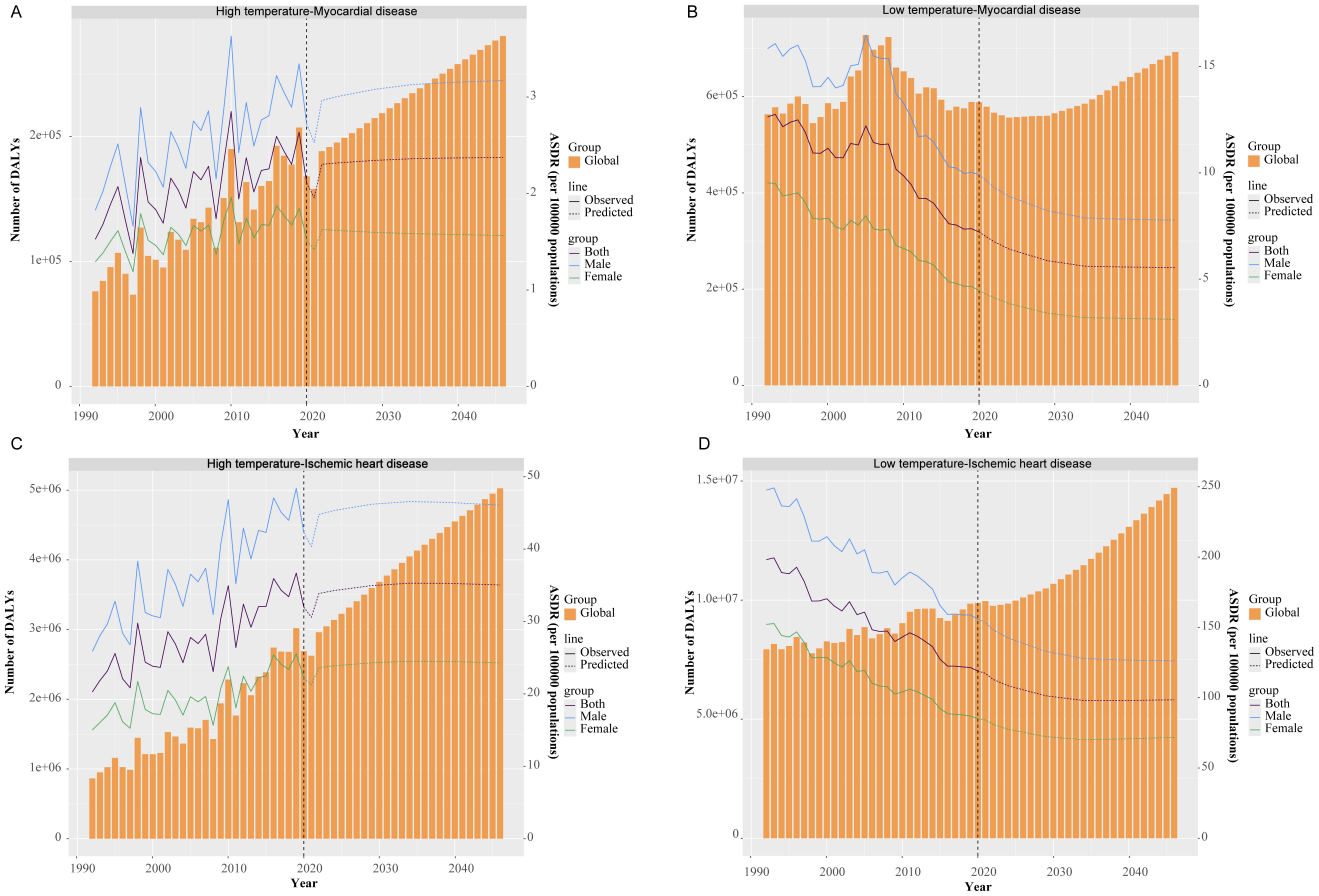
Temporal trends in ASDR at the global level for temperature-attributable myocardial and ischemic heart disease, with projections to 2040: **(A)** myocardial disease–high temperatures; **(B)** myocardial disease–low temperatures; **(C)** ischemic heart disease–high temperatures; **(D)** ischemic heart disease–low temperatures. Solid lines indicate observed rates; dashed lines indicate predicted rates. ASDR, age-standardized DALY rate.

From 2021 to 2040, the global heat-attributable burden of MD and IHD is projected to rise. MD DALYs and mortality are expected to increase steadily, with the global ASDR reaching 2.37 per 100,000 and the ASMR 0.077 per 100,000 (≈10,117 deaths) by 2040. For IHD, the heat-attributable ASDR is projected to climb to 35.24 per 100,000 and the ASMR to 1.523 per 100,000 (≈215,318 deaths), peaking around 2034 before a slight decline (Figure 6; Supplementary Table S12).

For cold-attributable burden, global DALYs and mortality are projected to continue declining. By 2040, the cold-attributable ASDR for MD is expected to fall to 5.56 per 100,000, with an ASMR of 0.217 per 100,000 (≈29,422 deaths). For IHD, the ASDR is forecast to decrease to 137.18 per 100,000, with an ASMR of 5.193 per 100,000 (≈756,304 deaths; Figure 6; Supplementary Table S12).

## Discussion

### Global trends and driving factors

Our study confirms that cold temperatures still account for a greater share of global cardiovascular mortality compared to heat. However, while cold-related burdens have steadily decreased, heat-related burdens have continued to rise since 1990. This divergence parallels the accelerating pace of anthropogenic warming and intensified urban heat-island effects highlighted by the IPCC and Lancet Countdown reports (23, 24). Additionally, incomplete classification and underreporting of heat-related deaths, particularly in regions with inadequate mortality registration systems, may further underestimate the true impact (10).

### Disease*-*specific observations and mechanisms

Our findings highlight distinct differences between IHD and MD in their susceptibility and mechanisms underlying temperature-related cardiovascular burdens. IHD demonstrates particular vulnerability to high-temperature exposures through mechanisms including increased vascular inflammation, plaque instability, hypercoagulability, and dehydration-induced hemoconcentration, all exacerbating existing atherosclerotic conditions (25). In contrast, MD—encompassing diverse etiologies such as viral myocarditis, autoimmune reactions, and genetic cardiomyopathies—is typically exacerbated by heat-induced systemic inflammation, oxidative stress, and direct myocardial injury rather than primarily through vascular or thrombotic pathways (26, 27). These pathophysiological differences help explain why IHD and MD show distinct epidemiological trends under temperature extremes and emphasize the need for disease-specific prevention and management strategies.

### Mechanisms of age and gender vulnerabilities

The age-related vulnerabilities to extreme temperatures involve specific physiological mechanisms. Children under five exhibit heightened sensitivity due to immature thermoregulatory systems and diagnostic delays (28, 29). Conversely, older adults face increased risks from impaired vasodilation, reduced sweating capacity, and diminished autonomic control exacerbated by chronic conditions like diabetes and hypertension (30). Gender disparities across socioeconomic development indices (SDI) showed men consistently having higher burdens, likely due to occupational exposure, lifestyle factors (e.g., smoking, alcohol), and less protective estrogenic vascular effects compared to women (31–33).

### Spatiotemporal patterns and socioeconomic factors

High-temperature burdens have risen fastest in low-SDI regions, where weak health systems, minimal cooling infrastructure, and widespread outdoor work intensify exposure. Each 1°C rise in temperature is associated with an ~9% increase in cardiovascular mortality in lower-middle-income countries compared to high-income settings (34). While high-SDI regions have largely stabilized or reduced heat- and cold-attributable burdens through better housing, air-conditioning, and heat-alert systems, several upper-middle-SDI economies are now seeing renewed increases, underscoring that economic growth alone does not guarantee resilience (35, 36).

Against this broader pattern, Eastern Europe has experienced the steepest rise in heat-attributable MD (ASDR +20%), likely driven by high rates of alcohol-related cardiomyopathy (37), rapid increases in extreme-heat days coupled with low air-conditioning coverage, and population aging combined with high smoking and hypertension prevalence (38, 39). Southeast Asia records the largest surge in heat-related IHD (ASDR +7.6%), reflecting urban heat islands, limited affordable cooling, and increasing rates of hypertension and diabetes (19, 40). Although cold-attributable MD has declined globally, Central Asia and Eastern Europe still show slight increases, likely due to poorly insulated housing, low indoor winter temperatures, and persistent high smoking rates (41). By 2021, heat-related cardiovascular mortality peaks had shifted to Central Asia, South Asia, and North Africa/Middle East, where ≥40°C heatwaves coincide with limited cooling infrastructure, whereas cold-related peaks remained concentrated in Eastern Europe and Central Asia (42).

Overall, heat-related mortality has been rising while cold-related mortality has decreased. Yet, three exceptional seasons disrupted these trends: the western Russia heatwave in 2010 (43) and the trans-Eurasian heatwave of 2020 (44) each triggered significant spikes in cardiovascular deaths, briefly reversing earlier gains from milder summers, heat-action plans, and expanded cooling access. Conversely, the 2005 winter saw an unusual cold-related spike linked to a strong negative Arctic Oscillation (45, 46), a resurgence of solid-fuel heating, and an influenza B outbreak (47). These episodes illustrate how single-season extremes can temporarily override long-term trends, and how recent adaptation gains are increasingly challenged by rising heat intensity and an aging global population.

### Comparisons with previous studies

Our findings extend previous work by providing a disease-specific breakdown of temperature-related cardiovascular burdens. Unlike earlier multi-country studies that aggregated all cardiovascular outcomes (48, 49), our analysis separately quantified IHD and MD burdens across diverse demographics and socioeconomic contexts. Previous GBD analyses primarily considered older populations without detailed sex or regional stratification (50), whereas our study highlights vulnerabilities across all ages and explicitly quantifies gender-specific risks. Additionally, previous single-country studies noted seasonally increased cardiomyopathy events without comprehensive mortality data (51, 52); our global perspective fills this gap and underscores sustained heat-related increases in MD. Our future projections, which incorporate updated demographic and epidemiological data, contrast with prior optimistic forecasts based solely on technological adaptation (53, 54).

### Decomposition analysis insights

Our decomposition analysis revealed that population growth significantly contributed (over 54% for MD and 30% for IHD) to rising heat-related cardiovascular burdens, particularly in rapidly urbanizing lower-SDI regions (55). Population aging accounted substantially for both MD and IHD burden increases (17.3 and 30.7%, respectively), reflecting age-related physiological vulnerabilities and chronic disease prevalence (30, 56). Epidemiological transitions, especially increased prevalence of obesity and diabetes, contributed more significantly to IHD (35.8%) compared to MD (27.9%), highlighting IHD's stronger association with modifiable risk factors (57). The declining cold-related burdens primarily reflect improved housing, heating infrastructure, and healthcare, though aging populations still sustain significant residual risks.

### Strengths and limitations

This study provides the first comprehensive, disease-specific global assessment of temperature-related cardiovascular burdens, incorporating long-term trend analyses and future projections with decomposition of demographic and epidemiological drivers. However, it shares inherent limitations associated with GBD methodologies, including potential misclassification of deaths, incomplete data coverage (particularly in low-SDI settings), and limited assessment of delayed temperature effects or adaptation behaviors. Future research should address these gaps by integrating finer-scale data and evaluating the impacts of adaptation measures more systematically.

## Conclusions and implications

Heat-related IHD represents an increasingly critical global health threat, while MD presents unique vulnerabilities, particularly for young children and older adults. Effective public health interventions must prioritize age-, gender-, and socioeconomic-specific strategies, such as enhancing heat-resilient infrastructure, improving chronic disease management during heatwaves, and targeted interventions addressing occupational and lifestyle risks. Comprehensive, tailored climate-adaptive cardiovascular planning is essential to mitigate growing temperature-related health burdens worldwide.

## Data Availability

The original contributions presented in the study are included in the article/Supplementary material, further inquiries can be directed to the corresponding authors.

## Supplementary material

Supplementary Table S1 Comprehensive data on DALYs and mortality rates (ASDR, ASMR), and TPC values for myocardial disease attributable to high and low temperatures across countries in 1990 vs. 2021.

Supplementary Table S2 Comprehensive data on DALYs and mortality rates (ASDR, ASMR), and TPC values for ischemic heart disease attributable to high and low temperatures across countries in 1990 vs. 2021.

Supplementary Table S3 Age-specific DALYs and ASDR for myocardial disease attributable to high temperature, categorized by 5-year age groups, presented globally and by SDI region in 2021.

Supplementary Table S4 Age-specific DALYs and ASDR for myocardial disease attributable to low temperature, categorized by 5-year age groups, presented globally and by SDI region in 2021.

Supplementary Table S5 Age-specific DALYs and ASDR for ischemic heart disease attributable to high temperature, categorized by 5-year age groups, presented globally and by SDI region in 2021.

Supplementary Table S6 Age-specific DALYs and ASDR for ischemic heart disease attributable to low temperature, categorized by 5-year age groups, presented globally and by SDI region in 2021.

Supplementary Table S7 Age-specific death numbers and ASMR for myocardial disease attributable to high temperature, categorized by 5-year age groups, presented globally and by SDI region in 2021.

Supplementary Table S8 Age-specific death numbers and ASMR for myocardial disease attributable to low temperature, categorized by 5-year age groups, presented globally and by SDI region in 2021.

Supplementary Table S9 Age-specific death numbers and ASMR for ischemic heart disease attributable to high temperature, categorized by 5-year age groups, presented globally and by SDI region in 2021.

Supplementary Table S10 Age-specific death numbers and ASMR for ischemic heart disease attributable to low temperature, categorized by 5-year age groups, presented globally and by SDI region in 2021.

Supplementary Table S11 Changes in DALYs and deaths for myocardial disease, and ischemic heart disease attributable to high and low temperatures from 1990 to 2021, categorized globally and by SDI regions.

Supplementary Table S12 Age-standardized DALYs and mortality rates (ASDR, ASMR) for cardiomyopathy, myocarditis, and ischemic heart disease related to high and low temperatures, from 1992 to projected estimates for 2040.
