# Supplementary material for: Comparative global burden of ischemic heart disease and myocardial disease attributable to non-optimal temperatures, 1990–2021: an analysis based on GBD 2021
Source: Front Public Health. 2025 Sep 23;13:1605624. doi: 10.3389/fpubh.2025.1605624 (PMC12500565; doi:10.3389/fpubh.2025.1605624)
Supplement: Supplementary Table S1 — Comprehensive data on DALYs and mortality rates (ASDR, ASMR), and TPC values for myocardial disease attributable to high and low temperatures across countries in 1990 vs. 2021. [file Table_1.docx]

| **Supplementary Table Legend**  **Table S1. Comprehensive data on DALYs and mortality rates (ASDR, ASMR), and TPC values for myocardial disease attributable to high and low temperatures across countries in 1990 vs. 2021.** | | | | | | | | |
| --- | --- | --- | --- | --- | --- | --- | --- | --- |
| Location | Myocardial Disease attributable to  High temperature | | Myocardial Disease attributable to  Low temperature | | Myocardial Disease attributable to  High temperature | | Myocardial Disease attributable to  Low temperature | |
|  | ASDR (95% UI) | TPC (95% UI) | ASDR (95% UI) | TPC (95% UI) | ASMR(95%UI) | TPC(95% UI) | ASMR (95%UI) | TPC(95% UI) |
| People's Republic of China | 1.26(0.01,2.59) | -0.14(-0.49,0.25) | 2.93(1.49,3.96) | -0.44(-0.69,-0.2) | 0.05(0.0,0.1) | 0.23 (-0.42,0.79) | 0.11(0.05,0.15) | -0.17 (-0.59, 0.2) |
| Democratic People's Republic of Korea | 0.36(-0.35,1.3) | 0.49 (-3.03,5.73) | 5.34(3.64,7.96) | -0.19(-0.45,0.18) | 0.01 (-0.01,0.05) | 0.66(-3.42, 6.39) | 0.2(0.13,0.33) | -0.1 (-0.41, 0.35) |
| Taiwan (Province of China) | 1.72(-0.64,3.85) | 0.16(-0.93,1.25) | 1.71(0.65,2.66) | -0.44(-0.63,-0.34) | 0.06(-0.02,0.13) | -0.06(-0.94,0.81) | 0.06(0.02,0.09) | -0.54 (-0.7, -0.47) |
| Kingdom of Cambodia | -3.11 (-4.94,-1.44) | -0.16(-0.75,3.96) | 0.67(-0.24, 1.82) | -0.14 (-1.2, 3.04) | -0.15(-0.22,-0.07) | -0.13 (-0.7,4.13) | 0.03(-0.01,0.09) | -0.11 (-1.21, 3.09) |
| Republic of Maldives | -1.78(-2.61,-0.6) | -0.31(-0.81,0.45) | 0.06(0.01,0.26) | -0.59(-0.92,2.14) | -0.1(-0.14,-0.03) | -0.22 (-0.77,0.7) | 0.0(0.0,0.01) | -0.53 (-0.9, 2.53) |
| Republic of the Union of Myanmar | -0.01 (-2.22,2.42) | -0.99(-19.85,9.05) | 1.45(0.39,2.73) | -0.51(-0.69,-0.2) | -0.0(-0.1,0.11) | -0.99 (-19.67,8.66) | 0.06(0.02,0.12) | -0.51 (-0.71, -0.19) |
| Malaysia | -1.56(-2.01,-0.67) | -0.28(-0.7,0.11) | 0.32 (0.16,0.59) | -0.6(-0.75,-0.24) | -0.07(-0.09,-0.03) | -0.27 (-0.69,0.22) | 0.01 (0.01,0.03) | -0.59 (-0.75, -0.21) |
| Republic of the Philippines | -2.42 (-3.09,-1.56) | -0.16(-0.47,0.2) | 0.44(0.15,0.78) | -0.37(-0.57,0.18) | -0.1 (-0.13,-0.06) | -0.33 (-0.57,-0.03) | 0.02 (0.01,0.04) | -0.48 (-0.64, -0.04) |
| Kingdom of Thailand | -0.69(-1.09,-0.2) | -0.1(-0.72,0.76) | 0.4(0.05,0.86) | 1.57(0.1,3.96) | -0.02 (-0.04,-0.01) | -0.21 (-0.74,0.5) | 0.01 (0.0,0.03) | 1.26 (0.03, 3.45) |
| Democratic Socialist Republic of Sri Lanka | -1.4(-2.35,-0.9) | -0.77(-0.87,-0.41) | 0.48(0.09, 1.05) | -0.82 (-0.92,-0.57) | -0.05(-0.1,-0.03) | -0.78(-0.88,-0.39) | 0.02 (0.0,0.04) | -0.82 (-0.93, -0.53) |
| Lao People's Democratic Republic | 0.92 (-0.57,2.54) | 6.34(-15.36,24.94) | 2.36(0.83,4.27) | -0.17 (-0.61,1.17) | 0.04(-0.02,0.11) | 6.18(-17.26,24.66) | 0.11 (0.04,0.19) | -0.17 (-0.58, 1.13) |
| Democratic Republic of Timor-Leste | -0.0(-0.18,0.12) | -0.99(-3.23,1.61) | 0.6(-0.56,1.27) | -0.43(-1.89,0.03) | -0.0(-0.01,0.01) | -0.99 (-3.08,1.62) | 0.03(-0.03,0.06) | -0.44 (-1.88, 0.05) |
| Republic of Indonesia | -1.05(-1.41,-0.59) | 0.2(-0.32,1.0) | 0.38(-0.51,0.77) | -0.51 (-1.72,0.2) | -0.05(-0.07,-0.03) | 0.36(-0.19,1.25) | 0.02 (-0.03,0.04) | -0.47 (-1.84, 0.21) |
| Socialist Republic of Viet Nam | 1.7(-0.54,3.94) | 3.78(-26.88, 19.27) | 2.11(0.86,3.59) | -0.08(-0.4,0.44) | 0.09(-0.03,0.21) | 3.76(-24.31,18.83) | 0.11(0.04,0.19) | -0.08 (-0.43, 0.45) |
| Republic of Kiribati | -1.86(-3.94,-0.96) | -0.37(-0.6,0.01) | 0.65(0.12,1.09) | 7.82 (1.74, 14.49) | -0.06 (-0.13,-0.03) | -0.37 (-0.59,-0.01) | 0.02(0.0,0.04) | 7.82 (1.76, 15.65) |
| Republic of Fiji | 0.11 (-0.2, 0.37) | -20.92 (-49.7,57.3) | 0.69(-1.99,1.56) | -0.61 (-1.76,-0.1) | 0.0(-0.01,0.01) | -19.97(-45.83,55.9) | 0.02 (-0.06,0.04) | -0.63 (-1.7, -0.2) |
| Republic of the Marshall Islands | -6.03 (-8.89,-3.7) | 0.28(-0.06,0.8) | 0.01(0.01,0.02) | -0.82(-0.87,-0.74) | -0.19(-0.27,-0.12) | 0.2 (-0.13,0.75) | 0.0 (0.0,0.0) | -0.83 (-0.88, -0.75) |
| Independent State of Samoa | -0.21(-0.29,-0.14) | -0.38(-0.84,0.28) | 1.54(1.08,2.18) | -0.12 (-0.39, 1.52) | -0.01(-0.01,-0.0) | -0.39(-0.85,0.28) | 0.05(0.04,0.07) | -0.14 (-0.4, 1.44) |
| Federated States of Micronesia | -6.06(-8.5, - 4.08) | 0.06(-0.22,0.58) | 0.02(0.01,0.02) | -0.75(-0.83,-0.61) | -0.2 (-0.28,-0.13) | 0.0(-0.27,0.51) | 0.0 (0.0,0.0) | -0.77 (-0.83, -0.63) |
| Solomon Islands | -1.42 (-3.2, - 0.71) | 0.35(-0.44,2.28) | 0.22 (0.04,0.43) | -0.61(-0.85,-0.01) | -0.05(-0.12,-0.03) | 0.34(-0.43,2.37) | 0.01(0.0,0.02) | -0.62 (-0.85, -0.03) |
| Independent State of Papua New Guinea | -0.63 (-1.33,-0.3) | 0.55(-0.27,3.3) | 3.07(-0.86,5.67) | -0.26(-0.89,0.26) | -0.02 (-0.04,-0.01) | 0.55(-0.26,3.25) | 0.08(-0.02,0.16) | -0.26 (-0.89, 0.24) |
| Kingdom of Tonga | -0.77(-1.1, - 0.53) | -0.16 (-0.76,0.53) | 0.17(0.06,0.26) | -0.42 (-0.64,4.41) | -0.03(-0.04,-0.02) | -0.18(-0.77,0.49) | 0.01(0.0,0.01) | -0.44 (-0.66, 4.13) |
| Republic of Vanuatu | -0.32(-0.87,-0.15) | -0.19(-0.78,0.65) | 1.0(0.29,1.78) | -0.29(-0.59,0.27) | -0.01 (-0.03,-0.0) | -0.22 (-0.78,0.58) | 0.03(0.01,0.05) | -0.32 (-0.6, 0.21) |
| Republic of Armenia | 0.48(-0.59,1.8) | 0.9(-6.21,4.27) | 8.77(5.77,11.46) | -0.41(-0.59,-0.15) | 0.02 (-0.02,0.07) | 0.8 (-6.06,4.16) | 0.32 (0.22,0.42) | -0.44 (-0.61, -0.23) |
| Republic of Azerbaijan | 7.76(0.35,16.94) | 1.28(-0.11,3.47) | 33.43(17.75,52.49) | -0.02 (-0.49,0.67) | 0.26(0.01,0.57) | 1.26(-0.06,3.42) | 1.11 (0.64, 1.76) | -0.04 (-0.45, 0.68) |
| Georgia | 0.85(-0.76,2.82) | 4.97(-20.69,22.65) | 14.16(7.7,19.14) | 0.13 (-0.26,0.61) | 0.03(-0.03,0.1) | 4.79(-20.36,21.44) | 0.5(0.27,0.67) | 0.09 (-0.27, 0.52) |
| Republic of Kazakhstan | 12.85(-5.84,35.18) | 45.84(-91.26,201.71) | 75.5(57.63,95.29) | 17.59 (12.86, 24.18) | 0.45(-0.2, 1.25) | 53.34 (-105.24,234.23) | 2.65(2.07,3.3) | 20.55 (15.16, 28.55) |
| Mongolia | 0.21(-0.83,1.33) | 1.69(-3.54,9.95) | 20.16(7.4,35.54) | 0.06(-0.47,1.16) | 0.01(-0.03,0.04) | 1.68(-3.64,9.27) | 0.7(0.26,1.22) | 0.06 (-0.47, 1.11) |
| Kyrgyz Republic | 0.88(-1.58,3.88) | 1.33(-5.75,6.28) | 23.33(16.35,30.11) | 0.92 (0.44,1.54) | 0.03(-0.05,0.11) | 1.55(-6.26,6.88) | 0.69 (0.48,0.9) | 1.1 (0.57, 1.81) |
| Republic of Tajikistan | 1.17(0.24,2.18) | 0.31(-0.21,1.21) | 2.9 (1.92,4.11) | -0.29(-0.57,0.09) | 0.04 (0.01,0.07) | 0.21(-0.22,0.96) | 0.09 (0.06,0.13) | -0.34 (-0.58, -0.02) |
| Turkmenistan | 41.22(15.53,70.44) | 0.18(-0.11,0.66) | 43.79(30.83,61.39) | -0.24(-0.45,0.02) | 1.22 (0.45,2.1) | 0.09(-0.17,0.54) | 1.3(0.91, 1.86) | -0.29 (-0.49, -0.05) |
| Republic of Uzbekistan | 2.79(0.57,5.31) | 1.53(0.83,2.91) | 5.8(4.08,7.49) | 0.54(0.14,1.08) | 0.09(0.02,0.17) | 1.88(1.1,3.34) | 0.19(0.13,0.25) | 0.75 (0.3, 1.37) |
| Republic of Albania | 1.43 (0.0, 3.43) | 2.1(-4.65,9.39) | 12.49(5.84,18.38) | -0.31(-0.55,0.03) | 0.07(0.0,0.17) | 1.86(-4.23,8.63) | 0.65(0.33,0.95) | -0.37 (-0.59, -0.05) |
| Bosnia and Herzegovina | 0.89(-1.16,3.72) | 6.14(-16.43,20.51) | 29.49(16.61,42.14) | 0.04(-0.3,0.52) | 0.05(-0.06,0.19) | 6.04(-16.33,20.61) | 1.52(0.9, 2.18) | 0.03 (-0.3, 0.46) |
| Republic of Bulgaria | 0.5 (-0.66, 2.01) | 5.95(-25.0,25.44) | 11.63(7.63,15.49) | 0.78(0.35,1.34) | 0.02(-0.03,0.09) | 5.91(-25.42,24.22) | 0.53(0.35,0.68) | 0.76 (0.39, 1.23) |
| Republic of Croatia | 0.87(-0.95,3.16) | 5.84(-18.49, 19.06) | 16.23(11.41,20.75) | 0.26(-0.02,0.6) | 0.04(-0.05,0.15) | 4.79(-15.86, 15.58) | 0.8(0.58,1.0) | 0.06 (-0.13, 0.32) |
| Czech Republic | 0.0(-0.17,0.11) | -1.15(-4.07,6.65) | 7.62(5.87,9.44) | -0.06(-0.29,0.2) | 0.0(-0.01,0.0) | -1.19(-4.15,6.78) | 0.31(0.25,0.38) | -0.03 (-0.24, 0.22) |
| Hungary | 1.62(-1.66,6.13) | 1.58(-10.12, 7.35) | 29.59(22.67,35.9) | -0.27(-0.41,-0.11) | 0.06(-0.06,0.23) | 1.01 (-8.12, 5.47) | 1.1(0.85,1.32) | -0.44 (-0.53, -0.32) |
| North Macedonia | 2.02 (-2.02,7.27) | 2.66(-19.51,13.6) | 39.59(22.62,57.5) | 0.01(-0.3,0.39) | 0.11(-0.11,0.39) | 2.96 (-20.56,15.11) | 2.16(1.19,3.1) | 0.09 (-0.28, 0.55) |
| Montenegro | 3.32 (-0.41,8.45) | 4.93(-12.32, 24.18) | 41.79(25.0,58.63) | 0.02(-0.25,0.47) | 0.18(-0.02,0.47) | 5.55(-12.68,28.67) | 2.24(1.32,3.13) | 0.13 (-0.19, 0.8) |
| Republic of Poland | 0.06(-0.53,0.46) | -1.93 (-22.17,26.86) | 24.9 (21.14,28.51) | -0.25(-0.35,-0.14) | 0.0(-0.03,0.02) | -1.97(-20.89,31.94) | 1.21(1.05,1.36) | -0.25 (-0.32, -0.16) |
| Romania | 2.42 (-2.96,9.35) | 1.81(-8.09,10.74) | 56.41(41.83,68.59) | -0.02(-0.21,0.25) | 0.11(-0.13,0.41) | 1.18(-6.56,7.9) | 2.45(1.83,2.95) | -0.24 (-0.4, -0.03) |
| Slovak Republic | 0.17(-0.44,0.83) | 4.19(-10.04, 14.74) | 11.37(7.87,14.88) | 0.16(-0.25,0.65) | 0.01 (-0.02,0.03) | 4.12 (-9.66,14.22) | 0.45(0.33,0.58) | 0.15 (-0.21, 0.59) |
| Republic of Serbia | 1.92 (-1.59,6.72) | 1.84(-14.47, 10.06) | 31.25(18.89,40.94) | -0.26(-0.45,-0.0) | 0.1(-0.08,0.35) | 1.52(-12.46,9.13) | 1.61(0.96,2.22) | -0.35 (-0.55, -0.08) |
| Republic of Slovenia | 0.31 (-0.5, 1.38) | 0.21(-6.59,6.93) | 18.19(13.73,22.65) | -0.78(-0.82,-0.73) | 0.02 (-0.03,0.08) | 0.15(-6.28,6.87) | 1.08(0.84,1.32) | -0.79 (-0.83, -0.76) |
| Republic of Belarus | 0.24(-1.75, 1.77) | -13.25(-339.9, 1378.51) | 30.46 (21.49,38.81) | -0.05(-0.36,0.32) | 0.01 (-0.04,0.04) | -11.27(-277.42,1140.23) | 0.74(0.52,0.95) | -0.21 (-0.46, 0.09) |
| Republic of Latvia | 0.7(-2.39,3.28) | N/A | 69.64(48.48,89.0) | 0.61(0.33,1.0) | 0.02 (-0.07,0.09) | N/A | 2.01(1.39,2.55) | 0.79 (0.47, 1.23) |
| Republic of Estonia | 0.27(-0.77,1.31) | N/A | 13.35(6.07,19.41) | -0.45(-0.7, - 0.19) | 0.01(-0.03,0.05) | N/A | 0.46 (0.21,0.66) | -0.3 (-0.63, 0.03) |
| Republic of Lithuania | 0.13 (-1.09,0.93) | -3.79(-525.03, 6925.37) | 29.16(23.02,35.1) | 0.5(0.2,0.91) | 0.0(-0.03,0.03) | -4.04(-565.34,7540.65) | 0.87(0.69,1.04) | 0.63 (0.33, 1.05) |
| Republic of Moldova | 0.54(-1.33,2.72) | 5.5(-9.1, 16.75) | 21.59(17.18,25.98) | 1.07(0.71,1.48) | 0.02 (-0.04,0.08) | 6.1 (-9.83,18.0) | 0.62 (0.5,0.75) | 1.25 (0.9, 1.67) |
| Russian Federation | 2.84(-4.86,11.63) | 17.66(-57.61,66.11) | 51.08(35.44,68.77) | 1.51(1.11,2.07) | 0.08(-0.13,0.31) | 13.04(-26.87,103.06) | 1.38(0.98,1.85) | 1.13 (0.73, 1.71) |
| Brunei Darussalam | -4.73(-5.77,-3.75) | -0.21 (-0.39,0.03) | 0.47(0.27,0.77) | -0.61(-0.72,-0.43) | -0.18(-0.22,-0.14) | -0.2 (-0.42,0.03) | 0.02 (0.01,0.03) | -0.61 (-0.72, -0.42) |
| Ukraine | 0.95(-4.49,6.11) | 37.22 (-15.73,23.63) | 68.27(48.26,91.62) | 0.21 (-0.17,0.73) | 0.03(-0.13,0.18) | 36.78(-15.89,24.37) | 2.05(1.51,2.7) | 0.24 (-0.12, 0.69) |
| Republic of Korea | 0.3 (-0.28,1.02) | -0.38(-2.03,0.87) | 3.19(2.15,4.06) | -0.52 (-0.68,-0.29) | 0.01 (-0.01,0.04) | -0.32 (-2.1,1.02) | 0.13(0.09,0.16) | -0.47 (-0.62, -0.2) |
| Republic of Singapore | -0.61(-1.59,-0.39) | -0.9(-0.94,-0.72) | 0.3 (0.04,0.4) | -0.64(-0.9,-0.12) | -0.02 (-0.06,-0.01) | -0.88(-0.94,-0.69) | 0.01(0.0,0.01) | -0.58 (-0.89, 0.01) |
| New Zealand | 0.0(-0.03,0.03) | -5.15(-1.42,0.64) | 7.73 (3.11,11.03) | -0.53 (-0.65,-0.46) | 0.0 (-0.0,0.0) | -7.3 (-1.66,0.64) | 0.27 (0.1,0.39) | -0.47 (-0.61, -0.4) |
| Japan | 0.55(0.03,1.13) | -0.75(-0.83,-0.71) | 3.68(2.65,4.22) | -0.68(-0.7,-0.65) | 0.02 (0.0,0.04) | -0.77 (-0.84,-0.73) | 0.15(0.11,0.17) | -0.7 (-0.72, -0.67) |
| Republic of Austria | 0.11(-0.28,0.59) | -0.38(-2.03,0.85) | 11.36(9.46,12.83) | -0.79(-0.81,-0.78) | 0.01 (-0.02,0.03) | -0.46 (-1.9,0.58) | 0.64(0.53,0.74) | -0.82 (-0.84, -0.8) |
| Australia | 0.26(-0.16,0.77) | -0.71(-1.01,-0.39) | 3.52(1.05,4.79) | -0.61(-0.75,-0.22) | 0.01 (-0.01,0.03) | -0.71 (-1.01,-0.39) | 0.14(0.04,0.19) | -0.61 (-0.75, -0.22) |
| Kingdom of Belgium | 0.01 (-0.05,0.07) | -0.86(-1.25,-0.7) | 5.33(3.92,6.3) | -0.65(-0.69,-0.6) | 0.0(-0.0,0.0) | -0.85 (-1.26,-0.67) | 0.31(0.22,0.37) | -0.62 (-0.66, -0.57) |
| Principality of Andorra | 0.0(-0.01,0.02) | N/A | 6.32 (3.22,9.87) | -0.46(-0.68,-0.18) | 0.0(-0.0,0.0) | N/A | 0.35(0.18,0.55) | -0.44 (-0.66, -0.11) |
| Republic of Cyprus | 3.08(0.53,5.98) | -0.32(-0.59,0.37) | 2.57(0.09,4.48) | -0.68(-0.96,-0.47) | 0.19(0.03,0.37) | -0.32 (-0.6,0.44) | 0.16(0.01,0.28) | -0.68 (-0.96, -0.45) |
| Kingdom of Denmark | 0.0(-0.01,0.01) | -1.03(-2.78,2.9) | 3.08(2.62,3.52) | -0.53(-0.6, - 0.43) | 0.0(-0.0,0.0) | -1.04(-3.08,3.55) | 0.15(0.12,0.17) | -0.44 (-0.53, -0.33) |
| Republic of Finland | 0.07(-0.26,0.46) | -23.9 (-150.82, 832.08) | 6.0 (2.3,8.48) | -0.49(-0.64,-0.35) | 0.0(-0.01,0.02) | -26.93(-166.13,934.76) | 0.23(0.09,0.32) | -0.43 (-0.6, -0.28) |
| French Republic | 0.04(-0.09,0.21) | -0.76(-1.19,-0.51) | 5.16(3.74,6.22) | -0.6(-0.65,-0.56) | 0.0(-0.0,0.01) | -0.74(-1.2,-0.48) | 0.28(0.2,0.34) | -0.58 (-0.63, -0.53) |
| Federal Republic of Germany | 0.03(-0.14,0.18) | -0.23 (-1.76,0.62) | 9.61 (8.2, 10.82) | -0.54(-0.61,-0.47) | 0.0(-0.01,0.01) | -0.19 (-1.81,0.73) | 0.45(0.37,0.5) | -0.52 (-0.59, -0.45) |
| Hellenic Republic | 1.55(0.38,2.93) | 1.28(0.4, 5.08) | 4.45(2.22,5.93) | -0.25(-0.39,0.18) | 0.06(0.01,0.11) | 0.66 (0.03,3.45) | 0.17(0.08,0.23) | -0.45 (-0.56, -0.13) |
| Republic of Iceland | -0.0(-0.0,0.0) | N/A | 2.05(1.01,2.96) | -0.58(-0.68,-0.48) | -0.0(-0.0,0.0) | N/A | 0.1 (0.05,0.15) | -0.55 (-0.66, -0.45) |
| Ireland | 0.0(-0.03,0.03) | N/A | 7.1(5.31,8.55) | -0.69(-0.73,-0.66) | 0.0(-0.0,0.0) | N/A | 0.37(0.27,0.46) | -0.7 (-0.74, -0.66) |
| State of Israel | 0.78(-0.26,1.91) | 0.09(-2.5,4.14) | 0.77(0.02,1.25) | -0.78(-0.98,-0.47) | 0.04(-0.01,0.1) | 0.24(-2.69,4.83) | 0.04 (0.0,0.06) | -0.75 (-0.98, -0.39) |
| Republic of Italy | 0.39(-0.06,0.98) | -0.44 (-1.94,1.77) | 4.16(2.17,5.29) | -0.87(-0.91,-0.85) | 0.02 (-0.0,0.05) | -0.59 (-1.61,1.08) | 0.23 (0.12,0.29) | -0.9 (-0.93, -0.89) |
| Grand Duchy of Luxembourg | 0.0(-0.04,0.04) | -0.91 (-1.24,-0.63) | 4.77(3.93,5.57) | -0.73(-0.77,-0.69) | 0.0(-0.0,0.0) | -0.9 (-1.29,-0.57) | 0.28(0.23,0.33) | -0.69 (-0.73, -0.64) |
| Republic of Malta | 1.1 (-0.29, 2.97) | -0.3 (-2.4,4.36) | 1.87(-0.88,3.71) | -0.78(-0.99,-0.58) | 0.05(-0.01,0.14) | -0.39 (-2.2,3.61) | 0.09 (-0.04,0.17) | -0.81 (-0.99, -0.63) |
| Kingdom of the Netherlands | 0.0(-0.04,0.05) | -0.88(-1.15,-0.6) | 4.76(3.42,5.59) | -0.71(-0.75,-0.67) | 0.0(-0.0,0.0) | -0.87 (-1.15,-0.59) | 0.26(0.18,0.31) | -0.7 (-0.74, -0.66) |
| Kingdom of Norway | 0.0(-0.01,0.01) | 26.04(-4.01,14.59) | 2.99(1.96,3.81) | -0.37(-0.44,-0.23) | 0.0(-0.0,0.0) | 42.12 (-6.87, 19.43) | 0.12(0.08,0.16) | -0.08 (-0.19, 0.15) |
| Portuguese Republic | 0.05(0.0,0.11) | -0.84(-0.89,-0.78) | 3.16(1.01,4.59) | -0.43(-0.6, - 0.16) | 0.0(0.0,0.01) | -0.84 (-0.89,-0.79) | 0.14(0.05,0.2) | -0.45 (-0.62, -0.19) |
| Kingdom of Spain | 0.7(0.06,1.52) | -0.47(-0.63,-0.12) | 6.42(3.76,8.22) | -0.54(-0.61,-0.46) | 0.03(0.0,0.07) | -0.53 (-0.68,-0.24) | 0.31(0.18,0.38) | -0.6 (-0.66, -0.52) |
| Republic of Chile | 0.0 (-0.01,0.02) | -0.85(-2.13,6.19) | 4.82 (2.17,6.52) | -0.61(-0.7,-0.54) | 0.0(-0.0,0.0) | -0.84 (-2.18,6.53) | 0.19(0.08,0.26) | -0.59 (-0.69, -0.52) |
| Kingdom of Sweden | 0.0(-0.03,0.04) | -4.2(-12.98,22.14) | 5.03(3.75,6.18) | -0.48(-0.56,-0.4) | 0.0(-0.0,0.0) | -4.89 (-15.49, 26.88) | 0.19(0.14,0.23) | -0.38 (-0.46, -0.27) |
| Swiss Confederation | 0.0(-0.03,0.03) | -1.36(-1.83,0.6) | 3.33 (2.66,4.0) | -0.66 (-0.72,-0.59) | 0.0(-0.0,0.0) | -1.44 (-1.97,0.97) | 0.17(0.14,0.21) | -0.59 (-0.66, -0.5) |
| Canada | 0.05(-0.2, 0.28) | 2.35(-2.48,2.49) | 4.19(3.55,4.72) | -0.4(-0.48,-0.31) | 0.0(-0.01,0.01) | 2.54(-2.59,2.65) | 0.16(0.13,0.18) | -0.37 (-0.44, -0.27) |
| United Kingdom of Great Britain and Northern Ireland | 0.0(-0.03,0.04) | -0.53 (-1.35,0.46) | 6.74(4.87,8.0) | -0.2 (-0.3,-0.08) | 0.0(-0.0,0.0) | -0.36 (-1.46,0.77) | 0.26(0.19,0.31) | -0.02 (-0.15, 0.11) |
| Argentine Republic | 2.2(-0.18,4.65) | -0.33(-0.5,-0.11) | 11.97(7.94,14.62) | -0.51(-0.61,-0.33) | 0.11(-0.01,0.22) | -0.27 (-0.46,-0.01) | 0.57(0.38,0.71) | -0.46 (-0.58, -0.27) |
| Eastern Republic of Uruguay | 0.46 (0.0, 1.16) | -0.76(-0.9,-0.68) | 4.96 (0.64,7.36) | -0.63(-0.79,-0.45) | 0.02(0.0,0.05) | -0.75(-0.9,-0.67) | 0.22 (0.03,0.32) | -0.62 (-0.78, -0.44) |
| United States of America | 2.12 (-0.26,4.91) | -0.32(-0.91,0.03) | 9.3(6.87, 11.21) | -0.51(-0.55,-0.45) | 0.08(-0.01,0.17) | -0.31(-0.86,0.02) | 0.35(0.26,0.42) | -0.48 (-0.53, -0.42) |
| Antigua and Barbuda | -1.15(-1.4, - 0.96) | 0.49 (0.31,0.73) | 1.0(0.57, 1.28) | -0.3(-0.39,-0.19) | -0.05(-0.06,-0.04) | 0.72(0.5,0.98) | 0.04(0.02,0.05) | -0.19 (-0.29, -0.07) |
| Barbados | -1.42 (-1.85,-1.08) | 0.09 (-0.21,0.43) | 0.41(0.23,0.59) | -0.66(-0.76,-0.55) | -0.06(-0.07,-0.04) | 0.2 (-0.1,0.54) | 0.02(0.01,0.02) | -0.62 (-0.72, -0.52) |
| Belize | -0.85 (-2.93,1.59) | -0.01(-10.22,3.27) | 1.34(0.64,2.21) | -0.44 (-0.6, - 0.2) | -0.03(-0.1,0.06) | 0.15(-11.87,3.97) | 0.05(0.02,0.08) | -0.35 (-0.54, -0.05) |
| Commonwealth of the Bahamas | 2.12 (-2.74,6.11) | -14.13 (-24.32, 29.41) | 4.86(3.08, 7.0) | 0.28(-0.12,1.1) | 0.07(-0.09,0.21) | -15.03(-25.38, 31.38) | 0.16(0.1,0.24) | 0.36 (-0.06, 1.21) |
| Republic of Cuba | 0.18(-1.28,1.41) | -2.49(-29.52, 34.78) | 2.5(1.57,3.47) | 0.87(0.56,1.26) | 0.01(-0.05,0.05) | -2.84 (-36.69,43.72) | 0.09(0.06,0.13) | 1.31 (0.94, 1.8) |
| Commonwealth of Dominica | -2.81(-3.54,-2.17) | 0.63 (0.1, 1.3) | 3.16(1.9,4.36) | -0.37(-0.58,-0.12) | -0.1(-0.13,-0.08) | 0.57(0.05,1.2) | 0.12 (0.07,0.16) | -0.4 (-0.59, -0.17) |
| Dominican Republic | -0.04 (-0.4, 0.22) | -0.88(-1.81,0.47) | 1.09 (-0.19, 1.72) | -0.42 (-0.98,-0.05) | -0.0(-0.01,0.01) | -0.87(-1.98,0.72) | 0.03(-0.01,0.05) | -0.34 (-0.98, 0.13) |
| Republic of Guyana | -3.62(-8.75,-1.46) | 0.4(-0.64,2.58) | 1.67(0.34,3.08) | -0.35(-0.71,0.94) | -0.12 (-0.3,-0.05) | 0.51(-0.62,2.83) | 0.06(0.01,0.1) | -0.29 (-0.69, 1.08) |
| Grenada | -2.84(-3.48,-2.32) | 0.91 (0.59, 1.32) | 0.6(0.33,0.81) | -0.6(-0.68,-0.5) | -0.11(-0.13,-0.09) | 1.23 (0.87,1.66) | 0.02 (0.01,0.03) | -0.53 (-0.63, -0.43) |
| Republic of Haiti | -3.45(-5.87,-1.42) | -0.02 (-0.47,1.54) | 1.93(0.8, 3.61) | -0.31(-0.57,0.37) | -0.11(-0.18,-0.04) | -0.03 (-0.48, 1.48) | 0.06(0.02,0.11) | -0.32 (-0.57, 0.34) |
| Jamaica | -0.2 (-1.19,0.6) | -0.31(-23.46, 2.16) | 1.01 (0.74, 1.39) | -0.45(-0.61,-0.27) | -0.01(-0.04,0.02) | -0.25 (-25.13,2.38) | 0.04(0.03,0.05) | -0.41 (-0.57, -0.23) |
| Saint Lucia | -2.97(-6.51,-2.23) | -0.0(-0.65,0.6) | 0.88(0.14, 1.22) | -0.56(-0.72,0.78) | -0.12(-0.25,-0.09) | -0.0(-0.65,0.57) | 0.03(0.01,0.05) | -0.56 (-0.72, 0.77) |
| Saint Vincent and the Grenadines | -1.23 (-1.5, - 1.0) | 0.81(-0.46,1.72) | 0.26(0.14,0.36) | -0.49(-0.62,2.13) | -0.05(-0.05,-0.04) | 0.98(-0.41,1.93) | 0.01 (0.01,0.01) | -0.44 (-0.58, 2.41) |
| Republic of Suriname | -1.17(-3.58,-0.74) | -0.29(-0.77,0.62) | 0.53(0.08,0.8) | -0.38(-0.67, 1.7) | -0.04(-0.12,-0.02) | -0.26(-0.77,0.71) | 0.02 (0.0, 0.03) | -0.35 (-0.66, 1.93) |
| Republic of Trinidad and Tobago | -0.94(-3.21,-0.61) | 0.29 (-0.12,2.78) | 0.53(0.06,0.82) | -0.51 (-0.9, - 0.34) | -0.03(-0.1,-0.02) | 0.22 (-0.17,2.67) | 0.02(0.0,0.03) | -0.53 (-0.91, -0.37) |
| Plurinational State of Bolivia | 0.38(-0.04,0.82) | 1.09(-7.61,10.15) | 4.16(2.64,5.62) | -0.47(-0.64,-0.16) | 0.01(-0.0,0.03) | 1.37(-8.24,11.75) | 0.13 (0.08,0.18) | -0.4 (-0.57, -0.09) |
| Republic of Ecuador | -0.02(-0.08,0.04) | -0.82 (-1.55,-0.01) | 1.61 (0.8, 2.23) | -0.43(-0.63,-0.21) | -0.0 (-0.0,0.0) | -0.82 (-1.56,-0.01) | 0.05(0.03,0.08) | -0.42 (-0.63, -0.2) |
| Republic of Peru | 0.01(-0.02,0.04) | -6.43 (-4.09, 2.04) | 1.25(0.28,1.89) | -0.66 (-0.79,-0.44) | 0.0(-0.0,0.0) | -7.26(-4.13,2.42) | 0.04(0.01,0.06) | -0.64 (-0.78, -0.38) |
| Republic of Colombia | -0.44(-0.55,-0.34) | -0.55(-0.74,-0.05) | 1.22 (-0.84,2.16) | -0.58(-1.29,-0.1) | -0.02 (-0.02,-0.01) | -0.54 (-0.74,-0.05) | 0.04(-0.03,0.08) | -0.57 (-1.3, -0.06) |
| Republic of Costa Rica | -0.12 (-0.18,-0.1) | -0.37(-0.62,-0.08) | 0.98(-1.53,2.09) | -0.38(-1.15,0.06) | -0.0(-0.01,-0.0) | -0.39 (-0.64,-0.12) | 0.04(-0.06,0.08) | -0.4 (-1.15, 0.01) |
| Republic of El Salvador | -0.78(-1.07,-0.43) | -0.6(-0.78,-0.22) | 0.09(0.01,0.17) | -0.67(-0.83,-0.34) | -0.03(-0.03,-0.01) | -0.55(-0.75,-0.11) | 0.0(0.0,0.01) | -0.63 (-0.82, -0.25) |
| Republic of Guatemala | -0.15(-0.26,0.02) | -0.54(-1.07,1.25) | 0.77(-1.27,1.58) | -0.64(-1.86,0.06) | -0.0(-0.01,0.0) | -0.62 (-1.06,0.79) | 0.02 (-0.04,0.04) | -0.7 (-1.72, -0.14) |
| Republic of Honduras | 0.11(-0.4,0.66) | -1.23(-2.56,0.02) | 0.7(-1.62,1.61) | -0.57(-1.96,0.06) | 0.0(-0.02,0.02) | -1.26 (-2.84,0.16) | 0.03(-0.07,0.06) | -0.51 (-2.02, 0.25) |
| United Mexican States | 0.54(-0.08,1.07) | 4.03 (-20.32,34.68) | 1.64(0.51,2.28) | 0.18(-0.39,0.53) | 0.02 (-0.0,0.03) | 3.01(-11.66,25.47) | 0.05(0.01,0.06) | 0.11 (-0.43, 0.42) |
| Republic of Nicaragua | -0.85(-1.05,-0.67) | -0.56(-0.77,-0.14) | 0.24(-0.03,0.42) | -0.52 (-1.09,-0.25) | -0.03(-0.04,-0.02) | -0.46(-0.72,0.05) | 0.01(-0.0,0.02) | -0.42 (-1.1, -0.06) |
| Republic of Panama | -0.75(-1.9, - 0.49) | -0.5(-0.71,0.28) | 0.59(0.14,0.9) | -0.07(-0.58,0.54) | -0.03 (-0.07,-0.02) | -0.48(-0.7,0.32) | 0.02 (0.01,0.03) | -0.04 (-0.57, 0.58) |
| Bolivarian Republic of Venezuela | -1.16(-1.56,-0.87) | -0.44(-0.62,-0.2) | 0.9(0.15,1.58) | -0.62 (-0.86,-0.45) | -0.04(-0.06,-0.03) | -0.47 (-0.64,-0.25) | 0.03 (0.01,0.06) | -0.64 (-0.87, -0.48) |
| Federative Republic of Brazil | -0.45(-1.22,0.55) | -0.22 (-2.22,1.51) | 4.8(1.69,7.46) | -0.56 (-0.7, - 0.46) | -0.01(-0.04,0.02) | -0.12(-2.52,0.92) | 0.18(0.06,0.29) | -0.59 (-0.73, -0.49) |
| Republic of Paraguay | 2.24(-0.61,5.12) | -0.4(-0.64, - 0.08) | 3.8(1.33,6.43) | -0.53(-0.69,-0.3) | 0.09(-0.02,0.22) | -0.39 (-0.64,-0.03) | 0.16(0.06,0.27) | -0.52 (-0.69, -0.27) |
| People's Democratic Republic of Algeria | 2.75(0.86,5.53) | 0.07 (-0.32, 1.02) | 2.9(1.02,5.57) | -0.54(-0.8, - 0.27) | 0.11(0.03,0.24) | 0.33(-0.1,1.37) | 0.12 (0.04,0.24) | -0.43 (-0.73, -0.14) |
| Kingdom of Bahrain | 11.86(2.2,22.14) | -0.34(-0.5,-0.12) | 5.98(1.9, 11.07) | -0.6(-0.7,-0.47) | 0.56(0.11,1.02) | -0.27 (-0.43,-0.03) | 0.28(0.09,0.51) | -0.56 (-0.66, -0.4) |
| Arab Republic of Egypt | 6.18(0.39,13.79) | 0.13 (-1.15,2.03) | 3.19(0.85,6.75) | -0.67 (-0.88,-0.35) | 0.2(0.01,0.49) | 0.57(-1.22,3.22) | 0.1 (0.03,0.24) | -0.54 (-0.83, -0.18) |
| Islamic Republic of Iran | 2.15(0.27,4.07) | -0.33(-0.53,0.07) | 3.01(2.29,3.98) | -0.59 (-0.71,-0.39) | 0.08(0.01,0.15) | -0.25 (-0.48,0.16) | 0.12 (0.09,0.16) | -0.47 (-0.63, -0.23) |
| Republic of Iraq | 27.26(7.59,45.31) | -0.2 (-0.42,0.18) | 15.36(5.27,26.52) | -0.51(-0.65,-0.29) | 0.96 (0.26, 1.77) | -0.04 (-0.36,0.43) | 0.54(0.18,1.01) | -0.41 (-0.61, -0.17) |
| State of Kuwait | 5.29(0.88,8.95) | -0.64(-0.7,-0.56) | 2.48(0.61,4.76) | -0.77 (-0.8, - 0.72) | 0.19(0.03,0.33) | -0.56 (-0.64,-0.46) | 0.09(0.02,0.18) | -0.72 (-0.77, -0.66) |
| Hashemite Kingdom of Jordan | 0.49(0.02,1.02) | 0.08(-1.36,3.47) | 0.53(-0.02,0.92) | -0.72(-0.99,-0.56) | 0.02 (0.0,0.04) | 0.18(-1.36,3.81) | 0.02 (-0.0, 0.03) | -0.69 (-0.99, -0.52) |
| Lebanese Republic | 0.34(-0.04,0.85) | 1.48(-7.97,20.83) | 1.19 (0.23,1.82) | -0.67(-0.92,-0.36) | 0.02 (-0.0, 0.04) | 1.96(-9.03,25.84) | 0.05(0.01,0.09) | -0.6 (-0.91, -0.19) |
| State of Libya | 6.48(0.26,14.54) | 0.17(-1.96,2.0) | 3.99 (0.79,7.89) | -0.46 (-0.79,-0.13) | 0.14(0.01,0.31) | 0.29(-1.94,2.31) | 0.09(0.02,0.18) | -0.41 (-0.76, -0.07) |
| Kingdom of Morocco | 1.49 (0.31,3.27) | -0.24(-0.55,0.47) | 2.55(-1.05,5.74) | -0.54(-1.36,-0.19) | 0.06 (0.01,0.12) | 0.0(-0.33,0.89) | 0.1 (-0.04,0.22) | -0.4 (-1.5, 0.08) |
| Sultanate of Oman | 12.16(-5.51,33.92) | -0.27(-0.68,0.63) | 7.48(0.55,17.79) | -0.59(-0.81,-0.31) | 0.47(-0.21,1.31) | -0.23 (-0.62,0.79) | 0.29(0.02,0.68) | -0.57 (-0.76, -0.27) |
| Palestine | 3.95(-0.52,8.76) | 0.93(-8.62,7.39) | 3.21(-0.21,5.94) | -0.57(-0.82,-0.28) | 0.17(-0.02,0.37) | 1.12 (-8.88,8.65) | 0.14(-0.01,0.25) | -0.53 (-0.8, -0.21) |
| State of Qatar | 7.84(-1.03,20.36) | -0.55(-1.06,0.03) | 4.44 (0.17,10.53) | -0.76(-0.87,-0.54) | 0.36(-0.05,0.93) | -0.55 (-1.07,-0.01) | 0.21(0.01,0.49) | -0.76 (-0.86, -0.56) |
| Kingdom of Saudi Arabia | 16.78(0.39,34.7) | -0.13(-0.61,0.78) | 7.82 (1.78,15.92) | -0.59 (-0.73,-0.35) | 0.68(0.02,1.39) | -0.02(-0.57,1.21) | 0.32 (0.07,0.64) | -0.54 (-0.7, -0.25) |
| Syrian Arab Republic | 3.73 (0.96,7.86) | -0.02 (-0.44,0.96) | 3.22 (0.06,6.33) | -0.6(-0.98,-0.28) | 0.12 (0.03,0.27) | 0.16(-0.28,1.21) | 0.1(0.0,0.22) | -0.53 (-0.98, -0.21) |
| Republic of Turkey | 0.55(0.04,1.18) | -0.05(-0.88,0.96) | 2.89(1.72,3.87) | -0.62 (-0.78,-0.35) | 0.02(0.0, 0.04) | 0.15(-0.83,1.28) | 0.1(0.06,0.15) | -0.54 (-0.7, -0.27) |
| Republic of Tunisia | 3.2 (0.56,7.23) | 0.3(-2.36,2.91) | 2.28(0.09,5.15) | -0.52 (-0.86,-0.23) | 0.14(0.03,0.32) | 0.86(-3.04,4.96) | 0.1(0.0,0.23) | -0.31 (-0.81, 0.11) |
| United Arab Emirates | 20.51(-5.2,49.34) | 0.03(-2.26,2.5) | 10.47(1.04,24.08) | -0.6 (-0.76,-0.44) | 0.93 (-0.23,2.2) | 0.2 (-2.4,3.0) | 0.47(0.05,1.09) | -0.54 (-0.73, -0.35) |
| Republic of Yemen | 0.73(-1.01,3.14) | -0.21 (-0.93,0.6) | 1.32 (-1.5,3.49) | -0.46(-1.77,0.2) | 0.03(-0.04,0.11) | -0.14(-0.92,0.71) | 0.04(-0.05,0.12) | -0.41 (-1.83, 0.24) |
| People's Republic of Bangladesh | 4.81 (-1.11, 11.57) | -104.11(-37.22,43.12) | 6.6(2.25, 12.87) | 0.01 (-0.38,1.04) | 0.21 (-0.05,0.49) | -153.82 (-37.16,46.79) | 0.28(0.1,0.54) | 0.06 (-0.31, 1.21) |
| Islamic Republic of Afghanistan | 3.19(0.48,7.23) | 0.16(-0.24,0.83) | 6.85(3.68, 12.34) | -0.22 (-0.48,0.18) | 0.11 (0.02,0.26) | 0.3 (-0.19,1.07) | 0.23 (0.11,0.45) | -0.13 (-0.43, 0.31) |
| Kingdom of Bhutan | 0.16(-0.13,0.53) | 0.76(-0.73,6.24) | 9.06(4.99, 14.54) | -0.13 (-0.5,0.66) | 0.01(-0.01,0.02) | 0.78(-0.7,6.21) | 0.38(0.21,0.6) | -0.12 (-0.47, 0.72) |
| Republic of India | 4.07(-0.94,9.86) | 0.28(-0.3, 2.22) | 6.65(1.96,12.3) | -0.02(-0.25,0.42) | 0.15(-0.04,0.37) | 0.37(-0.14,1.38) | 0.25(0.07,0.46) | 0.07 (-0.17, 0.59) |
| Federal Democratic Republic of Nepal | 2.54(-0.58,6.51) | 0.05(-0.37,0.95) | 6.62 (3.61,11.51) | -0.22(-0.53,0.42) | 0.11 (-0.02,0.26) | 0.13(-0.37,1.14) | 0.27(0.15,0.48) | -0.16 (-0.51, 0.59) |
| Islamic Republic of Pakistan | 17.76(1.67,35.01) | 0.07 (-0.19,0.64) | 14.92 (4.44,26.91) | -0.02 (-0.25,0.49) | 0.67(0.06,1.31) | 0.14(-0.17,0.82) | 0.56(0.17,1.01) | 0.04 (-0.23, 0.67) |
| Republic of Angola | 0.79(-1.87,3.53) | -1.28(-7.45,8.82) | 4.54(-1.32,9.06) | -0.4(-1.21,0.49) | 0.03(-0.07,0.14) | -1.3 (-7.93,9.28) | 0.18(-0.05,0.35) | -0.36 (-1.23, 0.57) |
| Central African Republic | -5.27(-15.32,2.8) | -0.11(-4.84,4.31) | 1.73 (0.38,4.09) | -0.49 (-0.77,0.16) | -0.2(-0.59,0.1) | -0.11 (-4.96,4.12) | 0.07(0.01,0.16) | -0.49 (-0.77, 0.15) |
| Democratic Republic of the Congo | 0.11(-2.82,2.43) | -1.05(-10.24,0.5) | 3.06(0.04,6.63) | -0.47(-0.76,0.35) | 0.0(-0.12,0.1) | -1.06 (-10.48, 0.59) | 0.12 (0.0, 0.28) | -0.44 (-0.76, 0.44) |
| Republic of the Congo | -0.58(-2.75,1.44) | -0.58(-2.76,1.51) | 2.47(1.11,4.26) | -0.64 (-0.79,-0.36) | -0.02 (-0.11,0.06) | -0.57(-2.85,1.53) | 0.1 (0.04,0.17) | -0.63 (-0.78, -0.33) |
| Republic of Equatorial Guinea | -0.57(-2.04,0.21) | -0.44(-1.33,2.3) | 1.27(0.32,2.68) | -0.81(-0.92,-0.56) | -0.02 (-0.08,0.01) | -0.44 (-1.37,2.34) | 0.05(0.01,0.1) | -0.8 (-0.92, -0.54) |
| Gabonese Republic | -0.42 (-1.69,0.25) | -0.32(-5.23,1.25) | 2.02 (0.62,3.38) | -0.72 (-0.84,-0.56) | -0.02 (-0.07,0.01) | -0.33(-5.11,1.21) | 0.08(0.03,0.13) | -0.73 (-0.85, -0.57) |
| Republic of Burundi | 0.01 (-0.1,0.1) | -1.22(-34.05,28.48) | 1.83 (-4.23,4.25) | -0.61(-0.98,-0.1) | 0.0(-0.0,0.0) | -1.25(-40.31,32.62) | 0.06(-0.13,0.12) | -0.56 (-0.98, 0.01) |
| Republic of Djibouti | 6.41(-5.15,23.09) | -0.48(-2.59, 1.4) | 4.01(0.18, 10.7) | -0.22 (-0.63,0.27) | 0.2(-0.15,0.67) | -0.45(-2.61,1.51) | 0.12 (0.01,0.32) | -0.17 (-0.6, 0.34) |
| State of Eritrea | 2.01(-2.71,8.4) | 1.97(-7.65, 11.84) | 2.8(0.32,5.81) | -0.31(-0.73,0.44) | 0.06(-0.08,0.25) | 2.38(-9.08,13.34) | 0.08(0.01,0.18) | -0.22 (-0.7, 0.66) |
| Union of the Comoros | -0.34(-0.86,0.04) | -0.57(-1.24,0.28) | 2.33(1.04,3.88) | -0.36(-0.64,0.17) | -0.01(-0.03,0.0) | -0.54(-1.26,0.44) | 0.07(0.03,0.11) | -0.3 (-0.62, 0.27) |
| Republic of Kenya | -0.39(-0.6, - 0.21) | 0.34(-0.42,2.63) | 1.59(-1.44,2.77) | -0.41(-1.24,0.05) | -0.01 (-0.02,-0.01) | 0.34(-0.44,2.55) | 0.05(-0.04,0.09) | -0.4 (-1.28, 0.05) |
| Republic of Malawi | 2.01(-0.75,5.09) | 1.66(-3.08,12.33) | 3.81(0.17,6.83) | 0.12 (-2.28,1.08) | 0.06(-0.02,0.15) | 1.9 (-3.48,12.06) | 0.11 (0.01,0.2) | 0.23 (-2.44, 1.26) |
| Republic of Madagascar | 0.92(-0.74,2.77) | -7.67(-22.96,37.65) | 5.84(-0.6, 10.22) | -0.32 (-0.73,0.29) | 0.03(-0.02,0.08) | -7.8(-22.66,40.55) | 0.16(-0.02,0.28) | -0.3 (-0.73, 0.38) |
| Republic of Mauritius | 0.24(-0.12,0.68) | N/A | 0.7(-3.0,1.97) | 1.35(-5.77,4.4) | 0.01 (-0.0,0.03) | N/A | 0.03(-0.11,0.08) | 1.26 (-5.56, 4.27) |
| Federal Democratic Republic of Ethiopia | -0.03(-0.38,0.4) | -0.2(-3.83,3.29) | 2.77(0.23,4.42) | -0.46(-0.81,0.16) | -0.0(-0.01,0.01) | -0.69 (-4.25,3.24) | 0.09 (0.01,0.14) | -0.42 (-0.79, 0.24) |
| Republic of Mozambique | 2.19(-0.52,4.79) | 1.81 (-8.19,8.42) | 3.05(1.24,5.15) | 0.06(-0.42,0.63) | 0.07(-0.02,0.15) | 1.91(-8.26,8.34) | 0.09(0.04,0.15) | 0.1 (-0.45, 0.74) |
| Federal Republic of Somalia | -6.08(-11.64,-2.61) | 0.21 (-0.77,6.11) | 1.04 (0.22,2.18) | -0.32 (-0.91,0.33) | -0.18(-0.34,-0.08) | 0.26(-0.74,6.04) | 0.03 (0.01,0.06) | -0.29 (-0.91, 0.46) |
| Republic of Seychelles | -0.95(-1.29,-0.73) | -0.43(-0.56,-0.26) | 1.74(1.04,2.39) | -0.54(-0.64,-0.41) | -0.04(-0.05,-0.03) | -0.42 (-0.56,-0.23) | 0.08(0.05,0.11) | -0.53 (-0.63, -0.38) |
| Republic of Rwanda | 0.01(-0.02,0.05) | N/A | 1.89(-3.54,3.98) | -0.69(-1.29,0.06) | 0.0(-0.0,0.0) | N/A | 0.06(-0.11,0.12) | -0.66 (-1.34, 0.17) |
| United Republic of Tanzania | 0.12(-0.37,0.65) | -1.61 (-32.43, 22.46) | 2.01 (-0.62,3.83) | -0.41 (-1.17,0.12) | 0.0(-0.01,0.02) | -1.6 (-32.89,22.73) | 0.06(-0.02,0.11) | -0.41 (-1.19, 0.16) |
| Republic of Uganda | 0.12 (-0.15,0.38) | -2.53(-82.55,99.76) | 1.2 (-0.88,2.51) | -0.64 (-1.02,-0.35) | 0.0(-0.0,0.01) | -2.58(-87.62,98.56) | 0.03 (-0.03,0.07) | -0.62 (-1.02, -0.3) |
| Republic of Zambia | 3.49 (-1.65,9.69) | 2.75(-0.38, 10.21) | 6.79 (-0.44, 14.05) | 1.02 (-2.38,2.78) | 0.11(-0.05,0.29) | 2.85(-0.36,9.73) | 0.21(-0.01,0.42) | 1.07 (-2.59, 2.93) |
| Kingdom of Lesotho | 0.0(-0.03,0.03) | N/A | 25.07(14.25,36.89) | -0.07(-0.42,0.43) | 0.0(-0.0,0.0) | N/A | 1.11 (0.62, 1.62) | -0.17 (-0.49, 0.3) |
| Republic of Botswana | 3.88(-1.72,9.54) | -0.49 (-0.67,-0.13) | 10.95(4.64,18.96) | -0.25(-0.54,0.19) | 0.18(-0.08,0.44) | -0.49 (-0.67,-0.13) | 0.5 (0.21,0.84) | -0.25 (-0.55, 0.21) |
| Republic of Namibia | 4.88(-0.87,11.03) | 0.29(-0.13,0.93) | 9.71 (4.25, 16.18) | -0.13(-0.43,0.26) | 0.22 (-0.04,0.5) | 0.27(-0.14,0.89) | 0.44(0.19,0.74) | -0.15 (-0.45, 0.22) |
| Republic of South Africa | 0.77(-0.41,2.13) | -0.24(-0.93,0.44) | 12.93(6.17,17.22) | -0.32 (-0.53,-0.12) | 0.03 (-0.02,0.09) | -0.22 (-0.94,0.46) | 0.55(0.26,0.73) | -0.34 (-0.56, -0.07) |
| Kingdom of Eswatini | 1.01(-0.77,2.95) | 0.12(-0.55,1.72) | 10.79(4.61,17.76) | -0.31 (-0.6,0.11) | 0.04(-0.03,0.12) | 0.01 (-0.58,1.41) | 0.44 (0.19,0.71) | -0.38 (-0.64, -0.01) |
| Republic of Benin | -4.6(-11.36,0.83) | -0.39(-3.29,5.72) | 0.47(-0.02,1.38) | -0.81(-1.32,0.12) | -0.18(-0.44,0.03) | -0.39 (-3.2,5.44) | 0.02 (-0.0,0.05) | -0.82 (-1.33, 0.12) |
| Republic of Zimbabwe | 4.77(-1.14,11.36) | 1.12 (-0.07,3.63) | 11.71 (-2.18,20.79) | 0.27(-0.79,1.65) | 0.2 (-0.05,0.48) | 0.86(-0.21,3.26) | 0.5(-0.09,0.87) | 0.11 (-0.82, 1.41) |
| Burkina Faso | -0.78(-14.6,20.09) | -0.83(-8.35, 10.44) | 1.14(-0.5,3.62) | -0.79 (-1.22,-0.09) | -0.03(-0.62,0.88) | -0.83 (-8.35,11.82) | 0.05(-0.02,0.16) | -0.79 (-1.22, -0.04) |
| Republic of Cameroon | 0.35(-3.34,5.64) | -1.48(-6.3,9.82) | 2.01(-0.43,4.46) | -0.63(-0.9,-0.31) | 0.01(-0.13,0.21) | -1.46(-6.03,9.45) | 0.08(-0.02,0.17) | -0.65 (-0.9, -0.33) |
| Republic of Chad | 3.15(-9.16,24.26) | 0.41(-3.73,4.59) | 3.27(0.09,9.11) | -0.47(-0.78,-0.09) | 0.12 (-0.36,0.96) | 0.37(-3.64,4.7) | 0.13(0.0,0.35) | -0.48 (-0.79, -0.09) |
| Republic of Cabo Verde | 0.07(-0.11,0.28) | N/A | 1.25(-1.7,2.85) | -0.08(-1.4,0.24) | 0.0(-0.0,0.01) | N/A | 0.05(-0.07,0.11) | -0.04 (-1.49, 0.49) |
| Republic of Côte d'Ivoire | -3.83(-7.62,-1.56) | -0.22 (-3.28,8.83) | 0.97(0.21,1.95) | -0.73(-0.88,0.47) | -0.15(-0.28,-0.06) | -0.25 (-3.13,8.08) | 0.04(0.01,0.07) | -0.74 (-0.88, 0.48) |
| Republic of the Gambia | -2.89(-8.12,3.4) | -0.33(-3.19,2.23) | 2.34(1.02,4.14) | -0.56(-0.76,-0.12) | -0.11(-0.31,0.13) | -0.34 (-3.13,2.18) | 0.09(0.04,0.16) | -0.56 (-0.76, -0.13) |
| Republic of Ghana | -5.85(-12.92,2.09) | -0.02 (-1.58,2.71) | 1.28(0.15,2.55) | -0.76(-0.92,-0.55) | -0.23(-0.5,0.09) | -0.0(-1.59,2.51) | 0.05(0.01,0.09) | -0.76 (-0.91, -0.56) |
| Republic of Guinea | 0.3(-3.2,4.49) | -1.39(-2.48,2.71) | 2.04(0.88,3.62) | -0.59(-0.75,-0.28) | 0.01(-0.12,0.17) | -1.4 (-2.4, 2.73) | 0.08(0.03,0.14) | -0.58 (-0.76, -0.25) |
| Republic of Liberia | -0.68(-2.35,0.67) | -0.43(-2.85,0.99) | 1.36(0.59,2.51) | -0.67(-0.83,-0.33) | -0.03(-0.09,0.03) | -0.4(-3.01,1.08) | 0.05(0.02,0.1) | -0.65 (-0.82, -0.28) |
| Republic of Guinea-Bissau | -12.54(-19.22,-6.44) | -0.27(-0.56,0.57) | 0.42 (-0.26, 1.52) | -0.8(-1.14,-0.37) | -0.46 (-0.7,-0.24) | -0.28(-0.56,0.55) | 0.02 (-0.01,0.06) | -0.8 (-1.14, -0.41) |
| Republic of Mali | 4.24(-9.32,22.45) | 0.02 (-1.35,1.51) | 1.02(-0.34,2.98) | -0.82 (-1.47,-0.47) | 0.16(-0.35,0.86) | 0.02(-1.35,1.47) | 0.04(-0.01,0.12) | -0.82 (-1.47, -0.47) |
| Islamic Republic of Mauritania | 18.01 (-9.49,51.52) | -0.12 (-2.09,1.26) | 3.28(0.64,7.22) | -0.68(-0.78,-0.48) | 0.74(-0.39,2.16) | -0.09 (-2.12,1.27) | 0.14(0.03,0.29) | -0.67 (-0.78, -0.48) |
| Republic of the Niger | 7.05 (-11.08, 34.34) | -0.23(-2.76,4.04) | 5.25 (0.32, 14.43) | -0.53(-0.88,-0.11) | 0.3(-0.48,1.43) | -0.18(-2.78,4.35) | 0.22 (0.01,0.6) | -0.5 (-0.87, -0.05) |
| Federal Republic of Nigeria | -1.23(-4.56,3.98) | -0.47(-3.14,1.57) | 1.88(0.42,3.94) | -0.66 (-0.8, - 0.47) | -0.05(-0.17,0.13) | -0.49 (-2.1,1.97) | 0.07(0.02,0.14) | -0.7 (-0.81, -0.54) |
| Republic of Senegal | -1.44 (-11.71, 11.07) | -0.47(-3.96, 6.9) | 1.91 (0.27,4.11) | -0.67(-0.82,-0.34) | -0.06(-0.46,0.42) | -0.45(-4.09,7.41) | 0.08(0.01,0.16) | -0.66 (-0.83, -0.33) |
| Democratic Republic of Sao Tome and Principe | 0.14(-0.74,0.8) | -1.84(-1.7,2.8) | 0.81(0.59,1.18) | -0.72(-0.85,-0.42) | 0.01 (-0.03,0.03) | -1.84 (-1.81,3.2) | 0.03(0.02,0.04) | -0.73 (-0.85, -0.44) |
| Republic of Sierra Leone | -1.25(-4.07,0.91) | 0.04(-7.02,6.96) | 1.71(0.65,2.97) | -0.64(-0.8, - 0.36) | -0.05(-0.15,0.03) | 0.03(-6.71,6.81) | 0.06(0.02,0.11) | -0.64 (-0.81, -0.36) |
| Togolese Republic | -4.8(-9.91,0.37) | -0.45(-1.35,1.49) | 0.7(0.1,1.52) | -0.76(-0.9,-0.26) | -0.19(-0.38,0.01) | -0.45(-1.37,1.47) | 0.03 (0.0,0.06) | -0.76 (-0.9, -0.23) |
| American Samoa | -0.72 (-0.96,-0.49) | -0.48(-0.85,0.52) | 1.74(0.92,2.46) | 0.11(-0.33,7.19) | -0.02 (-0.03,-0.02) | -0.51 (-0.86,0.44) | 0.06(0.03,0.08) | 0.06 (-0.37, 6.68) |
| Cook Islands | -0.27(-0.37,-0.17) | -0.48(-0.68,-0.26) | 0.01 (0.0,0.01) | 0.17(-0.39,0.67) | -0.01(-0.01,-0.0) | -0.51 (-0.67,-0.29) | 0.0 (0.0,0.0) | 0.12 (-0.37, 0.59) |
| Bermuda | 1.21 (-0.84,3.44) | 1.68(-1.36,6.43) | 4.04(1.99,6.03) | 0.09 (-0.3, 1.09) | 0.05(-0.04,0.15) | 1.81(-1.38,6.62) | 0.17(0.08,0.25) | 0.14 (-0.26, 1.14) |
| Greenland | 0.23 (-1.15,1.55) | -1.31 (-4.81,6.12) | 10.96(-1.78,24.59) | -0.32 (-4.25,1.9) | 0.01(-0.04,0.06) | -1.36 (-5.29,7.15) | 0.39 (-0.06,0.9) | -0.23 (-4.64, 2.37) |
| Guam | -3.45(-4.4,-1.21) | -0.2(-0.72,0.05) | 0.03(0.0,0.31) | -0.29(-0.92,6.56) | -0.09 (-0.11,-0.03) | -0.4 (-0.79,-0.24) | 0.0(0.0,0.01) | -0.47 (-0.94, 4.66) |
| Republic of Nauru | -8.24 (-10.97,-6.03) | 0.26(-0.08,0.85) | 0.03(0.02,0.05) | -0.68(-0.77,-0.49) | -0.3(-0.43,-0.2) | 0.42 (-0.06,1.12) | 0.0(0.0,0.0) | -0.63 (-0.76, -0.38) |
| Republic of Niue | -1.06(-1.35,-0.43) | 0.57(-0.01,2.39) | 1.51(0.09,2.4) | -0.05(-0.66,0.6) | -0.02 (-0.03,-0.01) | 0.11(-0.29,1.37) | 0.03(0.0, 0.06) | -0.33 (-0.76, 0.08) |
| Principality of Monaco | 0.12 (-0.07,0.56) | -0.07(-1.06,2.01) | 8.6(4.1, 12.41) | -0.48(-0.64,-0.03) | 0.01 (-0.0,0.03) | -0.06 (-1.06,2.01) | 0.44(0.22,0.64) | -0.48 (-0.64, -0.02) |
| Northern Mariana Islands | -2.93(-3.56,-2.22) | -0.07(-0.51,0.72) | 0.79 (0.43,1.19) | -0.29(-0.53,0.17) | -0.1 (-0.13,-0.08) | -0.08(-0.5,0.71) | 0.03(0.01,0.04) | -0.29 (-0.52, 0.19) |
| Saint Kitts and Nevis | -2.13(-2.67,-1.65) | 0.08(-0.14,0.34) | 1.85(1.1, 2.51) | -0.49(-0.61,-0.38) | -0.09(-0.11,-0.07) | 0.23 (0.01,0.47) | 0.07(0.04,0.1) | -0.42 (-0.54, -0.3) |
| Republic of Palau | -2.52 (-3.49,-1.04) | 0.02 (-0.3, 0.43) | 0.11(0.05,0.39) | -0.5(-0.68,0.66) | 0.08(-0.1,-0.03) | 0.01(-0.33,0.42) | 0.0(0.0,0.01) | -0.51 (-0.68, 0.59) |
| Puerto Rico | -0.43(-0.57,-0.33) | -0.54(-0.65,-0.43) | 0.27(0.16,0.36) | -0.82 (-0.85,-0.79) | -0.02 (-0.02,-0.01) | -0.57(-0.67,-0.47) | 0.01(0.01,0.01) | -0.83 (-0.86, -0.8) |
| Republic of San Marino | 0.42 (-0.09,1.18) | 0.5(-3.19,5.74) | 4.95(3.0, 7.78) | -0.64(-0.77,-0.42) | 0.02(-0.0,0.06) | 0.49(-3.12,5.95) | 0.27(0.16,0.41) | -0.64 (-0.77, -0.43) |
| Tokelau | -7.94(-11.39,-2.28) | 0.5(0.0,1.4) | 0.2 (0.1, 1.12) | 0.27(-0.15, 1.12) | -0.19 (-0.27,-0.06) | 0.09 (-0.3,0.73) | 0.0(0.0,0.03) | -0.09 (-0.39, 0.5) |
| Tuvalu | -4.52(-6.09,-3.06) | -0.14(-0.4, 1.94) | 0.12 (0.07,0.17) | -0.59(-0.89,-0.07) | -0.14(-0.2, - 0.1) | -0.14 (-0.39,1.97) | 0.0 (0.0,0.01) | -0.59 (-0.89, -0.09) |
| United States Virgin Islands | -1.37(-1.92,-0.97) | -0.4(-0.8,0.08) | 1.19(0.68, 1.8) | -0.59(-0.73,0.46) | -0.04(-0.06,-0.03) | -0.46 (-0.81,-0.03) | 0.04(0.02,0.05) | -0.63 (-0.75, 0.33) |
| Republic of South Sudan | -1.4(-9.04,8.97) | -0.64(-5.32,1.89) | 1.14(-0.16,3.04) | -0.56(-0.99,-0.01) | -0.04(-0.23,0.24) | -0.66 (-5.15,1.74) | 0.03(-0.0, 0.08) | -0.58 (-0.99, -0.08) |
| Republic of Sudan | 5.17(-4.64,18.33) | -0.19(-1.54, 1.39) | 1.89(-0.02,4.87) | -0.47(-0.72,0.07) | 0.16(-0.14,0.56) | -0.09 (-1.57,1.83) | 0.06 (-0.0, 0.15) | -0.41 (-0.67, 0.06) |
| DALYs: Disability-adjusted life years. ASDR: Age-Standardized Death Rate. ASMR: Age-Standardized Mortality Rate. TPC: Total Percent Change. | | | | | | | | |

| **Table S2. Comprehensive data on DALYs and mortality rates (ASDR, ASMR), and TPC values for ischemic heart disease attributable to high and low temperatures across**  **countries in 1990 vs. 2021.** | | | | | | | | |
| --- | --- | --- | --- | --- | --- | --- | --- | --- |
| **Location** | Ischemic Heart Disease attributable to  High temperature | | Ischemic Heart Disease attributable to  Low temperature | | Ischemic Heart Disease attributable to  High temperature | | Ischemic Heart Disease attributable to  Low temperature | |
|  | ASDR (95% UI) | TPC (95% UI) | ASDR (95% UI) | TPC (95% UI) | ASMR (95% UI) | TPC (95% UI) | ASMR (95% UI) | TPC (95% UI) |
| Democratic People's Republic of Korea | 16.62 (-1.47, 60.95) | 0.73 (-1.95, 3.6) | 156.26 (111.98, 209.47) | 0.12 (-0.16, 0.48) | 0.82 (-0.07, 3.12) | 0.67 (-1.92, 3.36) | 7.66 (5.48, 10.34) | 0.08 (-0.15, 0.39) |
| People's Republic of China | 13.93 (-5.46, 50.36) | 0.47 (-2.77, 2.88) | 128.6 (104.68, 158.35) | -0.01 (-0.19, 0.24) | 0.87 (-0.33, 3.13) | 0.64 (-2.79, 3.6) | 7.92 (6.46, 9.67) | 0.12 (-0.08, 0.38) |
| Taiwan (Province of China) | 4.71 (-9.65, 22.49) | 0.52 (-3.81, 2.41) | 29.25 (22.69, 35.58) | -0.54 (-0.58, -0.49) | 0.25 (-0.5, 1.17) | 0.41 (-3.63, 2.15) | 1.53 (1.14, 1.88) | -0.58 (-0.61, -0.53) |
| Republic of Maldives | 3.95 (-0.0, 10.36) | 2.16 (-22.46, 23.9) | 0.38 (0.19, 1.25) | -0.88 (-0.93, -0.62) | 0.22 (-0.0, 0.57) | 2.97 (-27.82, 29.15) | 0.02 (0.01, 0.07) | -0.85 (-0.91, -0.53) |
| Lao People's Democratic Republic | 22.23 (-1.08, 52.11) | 4.56 (-15.28, 12.93) | 111.67 (80.41, 149.99) | -0.31 (-0.5, -0.0) | 1.08 (-0.05, 2.54) | 5.26 (-17.14, 14.4) | 5.43 (4.0, 7.11) | -0.23 (-0.42, 0.07) |
| Democratic Republic of Timor-Leste | -0.52 (-2.02, 0.53) | -0.95 (-1.11, -0.64) | 43.93 (29.84, 60.28) | 0.22 (-0.22, 1.22) | -0.03 (-0.1, 0.03) | -0.95 (-1.12, -0.63) | 2.21 (1.5, 3.02) | 0.29 (-0.16, 1.36) |
| Republic of Indonesia | 3.23 (1.68, 5.65) | -1.87 (-27.07, 18.15) | 26.28 (19.24, 35.02) | -0.08 (-0.3, 0.35) | 0.16 (0.09, 0.27) | -2.11 (-36.36, 27.48) | 1.26 (0.93, 1.65) | 0.0 (-0.25, 0.48) |
| Republic of the Philippines | 23.26 (18.18, 28.94) | -6.12 (-17.63, 35.21) | 14.43 (10.89, 18.51) | -0.23 (-0.4, 0.24) | 1.05 (0.82, 1.32) | -6.18 (-22.11, 35.12) | 0.66 (0.51, 0.85) | -0.29 (-0.44, 0.11) |
| Democratic Socialist Republic of Sri Lanka | 5.83 (3.42, 7.91) | 1.27 (-19.88, 7.41) | 20.81 (13.3, 29.86) | -0.36 (-0.58, -0.05) | 0.29 (0.18, 0.38) | 1.27 (-20.27, 7.35) | 1.03 (0.68, 1.43) | -0.35 (-0.57, -0.07) |
| Kingdom of Cambodia | 42.47 (-5.72, 61.51) | 6.27 (-26.89, 14.07) | 31.36 (16.52, 45.95) | 0.39 (-0.13, 1.39) | 2.14 (-0.29, 3.07) | 7.3 (-29.71, 15.74) | 1.58 (0.83, 2.31) | 0.56 (-0.0, 1.67) |
| Republic of Fiji | -1.61 (-7.27, 3.34) | -0.24 (-4.61, 3.21) | 84.63 (58.46, 127.65) | -0.46 (-0.61, -0.25) | -0.07 (-0.33, 0.15) | -0.18 (-4.86, 3.57) | 3.82 (2.71, 5.63) | -0.42 (-0.58, -0.21) |
| Republic of Kiribati | -9.67 (-30.49, -4.09) | -0.01 (-0.76, 2.26) | 10.33 (3.23, 15.05) | 7.97 (2.39, 12.81) | -0.4 (-1.24, -0.17) | 0.01 (-0.75, 2.3) | 0.43 (0.14, 0.61) | 8.21 (2.53, 13.06) |
| Republic of the Marshall Islands | 7.07 (-5.76, 21.7) | -1.49 (-5.22, -0.71) | 0.32 (0.22, 0.45) | -0.82 (-0.87, -0.76) | 0.3 (-0.25, 0.92) | -1.49 (-5.27, -0.7) | 0.01 (0.01, 0.02) | -0.82 (-0.86, -0.77) |
| Socialist Republic of Viet Nam | 19.24 (-2.84, 44.1) | 3.59 (-18.71, 16.4) | 39.47 (28.8, 51.69) | 0.22 (-0.09, 0.64) | 1.04 (-0.15, 2.41) | 3.82 (-19.29, 17.35) | 2.13 (1.57, 2.75) | 0.28 (-0.02, 0.68) |
| Malaysia | 11.09 (8.36, 18.76) | 5.56 (-3.0, 23.62) | 9.9 (7.84, 13.12) | -0.48 (-0.56, -0.28) | 0.53 (0.4, 0.9) | 5.79 (-3.06, 24.49) | 0.48 (0.37, 0.63) | -0.46 (-0.54, -0.25) |
| Independent State of Samoa | -1.16 (-1.9, -0.6) | -0.57 (-0.95, 0.75) | 37.96 (28.49, 50.65) | 0.16 (-0.14, 1.91) | -0.05 (-0.09, -0.03) | -0.58 (-0.95, 0.66) | 1.74 (1.32, 2.32) | 0.15 (-0.12, 1.82) |
| Republic of the Union of Myanmar | 52.59 (24.42, 88.85) | 0.4 (-1.37, 1.1) | 52.94 (38.61, 71.58) | -0.58 (-0.7, -0.41) | 2.66 (1.24, 4.45) | 0.59 (-1.42, 1.3) | 2.67 (1.97, 3.57) | -0.53 (-0.64, -0.34) |
| Kingdom of Thailand | 15.68 (5.56, 25.94) | -4.9 (-65.3, 0.9) | 18.19 (10.99, 26.92) | 0.03 (-0.25, 0.44) | 0.74 (0.26, 1.22) | -4.6 (-58.53, 0.75) | 0.86 (0.52, 1.27) | -0.05 (-0.3, 0.31) |
| Federated States of Micronesia | 6.08 (-5.98, 20.18) | -1.45 (-4.95, 6.64) | 0.4 (0.25, 0.58) | -0.7 (-0.79, -0.58) | 0.26 (-0.25, 0.85) | -1.46 (-5.08, 6.59) | 0.02 (0.01, 0.02) | -0.7 (-0.79, -0.59) |
| Solomon Islands | -11.24 (-47.71, 0.3) | -0.12 (-1.27, 5.11) | 9.59 (4.61, 16.13) | -0.6 (-0.75, -0.3) | -0.5 (-2.09, 0.01) | -0.11 (-1.27, 5.06) | 0.43 (0.21, 0.71) | -0.6 (-0.74, -0.33) |
| Independent State of Papua New Guinea | -0.39 (-5.54, 1.56) | -0.83 (-4.7, 5.11) | 118.78 (84.52, 160.08) | -0.13 (-0.38, 0.25) | -0.02 (-0.24, 0.07) | -0.83 (-4.9, 5.36) | 5.16 (3.71, 6.95) | -0.1 (-0.34, 0.24) |
| Kingdom of Tonga | -0.26 (-1.65, 1.28) | -0.95 (-2.78, -0.43) | 2.75 (1.68, 3.96) | -0.36 (-0.57, 2.59) | -0.01 (-0.08, 0.06) | -0.95 (-2.79, -0.43) | 0.13 (0.08, 0.18) | -0.34 (-0.55, 2.65) |
| Republic of Vanuatu | -2.29 (-16.0, -0.63) | -0.66 (-0.96, 0.24) | 63.62 (45.12, 85.38) | -0.09 (-0.35, 0.35) | -0.1 (-0.69, -0.03) | -0.66 (-0.96, 0.23) | 2.75 (1.92, 3.66) | -0.09 (-0.32, 0.33) |
| Republic of Armenia | 21.64 (1.45, 70.17) | 0.24 (-4.33, 4.36) | 226.37 (193.06, 277.81) | -0.39 (-0.46, -0.32) | 1.22 (0.08, 4.0) | 0.27 (-4.41, 4.5) | 12.78 (10.86, 15.82) | -0.38 (-0.44, -0.31) |
| Republic of Azerbaijan | 49.21 (3.13, 170.13) | 0.82 (-9.52, 10.86) | 425.96 (363.64, 513.62) | -0.25 (-0.35, -0.15) | 2.78 (0.17, 9.46) | 1.0 (-10.44, 12.04) | 24.02 (20.6, 29.15) | -0.18 (-0.27, -0.08) |
| Georgia | 12.38 (-2.02, 50.19) | -0.26 (-1.34, 3.84) | 157.06 (129.94, 185.39) | -0.68 (-0.74, -0.64) | 0.61 (-0.1, 2.48) | -0.31 (-1.33, 3.51) | 7.79 (6.4, 9.22) | -0.7 (-0.75, -0.66) |
| Mongolia | 1.75 (-0.29, 8.46) | 0.89 (-3.09, 7.82) | 293.08 (228.97, 396.49) | -0.37 (-0.48, -0.22) | 0.1 (-0.02, 0.49) | 1.0 (-3.18, 8.35) | 16.83 (13.03, 22.75) | -0.33 (-0.43, -0.18) |
| Republic of Tajikistan | 57.65 (5.86, 158.64) | 0.4 (-5.77, 4.59) | 327.9 (266.11, 419.05) | -0.31 (-0.43, -0.16) | 3.26 (0.33, 9.06) | 0.53 (-6.12, 5.05) | 18.52 (15.06, 23.46) | -0.25 (-0.37, -0.1) |
| Republic of Kazakhstan | 29.1 (-0.29, 87.3) | 0.18 (-3.77, 4.6) | 260.38 (216.07, 322.22) | -0.35 (-0.44, -0.25) | 1.76 (-0.02, 5.3) | 0.38 (-4.18, 5.52) | 15.77 (13.2, 19.42) | -0.24 (-0.34, -0.14) |
| Turkmenistan | 200.83 (57.29, 455.73) | 0.32 (-0.09, 3.6) | 556.82 (433.96, 734.69) | -0.21 (-0.38, -0.02) | 10.72 (3.04, 24.27) | 0.34 (-0.05, 3.7) | 29.72 (23.57, 38.98) | -0.19 (-0.35, -0.02) |
| Republic of Uzbekistan | 110.67 (19.57, 310.76) | 0.65 (-0.93, 4.89) | 518.09 (432.16, 652.21) | -0.03 (-0.16, 0.11) | 6.11 (1.09, 17.1) | 0.73 (-0.91, 5.2) | 28.6 (23.92, 35.65) | 0.02 (-0.11, 0.15) |
| Republic of Albania | 9.82 (-0.98, 37.93) | 2.28 (-19.98, 39.89) | 189.34 (157.71, 227.31) | -0.16 (-0.31, 0.01) | 0.63 (-0.06, 2.39) | 2.7 (-22.06, 44.86) | 12.05 (9.98, 14.5) | -0.06 (-0.22, 0.14) |
| Bosnia and Herzegovina | 9.68 (-0.95, 35.52) | 0.57 (-8.49, 13.87) | 152.92 (115.71, 200.01) | -0.39 (-0.51, -0.26) | 0.56 (-0.05, 2.05) | 0.77 (-9.49, 15.65) | 8.89 (6.87, 11.6) | -0.31 (-0.44, -0.17) |
| Kyrgyz Republic | 17.5 (-1.64, 62.77) | 0.31 (-1.28, 2.19) | 331.53 (273.17, 415.11) | -0.06 (-0.22, 0.11) | 1.02 (-0.09, 3.7) | 0.47 (-1.31, 2.56) | 19.27 (15.74, 24.09) | 0.05 (-0.11, 0.22) |
| Republic of Bulgaria | 19.12 (-2.7, 75.03) | 0.03 (-3.95, 7.79) | 257.75 (209.72, 331.61) | -0.43 (-0.51, -0.33) | 1.03 (-0.15, 4.1) | -0.08 (-3.6, 6.72) | 13.91 (11.34, 17.68) | -0.49 (-0.56, -0.42) |
| Republic of Croatia | 12.31 (-1.15, 46.3) | 0.04 (-5.12, 8.94) | 152.41 (124.04, 196.53) | -0.56 (-0.61, -0.51) | 0.79 (-0.07, 2.97) | 0.11 (-5.41, 9.84) | 9.82 (8.01, 12.52) | -0.53 (-0.58, -0.48) |
| Czech Republic | 2.05 (0.04, 10.19) | -0.43 (-1.78, 1.35) | 127.71 (109.24, 155.62) | -0.63 (-0.67, -0.57) | 0.13 (0.0, 0.64) | -0.31 (-1.94, 1.83) | 8.0 (6.77, 9.76) | -0.55 (-0.6, -0.49) |
| North Macedonia | 16.1 (-2.54, 61.46) | 0.58 (-7.11, 10.77) | 205.5 (155.72, 266.13) | -0.26 (-0.39, -0.1) | 1.02 (-0.16, 3.87) | 1.0 (-8.5, 13.73) | 13.05 (10.01, 16.84) | -0.06 (-0.21, 0.12) |
| Hungary | 20.25 (0.11, 67.91) | 0.18 (-2.83, 5.76) | 196.08 (155.12, 261.58) | -0.44 (-0.5, -0.37) | 1.16 (0.01, 3.88) | 0.33 (-3.04, 6.52) | 11.22 (8.94, 14.93) | -0.37 (-0.44, -0.3) |
| Montenegro | 11.27 (-2.43, 44.2) | 4.11 (-77.04, 67.98) | 250.11 (211.14, 294.34) | 0.12 (-0.05, 0.33) | 0.69 (-0.15, 2.75) | 5.08 (-91.57, 82.44) | 15.42 (12.92, 18.07) | 0.33 (0.14, 0.58) |
| Republic of Poland | 2.15 (0.14, 10.11) | -0.07 (-6.42, 8.84) | 117.16 (102.7, 140.17) | -0.62 (-0.66, -0.59) | 0.13 (0.01, 0.6) | 0.12 (-7.77, 11.46) | 6.97 (5.98, 8.41) | -0.55 (-0.59, -0.51) |
| Romania | 14.28 (-1.86, 53.6) | 0.1 (-3.87, 4.22) | 193.06 (159.26, 245.37) | -0.39 (-0.45, -0.32) | 0.8 (-0.1, 2.96) | 0.07 (-3.8, 3.95) | 10.75 (8.85, 13.63) | -0.4 (-0.46, -0.35) |
| Republic of Serbia | 18.76 (-2.78, 68.59) | 0.21 (-5.14, 7.08) | 209.84 (167.01, 272.22) | -0.41 (-0.5, -0.31) | 1.14 (-0.17, 4.12) | 0.22 (-5.17, 7.07) | 12.72 (10.0, 16.58) | -0.41 (-0.5, -0.3) |
| Slovak Republic | 8.35 (0.11, 31.08) | 0.19 (-3.32, 4.7) | 193.73 (162.38, 244.43) | -0.51 (-0.57, -0.44) | 0.51 (0.01, 1.9) | 0.39 (-3.75, 5.76) | 11.91 (10.03, 14.96) | -0.42 (-0.49, -0.35) |
| Republic of Belarus | 8.1 (0.88, 32.28) | 13.18 (-244.55, 1544.42) | 388.06 (313.48, 488.87) | 0.12 (-0.09, 0.35) | 0.45 (0.05, 1.77) | 13.2 (-240.84, 1546.28) | 21.48 (17.5, 26.95) | 0.13 (-0.07, 0.33) |
| Republic of Estonia | 1.22 (-0.4, 5.17) | N/A | 84.16 (69.69, 104.2) | -0.77 (-0.82, -0.74) | 0.08 (-0.02, 0.32) | N/A | 5.21 (4.3, 6.46) | -0.75 (-0.8, -0.72) |
| Republic of Latvia | 2.37 (0.12, 9.63) | N/A | 168.31 (144.97, 199.49) | -0.52 (-0.58, -0.46) | 0.13 (0.01, 0.54) | N/A | 9.48 (8.13, 11.16) | -0.5 (-0.56, -0.44) |
| Republic of Moldova | 22.73 (-0.23, 80.99) | -0.09 (-3.36, 2.18) | 300.14 (240.99, 387.78) | -0.27 (-0.34, -0.2) | 1.21 (-0.01, 4.31) | -0.19 (-3.1, 1.86) | 16.03 (12.8, 20.78) | -0.35 (-0.41, -0.29) |
| Republic of Slovenia | 2.0 (-0.27, 8.58) | -0.15 (-5.2, 6.33) | 48.63 (40.09, 62.76) | -0.68 (-0.72, -0.64) | 0.12 (-0.02, 0.53) | -0.04 (-5.79, 7.25) | 2.98 (2.4, 3.79) | -0.64 (-0.68, -0.6) |
| Russian Federation | 11.51 (-0.18, 39.85) | 1.29 (-24.55, 17.88) | 263.01 (226.52, 332.04) | -0.3 (-0.37, -0.24) | 0.63 (-0.01, 2.16) | 1.27 (-14.49, 28.8) | 13.89 (11.93, 17.6) | -0.3 (-0.38, -0.24) |
| Brunei Darussalam | 1.78 (-0.43, 4.18) | -1.73 (-14.73, 7.84) | 3.66 (2.77, 4.8) | -0.68 (-0.75, -0.57) | 0.09 (-0.02, 0.2) | -1.75 (-15.04, 8.07) | 0.18 (0.14, 0.24) | -0.67 (-0.74, -0.57) |
| Ukraine | 24.4 (2.21, 85.24) | 2.18 (-9.8, 18.03) | 428.79 (321.11, 569.86) | 0.22 (-0.06, 0.5) | 1.41 (0.13, 5.15) | 2.18 (-10.06, 17.84) | 24.81 (18.79, 32.62) | 0.22 (-0.04, 0.48) |
| Japan | 1.68 (-0.69, 6.78) | -0.67 (-1.11, -0.13) | 36.05 (31.87, 39.25) | -0.59 (-0.6, -0.57) | 0.09 (-0.03, 0.36) | -0.71 (-1.11, -0.29) | 1.9 (1.6, 2.12) | -0.64 (-0.65, -0.62) |
| Republic of Korea | 3.45 (-1.29, 13.91) | -0.49 (-1.71, 0.73) | 31.73 (23.89, 40.66) | -0.6 (-0.65, -0.52) | 0.22 (-0.08, 0.89) | -0.43 (-1.79, 0.93) | 2.01 (1.48, 2.58) | -0.55 (-0.61, -0.46) |
| Republic of Singapore | 5.21 (2.26, 6.07) | -0.04 (-11.26, 7.89) | 0.97 (0.4, 1.58) | -0.8 (-0.89, -0.67) | 0.27 (0.12, 0.32) | 0.05 (-12.27, 8.63) | 0.05 (0.02, 0.08) | -0.78 (-0.88, -0.64) |
| Australia | 0.72 (-1.04, 3.54) | -0.86 (-2.01, 0.15) | 50.02 (43.95, 58.34) | -0.78 (-0.79, -0.76) | 0.04 (-0.06, 0.22) | -0.85 (-2.09, 0.24) | 3.02 (2.55, 3.57) | -0.76 (-0.78, -0.74) |
| Republic of Lithuania | 2.85 (0.37, 11.12) | 4.03 (-279.48, 2813.49) | 204.92 (176.29, 248.71) | -0.45 (-0.52, -0.39) | 0.17 (0.02, 0.67) | 4.35 (-294.41, 3003.25) | 12.37 (10.6, 15.1) | -0.42 (-0.48, -0.36) |
| Republic of Austria | 2.15 (-0.02, 9.21) | -0.31 (-1.78, 1.41) | 68.33 (58.74, 82.42) | -0.64 (-0.66, -0.62) | 0.14 (-0.0, 0.59) | -0.22 (-1.9, 1.78) | 4.35 (3.62, 5.29) | -0.59 (-0.62, -0.56) |
| New Zealand | 0.09 (-0.02, 0.53) | -0.87 (-1.47, -0.02) | 67.59 (56.36, 81.78) | -0.74 (-0.76, -0.71) | 0.01 (-0.0, 0.03) | -0.85 (-1.57, 0.02) | 4.07 (3.34, 4.97) | -0.7 (-0.73, -0.67) |
| Kingdom of Belgium | 0.36 (-0.05, 1.95) | -0.88 (-1.0, -0.83) | 41.25 (32.33, 53.84) | -0.73 (-0.77, -0.71) | 0.02 (-0.0, 0.11) | -0.88 (-1.0, -0.82) | 2.37 (1.8, 3.13) | -0.72 (-0.76, -0.7) |
| Principality of Andorra | 0.02 (-0.01, 0.14) | N/A | 42.67 (30.29, 57.71) | -0.53 (-0.69, -0.31) | 0.0 (-0.0, 0.01) | N/A | 2.56 (1.84, 3.47) | -0.49 (-0.65, -0.28) |
| Republic of Cyprus | 17.98 (-5.28, 53.75) | -0.32 (-4.09, 2.11) | 94.73 (76.74, 113.62) | -0.72 (-0.76, -0.67) | 1.15 (-0.34, 3.42) | -0.35 (-3.89, 1.94) | 6.08 (5.06, 7.26) | -0.73 (-0.77, -0.68) |
| Kingdom of Denmark | 0.1 (-0.0, 0.55) | -0.85 (-1.0, -0.22) | 36.76 (28.73, 41.53) | -0.82 (-0.87, -0.8) | 0.01 (-0.0, 0.03) | -0.84 (-1.0, -0.18) | 2.18 (1.71, 2.48) | -0.81 (-0.86, -0.79) |
| Federal Republic of Germany | 0.98 (0.0, 4.94) | -0.72 (-1.08, -0.33) | 64.16 (54.89, 78.02) | -0.69 (-0.71, -0.67) | 0.06 (0.0, 0.3) | -0.7 (-1.08, -0.28) | 3.87 (3.18, 4.71) | -0.66 (-0.69, -0.64) |
| Republic of Finland | 0.81 (-0.09, 3.84) | 1.18 (-364.35, 528.62) | 78.49 (67.83, 95.55) | -0.7 (-0.73, -0.68) | 0.05 (-0.01, 0.24) | 1.59 (-428.47, 622.22) | 4.86 (4.08, 5.99) | -0.65 (-0.68, -0.62) |
| Hellenic Republic | 8.47 (-1.52, 26.56) | 0.79 (-7.59, 12.23) | 111.88 (102.25, 123.96) | -0.47 (-0.5, -0.44) | 0.44 (-0.07, 1.39) | 0.69 (-7.26, 11.58) | 5.79 (5.13, 6.48) | -0.5 (-0.53, -0.47) |
| French Republic | 0.57 (-0.17, 2.96) | -0.78 (-1.33, -0.53) | 33.84 (27.5, 43.07) | -0.65 (-0.68, -0.62) | 0.03 (-0.01, 0.18) | -0.78 (-1.34, -0.52) | 2.0 (1.57, 2.57) | -0.64 (-0.68, -0.61) |
| Republic of Iceland | 0.0 (0.0, 0.0) | N/A | 67.83 (58.33, 82.74) | -0.71 (-0.75, -0.67) | 0.0 (0.0, 0.0) | N/A | 4.12 (3.4, 4.99) | -0.68 (-0.72, -0.64) |
| Republic of Italy | 2.54 (-0.6, 11.24) | 0.03 (-10.38, 8.0) | 54.89 (47.06, 62.7) | -0.65 (-0.67, -0.63) | 0.16 (-0.04, 0.71) | 0.14 (-11.73, 8.71) | 3.43 (2.78, 3.98) | -0.6 (-0.63, -0.58) |
| Ireland | 0.17 (-0.02, 1.01) | N/A | 49.4 (39.67, 62.67) | -0.8 (-0.83, -0.78) | 0.01 (-0.0, 0.06) | N/A | 2.96 (2.28, 3.77) | -0.78 (-0.81, -0.76) |
| Grand Duchy of Luxembourg | 0.34 (-0.02, 1.94) | -0.88 (-0.98, -0.8) | 44.47 (36.97, 53.93) | -0.73 (-0.76, -0.69) | 0.02 (-0.0, 0.12) | -0.86 (-0.98, -0.78) | 2.73 (2.23, 3.34) | -0.7 (-0.73, -0.66) |
| State of Israel | 4.07 (-7.35, 20.37) | -0.24 (-6.79, 4.69) | 28.14 (22.29, 34.51) | -0.87 (-0.89, -0.86) | 0.26 (-0.46, 1.29) | -0.16 (-7.34, 5.23) | 1.76 (1.36, 2.18) | -0.85 (-0.88, -0.84) |
| Republic of Malta | 4.25 (-6.99, 24.47) | 0.27 (-6.73, 8.32) | 82.09 (69.71, 99.03) | -0.69 (-0.73, -0.66) | 0.25 (-0.4, 1.43) | 0.33 (-7.07, 8.78) | 4.78 (3.93, 5.8) | -0.67 (-0.71, -0.64) |
| Kingdom of the Netherlands | 0.23 (-0.04, 1.44) | -0.91 (-0.98, -0.78) | 34.57 (26.06, 45.13) | -0.78 (-0.83, -0.76) | 0.01 (-0.0, 0.09) | -0.89 (-0.98, -0.73) | 2.18 (1.6, 2.88) | -0.74 (-0.8, -0.72) |
| Kingdom of Norway | 0.03 (-0.01, 0.15) | -0.77 (-6.7, 5.26) | 40.06 (35.65, 45.06) | -0.8 (-0.82, -0.79) | 0.0 (-0.0, 0.01) | -0.73 (-6.86, 6.88) | 2.48 (2.13, 2.82) | -0.77 (-0.79, -0.75) |
| Portuguese Republic | 0.21 (-0.12, 1.02) | -0.92 (-1.12, -0.76) | 52.59 (43.18, 63.1) | -0.67 (-0.71, -0.65) | 0.01 (-0.01, 0.06) | -0.92 (-1.11, -0.77) | 2.83 (2.27, 3.42) | -0.69 (-0.72, -0.66) |
| Kingdom of Spain | 1.52 (-0.6, 5.92) | -0.64 (-1.04, 0.41) | 50.49 (44.41, 56.63) | -0.65 (-0.68, -0.62) | 0.08 (-0.03, 0.33) | -0.64 (-1.04, 0.4) | 2.78 (2.35, 3.17) | -0.65 (-0.68, -0.62) |
| Kingdom of Sweden | 0.12 (-0.0, 0.64) | -0.13 (-5.93, 11.37) | 47.19 (40.87, 53.86) | -0.74 (-0.77, -0.71) | 0.01 (-0.0, 0.04) | -0.03 (-7.73, 12.7) | 3.07 (2.59, 3.5) | -0.7 (-0.74, -0.67) |
| Republic of Chile | 0.06 (-0.03, 0.39) | -0.71 (-3.48, 6.38) | 56.95 (48.0, 65.86) | -0.64 (-0.69, -0.61) | 0.0 (-0.0, 0.02) | -0.74 (-3.18, 5.49) | 2.86 (2.4, 3.32) | -0.69 (-0.72, -0.66) |
| Swiss Confederation | 0.31 (-0.02, 1.67) | -0.78 (-1.6, 0.38) | 37.97 (31.87, 44.99) | -0.76 (-0.78, -0.74) | 0.02 (-0.0, 0.11) | -0.75 (-1.7, 0.59) | 2.51 (2.02, 3.0) | -0.72 (-0.75, -0.7) |
| United Kingdom of Great Britain and Northern Ireland | 0.19 (-0.02, 1.16) | -0.89 (-1.1, -0.7) | 51.9 (40.52, 61.26) | -0.76 (-0.81, -0.74) | 0.01 (-0.0, 0.06) | -0.88 (-1.1, -0.69) | 2.86 (2.2, 3.42) | -0.75 (-0.8, -0.73) |
| Canada | 1.16 (-0.0, 4.7) | -0.63 (-0.84, 0.97) | 52.42 (46.58, 62.13) | -0.71 (-0.73, -0.7) | 0.07 (-0.0, 0.27) | -0.61 (-0.83, 1.08) | 3.04 (2.56, 3.61) | -0.7 (-0.72, -0.68) |
| Argentine Republic | 2.0 (-3.37, 9.9) | -0.64 (-1.58, 1.1) | 81.19 (73.98, 88.01) | -0.66 (-0.69, -0.64) | 0.11 (-0.18, 0.52) | -0.64 (-1.58, 1.09) | 4.28 (3.8, 4.67) | -0.66 (-0.69, -0.64) |
| Eastern Republic of Uruguay | 0.62 (-1.63, 3.52) | -0.84 (-2.03, 0.23) | 89.65 (81.54, 100.55) | -0.63 (-0.65, -0.6) | 0.03 (-0.08, 0.18) | -0.85 (-2.02, 0.21) | 4.64 (4.13, 5.23) | -0.63 (-0.66, -0.61) |
| United States of America | 10.42 (-2.76, 38.04) | -0.45 (-1.06, 0.41) | 100.66 (88.89, 119.5) | -0.57 (-0.59, -0.56) | 0.55 (-0.14, 2.03) | -0.45 (-1.09, 0.4) | 5.31 (4.43, 6.34) | -0.58 (-0.6, -0.56) |
| Antigua and Barbuda | 0.52 (0.13, 0.87) | 1.24 (-6.25, 12.1) | 3.62 (2.71, 4.79) | -0.77 (-0.8, -0.74) | 0.03 (0.01, 0.05) | 1.49 (-6.84, 13.65) | 0.21 (0.15, 0.27) | -0.74 (-0.77, -0.71) |
| Barbados | 0.25 (-0.16, 0.64) | -1.59 (-12.75, 11.68) | 1.1 (0.73, 1.61) | -0.84 (-0.88, -0.8) | 0.01 (-0.01, 0.04) | -1.61 (-12.89, 11.95) | 0.06 (0.04, 0.09) | -0.84 (-0.87, -0.8) |
| Commonwealth of the Bahamas | 4.9 (-0.41, 10.4) | -3.35 (-2.97, 1.83) | 20.43 (15.47, 26.62) | -0.4 (-0.56, -0.14) | 0.24 (-0.02, 0.5) | -3.52 (-3.13, 1.98) | 0.99 (0.76, 1.28) | -0.35 (-0.52, -0.09) |
| Belize | 0.58 (-3.33, 7.96) | -1.17 (-7.53, 2.43) | 9.49 (7.26, 12.3) | -0.74 (-0.78, -0.68) | 0.03 (-0.17, 0.41) | -1.17 (-7.82, 2.57) | 0.49 (0.37, 0.63) | -0.73 (-0.77, -0.67) |
| Republic of Cuba | 5.6 (2.28, 8.73) | -0.2 (-0.48, -0.06) | 24.94 (19.71, 31.13) | -0.53 (-0.59, -0.47) | 0.3 (0.12, 0.47) | -0.22 (-0.48, -0.09) | 1.34 (1.05, 1.67) | -0.55 (-0.6, -0.49) |
| Commonwealth of Dominica | -0.2 (-0.81, 0.21) | -0.8 (-1.53, -0.56) | 5.65 (4.25, 7.66) | -0.72 (-0.76, -0.65) | -0.01 (-0.04, 0.01) | -0.8 (-1.53, -0.57) | 0.31 (0.23, 0.41) | -0.72 (-0.76, -0.67) |
| Dominican Republic | 0.82 (-0.16, 1.79) | -1.17 (-1.63, 2.41) | 47.15 (35.57, 61.33) | -0.12 (-0.36, 0.21) | 0.04 (-0.01, 0.08) | -1.16 (-1.59, 2.08) | 2.24 (1.71, 2.95) | -0.19 (-0.41, 0.13) |
| Grenada | 0.84 (0.1, 1.5) | -4.58 (-25.92, 29.57) | 1.44 (0.99, 1.98) | -0.84 (-0.87, -0.81) | 0.04 (0.01, 0.08) | -5.03 (-28.88, 33.16) | 0.07 (0.05, 0.1) | -0.82 (-0.85, -0.79) |
| Republic of Guyana | -5.41 (-23.99, 2.04) | -0.36 (-7.78, 0.42) | 8.81 (3.65, 15.09) | -0.68 (-0.8, -0.36) | -0.26 (-1.13, 0.1) | -0.34 (-8.0, 0.44) | 0.42 (0.18, 0.71) | -0.67 (-0.8, -0.35) |
| Republic of Haiti | -8.0 (-22.81, 3.79) | -0.61 (-9.43, 6.44) | 26.64 (18.14, 39.44) | -0.38 (-0.59, 0.14) | -0.39 (-1.12, 0.18) | -0.6 (-9.68, 6.65) | 1.3 (0.89, 1.89) | -0.36 (-0.57, 0.15) |
| Jamaica | 1.02 (0.44, 1.67) | 33.78 (-260.18, 173.21) | 4.86 (3.54, 6.8) | -0.61 (-0.7, -0.49) | 0.05 (0.02, 0.09) | 32.43 (-254.35, 166.59) | 0.25 (0.19, 0.35) | -0.62 (-0.7, -0.53) |
| Saint Lucia | 0.2 (-5.43, 0.68) | -1.06 (-11.66, 4.78) | 1.13 (0.33, 1.59) | -0.87 (-0.91, -0.66) | 0.01 (-0.3, 0.04) | -1.06 (-11.01, 4.58) | 0.06 (0.02, 0.09) | -0.88 (-0.92, -0.67) |
| Saint Vincent and the Grenadines | 0.74 (0.07, 1.4) | -1.3 (-20.86, 26.61) | 1.35 (0.92, 1.9) | -0.85 (-0.88, -0.47) | 0.04 (0.0, 0.08) | -1.32 (-22.24, 28.36) | 0.08 (0.05, 0.11) | -0.84 (-0.87, -0.43) |
| Republic of Suriname | -0.38 (-15.35, 1.62) | -0.94 (-1.06, -0.33) | 3.74 (0.93, 5.58) | -0.66 (-0.8, -0.03) | -0.02 (-0.7, 0.07) | -0.94 (-1.06, -0.35) | 0.17 (0.04, 0.26) | -0.67 (-0.8, -0.05) |
| Republic of Trinidad and Tobago | -1.51 (-17.35, 0.23) | -0.29 (-1.77, 12.31) | 4.2 (0.73, 6.45) | -0.77 (-0.92, -0.7) | -0.07 (-0.84, 0.01) | -0.32 (-1.74, 11.73) | 0.2 (0.04, 0.31) | -0.78 (-0.93, -0.72) |
| Plurinational State of Bolivia | 1.84 (-3.27, 7.79) | -1.72 (-14.99, 13.93) | 77.02 (54.34, 112.5) | -0.5 (-0.61, -0.33) | 0.1 (-0.17, 0.42) | -1.78 (-16.17, 14.63) | 4.18 (3.01, 6.02) | -0.47 (-0.57, -0.3) |
| Republic of Ecuador | -1.04 (-2.52, -0.05) | -0.59 (-0.98, 0.17) | 47.74 (36.83, 61.25) | -0.33 (-0.47, -0.13) | -0.06 (-0.14, -0.0) | -0.58 (-0.98, 0.2) | 2.67 (2.09, 3.33) | -0.31 (-0.44, -0.13) |
| Republic of Peru | -0.03 (-0.37, 0.34) | -0.97 (-2.46, -0.61) | 46.98 (34.74, 61.93) | -0.45 (-0.58, -0.26) | -0.0 (-0.02, 0.02) | -0.97 (-2.49, -0.6) | 2.44 (1.81, 3.21) | -0.44 (-0.57, -0.25) |
| Republic of Colombia | 2.83 (0.77, 3.76) | -1.17 (-1.31, -1.03) | 65.66 (53.0, 83.58) | -0.53 (-0.62, -0.39) | 0.15 (0.04, 0.2) | -1.19 (-1.34, -1.03) | 3.57 (2.88, 4.55) | -0.49 (-0.58, -0.34) |
| Republic of Costa Rica | 0.01 (-0.26, 0.08) | -0.41 (-1.22, -0.38) | 41.5 (34.55, 54.23) | -0.48 (-0.55, -0.4) | 0.0 (-0.01, 0.0) | -0.48 (-1.2, -0.44) | 2.07 (1.69, 2.71) | -0.52 (-0.58, -0.45) |
| Republic of El Salvador | -4.15 (-11.72, 4.98) | -0.88 (-1.31, -0.55) | 4.56 (3.18, 7.27) | -0.56 (-0.72, -0.31) | -0.21 (-0.59, 0.25) | -0.87 (-1.33, -0.53) | 0.23 (0.16, 0.37) | -0.54 (-0.71, -0.28) |
| Republic of Guatemala | -0.4 (-2.38, 2.15) | -0.95 (-2.2, 0.42) | 62.73 (51.09, 86.22) | -0.55 (-0.63, -0.42) | -0.02 (-0.14, 0.13) | -0.94 (-2.28, 0.5) | 3.61 (2.95, 4.92) | -0.52 (-0.59, -0.38) |
| Republic of Honduras | 7.28 (-0.03, 15.66) | -2.41 (-7.44, 0.86) | 70.45 (54.87, 99.85) | 0.27 (-0.04, 1.0) | 0.4 (-0.0, 0.85) | -2.54 (-8.08, 1.09) | 3.9 (3.04, 5.54) | 0.39 (0.08, 1.18) |
| Republic of Nicaragua | 7.47 (6.0, 8.89) | -1.78 (-3.45, 0.36) | 14.16 (10.96, 18.24) | 0.11 (-0.22, 0.77) | 0.41 (0.33, 0.49) | -1.82 (-3.6, 0.44) | 0.78 (0.6, 1.01) | 0.17 (-0.16, 0.83) |
| Republic of Panama | -0.78 (-5.99, 0.04) | -0.91 (-1.01, -0.35) | 7.63 (5.53, 9.82) | -0.42 (-0.59, -0.26) | -0.04 (-0.31, 0.0) | -0.91 (-1.01, -0.39) | 0.4 (0.28, 0.51) | -0.44 (-0.6, -0.3) |
| Bolivarian Republic of Venezuela | 8.78 (-3.57, 12.17) | 2.51 (-2.1, 1.47) | 56.17 (42.4, 74.2) | -0.17 (-0.37, 0.11) | 0.43 (-0.17, 0.59) | 2.54 (-2.1, 1.48) | 2.75 (2.1, 3.57) | -0.16 (-0.35, 0.11) |
| Federative Republic of Brazil | -0.83 (-4.76, 4.3) | -0.81 (-2.71, 1.25) | 50.26 (41.45, 59.57) | -0.55 (-0.58, -0.52) | -0.03 (-0.21, 0.2) | -0.82 (-2.9, 0.9) | 2.28 (1.86, 2.73) | -0.57 (-0.59, -0.54) |
| Republic of Paraguay | 8.36 (-16.9, 40.63) | -0.26 (-1.15, 1.73) | 77.87 (54.1, 105.96) | -0.35 (-0.5, -0.17) | 0.42 (-0.84, 2.02) | -0.27 (-1.15, 1.7) | 3.9 (2.69, 5.29) | -0.36 (-0.5, -0.19) |
| People's Democratic Republic of Algeria | 61.43 (8.07, 151.0) | 0.36 (-5.12, 5.33) | 265.22 (210.32, 331.27) | -0.43 (-0.53, -0.31) | 3.85 (0.51, 9.31) | 0.48 (-5.54, 6.03) | 16.6 (13.23, 20.23) | -0.38 (-0.47, -0.26) |
| Kingdom of Bahrain | 176.32 (55.53, 313.97) | -0.55 (-0.62, -0.48) | 149.42 (98.59, 199.72) | -0.72 (-0.76, -0.67) | 10.47 (3.3, 18.56) | -0.5 (-0.57, -0.43) | 8.87 (5.9, 11.91) | -0.69 (-0.73, -0.64) |
| Arab Republic of Egypt | 200.54 (-26.59, 523.96) | 2.16 (-8.97, 13.56) | 373.72 (293.47, 476.06) | -0.22 (-0.36, 0.03) | 10.17 (-1.35, 26.42) | 2.19 (-9.02, 13.88) | 18.95 (15.0, 24.18) | -0.21 (-0.34, 0.02) |
| Islamic Republic of Iran | 71.97 (11.9, 173.73) | 0.08 (-4.88, 7.84) | 194.73 (164.92, 245.27) | -0.53 (-0.57, -0.46) | 3.84 (0.62, 9.38) | 0.13 (-5.22, 8.4) | 10.66 (8.9, 13.47) | -0.5 (-0.54, -0.42) |
| Republic of Iraq | 467.99 (86.79, 900.07) | 0.17 (-0.15, 0.63) | 456.11 (276.03, 669.94) | -0.26 (-0.45, -0.04) | 24.7 (4.59, 47.07) | 0.35 (0.03, 0.84) | 24.08 (14.78, 34.75) | -0.15 (-0.35, 0.08) |
| Hashemite Kingdom of Jordan | 14.65 (-13.82, 56.57) | 1.08 (-7.15, 7.73) | 123.34 (95.53, 157.65) | -0.63 (-0.71, -0.52) | 0.77 (-0.73, 2.99) | 1.27 (-7.7, 8.52) | 6.46 (4.98, 8.2) | -0.59 (-0.68, -0.48) |
| State of Kuwait | 271.88 (73.38, 474.14) | -0.33 (-0.45, -0.17) | 210.88 (122.05, 303.46) | -0.57 (-0.65, -0.48) | 13.43 (3.66, 23.52) | -0.3 (-0.41, -0.15) | 10.41 (6.03, 14.97) | -0.55 (-0.63, -0.46) |
| Lebanese Republic | 4.39 (-4.53, 20.58) | 0.74 (-9.95, 10.71) | 110.78 (90.8, 133.84) | -0.73 (-0.79, -0.65) | 0.24 (-0.25, 1.15) | 0.98 (-11.19, 12.51) | 6.14 (4.99, 7.43) | -0.69 (-0.75, -0.61) |
| State of Libya | 80.91 (-19.98, 241.45) | 1.42 (-12.22, 11.16) | 220.68 (165.27, 294.73) | -0.02 (-0.26, 0.36) | 3.91 (-0.97, 11.62) | 1.43 (-12.29, 11.07) | 10.67 (7.9, 14.12) | -0.01 (-0.25, 0.33) |
| Kingdom of Morocco | 37.38 (1.06, 107.67) | 0.27 (-4.52, 4.23) | 388.67 (291.47, 478.79) | -0.22 (-0.41, -0.04) | 1.95 (0.05, 5.52) | 0.39 (-4.9, 4.78) | 20.21 (15.41, 24.87) | -0.15 (-0.33, 0.03) |
| Palestine | 37.31 (-32.55, 145.05) | 2.38 (-17.15, 12.54) | 194.58 (157.96, 236.53) | -0.51 (-0.61, -0.36) | 2.08 (-1.82, 7.94) | 2.56 (-17.94, 13.26) | 10.82 (8.72, 13.08) | -0.48 (-0.58, -0.34) |
| Sultanate of Oman | 278.77 (115.34, 493.59) | -0.31 (-0.49, -0.01) | 233.29 (132.74, 335.6) | -0.61 (-0.71, -0.47) | 15.03 (6.27, 26.9) | -0.21 (-0.41, 0.1) | 12.58 (7.17, 17.96) | -0.56 (-0.66, -0.41) |
| State of Qatar | 193.11 (49.27, 373.22) | -0.65 (-0.75, -0.54) | 155.91 (87.06, 236.31) | -0.81 (-0.85, -0.75) | 11.55 (2.94, 22.12) | -0.62 (-0.72, -0.51) | 9.32 (5.22, 14.09) | -0.79 (-0.84, -0.74) |
| Kingdom of Saudi Arabia | 356.57 (100.76, 667.26) | 0.31 (-0.01, 0.95) | 271.74 (164.71, 384.72) | -0.34 (-0.51, -0.06) | 16.01 (4.55, 30.12) | 0.26 (-0.02, 0.85) | 12.19 (7.52, 17.27) | -0.37 (-0.51, -0.14) |
| Syrian Arab Republic | 115.62 (19.49, 297.84) | 0.58 (-3.84, 5.95) | 482.44 (371.55, 617.12) | -0.29 (-0.48, -0.02) | 6.17 (1.05, 15.69) | 0.78 (-4.18, 6.72) | 25.74 (20.55, 32.18) | -0.2 (-0.39, 0.06) |
| Republic of Turkey | 14.95 (-0.15, 52.05) | 0.2 (-5.08, 4.03) | 171.12 (138.07, 213.49) | -0.49 (-0.58, -0.37) | 0.85 (-0.01, 2.95) | 0.39 (-5.75, 4.7) | 9.69 (7.82, 12.0) | -0.41 (-0.51, -0.28) |
| Republic of Tunisia | 60.33 (1.31, 164.21) | 1.23 (-17.72, 10.14) | 218.56 (159.37, 304.4) | -0.32 (-0.5, -0.05) | 3.32 (0.07, 8.92) | 1.32 (-18.41, 10.17) | 12.02 (8.94, 16.39) | -0.29 (-0.46, -0.04) |
| United Arab Emirates | 314.19 (112.82, 578.57) | -0.19 (-0.39, 0.01) | 279.44 (150.87, 398.31) | -0.62 (-0.69, -0.54) | 18.52 (6.79, 34.06) | -0.02 (-0.24, 0.2) | 16.47 (8.91, 23.38) | -0.54 (-0.61, -0.46) |
| Republic of Yemen | 74.0 (31.48, 132.56) | -0.07 (-0.39, 1.36) | 238.25 (172.69, 317.9) | -0.23 (-0.44, 0.08) | 3.62 (1.54, 6.56) | 0.0 (-0.34, 1.5) | 11.67 (8.65, 15.38) | -0.17 (-0.38, 0.14) |
| People's Republic of Bangladesh | 39.53 (8.58, 73.6) | -28.41 (-42.2, 55.02) | 89.95 (61.37, 123.47) | -0.14 (-0.38, 0.32) | 1.84 (0.4, 3.44) | -33.01 (-49.63, 64.08) | 4.19 (2.87, 5.67) | -0.01 (-0.27, 0.49) |
| Islamic Republic of Afghanistan | 81.19 (-3.14, 224.05) | 0.05 (-1.68, 1.25) | 403.62 (291.63, 544.76) | -0.36 (-0.51, -0.16) | 3.71 (-0.14, 10.34) | 0.11 (-1.71, 1.38) | 18.46 (13.67, 24.57) | -0.32 (-0.47, -0.14) |
| Kingdom of Bhutan | 0.27 (-1.6, 3.49) | -23.4 (-2.98, 9.64) | 139.51 (102.8, 182.45) | -0.13 (-0.37, 0.23) | 0.01 (-0.08, 0.17) | -30.26 (-3.2, 11.12) | 6.89 (5.31, 8.75) | -0.01 (-0.26, 0.37) |
| Republic of India | 83.19 (26.76, 141.63) | 0.33 (0.11, 1.56) | 145.99 (97.35, 192.75) | -0.03 (-0.16, 0.12) | 3.7 (1.22, 6.26) | 0.42 (0.19, 1.78) | 6.46 (4.33, 8.54) | 0.02 (-0.11, 0.19) |
| Federal Democratic Republic of Nepal | 14.45 (-11.33, 46.54) | 0.86 (-4.44, 4.49) | 167.97 (132.18, 220.77) | -0.08 (-0.31, 0.26) | 0.69 (-0.54, 2.28) | 1.06 (-4.81, 5.02) | 8.07 (6.43, 10.5) | 0.02 (-0.23, 0.38) |
| Islamic Republic of Pakistan | 224.85 (35.32, 460.52) | 0.31 (0.03, 0.8) | 340.62 (213.59, 492.72) | 0.21 (-0.02, 0.58) | 10.29 (1.6, 20.97) | 0.33 (0.07, 0.78) | 15.57 (9.85, 22.46) | 0.23 (0.01, 0.56) |
| Republic of Angola | -4.63 (-14.76, 2.65) | -0.8 (-1.27, 0.12) | 60.98 (44.22, 81.67) | -0.2 (-0.44, 0.2) | -0.23 (-0.73, 0.14) | -0.79 (-1.29, 0.19) | 3.07 (2.24, 4.08) | -0.15 (-0.39, 0.26) |
| Central African Republic | -13.7 (-91.54, 25.4) | -0.56 (-6.34, 3.92) | 12.64 (5.54, 22.75) | -0.46 (-0.66, 0.06) | -0.64 (-4.34, 1.18) | -0.55 (-6.47, 3.86) | 0.59 (0.26, 1.04) | -0.45 (-0.65, 0.07) |
| Democratic Republic of the Congo | -4.88 (-17.09, 0.37) | -0.64 (-1.11, 0.67) | 37.38 (26.1, 51.03) | -0.32 (-0.52, -0.02) | -0.24 (-0.85, 0.02) | -0.63 (-1.11, 0.69) | 1.86 (1.31, 2.53) | -0.31 (-0.5, -0.03) |
| Republic of Equatorial Guinea | -7.06 (-19.09, -1.82) | -0.07 (-0.71, 1.82) | 25.2 (15.97, 37.3) | -0.56 (-0.72, -0.33) | -0.37 (-0.98, -0.1) | 0.04 (-0.66, 2.14) | 1.32 (0.84, 1.92) | -0.51 (-0.69, -0.26) |
| Republic of the Congo | -8.56 (-16.84, 1.11) | -0.16 (-1.15, 0.91) | 20.22 (12.76, 31.96) | -0.63 (-0.75, -0.42) | -0.43 (-0.83, 0.06) | -0.11 (-1.15, 0.99) | 1.01 (0.65, 1.59) | -0.61 (-0.73, -0.4) |
| Gabonese Republic | -3.98 (-10.5, -0.13) | 0.26 (-0.96, 2.21) | 19.3 (12.48, 29.31) | -0.6 (-0.71, -0.45) | -0.21 (-0.54, -0.01) | 0.31 (-0.96, 2.3) | 1.0 (0.64, 1.5) | -0.58 (-0.7, -0.42) |
| Republic of Burundi | -0.63 (-2.05, -0.06) | 0.06 (-0.89, 2.44) | 67.25 (47.25, 94.79) | -0.39 (-0.55, -0.12) | -0.03 (-0.1, -0.0) | 0.12 (-0.89, 2.58) | 3.2 (2.26, 4.45) | -0.36 (-0.52, -0.09) |
| Republic of Djibouti | 108.5 (44.33, 210.32) | 0.19 (-0.2, 0.91) | 83.11 (43.58, 134.76) | 0.4 (-0.05, 1.06) | 5.29 (2.19, 10.15) | 0.21 (-0.15, 0.93) | 4.05 (2.19, 6.46) | 0.43 (0.0, 1.03) |
| Union of the Comoros | -2.85 (-6.67, -0.35) | -0.34 (-0.93, 0.77) | 19.67 (11.76, 31.4) | -0.15 (-0.45, 0.39) | -0.14 (-0.33, -0.02) | -0.3 (-0.92, 0.84) | 0.97 (0.59, 1.53) | -0.1 (-0.41, 0.43) |
| State of Eritrea | 35.13 (10.33, 70.62) | 0.71 (-2.17, 3.08) | 62.51 (44.8, 86.9) | -0.04 (-0.27, 0.3) | 1.65 (0.49, 3.32) | 0.83 (-2.27, 3.31) | 2.93 (2.1, 3.95) | 0.02 (-0.2, 0.37) |
| United Mexican States | 10.24 (-2.04, 24.78) | 3.38 (-19.98, 13.85) | 110.57 (94.5, 133.18) | -0.07 (-0.19, 0.1) | 0.53 (-0.1, 1.27) | 3.97 (-17.41, 22.52) | 6.02 (5.14, 7.16) | -0.1 (-0.21, 0.05) |
| Federal Democratic Republic of Ethiopia | 1.05 (-0.93, 3.96) | 0.0 (-3.29, 2.38) | 58.99 (45.69, 75.77) | -0.46 (-0.64, -0.28) | 0.04 (-0.05, 0.17) | 0.82 (-4.73, 3.68) | 2.92 (2.25, 3.76) | -0.38 (-0.57, -0.2) |
| Republic of Kenya | -0.29 (-1.86, 1.28) | -0.75 (-8.71, 6.81) | 50.64 (37.23, 68.44) | 0.22 (-0.05, 0.61) | -0.01 (-0.09, 0.06) | -0.75 (-10.24, 7.11) | 2.59 (1.89, 3.48) | 0.2 (-0.06, 0.57) |
| Republic of Malawi | 4.57 (-11.75, 25.33) | -5.39 (-38.68, 26.23) | 77.2 (58.58, 99.68) | 0.36 (0.04, 0.79) | 0.21 (-0.55, 1.14) | -5.34 (-37.61, 25.3) | 3.57 (2.73, 4.6) | 0.34 (0.04, 0.74) |
| Republic of Madagascar | 1.73 (-4.96, 10.19) | -1.86 (-31.02, 21.32) | 95.84 (68.4, 129.34) | 0.08 (-0.22, 0.46) | 0.08 (-0.22, 0.47) | -1.89 (-31.69, 22.38) | 4.44 (3.21, 5.87) | 0.12 (-0.17, 0.47) |
| Republic of Mauritius | 0.39 (-3.61, 4.65) | N/A | 55.28 (42.51, 78.31) | -0.65 (-0.76, -0.61) | 0.02 (-0.17, 0.22) | N/A | 2.64 (2.03, 3.72) | -0.64 (-0.76, -0.6) |
| Republic of Mozambique | 2.42 (-7.5, 13.66) | -3.34 (-31.86, 35.86) | 28.47 (19.49, 38.28) | 0.52 (0.07, 1.08) | 0.12 (-0.37, 0.65) | -3.22 (-29.84, 34.87) | 1.37 (0.96, 1.86) | 0.44 (0.03, 0.96) |
| Republic of Rwanda | -0.03 (-0.16, 0.13) | N/A | 62.64 (43.3, 92.78) | -0.51 (-0.65, -0.29) | -0.0 (-0.01, 0.01) | N/A | 3.21 (2.18, 4.86) | -0.45 (-0.6, -0.23) |
| Federal Republic of Somalia | -17.04 (-54.34, 5.41) | 0.16 (-8.48, 1.64) | 12.05 (7.56, 18.24) | -0.05 (-0.35, 0.38) | -0.77 (-2.47, 0.24) | 0.17 (-8.63, 1.76) | 0.55 (0.35, 0.81) | -0.03 (-0.33, 0.36) |
| United Republic of Tanzania | -0.91 (-4.15, 2.74) | -0.62 (-4.35, 1.66) | 56.01 (39.06, 76.52) | 0.23 (-0.15, 0.72) | -0.04 (-0.19, 0.13) | -0.62 (-4.44, 1.7) | 2.69 (1.9, 3.59) | 0.27 (-0.11, 0.75) |
| Republic of Seychelles | 1.69 (1.01, 2.24) | -0.13 (-0.3, 0.21) | 11.76 (9.22, 15.06) | -0.54 (-0.6, -0.48) | 0.08 (0.05, 0.11) | -0.04 (-0.22, 0.34) | 0.58 (0.45, 0.74) | -0.49 (-0.55, -0.43) |
| Republic of Uganda | -1.42 (-4.07, 0.38) | 0.55 (-1.9, 4.99) | 32.91 (23.87, 44.56) | -0.34 (-0.53, -0.06) | -0.07 (-0.2, 0.02) | 0.55 (-1.88, 4.95) | 1.61 (1.18, 2.25) | -0.34 (-0.51, -0.08) |
| Kingdom of Lesotho | 0.04 (-0.09, 0.43) | N/A | 117.6 (61.88, 189.46) | 0.82 (0.23, 1.75) | 0.0 (-0.0, 0.02) | N/A | 5.57 (3.02, 8.77) | 0.66 (0.15, 1.45) |
| Republic of Zambia | 3.8 (-13.28, 25.84) | -44.36 (-17.44, 27.53) | 81.6 (55.25, 110.83) | 0.53 (0.08, 1.1) | 0.19 (-0.63, 1.25) | -46.88 (-17.54, 27.47) | 3.98 (2.76, 5.33) | 0.56 (0.11, 1.1) |
| Republic of Namibia | 4.15 (-16.74, 29.9) | 0.6 (-1.05, 1.28) | 96.87 (70.39, 128.91) | -0.01 (-0.25, 0.3) | 0.21 (-0.85, 1.5) | 0.63 (-1.05, 1.26) | 4.87 (3.59, 6.42) | 0.01 (-0.22, 0.3) |
| Republic of Botswana | 3.57 (-11.2, 22.39) | -0.39 (-1.04, 0.16) | 84.61 (59.99, 111.3) | -0.19 (-0.42, 0.14) | 0.18 (-0.58, 1.12) | -0.37 (-1.04, 0.21) | 4.34 (3.13, 5.6) | -0.16 (-0.37, 0.15) |
| Republic of South Africa | 0.17 (-2.53, 3.51) | 3.16 (-1.5, 1.51) | 107.1 (95.01, 123.24) | -0.12 (-0.22, 0.01) | 0.01 (-0.13, 0.18) | 2.02 (-1.33, 1.96) | 5.47 (4.8, 6.27) | -0.03 (-0.14, 0.14) |
| Kingdom of Eswatini | 0.23 (-6.98, 8.93) | -1.53 (-7.8, 8.41) | 147.23 (101.46, 213.37) | 0.19 (-0.18, 0.68) | 0.01 (-0.33, 0.42) | -1.49 (-7.23, 7.92) | 6.84 (4.87, 9.61) | 0.12 (-0.2, 0.55) |
| Republic of Benin | 21.05 (-42.51, 47.88) | -2.92 (-2.69, 1.71) | 4.46 (1.59, 7.13) | -0.6 (-0.75, 0.19) | 1.11 (-2.21, 2.47) | -3.0 (-2.76, 1.75) | 0.23 (0.08, 0.38) | -0.58 (-0.74, 0.22) |
| Burkina Faso | 71.85 (32.42, 138.58) | 1.06 (-2.03, 1.73) | 13.49 (7.15, 22.9) | -0.57 (-0.71, -0.23) | 3.8 (1.81, 7.23) | 1.14 (-2.08, 1.78) | 0.71 (0.38, 1.21) | -0.56 (-0.7, -0.18) |
| Republic of Zimbabwe | 3.83 (-13.99, 25.69) | 10.4 (-7.16, 10.42) | 120.26 (85.69, 159.93) | 0.57 (0.16, 1.23) | 0.19 (-0.71, 1.28) | 9.08 (-6.4, 9.57) | 6.04 (4.42, 7.96) | 0.41 (0.08, 0.95) |
| Republic of Chad | 72.95 (13.4, 168.88) | 0.36 (-0.29, 1.53) | 32.73 (17.79, 54.27) | -0.08 (-0.31, 0.22) | 3.65 (0.67, 8.36) | 0.36 (-0.29, 1.47) | 1.64 (0.9, 2.7) | -0.08 (-0.3, 0.2) |
| Republic of Côte d'Ivoire | 2.32 (-32.75, 13.68) | -1.13 (-1.63, 0.6) | 7.24 (2.82, 11.68) | -0.6 (-0.76, 0.61) | 0.12 (-1.68, 0.7) | -1.14 (-1.61, 0.62) | 0.37 (0.14, 0.59) | -0.6 (-0.76, 0.61) |
| Republic of Ghana | 28.34 (9.11, 45.15) | 0.55 (-1.65, 4.25) | 5.43 (2.48, 8.02) | -0.83 (-0.89, -0.75) | 1.45 (0.46, 2.3) | 0.62 (-1.68, 4.49) | 0.28 (0.13, 0.41) | -0.82 (-0.89, -0.74) |
| Republic of Cameroon | 21.15 (6.78, 47.09) | 1.09 (0.15, 3.26) | 34.07 (23.99, 50.0) | -0.08 (-0.32, 0.33) | 1.09 (0.36, 2.42) | 1.1 (0.2, 3.21) | 1.75 (1.24, 2.54) | -0.07 (-0.3, 0.31) |
| Republic of Cabo Verde | -0.42 (-2.58, 2.03) | N/A | 82.19 (60.32, 109.83) | 0.66 (0.26, 1.26) | -0.02 (-0.14, 0.11) | N/A | 4.55 (3.43, 6.1) | 0.83 (0.43, 1.47) |
| Republic of Liberia | -5.46 (-13.27, 0.23) | -0.0 (-1.06, 3.4) | 9.84 (5.3, 16.9) | -0.44 (-0.67, -0.02) | -0.28 (-0.67, 0.01) | -0.0 (-1.06, 3.41) | 0.51 (0.28, 0.86) | -0.44 (-0.66, -0.03) |
| Republic of the Gambia | 43.04 (14.68, 68.2) | 2.63 (-4.52, 5.99) | 21.91 (13.51, 30.0) | -0.3 (-0.54, 0.03) | 2.21 (0.75, 3.5) | 2.71 (-4.6, 6.02) | 1.12 (0.7, 1.54) | -0.29 (-0.52, 0.04) |
| Republic of Guinea | 12.09 (-15.35, 28.13) | -18.21 (-3.26, 1.54) | 15.48 (10.01, 22.3) | -0.29 (-0.51, 0.19) | 0.62 (-0.78, 1.44) | -18.24 (-3.29, 1.54) | 0.79 (0.51, 1.12) | -0.28 (-0.5, 0.21) |
| Republic of Guinea-Bissau | 12.26 (-47.29, 82.83) | -1.7 (-6.91, 4.21) | 8.56 (4.25, 19.88) | -0.64 (-0.77, -0.19) | 0.59 (-2.34, 3.9) | -1.72 (-7.19, 4.24) | 0.42 (0.21, 0.94) | -0.62 (-0.76, -0.17) |
| Democratic Republic of Sao Tome and Principe | -0.82 (-1.26, -0.38) | -0.54 (-10.86, 20.12) | 5.69 (4.21, 7.57) | -0.56 (-0.68, -0.23) | -0.04 (-0.07, -0.02) | -0.54 (-11.1, 20.48) | 0.3 (0.22, 0.39) | -0.56 (-0.67, -0.25) |
| Republic of the Niger | 80.45 (32.31, 152.1) | 0.45 (-8.16, 4.78) | 43.95 (22.72, 68.23) | -0.04 (-0.31, 0.5) | 4.24 (1.76, 8.05) | 0.49 (-8.27, 4.84) | 2.32 (1.22, 3.59) | -0.01 (-0.29, 0.51) |
| Republic of Senegal | 86.8 (41.0, 141.13) | 0.39 (-26.38, 18.11) | 21.55 (14.55, 29.96) | -0.55 (-0.67, -0.35) | 4.59 (2.17, 7.33) | 0.47 (-28.06, 18.8) | 1.14 (0.77, 1.59) | -0.53 (-0.65, -0.32) |
| Republic of Mali | 71.48 (28.92, 143.41) | 0.26 (-0.18, 0.78) | 12.84 (7.21, 22.02) | -0.7 (-0.77, -0.56) | 3.65 (1.53, 7.21) | 0.29 (-0.15, 0.83) | 0.66 (0.37, 1.11) | -0.69 (-0.76, -0.55) |
| Republic of Sierra Leone | 3.53 (-9.48, 12.48) | -3.73 (-3.78, 6.56) | 12.11 (7.03, 18.53) | -0.45 (-0.63, -0.19) | 0.18 (-0.48, 0.63) | -3.71 (-3.77, 6.55) | 0.61 (0.36, 0.93) | -0.45 (-0.62, -0.21) |
| Islamic Republic of Mauritania | 180.94 (67.57, 335.45) | -0.02 (-0.29, 0.39) | 41.69 (24.03, 61.69) | -0.61 (-0.71, -0.46) | 9.76 (3.69, 17.95) | 0.06 (-0.24, 0.49) | 2.25 (1.32, 3.29) | -0.58 (-0.68, -0.42) |
| Federal Republic of Nigeria | 27.06 (0.94, 52.07) | 0.77 (-4.93, 6.77) | 18.69 (11.0, 28.03) | -0.4 (-0.54, -0.17) | 1.44 (0.11, 2.73) | 0.85 (-5.94, 6.47) | 0.98 (0.59, 1.44) | -0.39 (-0.52, -0.19) |
| Togolese Republic | 32.98 (-17.35, 63.36) | -3.44 (-9.94, 2.62) | 6.58 (2.92, 10.82) | -0.66 (-0.79, -0.31) | 1.67 (-0.89, 3.18) | -3.45 (-10.09, 2.57) | 0.33 (0.15, 0.54) | -0.65 (-0.78, -0.31) |
| Cook Islands | -7.39 (-11.99, -3.12) | -0.25 (-0.45, 0.78) | 0.87 (0.49, 1.35) | -0.04 (-0.36, 0.51) | -0.34 (-0.56, -0.15) | -0.23 (-0.43, 0.82) | 0.04 (0.02, 0.06) | -0.01 (-0.33, 0.54) |
| American Samoa | -2.29 (-3.66, -1.17) | -0.7 (-0.94, 0.53) | 15.13 (10.65, 20.86) | 0.23 (-0.15, 4.53) | -0.1 (-0.16, -0.05) | -0.7 (-0.94, 0.51) | 0.68 (0.48, 0.93) | 0.23 (-0.13, 4.55) |
| Greenland | 1.16 (-1.59, 5.63) | -1.33 (-3.68, 3.49) | 120.43 (77.05, 169.38) | -0.63 (-0.71, -0.36) | 0.06 (-0.08, 0.29) | -1.34 (-3.74, 3.67) | 6.1 (3.9, 8.52) | -0.62 (-0.7, -0.34) |
| Bermuda | 1.5 (-4.39, 10.67) | -0.39 (-1.79, 1.85) | 40.88 (33.12, 52.31) | -0.76 (-0.8, -0.71) | 0.08 (-0.24, 0.6) | -0.36 (-1.83, 2.0) | 2.29 (1.86, 2.95) | -0.75 (-0.79, -0.7) |
| Guam | 4.37 (-3.61, 20.42) | -1.53 (-4.79, 4.07) | 0.34 (0.04, 1.77) | -0.6 (-0.91, 0.96) | 0.16 (-0.14, 0.78) | -1.39 (-3.78, 2.73) | 0.01 (0.0, 0.07) | -0.71 (-0.93, 0.45) |
| Republic of Nauru | 22.14 (4.68, 41.45) | -10.27 (-57.98, 30.38) | 1.04 (0.69, 1.4) | -0.7 (-0.77, -0.6) | 0.9 (0.19, 1.68) | -10.32 (-59.17, 31.55) | 0.04 (0.03, 0.06) | -0.69 (-0.76, -0.6) |
| Republic of Niue | -1.93 (-2.9, 1.49) | -0.32 (-7.93, 9.17) | 59.86 (44.22, 78.64) | -0.25 (-0.42, -0.03) | -0.09 (-0.13, 0.07) | -0.3 (-8.27, 9.18) | 2.79 (2.13, 3.64) | -0.23 (-0.37, -0.05) |
| Principality of Monaco | 0.45 (-0.21, 2.9) | -0.24 (-2.45, 3.54) | 66.03 (52.41, 82.96) | -0.57 (-0.67, -0.42) | 0.03 (-0.01, 0.17) | -0.2 (-2.62, 3.76) | 3.79 (3.01, 4.67) | -0.54 (-0.64, -0.41) |
| Saint Kitts and Nevis | 0.66 (0.17, 1.15) | 0.52 (-6.58, 7.38) | 4.8 (3.47, 6.5) | -0.82 (-0.85, -0.79) | 0.04 (0.01, 0.06) | 0.61 (-6.98, 8.07) | 0.26 (0.19, 0.35) | -0.81 (-0.84, -0.78) |
| Northern Mariana Islands | 4.06 (2.63, 5.59) | -1.14 (-1.91, -1.05) | 20.16 (16.11, 24.82) | -0.26 (-0.43, 0.01) | 0.19 (0.12, 0.26) | -1.15 (-1.92, -1.05) | 0.93 (0.73, 1.16) | -0.24 (-0.4, 0.04) |
| Republic of Palau | 1.68 (-5.68, 14.38) | -1.19 (-3.44, 1.73) | 2.82 (1.95, 4.27) | -0.51 (-0.65, 0.02) | 0.08 (-0.25, 0.66) | -1.19 (-3.56, 1.81) | 0.13 (0.09, 0.19) | -0.5 (-0.63, 0.01) |
| Tokelau | -2.8 (-12.92, 14.44) | 0.87 (-2.44, 1.67) | 1.93 (1.32, 4.07) | -0.23 (-0.42, 0.12) | -0.13 (-0.6, 0.67) | 0.86 (-2.44, 1.64) | 0.09 (0.06, 0.19) | -0.23 (-0.41, 0.07) |
| Puerto Rico | 1.31 (0.8, 1.71) | 0.11 (-0.34, 0.56) | 2.38 (1.7, 3.26) | -0.85 (-0.88, -0.82) | 0.07 (0.04, 0.08) | 0.01 (-0.39, 0.41) | 0.12 (0.09, 0.16) | -0.87 (-0.89, -0.84) |
| Republic of San Marino | 1.4 (-0.44, 6.87) | -0.18 (-5.47, 5.01) | 31.63 (20.29, 44.9) | -0.7 (-0.8, -0.56) | 0.08 (-0.02, 0.4) | -0.18 (-5.55, 4.9) | 1.88 (1.25, 2.63) | -0.7 (-0.79, -0.59) |
| Tuvalu | -1.65 (-12.02, 9.91) | -20.06 (-1.47, 1.27) | 3.03 (2.27, 3.92) | -0.44 (-0.68, -0.16) | -0.07 (-0.53, 0.44) | -23.26 (-1.48, 1.27) | 0.13 (0.1, 0.17) | -0.43 (-0.67, -0.14) |
| Republic of South Sudan | 59.29 (25.28, 110.35) | 1.3 (-0.32, 3.62) | 17.85 (9.85, 28.93) | -0.19 (-0.49, 0.26) | 2.77 (1.23, 5.05) | 1.31 (-0.32, 3.49) | 0.83 (0.47, 1.31) | -0.18 (-0.48, 0.21) |
| United States Virgin Islands | 0.78 (0.21, 1.33) | -1.25 (-8.62, 9.0) | 5.47 (3.73, 7.66) | -0.77 (-0.83, -0.48) | 0.04 (0.01, 0.07) | -1.25 (-8.76, 9.19) | 0.3 (0.2, 0.41) | -0.76 (-0.82, -0.47) |
| Republic of Sudan | 460.02 (225.44, 815.73) | -0.2 (-0.41, 0.18) | 173.96 (100.69, 263.88) | -0.42 (-0.56, -0.21) | 22.36 (11.07, 39.11) | -0.14 (-0.34, 0.24) | 8.45 (4.97, 12.69) | -0.38 (-0.52, -0.17) |
| DALYs: Disability-adjusted life years. ASDR: Age-Standardized Death Rate. ASMR: Age-Standardized Mortality Rate. TPC: Total Percent Change. | | | | | | | | |

| **Table S3. Age-specific DALYs and ASDR for myocardial disease attributable to high temperature, categorized by 5-year age groups, presented globally and by SDI region in 2021.** | | | | | | | | | | | | | | | | | | | |
| --- | --- | --- | --- | --- | --- | --- | --- | --- | --- | --- | --- | --- | --- | --- | --- | --- | --- | --- | --- |
|  |  | Global | | | High SDI | | | High-middle SDI | | | Low SDI | | | Low-middle SDI | | | Middle SDI | | |
| age | metric | Both | Female | Male | Both | Female | Male | Both | Female | Male | Both | Female | Male | Both | Female | Male | Both | Female | Male |
| <5 years | Number95%(UI) | 22676.34(-6759.39,56899.75) | 11268.16(-2676.61,27904.58) | 11408.18(-4035.89,29553.37) | 591.48(-36.45,1254.09) | 240.45(-14.81,524.49) | 351.03(-21.64,765.62) | 761.35(-107.76,1657.32) | 293.82(-49.7,645.17) | 467.53(-62.67,1048.92) | 3069.11(-5082.14,13610.33) | 1581.55(-2265.08,6629.31) | 1487.56(-2832.71,7176.64) | 13642.74(-2379.02,32360.37) | 7069.23(-851.25,16953.19) | 6573.51(-1247.72,16308.15) | 4610.51(40.86,9158.05) | 2083.53(39.66,3982.93) | 2526.99(-3.88,5230.61) |
|  | Rate95%(UI) | 3.45(-1.03,8.65) | 3.54(-0.84,8.77) | 3.36(-1.19,8.69) | 1.1(-0.07,2.33) | 0.92(-0.06,2) | 1.27(-0.08,2.77) | 1.09(-0.15,2.37) | 0.88(-0.15,1.94) | 1.27(-0.17,2.86) | 1.85(-3.07,8.22) | 1.95(-2.8,8.19) | 1.76(-3.35,8.48) | 7.12(-1.24,16.89) | 7.61(-0.92,18.26) | 6.66(-1.26,16.52) | 2.61(0.02,5.19) | 2.46(0.05,4.71) | 2.75(0,5.68) |
| 5-9 years | Number95%(UI) | 2819.33(-1144.74,6980.34) | 1357.38(-285.02,3352.77) | 1461.95(-825.49,4174.7) | 124.87(-8.33,273.38) | 58.56(-4.52,128.43) | 66.31(-4.11,148.8) | 187.45(-26.17,419.69) | 72.96(-12.04,162.72) | 114.48(-15.83,263.11) | 278.78(-694.84,1605.26) | 125.68(-177.37,550.94) | 153.1(-523.87,1123.75) | 1445.26(-405.78,3683.47) | 740.81(-127.66,1770.91) | 704.46(-281.18,1930.66) | 782.39(-37.28,1667.3) | 359.17(-1.28,750) | 423.22(-36.03,933.25) |
|  | Rate95%(UI) | 0.41(-0.17,1.02) | 0.41(-0.09,1.01) | 0.41(-0.23,1.18) | 0.21(-0.01,0.47) | 0.2(-0.02,0.45) | 0.22(-0.01,0.49) | 0.23(-0.03,0.51) | 0.19(-0.03,0.41) | 0.27(-0.04,0.61) | 0.18(-0.45,1.05) | 0.17(-0.24,0.73) | 0.2(-0.67,1.44) | 0.74(-0.21,1.89) | 0.78(-0.13,1.87) | 0.7(-0.28,1.92) | 0.4(-0.02,0.85) | 0.38(0,0.79) | 0.41(-0.04,0.91) |
| 10-14 years | Number95%(UI) | 2741.46(-742.41,6335.03) | 1315.73(-393.79,3321.37) | 1425.73(-371.2,3419.47) | 151.09(-10.64,334.14) | 67.16(-5.38,148.74) | 83.94(-5.48,186.44) | 187.77(-40.27,441.27) | 75.37(-14.54,179.62) | 112.4(-25.13,269.48) | 288.58(-332,1139.5) | 165.97(-212.23,706.6) | 122.61(-123.08,478.02) | 1364.31(-300.89,3239.06) | 700.22(-144.16,1776.74) | 664.09(-167.68,1670.65) | 749.27(-75.4,1604.65) | 306.93(-36.18,680.01) | 442.34(-35.39,952.79) |
|  | Rate95%(UI) | 0.41(-0.11,0.95) | 0.41(-0.12,1.03) | 0.41(-0.11,0.99) | 0.25(-0.02,0.56) | 0.23(-0.02,0.51) | 0.27(-0.02,0.61) | 0.24(-0.05,0.56) | 0.2(-0.04,0.48) | 0.27(-0.06,0.66) | 0.2(-0.24,0.81) | 0.24(-0.31,1.02) | 0.17(-0.17,0.67) | 0.71(-0.16,1.67) | 0.75(-0.15,1.89) | 0.67(-0.17,1.68) | 0.39(-0.04,0.83) | 0.33(-0.04,0.73) | 0.44(-0.04,0.95) |
| 15-19 years | Number95%(UI) | 3232.83(-1275.8,8198.01) | 1255.78(-243.39,2971.54) | 1977.05(-1029.22,5539.96) | 265.18(-22.25,587.37) | 77.74(-5.7,174.2) | 187.45(-16.89,415.95) | 248.43(-73.02,588.48) | 74.6(-23.92,183.53) | 173.83(-50.71,423.73) | 382.73(-582.95,1674.68) | 161.68(-77.35,478.13) | 221.05(-500.61,1220.69) | 1492.17(-483.57,3800.94) | 655.82(-85.05,1659.87) | 836.36(-387.6,2282.05) | 844.19(-153.32,1822.62) | 286.03(-38.27,595.26) | 558.16(-121.21,1263.07) |
|  | Rate95%(UI) | 0.52(-0.2,1.31) | 0.41(-0.08,0.98) | 0.62(-0.32,1.73) | 0.44(-0.04,0.98) | 0.27(-0.02,0.6) | 0.6(-0.05,1.34) | 0.34(-0.1,0.81) | 0.22(-0.07,0.53) | 0.46(-0.13,1.11) | 0.31(-0.47,1.35) | 0.26(-0.13,0.78) | 0.35(-0.8,1.96) | 0.81(-0.26,2.06) | 0.73(-0.09,1.84) | 0.89(-0.41,2.42) | 0.46(-0.08,1) | 0.33(-0.04,0.68) | 0.59(-0.13,1.34) |
| 20-24 years | Number95%(UI) | 4162.2(-1572.43,10330.45) | 1646.91(-550.32,4028.22) | 2515.29(-975.33,6352.55) | 437.73(-39.58,1018.6) | 130.03(-9.73,293.12) | 307.7(-29.85,723.33) | 362.94(-167.82,924.69) | 103.79(-40.88,252.89) | 259.15(-126.89,672.02) | 477.06(-556.57,1781.01) | 226.92(-220.94,852.62) | 250.14(-314.45,990.53) | 1918.28(-561.56,4576.55) | 850.81(-185.96,2272.55) | 1067.47(-365.63,2718.02) | 966.26(-258.63,2220.53) | 335.56(-86.96,775.35) | 630.69(-168.81,1461.44) |
|  | Rate95%(UI) | 0.7(-0.26,1.73) | 0.56(-0.19,1.37) | 0.83(-0.32,2.09) | 0.67(-0.06,1.56) | 0.41(-0.03,0.93) | 0.91(-0.09,2.13) | 0.48(-0.22,1.23) | 0.29(-0.11,0.71) | 0.66(-0.32,1.71) | 0.46(-0.53,1.71) | 0.43(-0.42,1.62) | 0.49(-0.61,1.92) | 1.1(-0.32,2.62) | 0.98(-0.21,2.61) | 1.22(-0.42,3.1) | 0.55(-0.15,1.25) | 0.39(-0.1,0.89) | 0.7(-0.19,1.61) |
| 25-29 years | Number95%(UI) | 4898.28(-2618.19,13229.64) | 1795.6(-844.34,4861.09) | 3102.68(-1753.91,8651.37) | 593.17(-48.15,1288.91) | 180.43(-11.91,413.17) | 412.74(-36.24,906.74) | 527.2(-400.36,1456.37) | 127.7(-83.34,345.63) | 399.5(-314.55,1143.75) | 534.55(-883.83,2453.85) | 223.5(-350.26,1027.79) | 311.05(-551.25,1470.45) | 2174.76(-860.95,5639.26) | 892.67(-310.92,2474.45) | 1282.1(-540.72,3501.73) | 1068.97(-396.61,2643.9) | 371.59(-125.95,903.54) | 697.38(-283.08,1778.65) |
|  | Rate95%(UI) | 0.83(-0.45,2.25) | 0.62(-0.29,1.67) | 1.04(-0.59,2.91) | 0.83(-0.07,1.81) | 0.52(-0.03,1.2) | 1.12(-0.1,2.46) | 0.62(-0.47,1.72) | 0.32(-0.21,0.86) | 0.9(-0.71,2.58) | 0.62(-1.03,2.85) | 0.51(-0.8,2.33) | 0.74(-1.31,3.49) | 1.34(-0.53,3.48) | 1.1(-0.38,3.05) | 1.59(-0.67,4.34) | 0.58(-0.22,1.44) | 0.41(-0.14,1) | 0.75(-0.3,1.91) |
| 30-34 years | Number95%(UI) | 6592.4(-3260.9,16332.58) | 1930.91(-805.41,5015.94) | 4661.49(-2409.54,11775.42) | 747.51(-70.42,1654.31) | 232.05(-19.87,519.56) | 515.45(-51.84,1206.16) | 1043.7(-1010.27,3246.32) | 220.16(-208.13,687.63) | 823.54(-802.24,2608.85) | 665.27(-650.93,2288.49) | 228.39(-188.85,786.78) | 436.88(-474.77,1656.09) | 2649.17(-874.82,6298.16) | 837.48(-236.52,2330.86) | 1811.69(-630.78,4468.86) | 1486.94(-504.24,3336.24) | 412.94(-149.58,961.45) | 1074(-371.08,2483.71) |
|  | Rate95%(UI) | 1.09(-0.54,2.7) | 0.65(-0.27,1.68) | 1.53(-0.79,3.85) | 0.96(-0.09,2.13) | 0.62(-0.05,1.39) | 1.28(-0.13,3) | 0.98(-0.95,3.04) | 0.43(-0.4,1.34) | 1.49(-1.45,4.73) | 0.92(-0.9,3.16) | 0.62(-0.51,2.12) | 1.24(-1.35,4.69) | 1.79(-0.59,4.26) | 1.13(-0.32,3.15) | 2.45(-0.85,6.05) | 0.75(-0.25,1.67) | 0.42(-0.15,0.97) | 1.07(-0.37,2.47) |
| 35-39 years | Number95%(UI) | 8018.52(-4059.89,20410.82) | 2301.6(-1043.3,5648.59) | 5716.92(-2917.34,14921.68) | 1019.84(-91.4,2358.45) | 303.3(-18.35,699.12) | 716.54(-73.64,1708) | 1486.59(-1648.9,4877.75) | 316.49(-346.3,1036.45) | 1170.11(-1306.12,3820.94) | 743.84(-550.9,2367.56) | 249.68(-197.14,839.23) | 494.16(-361.95,1533.26) | 2869.67(-973.95,6944.93) | 905.76(-284.58,2399.25) | 1963.91(-673.17,4971.44) | 1898.51(-521.38,4219.08) | 526.49(-137.08,1178.97) | 1372.03(-396.24,3095.52) |
|  | Rate95%(UI) | 1.43(-0.72,3.64) | 0.83(-0.38,2.03) | 2.02(-1.03,5.27) | 1.3(-0.12,3) | 0.8(-0.05,1.84) | 1.76(-0.18,4.19) | 1.47(-1.63,4.81) | 0.64(-0.7,2.09) | 2.26(-2.52,7.36) | 1.19(-0.88,3.8) | 0.78(-0.62,2.63) | 1.63(-1.19,5.04) | 2.15(-0.73,5.2) | 1.36(-0.43,3.6) | 2.94(-1.01,7.44) | 1.03(-0.28,2.29) | 0.57(-0.15,1.29) | 1.48(-0.43,3.33) |
| 40-44 years | Number95%(UI) | 10118.27(-5065.63,26150.47) | 2506.32(-1211.06,6227.5) | 7611.95(-3735.53,19145.76) | 1294.85(-123.41,2898.05) | 360.82(-26.22,824.67) | 934.04(-98.75,2136.91) | 1805.28(-1977.62,5887.26) | 373.81(-425.35,1242.98) | 1431.47(-1534.24,4664.17) | 1024.57(-762.57,3202.68) | 243.28(-227.74,839.15) | 781.29(-529.37,2427.38) | 3748.53(-1145.02,9209.18) | 906.98(-336.32,2428.68) | 2841.55(-810.93,7241.23) | 2244.14(-602.06,5244.87) | 621.33(-139.46,1338.69) | 1622.81(-463.8,3790.18) |
|  | Rate95%(UI) | 2.02(-1.01,5.23) | 1.01(-0.49,2.51) | 3.02(-1.48,7.59) | 1.71(-0.16,3.83) | 0.98(-0.07,2.25) | 2.4(-0.25,5.49) | 1.95(-2.14,6.37) | 0.82(-0.93,2.73) | 3.05(-3.27,9.95) | 1.98(-1.48,6.2) | 0.93(-0.87,3.21) | 3.06(-2.08,9.52) | 3.25(-0.99,7.99) | 1.57(-0.58,4.21) | 4.93(-1.41,12.57) | 1.36(-0.37,3.18) | 0.76(-0.17,1.64) | 1.95(-0.56,4.56) |
| 45-49 years | Number95%(UI) | 11054.88(-5343.98,28424.6) | 2943.71(-1263.98,7179.24) | 8111.16(-3991.31,21315.74) | 1453.8(-124.53,3154.26) | 391.52(-25.66,827.66) | 1062.28(-98.87,2332.92) | 1899.45(-1977.5,6241.98) | 402.05(-428.49,1338.97) | 1497.39(-1539.36,4913.4) | 1033.45(-808.72,3312.32) | 286.67(-212.45,890.31) | 746.78(-580.03,2519.02) | 4033.71(-1381.76,10413.18) | 1100.83(-348.1,2891.32) | 2932.88(-1054.46,7503.16) | 2632.94(-649,5631.06) | 762.49(-164.14,1699.67) | 1870.44(-467.1,4171.19) |
|  | Rate95%(UI) | 2.33(-1.13,6) | 1.25(-0.54,3.05) | 3.41(-1.68,8.96) | 1.98(-0.17,4.3) | 1.09(-0.07,2.31) | 2.84(-0.26,6.23) | 1.96(-2.04,6.44) | 0.83(-0.89,2.78) | 3.08(-3.16,10.09) | 2.48(-1.94,7.94) | 1.38(-1.02,4.29) | 3.57(-2.77,12.04) | 4.1(-1.4,10.57) | 2.23(-0.7,5.85) | 5.98(-2.15,15.3) | 1.62(-0.4,3.46) | 0.94(-0.2,2.1) | 2.29(-0.57,5.12) |
| 50-54 years | Number95%(UI) | 11815.68(-5066.91,29820.91) | 3024.52(-1146.33,7117.3) | 8791.16(-3843.38,22810.01) | 1574.48(-163.76,3508.63) | 433.17(-35.62,940.59) | 1141.31(-129.76,2569.75) | 2031.06(-1940.71,6421.17) | 430.3(-412.67,1383.88) | 1600.75(-1511.51,5013.02) | 1013.16(-697.62,3058.49) | 241.73(-172.92,768.12) | 771.44(-541.82,2392.46) | 4214.63(-1482.47,10970.32) | 1113.02(-343.7,2913.1) | 3101.6(-1085.6,8115.89) | 2979.94(-600.02,6624.42) | 805.96(-131.91,1809.5) | 2173.97(-446.14,4927.79) |
|  | Rate95%(UI) | 2.66(-1.14,6.7) | 1.36(-0.51,3.19) | 3.96(-1.73,10.28) | 2.12(-0.22,4.72) | 1.18(-0.1,2.56) | 3.05(-0.35,6.86) | 2.1(-2,6.63) | 0.89(-0.85,2.85) | 3.31(-3.12,10.35) | 3.1(-2.13,9.35) | 1.49(-1.06,4.72) | 4.69(-3.3,14.55) | 5.02(-1.77,13.07) | 2.63(-0.81,6.89) | 7.45(-2.61,19.49) | 1.9(-0.38,4.23) | 1.02(-0.17,2.29) | 2.79(-0.57,6.33) |
| 55-59 years | Number95%(UI) | 13648.32(-6898.49,34167.35) | 4147.79(-1712.93,10021.02) | 9500.54(-5042.58,24100.52) | 1700.18(-216.45,3725.68) | 481.96(-45.93,1047.83) | 1218.21(-171.01,2712.85) | 2188.96(-1905.64,6699.59) | 565.71(-481.23,1766.89) | 1623.24(-1400.22,4907.49) | 1216.88(-1536.82,4479.23) | 426.52(-324.62,1431.17) | 790.36(-1199.39,3191.75) | 5043.71(-2234.53,12652.62) | 1536.93(-598.78,4139.2) | 3506.78(-1590.67,9087.1) | 3496.06(-718.32,7579.06) | 1136.29(-219.34,2430.48) | 2359.77(-514.56,5232.12) |
|  | Rate95%(UI) | 3.45(-1.74,8.63) | 2.06(-0.85,4.99) | 4.88(-2.59,12.38) | 2.34(-0.3,5.12) | 1.32(-0.13,2.87) | 3.36(-0.47,7.49) | 2.43(-2.12,7.45) | 1.23(-1.05,3.85) | 3.68(-3.18,11.14) | 4.8(-6.07,17.68) | 3.35(-2.55,11.24) | 6.27(-9.52,25.34) | 7.26(-3.22,18.22) | 4.35(-1.7,11.72) | 10.27(-4.66,26.61) | 2.53(-0.52,5.49) | 1.61(-0.31,3.45) | 3.49(-0.76,7.75) |
| 60-64 years | Number95%(UI) | 12569.73(-5847.07,31478.52) | 3979.93(-1595.43,9974.68) | 8589.8(-4177.09,22158.72) | 1662.02(-231.1,3790.93) | 489.6(-67.7,1103.87) | 1172.42(-163.83,2685.02) | 1913.26(-1681.14,5864.88) | 515.49(-418.69,1558.11) | 1397.77(-1261.36,4318.13) | 1218.46(-1099.86,4175.82) | 433.17(-310.48,1372.38) | 785.29(-799.45,2893.3) | 4718.89(-1930.64,12142.16) | 1524.11(-539.42,4052.33) | 3194.78(-1385.13,8609.86) | 3054.13(-752.72,6752.08) | 1017(-235.24,2211.44) | 2037.13(-539.29,4641.59) |
|  | Rate95%(UI) | 3.93(-1.83,9.84) | 2.42(-0.97,6.06) | 5.52(-2.69,14.25) | 2.44(-0.34,5.56) | 1.41(-0.19,3.17) | 3.52(-0.49,8.06) | 2.62(-2.31,8.04) | 1.36(-1.1,4.1) | 4(-3.61,12.36) | 6.18(-5.58,21.18) | 4.34(-3.11,13.76) | 8.06(-8.21,29.7) | 8.25(-3.38,21.24) | 5.18(-1.83,13.78) | 11.51(-4.99,31.01) | 3(-0.74,6.63) | 1.95(-0.45,4.24) | 4.11(-1.09,9.35) |
| 65-69 years | Number95%(UI) | 11666.23(-4690.83,28591.02) | 3974.55(-1329.35,9393.67) | 7691.69(-3271.86,19211.49) | 1589.84(-243.45,3624.73) | 474.01(-66.88,1080.75) | 1115.82(-177.04,2549.79) | 1616.09(-958.24,4483.39) | 506.59(-310.96,1424.31) | 1109.5(-653.04,3102.73) | 1146.11(-899.47,3660.83) | 418.48(-172.22,1141.03) | 727.63(-704.21,2623.81) | 4378.2(-1667.71,11440.9) | 1506.52(-441.28,3928.34) | 2871.68(-1188.11,7706.83) | 2932.72(-750.73,6555.95) | 1068.1(-248.37,2414.82) | 1864.62(-487.27,4343.42) |
|  | Rate95%(UI) | 4.23(-1.7,10.36) | 2.76(-0.92,6.52) | 5.83(-2.48,14.57) | 2.62(-0.4,5.98) | 1.5(-0.21,3.42) | 3.84(-0.61,8.78) | 2.43(-1.44,6.75) | 1.43(-0.88,4.02) | 3.59(-2.11,10.03) | 7.6(-5.97,24.28) | 5.45(-2.24,14.85) | 9.84(-9.52,35.49) | 9.87(-3.76,25.79) | 6.55(-1.92,17.07) | 13.45(-5.57,36.1) | 3.29(-0.84,7.35) | 2.31(-0.54,5.23) | 4.34(-1.13,10.1) |
| 70-74 years | Number95%(UI) | 9851.89(-3487.04,24021.4) | 3752.91(-1205.93,8861.56) | 6098.98(-2241.75,15040.72) | 1480.11(-240.7,3312.19) | 502.04(-78.92,1128.35) | 978.07(-163.72,2192.07) | 1260.74(-535.8,3276.7) | 468.44(-205.87,1268.71) | 792.31(-340.61,2033.03) | 1123.41(-631.15,3337.44) | 460.77(-198.54,1284.84) | 662.64(-429.53,2087.57) | 3678.58(-1452.15,9427.16) | 1413.48(-490.27,3685.29) | 2265.1(-932.54,5904.15) | 2305.89(-609.37,5014.85) | 907.03(-232.61,2028.25) | 1398.87(-366.46,3134.18) |
|  | Rate95%(UI) | 4.79(-1.69,11.67) | 3.43(-1.1,8.1) | 6.33(-2.33,15.6) | 2.77(-0.45,6.21) | 1.77(-0.28,3.98) | 3.91(-0.65,8.76) | 2.58(-1.1,6.71) | 1.74(-0.77,4.72) | 3.61(-1.55,9.26) | 10.83(-6.09,32.18) | 8.68(-3.74,24.2) | 13.09(-8.49,41.24) | 11.7(-4.62,29.98) | 8.58(-2.98,22.37) | 15.13(-6.23,39.44) | 3.74(-0.99,8.14) | 2.8(-0.72,6.27) | 4.78(-1.25,10.7) |
| 75-79 years | Number95%(UI) | 7812.68(-2762.8,18864.97) | 3228.59(-1123.16,7939.41) | 4584.1(-1598.24,11575.91) | 1132.99(-182.11,2581.82) | 423.84(-70.31,973.08) | 709.15(-112.15,1639.46) | 1023.1(-302.4,2493.84) | 427.81(-151.15,1116.29) | 595.29(-160.96,1419.22) | 816.32(-557.73,2587.34) | 368.32(-214.09,1194.49) | 448(-343.25,1486.1) | 2903.35(-1174.89,7407.78) | 1206.76(-442.54,3145.31) | 1696.59(-695.97,4566.87) | 1934.16(-437.7,4299.3) | 800.62(-200.56,1870.99) | 1133.55(-255.3,2591.38) |
|  | Rate95%(UI) | 5.92(-2.09,14.3) | 4.48(-1.56,11.01) | 7.67(-2.67,19.36) | 3.13(-0.5,7.13) | 2.13(-0.35,4.89) | 4.34(-0.69,10.04) | 3.43(-1.01,8.35) | 2.53(-0.89,6.6) | 4.59(-1.24,10.95) | 12.71(-8.69,40.3) | 11.08(-6.44,35.92) | 14.47(-11.09,48) | 14.46(-5.85,36.89) | 11.22(-4.12,29.26) | 18.19(-7.46,48.96) | 4.94(-1.12,10.97) | 3.78(-0.95,8.84) | 6.29(-1.42,14.38) |
| 80-84 years | Number95%(UI) | 6484.05(-2086.28,15607.7) | 2987.47(-1027.67,7213.97) | 3496.58(-1048.84,8782.21) | 949.32(-187.04,2162.65) | 394.91(-85.05,923.43) | 554.41(-104.79,1276.45) | 1087.67(-441.41,2939.52) | 577.78(-289.14,1654.1) | 509.89(-161.83,1292.95) | 583.31(-295.94,1678.73) | 271.28(-112.95,773.14) | 312.03(-181,1021.74) | 2153.73(-825.8,5447.78) | 923.27(-353.18,2468.61) | 1230.46(-458.85,3329.92) | 1707(-398.81,3960.18) | 818.61(-221.73,1978.43) | 888.4(-183.94,2025.45) |
|  | Rate95%(UI) | 7.4(-2.38,17.82) | 5.87(-2.02,14.16) | 9.54(-2.86,23.96) | 3.52(-0.69,8.02) | 2.53(-0.54,5.9) | 4.89(-0.92,11.26) | 4.98(-2.02,13.46) | 4.31(-2.15,12.33) | 6.06(-1.92,15.37) | 17.29(-8.77,49.77) | 14.94(-6.22,42.57) | 20.04(-11.62,65.62) | 18.73(-7.18,47.37) | 14.34(-5.49,38.34) | 24.31(-9.07,65.79) | 7.17(-1.67,16.62) | 6.03(-1.63,14.58) | 8.67(-1.79,19.76) |
| 85-89 years | Number95%(UI) | 4506.51(-1302.53,10898.95) | 2194.1(-659.47,5180.6) | 2312.41(-682.04,5515.64) | 787.71(-164.76,1832.66) | 387.71(-83.52,935.01) | 400(-84.34,925.95) | 910.28(-285.15,2352.15) | 499.8(-201.98,1365.6) | 410.48(-88.19,1003.46) | 275.09(-173.2,852.67) | 131.12(-43.32,378.8) | 143.97(-128.04,521.5) | 1193.37(-440.26,3134.89) | 518.62(-194.93,1476.06) | 674.75(-246.28,1835.28) | 1337.85(-272.96,2930.07) | 655.54(-159.09,1529.4) | 682.31(-119.78,1508.85) |
|  | Rate95%(UI) | 9.86(-2.85,23.84) | 7.71(-2.32,18.2) | 13.4(-3.95,31.97) | 4.77(-1,11.11) | 3.76(-0.81,9.06) | 6.48(-1.37,15) | 8(-2.51,20.67) | 6.71(-2.71,18.34) | 10.45(-2.24,25.53) | 20.61(-12.97,63.87) | 17.62(-5.82,50.9) | 24.37(-21.67,88.27) | 23.88(-8.81,62.73) | 18.08(-6.8,51.45) | 31.7(-11.57,86.23) | 11.67(-2.38,25.55) | 9.29(-2.26,21.68) | 15.46(-2.71,34.19) |
| 90-94 years | Number95%(UI) | 2364.72(-690.72,5788.36) | 1294.75(-433.72,3270.84) | 1069.97(-257.65,2540.93) | 562.77(-120.35,1345.37) | 327.62(-67.84,800.73) | 235.15(-52.65,550.78) | 542.34(-152.65,1358.94) | 328.13(-121.54,883.98) | 214.21(-34.31,496.82) | 73.25(-63.38,260.83) | 35.58(-31.97,135.01) | 37.68(-30.69,132.76) | 502.16(-198.43,1291.84) | 217.65(-118.35,634.11) | 284.51(-85.12,757.23) | 683.15(-182.32,1666.21) | 385.12(-125.08,984.31) | 298.03(-57.24,674.16) |
|  | Rate95%(UI) | 13.22(-3.86,32.36) | 10.74(-3.6,27.12) | 18.36(-4.42,43.59) | 7.34(-1.57,17.55) | 6.24(-1.29,15.24) | 9.74(-2.18,22.82) | 12.41(-3.49,31.08) | 10.59(-3.92,28.54) | 16.81(-2.69,39) | 20.44(-17.68,72.76) | 17.44(-15.68,66.2) | 24.38(-19.87,85.92) | 31.03(-12.26,79.83) | 22.98(-12.49,66.94) | 42.4(-12.69,112.86) | 17.72(-4.73,43.22) | 15.13(-4.91,38.66) | 22.76(-4.37,51.48) |
| 95+ years | Number95%(UI) | 995.01(-267.11,2438.38) | 674.43(-197.75,1667.99) | 320.58(-71.93,781.57) | 311.5(-63.77,771.35) | 212.54(-42.55,529.96) | 98.95(-21.22,235.3) | 212.69(-61.27,545.96) | 160.71(-46.45,415.91) | 51.98(-16.53,138.13) | 16.82(-15.44,60.31) | 9.47(-11.53,38.86) | 7.35(-4.19,23.38) | 167.17(-62.17,434.06) | 71.09(-43.64,234.92) | 96.08(-20.57,256.15) | 286.41(-83.95,739.76) | 220.37(-65.98,570.93) | 66.04(-19.46,166.33) |
|  | Rate95%(UI) | 18.26(-4.9,44.74) | 17.13(-5.02,42.35) | 21.2(-4.76,51.69) | 11.24(-2.3,27.84) | 10.31(-2.06,25.71) | 13.95(-2.99,33.18) | 19.27(-5.55,49.46) | 19.43(-5.61,50.27) | 18.8(-5.98,49.95) | 22.11(-20.3,79.27) | 19.52(-23.78,80.13) | 26.66(-15.18,84.77) | 36.86(-13.71,95.72) | 24.5(-15.04,80.96) | 58.83(-12.59,156.84) | 27.53(-8.07,71.11) | 31.18(-9.33,80.77) | 19.8(-5.83,49.87) |
| DALYs: Disability-adjusted life years. ASDR: age-standardized DALY rate. SDI: Sociodemographic Index. | | | | | | | | | | | | | | | | | | | |

| **Table S4. Age-specific DALYs and ASDR for myocardial disease attributable to low temperature, categorized by 5-year age groups, presented globally and by SDI region in 2021.** | | | | | | | | | | | | | | | | | | | |
| --- | --- | --- | --- | --- | --- | --- | --- | --- | --- | --- | --- | --- | --- | --- | --- | --- | --- | --- | --- |
|  |  | Global | | | High SDI | | | High-middle SDI | | | Low SDI | | | Low-middle SDI | | | Middle SDI | | |
| age | metric | Both | Female | Male | Both | Female | Male | Both | Female | Male | Both | Female | Male | Both | Female | Male | Both | Female | Male |
| <5 years | Number95%(UI) | 34443.86(19068.26,55858.4) | 16446.63(8466.72,27765.26) | 17997.23(10043.55,29630.61) | 1900.31(1505.7,2336.03) | 845.95(667.72,1013.1) | 1054.35(820.43,1325.93) | 2288.82(1511.99,2997.32) | 942.36(617.02,1233.31) | 1346.46(889.15,1813.2) | 8236.84(3847.4,14075.79) | 4078.06(1692.07,7316.48) | 4158.78(1806.19,7047.3) | 14698.98(4794.52,27375.28) | 7474.87(2310.49,14524.63) | 7224.11(2566.72,13123.99) | 7300.97(4858.48,10592.92) | 3095.62(2046.65,4520.64) | 4205.35(2722.98,6179.92) |
|  | Rate95%(UI) | 5.23(2.9,8.49) | 5.17(2.66,8.73) | 5.29(2.95,8.72) | 3.53(2.8,4.34) | 3.23(2.55,3.86) | 3.82(2.97,4.8) | 3.27(2.16,4.28) | 2.83(1.85,3.7) | 3.67(2.42,4.94) | 4.97(2.32,8.5) | 5.04(2.09,9.04) | 4.92(2.13,8.33) | 7.67(2.5,14.29) | 8.05(2.49,15.64) | 7.32(2.6,13.29) | 4.13(2.75,6) | 3.66(2.42,5.34) | 4.57(2.96,6.72) |
| 5-9 years | Number95%(UI) | 4793.27(3107.44,7341.31) | 2112.79(1284.37,3316.9) | 2680.48(1732.1,4023.02) | 382.98(305.89,456.9) | 183.45(143.44,220.88) | 199.53(158.01,243) | 546.79(330.63,708.98) | 221.37(142.62,295.93) | 325.41(189.21,444.91) | 814.05(379.33,1472.73) | 319.66(133.59,608.93) | 494.39(200.63,934.33) | 1515.8(619.69,2835.56) | 732.76(242.25,1449.87) | 783.05(305.26,1541.17) | 1531.5(1077.84,2141.07) | 654.55(436.32,942.92) | 876.94(587.92,1237.61) |
|  | Rate95%(UI) | 0.7(0.45,1.07) | 0.64(0.39,1) | 0.76(0.49,1.13) | 0.65(0.52,0.78) | 0.64(0.5,0.77) | 0.66(0.52,0.81) | 0.66(0.4,0.86) | 0.56(0.36,0.75) | 0.75(0.44,1.03) | 0.53(0.25,0.96) | 0.42(0.18,0.81) | 0.63(0.26,1.2) | 0.78(0.32,1.46) | 0.77(0.26,1.53) | 0.78(0.3,1.54) | 0.78(0.55,1.09) | 0.69(0.46,1) | 0.86(0.57,1.21) |
| 10-14 years | Number95%(UI) | 4902.09(3219.39,7085.25) | 2270.98(1362.09,3553.92) | 2631.1(1750.62,3795.19) | 470.55(377.91,553.45) | 198.44(158.22,237.53) | 272.11(213.34,326.82) | 675.41(453.55,854.71) | 257.08(163.61,334.96) | 418.33(274.45,539.02) | 695.06(344.82,1204.39) | 383.34(164.18,746.74) | 311.72(145.59,571.46) | 1503.94(581.22,2622.58) | 793.43(265.4,1580.46) | 710.51(282.42,1321.91) | 1554.51(1100,2140.4) | 637.43(433.34,910.89) | 917.09(632.26,1276.83) |
|  | Rate95%(UI) | 0.74(0.48,1.06) | 0.7(0.42,1.1) | 0.77(0.51,1.1) | 0.78(0.63,0.92) | 0.68(0.54,0.81) | 0.88(0.69,1.06) | 0.86(0.58,1.09) | 0.69(0.44,0.9) | 1.02(0.67,1.31) | 0.49(0.24,0.85) | 0.55(0.24,1.07) | 0.44(0.2,0.8) | 0.78(0.3,1.36) | 0.84(0.28,1.68) | 0.71(0.28,1.33) | 0.8(0.57,1.11) | 0.69(0.47,0.98) | 0.91(0.63,1.27) |
| 15-19 years | Number95%(UI) | 7105.26(4841.99,10348.21) | 2335.5(1446.59,3611.53) | 4769.76(3152.18,7029.26) | 979.91(792.17,1156.23) | 261.35(205.52,311.28) | 718.55(580.31,852.6) | 1263.36(904.18,1593.13) | 356.73(242.37,450.54) | 906.64(651.8,1162.99) | 965.4(471.95,1741.69) | 331.26(144.11,590.25) | 634.14(285.49,1187.7) | 2014.65(802.31,3615.91) | 795.59(281.91,1610.71) | 1219.06(469.11,2266) | 1877.85(1298.07,2578.77) | 589.34(401.54,814.57) | 1288.51(874.87,1805.4) |
|  | Rate95%(UI) | 1.14(0.78,1.66) | 0.77(0.48,1.19) | 1.49(0.98,2.19) | 1.63(1.32,1.92) | 0.9(0.71,1.07) | 2.31(1.87,2.74) | 1.74(1.25,2.2) | 1.04(0.7,1.31) | 2.38(1.71,3.06) | 0.78(0.38,1.4) | 0.54(0.23,0.96) | 1.02(0.46,1.91) | 1.09(0.43,1.96) | 0.88(0.31,1.78) | 1.29(0.5,2.41) | 1.03(0.71,1.41) | 0.67(0.46,0.93) | 1.36(0.93,1.91) |
| 20-24 years | Number95%(UI) | 9811.48(6834.13,13877.88) | 3285.92(2055.96,5009.91) | 6525.56(4658.58,8986.14) | 1504.3(1194.17,1789.9) | 406.28(320.33,490.94) | 1098.02(870.73,1301.53) | 2243.17(1607.68,2763.86) | 563.8(393.06,720.29) | 1679.36(1178.89,2114.73) | 1081.5(526.99,1937.04) | 451.82(186.54,848.58) | 629.68(303.96,1182.98) | 2602.82(907.98,4705.48) | 1074.18(346.11,2271.05) | 1528.64(580.34,2822.98) | 2374.33(1598.12,3332.72) | 788.37(510.92,1111.49) | 1585.96(1038.01,2262.17) |
|  | Rate95%(UI) | 1.64(1.14,2.32) | 1.12(0.7,1.71) | 2.15(1.54,2.96) | 2.3(1.83,2.74) | 1.29(1.02,1.56) | 3.24(2.57,3.84) | 2.99(2.14,3.69) | 1.58(1.1,2.02) | 4.26(2.99,5.37) | 1.04(0.51,1.86) | 0.86(0.35,1.61) | 1.22(0.59,2.29) | 1.49(0.52,2.69) | 1.23(0.4,2.61) | 1.74(0.66,3.22) | 1.34(0.9,1.88) | 0.91(0.59,1.28) | 1.75(1.15,2.5) |
| 25-29 years | Number95%(UI) | 14433.12(10422.92,19920.72) | 4244.78(2720.61,6270.94) | 10188.34(7529.9,14026.87) | 2055.54(1643.25,2429.35) | 532.61(417.47,646.22) | 1522.92(1221.47,1816.5) | 4947.98(3709.23,6077.56) | 1045.43(770.35,1334.96) | 3902.55(2876.68,4869.23) | 1333.31(616.97,2448.29) | 491.76(196.17,908.55) | 841.55(399.28,1615.4) | 3174.75(1136.51,5627.66) | 1241.38(378.72,2500.02) | 1933.37(710.48,3626.7) | 2913.56(1963.69,4140.14) | 931.43(606.94,1330.24) | 1982.13(1307,2847.89) |
|  | Rate95%(UI) | 2.45(1.77,3.39) | 1.46(0.93,2.16) | 3.43(2.53,4.72) | 2.88(2.3,3.4) | 1.54(1.21,1.87) | 4.14(3.32,4.93) | 5.84(4.38,7.18) | 2.59(1.91,3.31) | 8.79(6.48,10.97) | 1.55(0.72,2.84) | 1.12(0.45,2.06) | 2(0.95,3.84) | 1.96(0.7,3.48) | 1.53(0.47,3.08) | 2.4(0.88,4.5) | 1.59(1.07,2.25) | 1.03(0.67,1.47) | 2.13(1.4,3.06) |
| 30-34 years | Number95%(UI) | 24697.56(18529.33,32057.29) | 6038.9(4240.55,8357.11) | 18658.67(13910.81,24139.48) | 2908.56(2318.79,3416.56) | 758.97(598.46,920.19) | 2149.59(1715.78,2530.6) | 12350.51(9139.3,15196.03) | 2565.66(1878.22,3310.8) | 9784.85(7206.69,12119.51) | 1511.51(737.3,2685.01) | 439.06(176.82,836.53) | 1072.44(503.16,2073.37) | 3982(1536.24,6840.12) | 1196.88(404.06,2364) | 2785.12(1065.1,4883.6) | 3932.48(2586.15,5518.17) | 1076.13(694.97,1537.01) | 2856.35(1862.39,4094.72) |
|  | Rate95%(UI) | 4.09(3.07,5.3) | 2.02(1.42,2.8) | 6.11(4.55,7.9) | 3.75(2.99,4.4) | 2.03(1.6,2.46) | 5.35(4.27,6.3) | 11.58(8.57,14.24) | 4.98(3.65,6.43) | 17.72(13.05,21.95) | 2.09(1.02,3.71) | 1.18(0.48,2.25) | 3.04(1.43,5.88) | 2.69(1.04,4.63) | 1.62(0.55,3.2) | 3.77(1.44,6.61) | 1.97(1.3,2.77) | 1.09(0.7,1.56) | 2.84(1.85,4.06) |
| 35-39 years | Number95%(UI) | 35084.46(25718.36,45746.97) | 8292.07(5982.63,11193) | 26792.39(19863.76,34521.13) | 3925.92(3158.27,4562.34) | 991.7(782.95,1194) | 2934.22(2356.44,3386.09) | 20201.68(15059.17,25212.7) | 4110.07(2994.86,5398.9) | 16091.61(12094.99,20210) | 1700.98(846.57,2956.4) | 523.34(214.21,930.31) | 1177.63(586.39,2170.26) | 4451.41(1719.41,7871.09) | 1385.72(465.1,2637.35) | 3065.69(1189.87,5445.32) | 4782.59(3070.88,6617.34) | 1277.66(816.48,1846.23) | 3504.93(2250.83,4859.25) |
|  | Rate95%(UI) | 6.26(4.59,8.16) | 2.98(2.15,4.03) | 9.47(7.02,12.2) | 4.99(4.02,5.8) | 2.61(2.06,3.15) | 7.2(5.79,8.31) | 19.92(14.85,24.86) | 8.29(6.04,10.9) | 31.01(23.31,38.95) | 2.73(1.36,4.75) | 1.64(0.67,2.92) | 3.87(1.93,7.14) | 3.34(1.29,5.9) | 2.08(0.7,3.96) | 4.59(1.78,8.15) | 2.59(1.66,3.58) | 1.39(0.89,2.01) | 3.77(2.42,5.23) |
| 40-44 years | Number95%(UI) | 43855.96(32204.19,57249.84) | 9839.27(7122.88,13358.21) | 34016.69(25141.44,44235.84) | 5294.19(4259.19,6164.68) | 1198.03(935.52,1462.7) | 4096.16(3314.26,4736.83) | 24793.93(18647.22,31008.07) | 5209.89(3825.47,6604.13) | 19584.04(14774.33,24831.05) | 2275.57(1094.42,4174.77) | 532.3(223.84,995.61) | 1743.27(829.06,3373.11) | 5945.92(2247.56,10281.39) | 1465.42(457.08,2765.25) | 4480.5(1625.13,8131.08) | 5507.55(3626.37,7728.92) | 1428.21(908.21,2042.52) | 4079.34(2651.06,5707.56) |
|  | Rate95%(UI) | 8.77(6.44,11.44) | 3.97(2.87,5.38) | 13.49(9.97,17.54) | 7(5.63,8.15) | 3.26(2.55,3.98) | 10.53(8.52,12.17) | 26.83(20.18,33.56) | 11.45(8.4,14.51) | 41.77(31.51,52.96) | 4.41(2.12,8.09) | 2.04(0.86,3.81) | 6.84(3.25,13.23) | 5.16(1.95,8.92) | 2.54(0.79,4.79) | 7.78(2.82,14.12) | 3.34(2.2,4.69) | 1.74(1.11,2.5) | 4.91(3.19,6.87) |
| 45-49 years | Number95%(UI) | 47629.53(35228.42,61011.61) | 11134.33(8080.34,15117.86) | 36495.2(27012.29,46398.86) | 6445.1(5216.64,7421.3) | 1435.04(1124.3,1690.6) | 5010.07(4071.22,5770.74) | 25942.82(19424.31,31892.44) | 5585(4150.09,7316.46) | 20357.83(15215.23,25177.33) | 2331.67(1148.56,4113.94) | 619.19(255.89,1108.88) | 1712.48(811.68,3256.99) | 6465.13(2411.14,11682.78) | 1726.09(566.32,3299.08) | 4739.04(1751.57,8779.3) | 6389.74(4127.16,8986.49) | 1761.31(1129.59,2543.74) | 4628.43(2943.79,6536.31) |
|  | Rate95%(UI) | 10.06(7.44,12.89) | 4.73(3.43,6.42) | 15.34(11.36,19.51) | 8.78(7.11,10.12) | 4(3.13,4.71) | 13.37(10.87,15.41) | 26.77(20.04,32.91) | 11.58(8.61,15.17) | 41.82(31.26,51.72) | 5.59(2.75,9.87) | 2.98(1.23,5.34) | 8.18(3.88,15.56) | 6.56(2.45,11.86) | 3.49(1.15,6.67) | 9.66(3.57,17.9) | 3.93(2.54,5.52) | 2.17(1.39,3.14) | 5.68(3.61,8.02) |
| 50-54 years | Number95%(UI) | 50845.52(38411.52,65493.81) | 11729.4(8734.28,15267.15) | 39116.12(29398.98,50648.1) | 8394.62(6588.98,9623.59) | 1912.24(1510.41,2283.27) | 6482.38(5085.33,7502.55) | 26041.86(19407.86,32363.16) | 5546.46(4129.3,7416.95) | 20495.4(15168.24,25515.39) | 2237.04(1082.22,4129.34) | 555.22(241.04,1033.74) | 1681.82(779.51,3261.73) | 6775.12(2610.69,12389.24) | 1759.28(568.37,3323.16) | 5015.84(1890.77,9333.02) | 7321(4811.09,10380.05) | 1945.2(1315.18,2736.37) | 5375.8(3522.68,7707.56) |
|  | Rate95%(UI) | 11.43(8.63,14.72) | 5.26(3.92,6.85) | 17.62(13.24,22.82) | 11.3(8.87,12.96) | 5.2(4.1,6.2) | 17.31(13.58,20.03) | 26.88(20.03,33.4) | 11.44(8.52,15.3) | 42.33(31.33,52.7) | 6.84(3.31,12.62) | 3.41(1.48,6.35) | 10.23(4.74,19.84) | 8.07(3.11,14.76) | 4.16(1.34,7.86) | 12.04(4.54,22.41) | 4.67(3.07,6.62) | 2.46(1.67,3.47) | 6.91(4.53,9.9) |
| 55-59 years | Number95%(UI) | 56466.05(42937.34,72800.14) | 15242.5(11338.2,20349.08) | 41223.55(31197.77,52925.29) | 10640.72(8370.64,11958.16) | 2465.86(1921.24,2881.14) | 8174.86(6430.16,9239.5) | 25790.89(19663.89,32056.45) | 6586.62(4950.44,8992.48) | 19204.28(14525.05,24003.55) | 2825.97(1252.76,4905.69) | 902.79(329.39,1694.01) | 1923.18(845.16,3489.67) | 8290.82(2887.78,14370) | 2497.31(826.37,4899.93) | 5793.51(2041.67,10486.92) | 8832.92(5742.37,12406.41) | 2775.6(1792.76,3965.88) | 6057.32(3859.98,8676.17) |
|  | Rate95%(UI) | 14.27(10.85,18.4) | 7.58(5.64,10.12) | 21.17(16.02,27.18) | 14.63(11.51,16.44) | 6.75(5.26,7.89) | 22.57(17.75,25.5) | 28.68(21.86,35.64) | 14.36(10.79,19.6) | 43.58(32.96,54.48) | 11.16(4.95,19.37) | 7.09(2.59,13.3) | 15.27(6.71,27.7) | 11.94(4.16,20.69) | 7.07(2.34,13.88) | 16.96(5.98,30.7) | 6.4(4.16,8.99) | 3.94(2.55,5.63) | 8.97(5.72,12.85) |
| 60-64 years | Number95%(UI) | 53054.97(40606.03,67434.62) | 14807.81(11060.96,19672.7) | 38247.16(29192.19,48841.91) | 11746.75(9266.66,13324.59) | 2949.84(2276.59,3404.41) | 8796.92(6937.86,10046.22) | 23267.84(17563.09,29166.75) | 5977.07(4498.56,8149.04) | 17290.78(12955.2,21817.69) | 2567(1116.1,4770.79) | 890.11(320.46,1715.47) | 1676.89(695.96,3308.61) | 7639.82(2626.9,13562.99) | 2420.54(756.33,4527.54) | 5219.28(1867.7,9368.79) | 7735.94(5086.03,10873.71) | 2550.02(1658.06,3600.1) | 5185.92(3344.85,7383.78) |
|  | Rate95%(UI) | 16.58(12.69,21.07) | 9(6.72,11.96) | 24.59(18.77,31.4) | 17.23(13.59,19.55) | 8.47(6.54,9.77) | 26.39(20.81,30.14) | 31.9(24.08,39.99) | 15.74(11.84,21.45) | 49.48(37.07,62.43) | 13.02(5.66,24.2) | 8.92(3.21,17.2) | 17.21(7.14,33.97) | 13.36(4.6,23.73) | 8.23(2.57,15.4) | 18.8(6.73,33.75) | 7.6(5,10.68) | 4.89(3.18,6.9) | 10.45(6.74,14.88) |
| 65-69 years | Number95%(UI) | 45226.03(35101.84,57770.18) | 14253.87(10817.18,18724.1) | 30972.15(23753.5,39110.97) | 12134.07(9721.69,13566.18) | 3315.54(2616.69,3780.82) | 8818.53(7032.51,9911.19) | 16308.21(12564.89,19770.65) | 5071.52(3784.35,6378.06) | 11236.69(8638.24,13508.23) | 2309.34(988.78,4333.32) | 811.53(301.04,1535.59) | 1497.82(610.07,2941.95) | 6963.83(2406.92,12455.32) | 2336.54(729.48,4408.31) | 4627.29(1632.26,8393.52) | 7406.51(4973.71,10486.7) | 2690.68(1770.78,3899.24) | 4715.83(3140.52,6631.39) |
|  | Rate95%(UI) | 16.4(12.73,20.94) | 9.9(7.51,13) | 23.49(18.02,29.67) | 20.01(16.04,22.38) | 10.5(8.29,11.98) | 30.35(24.21,34.11) | 24.56(18.92,29.77) | 14.3(10.67,17.98) | 36.32(27.92,43.66) | 15.32(6.56,28.74) | 10.56(3.92,19.99) | 20.26(8.25,39.79) | 15.7(5.43,28.08) | 10.15(3.17,19.16) | 21.68(7.65,39.32) | 8.31(5.58,11.76) | 5.83(3.84,8.45) | 10.97(7.31,15.43) |
| 70-74 years | Number95%(UI) | 38039.38(29598.27,48158.36) | 13706.13(10432.89,17857.2) | 24333.25(18879.02,30563.07) | 12393.91(9701.92,14062.24) | 3900.02(3037.7,4587.31) | 8493.88(6522.59,9590.94) | 11650.46(8586.03,14020.54) | 4327.71(3091.07,5451.32) | 7322.76(5416.01,8889.43) | 2100.86(808.17,3976.41) | 854.23(287.58,1660.06) | 1246.63(465.27,2539.23) | 5878.49(1876.06,10450.46) | 2234.3(692.5,4294.32) | 3644.19(1212.73,6659.22) | 5920.31(3944.52,8259.85) | 2356.08(1522.5,3360.45) | 3564.23(2318.81,5067.41) |
|  | Rate95%(UI) | 18.48(14.38,23.4) | 12.52(9.53,16.32) | 25.24(19.59,31.71) | 23.23(18.18,26.35) | 13.76(10.72,16.19) | 33.94(26.06,38.32) | 23.85(17.58,28.7) | 16.1(11.5,20.28) | 33.34(24.66,40.47) | 20.26(7.79,38.34) | 16.09(5.42,31.27) | 24.63(9.19,50.16) | 18.69(5.97,33.23) | 13.56(4.2,26.06) | 24.34(8.1,44.48) | 9.61(6.4,13.4) | 7.28(4.71,10.39) | 12.17(7.92,17.31) |
| 75-79 years | Number95%(UI) | 30442.32(23830.16,38050.87) | 12638.52(9807.32,16072.92) | 17803.8(13901.97,22325.05) | 10582.75(8296.9,11956.13) | 3917.35(3047.17,4498.19) | 6665.39(5259.8,7509.15) | 8720.23(6361,10317.49) | 3943.18(2936.96,4696.55) | 4777.05(3329.26,5774.18) | 1554.42(583.42,2895.1) | 695.07(229.32,1394.66) | 859.36(335.73,1706.93) | 4653.56(1538.38,8289.41) | 1959.28(642.31,3639.93) | 2694.29(839.19,5044.68) | 4852.46(3120.81,6839.48) | 2088.78(1297.64,2994.68) | 2763.68(1766.75,4002.33) |
|  | Rate95%(UI) | 23.08(18.07,28.85) | 17.53(13.6,22.29) | 29.78(23.25,37.34) | 29.22(22.91,33.02) | 19.7(15.33,22.62) | 40.81(32.21,45.98) | 29.2(21.3,34.54) | 23.33(17.37,27.78) | 36.85(25.68,44.54) | 24.21(9.09,45.09) | 20.9(6.9,41.94) | 27.76(10.84,55.14) | 23.18(7.66,41.28) | 18.22(5.97,33.86) | 28.88(9,54.08) | 12.38(7.97,17.46) | 9.87(6.13,14.15) | 15.34(9.81,22.22) |
| 80-84 years | Number95%(UI) | 31254.61(24644.92,37967.56) | 15829.4(12199.79,19222.32) | 15425.21(12353.77,18782.81) | 11085.32(8399.25,12692.54) | 4986.6(3722.86,5846.44) | 6098.73(4741.85,6980.6) | 11269.77(8607.78,13188.87) | 6604.1(5017.32,7819.74) | 4665.67(3450.97,5547.36) | 1080.58(406.48,2061.98) | 506.21(178.5,1024.74) | 574.37(212.03,1251.95) | 3460.97(1181.56,6286.91) | 1546.52(512.78,2931.38) | 1914.45(663.16,3673.41) | 4269.35(2741.53,6023.31) | 2136.75(1348.79,3156.73) | 2132.59(1408.6,3045.65) |
|  | Rate95%(UI) | 35.69(28.14,43.35) | 31.08(23.95,37.74) | 42.09(33.71,51.25) | 41.1(31.14,47.06) | 31.89(23.81,37.39) | 53.81(41.84,61.59) | 51.62(39.43,60.41) | 49.22(37.39,58.27) | 55.46(41.02,65.93) | 32.04(12.05,61.13) | 27.88(9.83,56.43) | 36.89(13.62,80.4) | 30.09(10.27,54.67) | 24.02(7.96,45.53) | 37.82(13.1,72.57) | 17.92(11.51,25.29) | 15.75(9.94,23.27) | 20.8(13.74,29.71) |
| 85-89 years | Number95%(UI) | 24870.97(18897.62,29733.06) | 13848.45(10395.46,16564.19) | 11022.52(8590.86,13284.54) | 10458.34(7777.08,12146.97) | 5595.51(4098.7,6629.17) | 4862.83(3668.11,5593.69) | 8749.57(6325.3,10264.34) | 5456.45(3981.86,6468.93) | 3293.12(2338.16,3863.74) | 518.84(199.79,965.19) | 240.13(87.51,477.13) | 278.71(105.6,565.05) | 1937.78(664.96,3519.43) | 900.01(301.66,1724.07) | 1037.77(353.11,1969.2) | 3132.33(1999.63,4451.28) | 1609.72(871.83,2379.31) | 1522.6(972.09,2147.54) |
|  | Rate95%(UI) | 54.4(41.33,65.03) | 48.64(36.51,58.18) | 63.89(49.79,77) | 63.39(47.14,73.63) | 54.2(39.7,64.21) | 78.76(59.41,90.6) | 76.9(55.6,90.22) | 73.27(53.47,86.86) | 83.8(59.5,98.32) | 38.87(14.97,72.3) | 32.27(11.76,64.11) | 47.18(17.87,95.65) | 38.78(13.31,70.43) | 31.37(10.52,60.1) | 48.76(16.59,92.52) | 27.31(17.44,38.82) | 22.82(12.36,33.73) | 34.5(22.03,48.66) |
| 90-94 years | Number95%(UI) | 15200.15(11312.87,18036.98) | 9623.61(6961.58,11535.18) | 5576.54(4243.83,6741.02) | 7542.56(5385.44,8879.59) | 4743.36(3339.69,5653.65) | 2799.2(2100.75,3243.72) | 5047.7(3641.4,6032.68) | 3460.47(2509.75,4221.6) | 1587.23(1132.26,1896.58) | 140.33(53.99,247.44) | 71.48(24.11,144.55) | 68.85(27.47,134.23) | 816.45(287.96,1493.38) | 402.63(133.3,751.84) | 413.82(133.15,786.58) | 1612.71(945.82,2351.48) | 918.44(504.26,1395.81) | 694.27(443.45,1002.62) |
|  | Rate95%(UI) | 84.97(63.24,100.83) | 79.79(57.72,95.64) | 95.68(72.81,115.66) | 98.38(70.25,115.82) | 90.29(63.57,107.62) | 116(87.05,134.42) | 115.46(83.29,137.99) | 111.71(81.02,136.28) | 124.59(88.87,148.87) | 39.15(15.06,69.03) | 35.05(11.82,70.87) | 44.56(17.78,86.87) | 50.45(17.79,92.28) | 42.5(14.07,79.37) | 61.68(19.85,117.23) | 41.83(24.53,60.99) | 36.08(19.81,54.83) | 53.01(33.86,76.56) |
| 95+ years | Number95%(UI) | 6779.54(4662.59,8110.51) | 4902.75(3300.72,5946.92) | 1876.8(1349.06,2207.76) | 3873.01(2595.25,4608.84) | 2814.21(1868.18,3390.29) | 1058.8(746.08,1249.37) | 1993.37(1351.81,2463.79) | 1475(984.91,1830.56) | 518.37(348.05,643.5) | 33.27(13.18,61.41) | 20.77(6.94,40.6) | 12.5(5.11,24.25) | 268.44(94.54,490.61) | 141.83(49.71,284.11) | 126.6(41.21,250.76) | 596.23(309.93,915) | 440.3(221.62,696.82) | 155.93(88.87,234.67) |
|  | Rate95%(UI) | 124.39(85.55,148.81) | 124.49(83.81,151) | 124.12(89.22,146.01) | 139.81(93.68,166.37) | 136.54(90.64,164.49) | 149.3(105.2,176.17) | 180.58(122.46,223.2) | 178.29(119.05,221.27) | 187.44(125.85,232.68) | 43.73(17.32,80.72) | 42.82(14.3,83.71) | 45.33(18.52,87.94) | 59.19(20.85,108.19) | 48.88(17.13,97.91) | 77.52(25.23,153.53) | 57.31(29.79,87.95) | 62.29(31.35,98.58) | 46.75(26.65,70.36) |
| DALYs: Disability-adjusted life years. ASDR: age-standardized DALY rate. SDI: Sociodemographic Index. | | | | | | | | | | | | | | | | | | | |

| **Table S5. Age-specific DALYs and ASDR for ischemic heart disease attributable to high temperature, categorized by 5-year age groups, presented globally and by SDI region in 2021.** | | | | | | | | | | | | | | | | | | | |
| --- | --- | --- | --- | --- | --- | --- | --- | --- | --- | --- | --- | --- | --- | --- | --- | --- | --- | --- | --- |
|  |  | Global | | | High SDI | | | High-middle SDI | | | Low SDI | | | Low-middle SDI | | | Middle SDI | | |
| age | metric | Both | Female | Male | Both | Female | Male | Both | Female | Male | Both | Female | Male | Both | Female | Male | Both | Female | Male |
| 15-19 years | Number95%(UI) | 13786.54(2798.34,28684.09) | 6711.42(1460.39,13310.37) | 7075.12(1180.17,15489.06) | 348.47(66.96,728.9) | 125.87(24.48,251.02) | 222.61(38.03,474.43) | 381.3(-77.65,1121.84) | 139.51(-15.46,390.72) | 241.79(-64,740.52) | 1568.36(101.29,3400.61) | 874.75(45.45,1927.29) | 693.61(48.81,1556.96) | 8505.48(1728.55,17600.09) | 4251.31(962.24,8283.87) | 4254.18(747.36,9363.12) | 2977.68(726.49,6095.33) | 1317.85(326.02,2533.03) | 1659.83(377.97,3567.08) |
|  | Rate95%(UI) | 2.21(0.45,4.6) | 2.21(0.48,4.38) | 2.21(0.37,4.84) | 0.58(0.11,1.21) | 0.43(0.08,0.86) | 0.72(0.12,1.53) | 0.53(-0.11,1.55) | 0.41(-0.04,1.13) | 0.64(-0.17,1.95) | 1.27(0.08,2.74) | 1.42(0.07,3.13) | 1.11(0.08,2.5) | 4.61(0.94,9.54) | 4.7(1.06,9.16) | 4.52(0.79,9.95) | 1.63(0.4,3.34) | 1.5(0.37,2.88) | 1.76(0.4,3.77) |
| 20-24 years | Number95%(UI) | 25480.89(5304,52557.8) | 10215.65(2234.71,19938.67) | 15265.24(2998.93,31906.32) | 921.67(187.64,2002.42) | 309(66.31,669.77) | 612.67(122.09,1359.83) | 815.87(-239.82,2513.13) | 238.46(-48.31,688.7) | 577.41(-184.97,1797.46) | 2575.96(241.18,5383.66) | 1279.2(116.67,2678.55) | 1296.76(106.29,2835.03) | 15671.7(3467.67,31519.71) | 6317.58(1462.69,12117.84) | 9354.12(1884.16,19375.72) | 5488.37(1290.08,11024.75) | 2068.64(512.75,4047.62) | 3419.73(793.14,7020.25) |
|  | Rate95%(UI) | 4.27(0.89,8.8) | 3.48(0.76,6.79) | 5.03(0.99,10.52) | 1.41(0.29,3.06) | 0.98(0.21,2.12) | 1.81(0.36,4.01) | 1.09(-0.32,3.35) | 0.67(-0.14,1.93) | 1.47(-0.47,4.56) | 2.47(0.23,5.16) | 2.43(0.22,5.08) | 2.52(0.21,5.5) | 8.97(1.98,18.03) | 7.26(1.68,13.93) | 10.65(2.15,22.07) | 3.1(0.73,6.22) | 2.39(0.59,4.67) | 3.78(0.88,7.75) |
| 25-29 years | Number95%(UI) | 37594.36(7743.29,76714.6) | 13193.55(2704.79,25721.66) | 24400.82(4931.44,49270.87) | 2318.9(479.23,4837.55) | 637.97(141.43,1283.45) | 1680.94(322.87,3579.06) | 1447.2(-380.15,4272.29) | 331.75(-65.32,975.87) | 1115.46(-336.86,3334.66) | 2836.25(222.3,6112.04) | 1191.21(76.65,2638.35) | 1645.03(141.23,3731.99) | 21819.65(4930.29,42783.22) | 8035.9(1815.13,15492.53) | 13783.75(3025.72,26754.89) | 9160.92(2272.59,18378.81) | 2992.99(643.93,5874.07) | 6167.93(1620.08,12384.96) |
|  | Rate95%(UI) | 6.39(1.32,13.04) | 4.53(0.93,8.84) | 8.21(1.66,16.57) | 3.25(0.67,6.78) | 1.85(0.41,3.71) | 4.56(0.88,9.72) | 1.71(-0.45,5.04) | 0.82(-0.16,2.42) | 2.51(-0.76,7.51) | 3.29(0.26,7.1) | 2.7(0.17,5.99) | 3.91(0.34,8.87) | 13.48(3.05,26.43) | 9.89(2.23,19.07) | 17.1(3.75,33.18) | 4.98(1.24,10) | 3.3(0.71,6.48) | 6.62(1.74,13.29) |
| 30-34 years | Number95%(UI) | 67198.62(13872.91,135897.55) | 20355.18(4511.44,39980.22) | 46843.44(9515.02,96249.72) | 5334.59(1107.53,11651.23) | 1273.29(275.52,2715.62) | 4061.3(835.38,9063.19) | 3173.62(-858.63,9826.48) | 645.55(-124.14,1912.68) | 2528.08(-753.28,7983.34) | 5070.81(513.49,10410.2) | 2031.16(267.9,4258.99) | 3039.64(253.49,6373.81) | 36633.04(8868.9,68847.49) | 11722.39(2759.66,22024.59) | 24910.65(5958.1,47255.06) | 16967.31(3994.75,33893.15) | 4676.9(1172.31,9439.2) | 12290.41(2854.09,24925.35) |
|  | Rate95%(UI) | 11.12(2.3,22.48) | 6.81(1.51,13.37) | 15.33(3.11,31.5) | 6.87(1.43,15.01) | 3.4(0.74,7.25) | 10.11(2.08,22.56) | 2.97(-0.8,9.21) | 1.25(-0.24,3.71) | 4.58(-1.36,14.46) | 7(0.71,14.38) | 5.47(0.72,11.47) | 8.62(0.72,18.07) | 24.78(6,46.57) | 15.86(3.73,29.8) | 33.7(8.06,63.93) | 8.51(2,16.99) | 4.74(1.19,9.56) | 12.2(2.83,24.74) |
| 35-39 years | Number95%(UI) | 93535.54(20354.36,188779) | 27173.86(5974.2,52750.41) | 66361.67(14345.33,133686.86) | 9383.37(1852.14,19347.11) | 2078.63(412.42,4409.41) | 7304.74(1455.54,15498.62) | 4690.86(-1220.87,14723.97) | 958.26(-188.89,2915.64) | 3732.6(-1018.56,11625.87) | 6890.32(752.65,14197.64) | 2545.23(272.73,5209.94) | 4345.09(435.6,9013.41) | 48278.66(12212.77,91734.72) | 15578.03(3772.77,28985.03) | 32700.63(8440.45,62493.69) | 24262.94(5748.78,49703.08) | 6005.3(1593.95,12033.18) | 18257.64(4240.21,37678.42) |
|  | Rate95%(UI) | 16.68(3.63,33.66) | 9.78(2.15,18.99) | 23.44(5.07,47.23) | 11.93(2.35,24.6) | 5.48(1.09,11.62) | 17.94(3.57,38.05) | 4.62(-1.2,14.52) | 1.93(-0.38,5.88) | 7.19(-1.96,22.41) | 11.07(1.21,22.8) | 7.99(0.86,16.35) | 14.29(1.43,29.65) | 36.18(9.15,68.75) | 23.4(5.67,43.53) | 48.92(12.63,93.48) | 13.14(3.11,26.92) | 6.55(1.74,13.13) | 19.64(4.56,40.52) |
| 40-44 years | Number95%(UI) | 146153.17(33553.51,293810.82) | 40806.9(9860.17,79381.05) | 105346.27(23513.26,215324.34) | 15949.62(3222.23,33565.48) | 3400.95(729.01,7134.3) | 12548.67(2411.77,26618.71) | 7229.08(-1656.24,22943.31) | 1506.94(-265.15,4583.21) | 5722.14(-1362.65,18236.23) | 10683.18(1248.49,21841.47) | 3546.54(380.72,7574.49) | 7136.64(779.21,15033.25) | 74056.98(19917.19,139290.26) | 22445.59(6238.73,41485.7) | 51611.39(13416.56,97962.66) | 38187.33(9305.03,76027.21) | 9894.25(2374.6,19297.29) | 28293.08(7029.79,56934.59) |
|  | Rate95%(UI) | 29.22(6.71,58.73) | 16.45(3.97,32) | 41.78(9.32,85.39) | 21.09(4.26,44.38) | 9.26(1.99,19.43) | 32.25(6.2,68.4) | 7.82(-1.79,24.83) | 3.31(-0.58,10.07) | 12.21(-2.91,38.9) | 20.7(2.42,42.31) | 13.58(1.46,29) | 27.99(3.06,58.95) | 64.25(17.28,120.84) | 38.91(10.82,71.92) | 89.63(23.3,170.12) | 23.16(5.64,46.1) | 12.09(2.9,23.58) | 34.06(8.46,68.54) |
| 45-49 years | Number95%(UI) | 191158.2(41538.82,389929.66) | 57001.4(13667.74,110867.69) | 134156.8(27952.37,274732.63) | 19993.27(3646.77,43553.18) | 4520.46(774.9,9825.11) | 15472.82(2940.31,33580.85) | 10193.97(-2353.96,33133.35) | 2236.48(-432.52,7162.17) | 7957.49(-1904.79,25687.39) | 14455.76(1765.68,30337.73) | 5039.23(640.34,10429.4) | 9416.53(1030.73,20046.69) | 90713.34(24322.65,170727.07) | 28817.2(8000.07,52396.67) | 61896.14(15894.02,118161.17) | 55734.93(13374.47,113725.98) | 16370.02(3914.82,32817.38) | 39364.91(9367.6,81237.69) |
|  | Rate95%(UI) | 40.37(8.77,82.35) | 24.19(5.8,47.05) | 56.4(11.75,115.5) | 27.25(4.97,59.36) | 12.59(2.16,27.36) | 41.31(7.85,89.65) | 10.52(-2.43,34.19) | 4.64(-0.9,14.85) | 16.35(-3.91,52.77) | 34.67(4.23,72.76) | 24.27(3.08,50.22) | 44.99(4.92,95.78) | 92.1(24.7,173.34) | 58.29(16.18,105.99) | 126.18(32.4,240.88) | 34.27(8.22,69.92) | 20.18(4.83,40.46) | 48.27(11.49,99.62) |
| 50-54 years | Number95%(UI) | 246271.78(51158.66,503634.93) | 70928.8(15675.37,142276.09) | 175342.98(35291.08,363578.78) | 21311.53(2806.71,49618.12) | 5085.08(678.23,11580.86) | 16226.45(2154.01,37987.1) | 14811.05(-3278.05,48936.58) | 3350.86(-671.61,11085.08) | 11460.18(-2735.15,37532.14) | 17881.57(2122.17,36794.59) | 5940.79(673.59,11913.51) | 11940.78(1358.62,25181.12) | 118010.06(31176.72,221418.31) | 35828.03(9630.42,68036.71) | 82182.03(21188.57,156092.83) | 74169.57(16801.45,153976.15) | 20699.89(4747.15,42297.96) | 53469.68(12089.31,110861.58) |
|  | Rate95%(UI) | 55.35(11.5,113.2) | 31.81(7.03,63.82) | 78.99(15.9,163.79) | 28.7(3.78,66.81) | 13.81(1.84,31.46) | 43.32(5.75,101.42) | 15.29(-3.38,50.51) | 6.91(-1.39,22.87) | 23.67(-5.65,77.52) | 54.65(6.49,112.45) | 36.5(4.14,73.2) | 72.62(8.26,153.14) | 140.59(37.14,263.79) | 84.72(22.77,160.88) | 197.34(50.88,374.81) | 47.31(10.72,98.22) | 26.23(6.02,53.6) | 68.69(15.53,142.42) |
| 55-59 years | Number95%(UI) | 301928.74(63112.34,626485.22) | 101917.34(24409.83,207408.43) | 200011.39(39061.25,425358.75) | 22128.08(2105.29,54105.92) | 5648.96(546.23,13372.16) | 16479.12(1281.66,40618.69) | 19833.87(-4195.88,65331.16) | 5577.48(-1036.6,17708.33) | 14256.39(-3235.31,47761.43) | 20947.3(2836.93,42875.97) | 8008.98(1016.9,15639.98) | 12938.32(1642.38,26666.83) | 144903.26(39613.61,273111.07) | 50059.2(14260.83,90385.13) | 94844.06(24524.97,181563.18) | 94014.16(21018.27,193643.31) | 32592.84(7943.88,66529.01) | 61421.32(13160.28,127536.86) |
|  | Rate95%(UI) | 76.3(15.95,158.31) | 50.7(12.14,103.19) | 102.72(20.06,218.44) | 30.42(2.89,74.39) | 15.47(1.5,36.63) | 45.49(3.54,112.12) | 22.05(-4.67,72.64) | 12.16(-2.26,38.6) | 32.35(-7.34,108.39) | 82.69(11.2,169.26) | 62.89(7.99,122.81) | 102.72(13.04,211.71) | 208.61(57.03,393.18) | 141.78(40.39,256) | 277.69(71.81,531.59) | 68.15(15.24,140.38) | 46.29(11.28,94.48) | 90.96(19.49,188.86) |
| 60-64 years | Number95%(UI) | 313897.78(63113.33,673570.3) | 110798(24049.67,227381.34) | 203099.78(39846.95,442486.8) | 21326.7(732.59,58638.49) | 5966.91(240.77,15977.67) | 15359.79(461.83,43434.28) | 24061.45(-4059.76,80072.07) | 7553.67(-1122.34,25429.63) | 16507.78(-3008.38,55237.87) | 21957.01(3088.95,43472.97) | 8681.78(1234.89,16833.6) | 13275.23(1922.35,26920.17) | 143320.89(38090.77,272952.24) | 52936.64(13976.52,97085.63) | 90384.25(23429.46,173744.72) | 103115.54(22633.26,214182.52) | 35621.39(8348.12,72735.26) | 67494.15(14670.07,140481.93) |
|  | Rate95%(UI) | 98.08(19.72,210.46) | 67.35(14.62,138.22) | 130.58(25.62,284.49) | 31.28(1.07,86.02) | 17.13(0.69,45.86) | 46.08(1.39,130.3) | 32.99(-5.57,109.79) | 19.89(-2.95,66.95) | 47.23(-8.61,158.06) | 111.36(15.67,220.49) | 87.03(12.38,168.75) | 136.28(19.73,276.35) | 250.71(66.63,477.48) | 180.03(47.53,330.17) | 325.58(84.4,625.85) | 101.31(22.24,210.43) | 68.29(16,139.44) | 136.03(29.57,283.12) |
| 65-69 years | Number95%(UI) | 323634.94(58735.59,706918.62) | 126072.11(24800.75,267954.59) | 197562.83(34717.41,435889.48) | 19968.96(-476.06,59050.28) | 5953.22(-221.84,17691.59) | 14015.74(-306.1,41358.68) | 29384.04(-5820.32,100232.73) | 10896.92(-1931.24,37381.04) | 18487.12(-4072.09,62929.65) | 23400.87(3154.91,46761.73) | 9702.44(1463.42,18975.61) | 13698.43(2138.38,27543.71) | 142618.03(37943.5,269128.94) | 58373.53(15772.83,109611.89) | 84244.5(21981.01,161151.22) | 108139.48(21665.24,229764.45) | 41101.35(8576.7,85398.85) | 67038.13(13130.31,141770.96) |
|  | Rate95%(UI) | 117.33(21.29,256.28) | 87.54(17.22,186.07) | 149.86(26.33,330.64) | 32.94(-0.79,97.4) | 18.86(-0.7,56.04) | 48.24(-1.05,142.35) | 44.25(-8.76,150.94) | 30.73(-5.45,105.4) | 59.75(-13.16,203.39) | 155.22(20.93,310.17) | 126.29(19.05,247) | 185.27(28.92,372.52) | 321.5(85.54,606.69) | 253.66(68.54,476.32) | 394.62(102.96,754.87) | 121.32(24.31,257.77) | 89.06(18.58,185.04) | 155.95(30.55,329.81) |
| 70-74 years | Number95%(UI) | 289601.45(49224.64,639533.95) | 119951.45(20657.74,264651.31) | 169650(28971.52,378075.93) | 18140.76(-1325.15,57856.04) | 6473.66(-311.88,20214.02) | 11667.1(-1012.23,37061.31) | 31086.92(-6433.49,107403.64) | 13438.67(-2343.03,47128.02) | 17648.25(-4269.31,60490.42) | 22036.39(3713.18,43169.29) | 9257.84(1275.04,18132.14) | 12778.55(2361,24776.32) | 126758.01(35467.19,234597.33) | 53744.19(14950.49,99183.55) | 73013.82(20084.15,135573.47) | 91464.2(15362.28,203993.44) | 36988.93(6315.17,82220.24) | 54475.26(8394.76,119829.98) |
|  | Rate95%(UI) | 140.69(23.91,310.7) | 109.6(18.87,241.81) | 176(30.06,392.23) | 34(-2.48,108.42) | 22.85(-1.1,71.34) | 46.62(-4.04,148.08) | 63.64(-13.17,219.87) | 49.99(-8.72,175.31) | 80.35(-19.44,275.39) | 212.49(35.81,416.27) | 174.4(24.02,341.58) | 252.43(46.64,489.44) | 403.09(112.78,746.01) | 326.2(90.74,601.99) | 487.7(134.15,905.57) | 148.41(24.93,331.01) | 114.35(19.52,254.19) | 186.04(28.67,409.23) |
| 75-79 years | Number95%(UI) | 223820.78(33936.17,512601.47) | 100740.42(15197.47,230484.66) | 123080.36(19063.02,280639.89) | 14003.14(-1863.99,47006.45) | 5414.83(-709.29,18151.31) | 8588.31(-1152.39,28631.78) | 26895.15(-6482.7,94224.12) | 12843.56(-2562.34,45425.65) | 14051.58(-3938.43,49047.72) | 15651.49(2612.62,30199.12) | 7321.84(1158.43,14316.96) | 8329.65(1510.83,16659.18) | 92668.24(26589.96,172500.3) | 42599.86(11728.71,79041.88) | 50068.38(14421.26,94337.35) | 74503.83(11329.09,170398.3) | 32512.86(5226.63,73707.35) | 41990.97(5831.62,97061.55) |
|  | Rate95%(UI) | 169.71(25.73,388.67) | 139.73(21.08,319.68) | 205.87(31.89,469.4) | 38.67(-5.15,129.8) | 27.23(-3.57,91.3) | 52.59(-7.06,175.32) | 90.05(-21.7,315.47) | 75.98(-15.16,268.73) | 108.39(-30.38,378.33) | 243.76(40.69,470.33) | 220.2(34.84,430.58) | 269.07(48.8,538.13) | 461.52(132.43,859.11) | 396.24(109.09,735.2) | 536.76(154.6,1011.34) | 190.15(28.91,434.9) | 153.61(24.69,348.23) | 233.08(32.37,538.77) |
| 80-84 years | Number95%(UI) | 176233.44(18686.85,426123.99) | 86675.15(9418.71,211771.86) | 89558.29(8840.56,218407.35) | 12533.24(-2248.48,44659.46) | 5521.38(-1079.63,20173.58) | 7011.87(-1177.38,24569.59) | 30550.84(-7258.58,106596.47) | 16859.88(-3220.83,60017.12) | 13690.96(-4082.19,48543.98) | 10362.26(1938.41,20309.71) | 4725.16(733.26,9540.86) | 5637.1(1155.96,11177.31) | 62428.76(17727.14,114942.18) | 30390.2(8387,55766.84) | 32038.56(9295.04,60787.58) | 60267.43(6640.47,143204.14) | 29128.54(3716.79,68656.29) | 31138.89(2747.51,73233.38) |
|  | Rate95%(UI) | 201.22(21.34,486.54) | 170.18(18.49,415.8) | 244.35(24.12,595.9) | 46.47(-8.34,165.58) | 35.31(-6.9,129) | 61.86(-10.39,216.77) | 139.93(-33.25,488.25) | 125.64(-24,447.26) | 162.73(-48.52,576.99) | 307.2(57.47,602.11) | 260.2(40.38,525.38) | 362.02(74.24,717.82) | 542.83(154.14,999.45) | 471.98(130.26,866.1) | 632.96(183.63,1200.93) | 253(27.88,601.18) | 214.68(27.39,506) | 303.73(26.8,714.32) |
| 85-89 years | Number95%(UI) | 106216.88(4029.92,282269.87) | 52255.77(1648.7,142673.65) | 53961.11(2381.23,142906.71) | 10829.13(-2215.82,40367.78) | 5358.52(-1194.89,20202.2) | 5470.61(-1066.71,19935.37) | 22566.09(-6394.14,82270.75) | 12502.49(-2968.58,46749.17) | 10063.6(-3582,36195.02) | 4242(749.25,8300.51) | 1889.85(284.22,3799.84) | 2352.15(447.99,4709.62) | 28183.17(7924.14,52903.35) | 13069.56(3558.99,24880.34) | 15113.6(4293.45,28632.76) | 40329.76(2946.45,100595.04) | 19395.33(1538.67,47966.57) | 20934.43(1407.78,51974.12) |
|  | Rate95%(UI) | 232.31(8.81,617.36) | 183.55(5.79,501.15) | 312.77(13.8,828.32) | 65.64(-13.43,244.68) | 51.9(-11.57,195.68) | 88.61(-17.28,322.9) | 198.34(-56.2,723.12) | 167.88(-39.86,627.73) | 256.08(-91.15,921.02) | 317.76(56.13,621.78) | 253.95(38.19,510.6) | 398.15(75.83,797.2) | 564(158.58,1058.69) | 455.6(124.06,867.31) | 710.1(201.72,1345.28) | 351.67(25.69,877.19) | 274.92(21.81,679.9) | 474.39(31.9,1177.77) |
| 90-94 years | Number95%(UI) | 49982.01(-464.45,142030.59) | 27350.58(-821.05,79927.31) | 22631.43(292.57,62521.92) | 7743.51(-1623.16,29441.41) | 4530.29(-1025.14,17551.68) | 3213.22(-621.9,11851.75) | 12022.1(-3456.36,44721.2) | 7684.89(-1948.73,29094.86) | 4337.21(-1441.56,16396.69) | 1348.86(230.46,2753.84) | 618.16(95.93,1281.94) | 730.7(126.39,1460.83) | 11355.62(3020.56,21433.94) | 5235.04(1551.51,9672.83) | 6120.58(1525.38,12222.15) | 17480.14(1131.77,43705.16) | 9262.04(497.86,23371.74) | 8218.11(626.59,20375.67) |
|  | Rate95%(UI) | 279.39(-2.6,793.94) | 226.77(-6.81,662.7) | 388.29(5.02,1072.69) | 101(-21.17,384.02) | 86.23(-19.51,334.1) | 133.15(-25.77,491.12) | 274.99(-79.06,1022.95) | 248.08(-62.91,939.21) | 340.44(-113.15,1287.03) | 376.29(64.29,768.23) | 303.1(47.04,628.56) | 472.9(81.8,945.44) | 701.73(186.66,1324.53) | 552.65(163.79,1021.14) | 912.2(227.34,1821.56) | 453.4(29.36,1133.62) | 363.82(19.56,918.07) | 627.51(47.85,1555.83) |
| 95+ years | Number95%(UI) | 17444.4(-742.32,51370.69) | 11426(-814.18,34976.98) | 6018.4(95.86,16387.71) | 4032.58(-946.76,15534.18) | 2817.3(-692.44,11035.76) | 1215.28(-262.24,4495.78) | 3913.36(-1257.89,14877.05) | 2930.96(-936.57,11346.36) | 982.4(-329.09,3657.79) | 339.46(61.41,708.41) | 188.73(32.77,390.76) | 150.73(27.53,308.72) | 3854.07(980.74,7599.29) | 1960.45(612.82,3645.58) | 1893.61(387.03,3846.11) | 5292.55(362.82,13153.76) | 3520.13(162.15,9213.56) | 1772.42(224.56,4047.52) |
|  | Rate95%(UI) | 320.06(-13.62,942.53) | 290.13(-20.67,888.13) | 398.03(6.34,1083.81) | 145.57(-34.18,560.75) | 136.69(-33.6,535.44) | 171.36(-36.98,633.94) | 354.51(-113.95,1347.72) | 354.27(-113.21,1371.47) | 355.23(-119,1322.63) | 446.19(80.71,931.13) | 389.11(67.56,805.61) | 546.6(99.85,1119.53) | 849.88(216.27,1675.75) | 675.64(211.2,1256.4) | 1159.41(236.97,2354.88) | 508.72(34.87,1264.33) | 498.01(22.94,1303.49) | 531.41(67.33,1213.53) |

| **Table S6. Age-specific DALYs and ASDR for ischemic heart disease attributable to low temperature, categorized by 5-year age groups, presented globally and by SDI region in 2021.** | | | | | | | | | | | | | | | | | | | |
| --- | --- | --- | --- | --- | --- | --- | --- | --- | --- | --- | --- | --- | --- | --- | --- | --- | --- | --- | --- |
|  |  | Global | | | High SDI | | | High-middle SDI | | | Low SDI | | | Low-middle SDI | | | Middle SDI | | |
| age | metric | Both | Female | Male | Both | Female | Male | Both | Female | Male | Both | Female | Male | Both | Female | Male | Both | Female | Male |
| 15-19 years | Number95%(UI) | 31664.43(25503.29,39874.94) | 14093.97(10799.41,18163.25) | 17570.46(14433.29,22497.26) | 770.19(641.64,961.37) | 230.77(183.22,294.27) | 539.42(453.13,678.72) | 2618.35(2173.95,3305.9) | 800.89(647.8,1021.17) | 1817.45(1485.82,2293.35) | 5268.78(3991.65,6651.49) | 2836.42(2089.22,3650.74) | 2432.35(1900.43,3126.89) | 13864.02(10346.65,18588.86) | 6739.65(4708.62,9137.44) | 7124.38(5314.88,9848.61) | 9128.44(7676.73,11239.69) | 3480.02(2772.88,4395.75) | 5648.41(4787.52,7022.11) |
|  | Rate95%(UI) | 5.07(4.09,6.39) | 4.64(3.56,5.98) | 5.49(4.51,7.02) | 1.28(1.07,1.6) | 0.79(0.63,1.01) | 1.73(1.46,2.18) | 3.61(3,4.56) | 2.33(1.88,2.97) | 4.78(3.91,6.03) | 4.25(3.22,5.36) | 4.6(3.39,5.92) | 3.9(3.05,5.02) | 7.51(5.61,10.07) | 7.46(5.21,10.11) | 7.57(5.65,10.46) | 5.01(4.21,6.16) | 3.96(3.16,5) | 5.98(5.07,7.43) |
| 20-24 years | Number95%(UI) | 58687.85(46676.69,74490.04) | 21627.58(16436.7,27876.62) | 37060.27(29751.63,47250.33) | 2031.65(1678.9,2526.62) | 553.47(433.81,716.13) | 1478.18(1227.36,1839.67) | 5637.64(4743.36,7082.23) | 1467.52(1163.75,1894.72) | 4170.12(3428.81,5209.45) | 7830.03(5961.6,9957.19) | 3760.58(2699.05,4993.94) | 4069.45(3101.42,5362.24) | 25559.48(18422.66,34139.7) | 10022.53(6805.68,14178.82) | 15536.95(11446.11,21162.87) | 17606.51(14796.38,21647.94) | 5815.1(4646.05,7311.46) | 11791.41(9854.67,14481.08) |
|  | Rate95%(UI) | 9.83(7.82,12.47) | 7.36(5.6,9.49) | 12.21(9.81,15.57) | 3.11(2.57,3.86) | 1.76(1.38,2.27) | 4.36(3.62,5.43) | 7.52(6.33,9.45) | 4.12(3.27,5.32) | 10.59(8.71,13.23) | 7.51(5.72,9.55) | 7.13(5.12,9.47) | 7.89(6.02,10.4) | 14.62(10.54,19.53) | 11.52(7.82,16.3) | 17.7(13.04,24.1) | 9.93(8.35,12.21) | 6.71(5.36,8.44) | 13.02(10.88,15.99) |
| 25-29 years | Number95%(UI) | 88277.12(70663.99,111591.03) | 28603.64(21726.67,36784.95) | 59673.47(48273.99,75535.8) | 4482.88(3563.13,5710.14) | 1066.55(821.42,1370.35) | 3416.32(2713.98,4346.71) | 10276.32(8853.22,12473.1) | 2138.37(1728.19,2750.23) | 8137.94(6989.32,9955.69) | 9521.77(7319.29,12155.72) | 3884.73(2776.81,5099.21) | 5637.04(4253.08,7517.85) | 35632.79(25924.07,47275.65) | 13016.73(8907.31,18015.38) | 22616.06(16481.82,30478.05) | 28325.87(23741.38,34824.2) | 8484.96(6653.18,10705.89) | 19840.91(16685.4,23962.13) |
|  | Rate95%(UI) | 15(12.01,18.97) | 9.83(7.47,12.64) | 20.07(16.23,25.4) | 6.28(4.99,8) | 3.09(2.38,3.97) | 9.28(7.37,11.8) | 12.13(10.45,14.73) | 5.3(4.29,6.82) | 18.34(15.75,22.43) | 11.05(8.5,14.11) | 8.82(6.3,11.58) | 13.39(10.11,17.86) | 22.01(16.01,29.2) | 16.02(10.96,22.17) | 28.05(20.44,37.8) | 15.41(12.92,18.95) | 9.37(7.34,11.82) | 21.29(17.9,25.71) |
| 30-34 years | Number95%(UI) | 166993.87(135766.24,210152.38) | 46175.4(35874.33,58684.72) | 120818.47(98706.3,152071.74) | 10539.69(8559.45,13748.58) | 2349.02(1846.42,3043.15) | 8190.67(6626.75,10738.02) | 25856.65(22343.68,31597.94) | 4911.89(3978.38,6257.62) | 20944.76(18091.43,25658.25) | 15306.34(11927.93,19347.37) | 5677.38(4017.17,7472.75) | 9628.95(7249.43,12502.05) | 60293.85(42807.44,80402.75) | 19175.42(13471.52,25996) | 41118.42(29712.39,54464.73) | 54927.28(46416.15,67025.97) | 14041.09(11214.61,17524.67) | 40886.19(34627.78,49613.47) |
|  | Rate95%(UI) | 27.63(22.46,34.77) | 15.45(12,19.63) | 39.54(32.3,49.77) | 13.58(11.03,17.72) | 6.28(4.93,8.13) | 20.39(16.49,26.73) | 24.23(20.94,29.61) | 9.54(7.73,12.15) | 37.94(32.77,46.48) | 21.14(16.48,26.72) | 15.3(10.82,20.13) | 27.29(20.55,35.44) | 40.79(28.96,54.39) | 25.94(18.23,35.17) | 55.62(40.19,73.68) | 27.53(23.27,33.6) | 14.22(11.36,17.75) | 40.59(34.38,49.25) |
| 35-39 years | Number95%(UI) | 240399.51(196564.93,298210.62) | 63656.69(49948.89,79518.5) | 176742.82(145298.02,221582.31) | 19673.9(16333.9,24921.44) | 4147.1(3323.98,5260.88) | 15526.8(12804.23,19581.45) | 41956.86(36328.13,51241.09) | 7752(6381.01,9686.45) | 34204.86(29076.94,42142.31) | 20774.44(16183,26229.07) | 7275.83(5319.65,9519.2) | 13498.6(10369.92,17306.9) | 79549.55(57258.98,106020.94) | 25763.06(17919.21,34804.03) | 53786.49(39140.15,72365.6) | 78329.64(66519.1,95815.65) | 18689.5(15388.81,23150.94) | 59640.14(49878.76,73126.23) |
|  | Rate95%(UI) | 42.86(35.05,53.17) | 22.91(17.98,28.62) | 62.44(51.33,78.28) | 25.01(20.77,31.68) | 10.93(8.76,13.87) | 38.12(31.44,48.08) | 41.36(35.81,50.52) | 15.64(12.88,19.55) | 65.93(56.04,81.22) | 33.36(25.99,42.12) | 22.83(16.69,29.87) | 44.4(34.11,56.93) | 59.62(42.91,79.45) | 38.69(26.91,52.27) | 80.46(58.55,108.25) | 42.43(36.03,51.9) | 20.39(16.79,25.26) | 64.14(53.65,78.65) |
| 40-44 years | Number95%(UI) | 375602.15(308616.17,469926.06) | 97687.87(78314.98,123351.56) | 277914.28(228101.32,350511.78) | 34939.16(28892.04,43460.61) | 7319.38(6053.56,9237.32) | 27619.78(22749.02,34640.29) | 67045.85(57975.98,82456.11) | 12866.32(10723.35,16044.27) | 54179.52(46451.06,67964.13) | 32737.6(25611.94,41209.06) | 10537.3(7772.84,13584.8) | 22200.29(17100.98,28497.88) | 124077.03(91686.75,164097.24) | 37139.05(26217.29,50093.25) | 86937.98(63907.46,116774.18) | 116599.39(97281.52,144000.45) | 29776.08(24170.83,36621.95) | 86823.31(72609.52,107675.39) |
|  | Rate95%(UI) | 75.08(61.69,93.94) | 39.38(31.57,49.72) | 110.21(90.46,139) | 46.2(38.2,57.47) | 19.94(16.49,25.16) | 70.97(58.46,89.01) | 72.56(62.74,89.24) | 28.27(23.56,35.25) | 115.56(99.08,144.97) | 63.42(49.62,79.83) | 40.34(29.76,52.01) | 87.06(67.06,111.76) | 107.64(79.54,142.36) | 64.39(45.45,86.84) | 150.97(110.98,202.78) | 70.7(58.99,87.32) | 36.38(29.53,44.74) | 104.52(87.41,129.62) |
| 45-49 years | Number95%(UI) | 524650.62(433650.62,651267.43) | 143403.85(113959.12,179783.9) | 381246.77(315672.54,475125.87) | 54250.27(46975.55,66658.81) | 11482.51(9756.24,14215.59) | 42767.77(36980.95,52271.55) | 102621.63(88548.06,126079.19) | 21181.93(17767.12,26836.52) | 81439.69(69193.26,100836.17) | 43794.45(34357.51,55663.88) | 14307.47(10639.15,18518.39) | 29486.98(22655.11,37869.47) | 155453.14(113169.2,203489.79) | 48471.02(35173.06,64312.24) | 106982.12(78369.04,140910.2) | 168208.11(141440.36,206293.29) | 47883.59(38669.56,59500.91) | 120324.52(101206.79,147329.75) |
|  | Rate95%(UI) | 110.8(91.58,137.54) | 60.86(48.36,76.3) | 160.28(132.71,199.75) | 73.95(64.03,90.86) | 31.98(27.17,39.59) | 114.17(98.72,139.54) | 105.9(91.37,130.1) | 43.92(36.84,55.65) | 167.3(142.14,207.15) | 105.03(82.4,133.5) | 68.89(51.23,89.17) | 140.89(108.24,180.94) | 157.83(114.9,206.61) | 98.05(71.15,130.09) | 218.09(159.76,287.25) | 103.41(86.96,126.83) | 59.03(47.67,73.36) | 147.56(124.11,180.67) |
| 50-54 years | Number95%(UI) | 736968.73(621503.07,908252.63) | 199157.66(163641.52,245821.35) | 537811.07(449050.61,658298.59) | 82590.19(73678.44,99370.76) | 17655.52(15554.73,21431.62) | 64934.67(57939.56,78096.8) | 154707(133242.33,188856.53) | 34171.73(28341.9,42516.08) | 120535.27(102906.79,148956.25) | 52710.43(41118.78,66791.45) | 16913.51(12668.94,21746.52) | 35796.92(27509.26,46052.31) | 203410.72(150159.32,266573.44) | 62311.97(45920.68,82767.03) | 141098.74(104067.31,186438.1) | 243082.58(205403.32,292586.77) | 67993.31(56064.69,82349.31) | 175089.27(147200.49,211473.84) |
|  | Rate95%(UI) | 165.64(139.69,204.14) | 89.33(73.4,110.26) | 242.28(202.29,296.56) | 111.21(99.21,133.81) | 47.97(42.26,58.22) | 173.36(154.69,208.5) | 159.68(137.52,194.92) | 70.5(58.47,87.71) | 248.96(212.55,307.66) | 161.1(125.67,204.13) | 103.91(77.84,133.61) | 217.7(167.3,280.07) | 242.34(178.9,317.59) | 147.34(108.58,195.71) | 338.81(249.89,447.68) | 155.07(131.03,186.65) | 86.16(71.04,104.35) | 224.93(189.1,271.67) |
| 55-59 years | Number95%(UI) | 959762.37(807986.56,1186290.18) | 294905.92(240027.41,364839.07) | 664856.45(562409.32,820102.48) | 115120.31(103793.3,136661.22) | 26187(23204.62,31372.71) | 88933.31(80443.53,105172.05) | 221193.66(190730.66,271881.2) | 58395.97(48671.62,73809.89) | 162797.69(139845,203256.7) | 60543.87(46937.21,76551.22) | 22428.36(16612.15,28373.24) | 38115.51(29478.54,49318.12) | 252777.96(189056.25,333299.3) | 86444.31(63733.54,113787.19) | 166333.64(123586.69,218580.37) | 309523.07(261861.93,374648.18) | 101296.03(82858.96,125782.88) | 208227.04(173716.56,251582.3) |
|  | Rate95%(UI) | 242.53(204.18,299.77) | 146.72(119.41,181.51) | 341.43(288.82,421.16) | 158.27(142.7,187.88) | 71.73(63.56,85.93) | 245.49(222.06,290.32) | 245.94(212.07,302.3) | 127.29(106.1,160.9) | 369.47(317.38,461.29) | 239.01(185.29,302.2) | 176.12(130.45,222.8) | 302.6(234.03,391.53) | 363.91(272.17,479.83) | 244.84(180.51,322.28) | 487(361.84,639.97) | 224.38(189.83,271.59) | 143.85(117.67,178.63) | 308.35(257.25,372.56) |
| 60-64 years | Number95%(UI) | 1088249.59(925530.03,1348966.95) | 356513.1(298977.76,438943.14) | 731736.49(620270.09,902244.24) | 145790.54(131932.61,173174.47) | 36812.32(32875.9,44134.08) | 108978.22(98953.23,129107.13) | 293689.74(256984.68,360345.03) | 89553.09(76219.06,112883.09) | 204136.64(176116.7,254512.91) | 61905.67(48422.27,77556.05) | 22656.95(16913.46,28794.76) | 39248.72(29987.26,49658.23) | 254611.57(194163.43,328551.81) | 92916.43(69346.62,118810.05) | 161695.13(121604.02,212401.5) | 331436.86(280271.71,405276.73) | 114341.4(95964.15,139322.48) | 217095.46(182972.92,264411.57) |
|  | Rate95%(UI) | 340.03(289.18,421.49) | 216.71(181.74,266.82) | 470.46(398.79,580.08) | 213.86(193.53,254.03) | 105.67(94.37,126.69) | 326.94(296.86,387.32) | 402.68(352.35,494.07) | 235.76(200.65,297.17) | 584.11(503.93,728.26) | 313.98(245.59,393.35) | 227.13(169.55,288.65) | 402.91(307.84,509.77) | 445.39(339.65,574.74) | 315.99(235.84,404.06) | 582.45(438.03,765.1) | 325.63(275.36,398.18) | 219.19(183.97,267.08) | 437.53(368.76,532.89) |
| 65-69 years | Number95%(UI) | 1227466.31(1055025.7,1507426.82) | 455820.29(383303.14,558971.5) | 771646.01(658933.07,934537.27) | 162337.69(146222.25,193521.14) | 46161.05(40273.19,55653.46) | 116176.64(105194.87,138219.25) | 357265.85(309734.29,433577.96) | 133070.78(112564.7,165954.21) | 224195.07(192348.08,276739.53) | 65158.86(50986.51,82386.18) | 26503.29(20050.86,33257.62) | 38655.57(29915,49272.9) | 253338.62(191556.89,326724.6) | 101844.71(76775.07,130469.27) | 151493.9(113250.8,199898.62) | 388392.49(330545.74,467217.61) | 147922.79(123558.84,179709.22) | 240469.7(201436.44,288297.16) |
|  | Rate95%(UI) | 444.99(382.47,546.48) | 316.52(266.17,388.15) | 585.32(499.82,708.88) | 267.77(241.19,319.21) | 146.21(127.56,176.28) | 399.87(362.07,475.74) | 538.02(466.44,652.94) | 375.22(317.4,467.95) | 724.61(621.68,894.44) | 432.19(338.19,546.46) | 344.99(261,432.91) | 522.8(404.59,666.4) | 571.09(431.82,736.53) | 442.57(333.63,566.96) | 709.64(530.5,936.38) | 435.73(370.83,524.16) | 320.52(267.73,389.39) | 559.42(468.61,670.68) |
| 70-74 years | Number95%(UI) | 1210486.96(1048360.97,1475220.69) | 495115.2(423247.71,610176.26) | 715371.77(612226.88,863219.33) | 178190.64(159756.57,211209.18) | 58769.49(50777.08,70457.65) | 119421.16(108121.51,140728.1) | 376300.16(326947.07,462645.56) | 166285.61(140337.47,208406.7) | 210014.55(180691.62,260527.01) | 61796.05(47307.64,77505.17) | 25341.26(19258.99,32304.61) | 36454.79(27724.91,45886.67) | 221324.82(167405.9,288061.28) | 93729.75(69918.74,121533.46) | 127595.07(95471.52,165866.84) | 371874.62(317465.64,449436.21) | 150591.97(126194.92,180812.25) | 221282.65(184363.76,268804.97) |
|  | Rate95%(UI) | 588.07(509.31,716.69) | 452.38(386.71,557.5) | 742.15(635.15,895.54) | 333.94(299.39,395.82) | 207.42(179.22,248.68) | 477.16(432.01,562.29) | 770.34(669.31,947.1) | 618.55(522.03,775.23) | 956.11(822.62,1186.07) | 595.89(456.18,747.37) | 477.39(362.81,608.57) | 720.14(547.69,906.46) | 703.8(532.34,916.02) | 568.89(424.37,737.65) | 852.28(637.71,1107.92) | 603.42(515.13,729.27) | 465.56(390.14,558.99) | 755.69(629.61,917.99) |
| 75-79 years | Number95%(UI) | 1006815.12(870778.51,1219530.58) | 455236.84(387384.38,555221.13) | 551578.28(471577.02,659296.71) | 164241.83(142965.82,195596.72) | 62701.25(52546.02,75494.81) | 101540.58(90567.42,120310.83) | 312017.59(271438.09,382700.29) | 155897.49(132448.03,195571.97) | 156120.09(132973.32,188899.25) | 43207.68(33242.32,53527.52) | 19479.12(14890.77,24332.68) | 23728.56(17937.15,30605.28) | 164459.4(125702.75,213005.4) | 76356.22(57352.69,98177.36) | 88103.18(66247.37,115529.18) | 321982.65(276489.09,378749.92) | 140372.24(115541.7,167241.73) | 181610.41(151347.8,218285.03) |
|  | Rate95%(UI) | 763.41(660.26,924.7) | 631.42(537.3,770.1) | 922.58(788.77,1102.75) | 453.54(394.79,540.13) | 315.37(264.29,379.71) | 621.76(554.57,736.7) | 1044.65(908.79,1281.3) | 922.27(783.55,1156.98) | 1204.23(1025.68,1457.07) | 672.93(517.73,833.65) | 585.83(447.83,731.8) | 766.48(579.41,988.62) | 819.06(626.04,1060.84) | 710.22(533.46,913.18) | 944.51(710.21,1238.53) | 821.77(705.66,966.66) | 663.2(545.88,790.14) | 1008.08(840.1,1211.66) |
| 80-84 years | Number95%(UI) | 990340.73(837240.93,1200792.6) | 509985.15(416332.7,621751.32) | 480355.57(408708.1,575218.72) | 171818.11(138965.31,206550.21) | 77229.13(59410.68,95135.58) | 94588.97(79741.79,112350.77) | 384476.81(323908.62,469162.29) | 223709.88(182666.9,278601.36) | 160766.93(137471.61,196819.02) | 28428.59(21457.31,35926.61) | 12800.45(9756.94,16429.12) | 15628.14(11651.95,20413.32) | 114736.37(87849.79,148059.1) | 57041.11(43277.32,73373.63) | 57695.26(43662.36,76408.65) | 289915.42(241901.21,340208.1) | 138664.15(112366.37,164463.72) | 151251.27(127506.49,179910.84) |
|  | Rate95%(UI) | 1130.74(955.94,1371.03) | 1001.32(817.44,1220.77) | 1310.59(1115.11,1569.41) | 637.02(515.21,765.79) | 493.85(379.91,608.36) | 834.55(703.55,991.26) | 1761.05(1483.63,2148.94) | 1667.13(1361.27,2076.19) | 1910.85(1633.96,2339.36) | 842.8(636.13,1065.09) | 704.88(537.28,904.7) | 1003.65(748.3,1310.96) | 997.66(763.88,1287.41) | 885.89(672.13,1139.55) | 1139.83(862.6,1509.54) | 1217.07(1015.51,1428.21) | 1021.96(828.15,1212.11) | 1475.3(1243.69,1754.84) |
| 85-89 years | Number95%(UI) | 713237.27(584514.39,853669.94) | 379392.73(294726.76,466565.44) | 333844.54(285098.43,396849.31) | 162375.88(126003.64,197086.17) | 83906.91(61516.68,103009.22) | 78468.97(64485.68,93674.91) | 276086.15(223839.26,338295.57) | 163153.89(129483.9,205265.46) | 112932.25(96050.03,134444.47) | 11724.26(8914.77,14731.76) | 5144.62(3778.36,6491.87) | 6579.65(5063.53,8413.83) | 53268.53(39909.63,68618.61) | 26009.31(19431.12,32829.16) | 27259.22(20570.64,35772.28) | 208970.77(172019.01,245684.37) | 100673.24(77850.66,121804.09) | 108297.53(92041.17,129550.91) |
|  | Rate95%(UI) | 1559.95(1278.42,1867.1) | 1332.65(1035.25,1638.85) | 1935.03(1652.49,2300.22) | 984.21(763.75,1194.6) | 812.72(595.85,997.75) | 1270.99(1044.49,1517.28) | 2426.66(1967.44,2973.45) | 2190.77(1738.66,2756.23) | 2873.68(2444.1,3421.09) | 878.25(667.79,1103.53) | 691.3(507.71,872.33) | 1113.74(857.11,1424.22) | 1066(798.66,1373.18) | 906.67(677.36,1144.41) | 1280.75(966.49,1680.72) | 1822.22(1500,2142.36) | 1426.98(1103.49,1726.5) | 2454.09(2085.71,2935.71) |
| 90-94 years | Number95%(UI) | 392582.28(307390.96,479003.06) | 238825.58(180300.46,297063.92) | 153756.71(126787.26,183649.92) | 120047.92(90022.98,147128.63) | 73693.14(52801.05,91029.49) | 46354.78(37435.44,55747.1) | 152806.44(121801.36,188071.72) | 101661.94(79067.93,127782.02) | 51144.5(42742.3,61733.61) | 3769.55(2828,4753.08) | 1676.31(1234.85,2147.05) | 2093.24(1565.17,2668.47) | 21666.25(15947.14,28126.46) | 10352.99(7392.94,13428.66) | 11313.26(8478.71,15106.91) | 93864.96(75073.3,110377.15) | 51152.63(38561.38,61817.12) | 42712.34(35424.77,50671.16) |
|  | Rate95%(UI) | 2194.5(1718.29,2677.58) | 1980.17(1494.92,2463.04) | 2638.02(2175.3,3150.9) | 1565.85(1174.22,1919.08) | 1402.76(1005.08,1732.76) | 1920.9(1551.29,2310.1) | 3495.29(2786.08,4301.95) | 3281.76(2552.4,4124.95) | 4014.51(3354.99,4845.68) | 1051.59(788.92,1325.96) | 821.93(605.47,1052.74) | 1354.73(1012.96,1727.01) | 1338.89(985.47,1738.1) | 1092.94(780.46,1417.63) | 1686.1(1263.65,2251.5) | 2434.65(1947.24,2862.94) | 2009.34(1514.74,2428.25) | 3261.4(2704.94,3869.12) |
| 95+ years | Number95%(UI) | 149046.41(107148.64,183205.81) | 107628.94(76145.69,132721.18) | 41417.47(31579.68,50355.93) | 63225.8(44102.91,78499.45) | 45913.97(31336.47,57184.57) | 17311.83(12720.65,21239.26) | 49229.8(36093.02,61065.06) | 37440.44(27193.25,46595.54) | 11789.35(9253.17,14332.25) | 913.02(670.62,1176.06) | 492.19(344.92,631.88) | 420.83(307.03,542.34) | 7084.97(4955.09,9329.43) | 3701.17(2542.32,4909.25) | 3383.8(2418.58,4511.68) | 28424.08(21022.19,34516.84) | 19956.42(14463.09,24760.75) | 8467.66(6539.18,10156.41) |
|  | Rate95%(UI) | 2734.64(1965.92,3361.38) | 2732.9(1933.48,3370.04) | 2739.17(2088.55,3330.33) | 2282.31(1592.02,2833.66) | 2227.68(1520.4,2774.51) | 2441.1(1793.71,2994.89) | 4459.76(3269.69,5531.92) | 4525.55(3286.94,5632.15) | 4262.95(3345.89,5182.45) | 1200.08(881.47,1545.82) | 1014.74(711.12,1302.75) | 1526.07(1113.39,1966.72) | 1562.33(1092.67,2057.27) | 1275.56(876.18,1691.91) | 2071.81(1480.84,2762.39) | 2732.11(2020.65,3317.75) | 2823.34(2046.17,3503.04) | 2538.77(1960.58,3045.09) |
| DALYs: Disability-adjusted life years. ASDR: age-standardized DALY rate. SDI: Sociodemographic Index. | | | | | | | | | | | | | | | | | | | |

| **Table S7. Age-specific death numbers and ASMR for myocardial disease attributable to high temperature, categorized by 5-year age groups, presented globally and**  **by SDI region in 2021.** | | | | | | | | | | | | | | | | | | | |
| --- | --- | --- | --- | --- | --- | --- | --- | --- | --- | --- | --- | --- | --- | --- | --- | --- | --- | --- | --- |
|  |  | Global | | | High SDI | | | High-middle SDI | | | Low SDI | | | Low-middle SDI | | | Middle SDI | | |
| age | metric | Both | Female | Male | Both | Female | Male | Both | Female | Male | Both | Female | Male | Both | Female | Male | Both | Female | Male |
| <5 years | Number95%(UI) | 254.24(-75.75,638.15) | 126.38(-29.98,313.08) | 127.86(-45.24,331.36) | 6.64(-0.41,14.07) | 2.7(-0.17,5.89) | 3.94(-0.24,8.59) | 8.55(-1.21,18.62) | 3.3(-0.56,7.25) | 5.25(-0.7,11.78) | 34.38(-57.07,152.71) | 17.72(-25.42,74.29) | 16.67(-31.74,80.43) | 152.91(-26.69,362.85) | 79.27(-9.55,190.23) | 73.64(-14.01,182.72) | 51.74(0.47,102.79) | 23.39(0.45,44.71) | 28.35(-0.04,58.65) |
|  | Rate95%(UI) | 0.04(-0.01,0.1) | 0.04(-0.01,0.1) | 0.04(-0.01,0.1) | 0.01(0,0.03) | 0.01(0,0.02) | 0.01(0,0.03) | 0.01(0,0.03) | 0.01(0,0.02) | 0.01(0,0.03) | 0.02(-0.03,0.09) | 0.02(-0.03,0.09) | 0.02(-0.04,0.1) | 0.08(-0.01,0.19) | 0.09(-0.01,0.2) | 0.07(-0.01,0.19) | 0.03(0,0.06) | 0.03(0,0.05) | 0.03(0,0.06) |
| 5-9 years | Number95%(UI) | 34.04(-13.82,84.28) | 16.38(-3.44,40.46) | 17.66(-9.96,50.42) | 1.51(-0.1,3.3) | 0.71(-0.05,1.55) | 0.8(-0.05,1.8) | 2.26(-0.32,5.07) | 0.88(-0.15,1.96) | 1.38(-0.19,3.18) | 3.37(-8.38,19.37) | 1.52(-2.14,6.65) | 1.85(-6.32,13.56) | 17.45(-4.9,44.47) | 8.94(-1.54,21.37) | 8.51(-3.4,23.32) | 9.45(-0.45,20.14) | 4.34(-0.02,9.05) | 5.11(-0.44,11.28) |
|  | Rate95%(UI) | 0(0,0.01) | 0(0,0.01) | 0(0,0.01) | 0(0,0.01) | 0(0,0.01) | 0(0,0.01) | 0(0,0.01) | 0(0,0.01) | 0(0,0.01) | 0(-0.01,0.01) | 0(0,0.01) | 0(-0.01,0.02) | 0.01(0,0.02) | 0.01(0,0.02) | 0.01(0,0.02) | 0(0,0.01) | 0(0,0.01) | 0(0,0.01) |
| 10-14 years | Number95%(UI) | 35.38(-9.58,81.75) | 16.97(-5.08,42.84) | 18.4(-4.79,44.13) | 1.95(-0.14,4.32) | 0.87(-0.07,1.92) | 1.08(-0.07,2.41) | 2.43(-0.52,5.7) | 0.97(-0.19,2.32) | 1.45(-0.32,3.48) | 3.72(-4.28,14.69) | 2.14(-2.74,9.11) | 1.58(-1.59,6.17) | 17.6(-3.88,41.78) | 9.03(-1.86,22.92) | 8.57(-2.16,21.55) | 9.67(-0.97,20.72) | 3.96(-0.47,8.78) | 5.71(-0.46,12.3) |
|  | Rate95%(UI) | 0.01(0,0.01) | 0.01(0,0.01) | 0.01(0,0.01) | 0(0,0.01) | 0(0,0.01) | 0(0,0.01) | 0(0,0.01) | 0(0,0.01) | 0(0,0.01) | 0(0,0.01) | 0(0,0.01) | 0(0,0.01) | 0.01(0,0.02) | 0.01(0,0.02) | 0.01(0,0.02) | 0.01(0,0.01) | 0(0,0.01) | 0.01(0,0.01) |
| 15-19 years | Number95%(UI) | 44.59(-17.58,113.06) | 17.31(-3.35,40.96) | 27.28(-14.19,76.41) | 3.67(-0.31,8.12) | 1.07(-0.08,2.41) | 2.59(-0.23,5.75) | 3.43(-1.01,8.12) | 1.03(-0.33,2.53) | 2.4(-0.7,5.85) | 5.27(-8.03,23.06) | 2.23(-1.07,6.59) | 3.04(-6.89,16.81) | 20.57(-6.67,52.41) | 9.04(-1.17,22.88) | 11.53(-5.34,31.47) | 11.65(-2.12,25.15) | 3.94(-0.53,8.21) | 7.7(-1.67,17.43) |
|  | Rate95%(UI) | 0.01(0,0.02) | 0.01(0,0.01) | 0.01(0,0.02) | 0.01(0,0.01) | 0(0,0.01) | 0.01(0,0.02) | 0(0,0.01) | 0(0,0.01) | 0.01(0,0.02) | 0(-0.01,0.02) | 0(0,0.01) | 0(-0.01,0.03) | 0.01(0,0.03) | 0.01(0,0.03) | 0.01(-0.01,0.03) | 0.01(0,0.01) | 0(0,0.01) | 0.01(0,0.02) |
| 20-24 years | Number95%(UI) | 61.55(-23.24,152.76) | 24.35(-8.13,59.55) | 37.2(-14.42,93.94) | 6.48(-0.59,15.08) | 1.93(-0.14,4.34) | 4.56(-0.44,10.71) | 5.37(-2.48,13.69) | 1.54(-0.6,3.74) | 3.84(-1.88,9.95) | 7.05(-8.22,26.3) | 3.35(-3.26,12.59) | 3.7(-4.64,14.63) | 28.36(-8.3,67.66) | 12.58(-2.75,33.59) | 15.78(-5.41,40.19) | 14.29(-3.83,32.83) | 4.96(-1.29,11.46) | 9.33(-2.5,21.61) |
|  | Rate95%(UI) | 0.01(0,0.03) | 0.01(0,0.02) | 0.01(0,0.03) | 0.01(0,0.02) | 0.01(0,0.01) | 0.01(0,0.03) | 0.01(0,0.02) | 0(0,0.01) | 0.01(0,0.03) | 0.01(-0.01,0.03) | 0.01(-0.01,0.02) | 0.01(-0.01,0.03) | 0.02(0,0.04) | 0.01(0,0.04) | 0.02(-0.01,0.05) | 0.01(0,0.02) | 0.01(0,0.01) | 0.01(0,0.02) |
| 25-29 years | Number95%(UI) | 78.15(-41.76,211.05) | 28.63(-13.46,77.5) | 49.52(-27.98,138.04) | 9.47(-0.77,20.57) | 2.88(-0.19,6.6) | 6.59(-0.58,14.47) | 8.43(-6.41,23.29) | 2.04(-1.33,5.53) | 6.39(-5.03,18.3) | 8.52(-14.08,39.09) | 3.56(-5.58,16.37) | 4.96(-8.78,23.43) | 34.67(-13.73,89.91) | 14.22(-4.96,39.43) | 20.45(-8.62,55.84) | 17.07(-6.33,42.2) | 5.93(-2.01,14.42) | 11.14(-4.52,28.4) |
|  | Rate95%(UI) | 0.01(-0.01,0.04) | 0.01(0,0.03) | 0.02(-0.01,0.05) | 0.01(0,0.03) | 0.01(0,0.02) | 0.02(0,0.04) | 0.01(-0.01,0.03) | 0.01(0,0.01) | 0.01(-0.01,0.04) | 0.01(-0.02,0.05) | 0.01(-0.01,0.04) | 0.01(-0.02,0.06) | 0.02(-0.01,0.06) | 0.02(-0.01,0.05) | 0.03(-0.01,0.07) | 0.01(0,0.02) | 0.01(0,0.02) | 0.01(0,0.03) |
| 30-34 years | Number95%(UI) | 114.22(-56.53,283.03) | 33.45(-13.96,86.91) | 80.77(-41.77,204.05) | 12.96(-1.22,28.68) | 4.02(-0.34,9.01) | 8.93(-0.9,20.9) | 18.12(-17.55,56.36) | 3.82(-3.62,11.95) | 14.29(-13.94,45.29) | 11.51(-11.27,39.61) | 3.95(-3.27,13.62) | 7.56(-8.22,28.66) | 45.85(-15.15,109.02) | 14.49(-4.1,40.34) | 31.36(-10.92,77.36) | 25.78(-8.73,57.84) | 7.16(-2.59,16.66) | 18.63(-6.42,43.06) |
|  | Rate95%(UI) | 0.02(-0.01,0.05) | 0.01(0,0.03) | 0.03(-0.01,0.07) | 0.02(0,0.04) | 0.01(0,0.02) | 0.02(0,0.05) | 0.02(-0.02,0.05) | 0.01(-0.01,0.02) | 0.03(-0.03,0.08) | 0.02(-0.02,0.05) | 0.01(-0.01,0.04) | 0.02(-0.02,0.08) | 0.03(-0.01,0.07) | 0.02(-0.01,0.05) | 0.04(-0.01,0.1) | 0.01(0,0.03) | 0.01(0,0.02) | 0.02(-0.01,0.04) |
| 35-39 years | Number95%(UI) | 151.89(-76.93,386.66) | 43.6(-19.77,106.99) | 108.29(-55.28,282.68) | 19.33(-1.73,44.69) | 5.75(-0.35,13.26) | 13.58(-1.4,32.36) | 28.17(-31.25,92.42) | 6(-6.56,19.64) | 22.17(-24.75,72.4) | 14.09(-10.43,44.83) | 4.72(-3.73,15.88) | 9.36(-6.85,29.05) | 54.36(-18.45,131.57) | 17.15(-5.39,45.44) | 37.21(-12.75,94.18) | 35.95(-9.88,79.9) | 9.97(-2.6,22.33) | 25.97(-7.51,58.62) |
|  | Rate95%(UI) | 0.03(-0.01,0.07) | 0.02(-0.01,0.04) | 0.04(-0.02,0.1) | 0.02(0,0.06) | 0.02(0,0.03) | 0.03(0,0.08) | 0.03(-0.03,0.09) | 0.01(-0.01,0.04) | 0.04(-0.05,0.14) | 0.02(-0.02,0.07) | 0.01(-0.01,0.05) | 0.03(-0.02,0.1) | 0.04(-0.01,0.1) | 0.03(-0.01,0.07) | 0.06(-0.02,0.14) | 0.02(-0.01,0.04) | 0.01(0,0.02) | 0.03(-0.01,0.06) |
| 40-44 years | Number95%(UI) | 211.37(-105.8,546.3) | 52.37(-25.3,130.12) | 159(-78.02,399.91) | 27.06(-2.58,60.56) | 7.54(-0.55,17.23) | 19.52(-2.07,44.66) | 37.72(-41.31,123) | 7.81(-8.89,25.98) | 29.91(-32.05,97.44) | 21.39(-15.92,66.86) | 5.08(-4.75,17.52) | 16.31(-11.05,50.67) | 78.28(-23.91,192.29) | 18.94(-7.03,50.72) | 59.34(-16.93,151.2) | 46.91(-12.58,109.62) | 13(-2.92,28.01) | 33.91(-9.69,79.2) |
|  | Rate95%(UI) | 0.04(-0.02,0.11) | 0.02(-0.01,0.05) | 0.06(-0.03,0.16) | 0.04(0,0.08) | 0.02(0,0.05) | 0.05(-0.01,0.11) | 0.04(-0.04,0.13) | 0.02(-0.02,0.06) | 0.06(-0.07,0.21) | 0.04(-0.03,0.13) | 0.02(-0.02,0.07) | 0.06(-0.04,0.2) | 0.07(-0.02,0.17) | 0.03(-0.01,0.09) | 0.1(-0.03,0.26) | 0.03(-0.01,0.07) | 0.02(0,0.03) | 0.04(-0.01,0.1) |
| 45-49 years | Number95%(UI) | 257.09(-124.22,660.97) | 68.48(-29.38,166.97) | 188.61(-92.78,495.63) | 33.81(-2.9,73.34) | 9.11(-0.6,19.26) | 24.69(-2.3,54.23) | 44.19(-45.98,145.19) | 9.36(-9.96,31.15) | 34.84(-35.79,114.29) | 24.01(-18.79,76.96) | 6.66(-4.94,20.68) | 17.35(-13.47,58.53) | 93.77(-32.12,242.03) | 25.61(-8.09,67.25) | 68.16(-24.51,174.35) | 61.28(-15.09,131.03) | 17.74(-3.82,39.55) | 43.54(-10.86,97.08) |
|  | Rate95%(UI) | 0.05(-0.03,0.14) | 0.03(-0.01,0.07) | 0.08(-0.04,0.21) | 0.05(0,0.1) | 0.03(0,0.05) | 0.07(-0.01,0.14) | 0.05(-0.05,0.15) | 0.02(-0.02,0.06) | 0.07(-0.07,0.23) | 0.06(-0.05,0.18) | 0.03(-0.02,0.1) | 0.08(-0.06,0.28) | 0.1(-0.03,0.25) | 0.05(-0.02,0.14) | 0.14(-0.05,0.36) | 0.04(-0.01,0.08) | 0.02(0,0.05) | 0.05(-0.01,0.12) |
| 50-54 years | Number95%(UI) | 309.2(-132.54,780.35) | 79.2(-30.01,186.36) | 230(-100.5,596.77) | 41.16(-4.29,91.72) | 11.33(-0.93,24.6) | 29.84(-3.4,67.16) | 53.16(-50.76,168.01) | 11.27(-10.8,36.24) | 41.89(-39.53,131.15) | 26.5(-18.23,79.98) | 6.33(-4.52,20.11) | 20.17(-14.16,62.54) | 110.3(-38.8,287.12) | 29.13(-9,76.25) | 81.17(-28.39,212.36) | 78.01(-15.7,173.4) | 21.12(-3.46,47.43) | 56.88(-11.67,128.94) |
|  | Rate95%(UI) | 0.07(-0.03,0.18) | 0.04(-0.01,0.08) | 0.1(-0.05,0.27) | 0.06(-0.01,0.12) | 0.03(0,0.07) | 0.08(-0.01,0.18) | 0.05(-0.05,0.17) | 0.02(-0.02,0.07) | 0.09(-0.08,0.27) | 0.08(-0.06,0.24) | 0.04(-0.03,0.12) | 0.12(-0.09,0.38) | 0.13(-0.05,0.34) | 0.07(-0.02,0.18) | 0.19(-0.07,0.51) | 0.05(-0.01,0.11) | 0.03(0,0.06) | 0.07(-0.01,0.17) |
| 55-59 years | Number95%(UI) | 407.15(-205.9,1019.45) | 123.79(-51.15,299.09) | 283.36(-150.48,718.83) | 50.76(-6.47,111.26) | 14.4(-1.38,31.3) | 36.36(-5.11,80.97) | 65.39(-57.01,200.25) | 16.91(-14.4,52.84) | 48.48(-41.88,146.65) | 36.29(-45.78,133.54) | 12.73(-9.67,42.7) | 23.56(-35.73,95.1) | 150.35(-66.61,377.16) | 45.83(-17.86,123.44) | 104.52(-47.41,270.83) | 104.29(-21.42,226.08) | 33.91(-6.55,72.53) | 70.38(-15.34,156.04) |
|  | Rate95%(UI) | 0.1(-0.05,0.26) | 0.06(-0.03,0.15) | 0.15(-0.08,0.37) | 0.07(-0.01,0.15) | 0.04(0,0.09) | 0.1(-0.01,0.22) | 0.07(-0.06,0.22) | 0.04(-0.03,0.12) | 0.11(-0.1,0.33) | 0.14(-0.18,0.53) | 0.1(-0.08,0.34) | 0.19(-0.28,0.76) | 0.22(-0.1,0.54) | 0.13(-0.05,0.35) | 0.31(-0.14,0.79) | 0.08(-0.02,0.16) | 0.05(-0.01,0.1) | 0.1(-0.02,0.23) |
| 60-64 years | Number95%(UI) | 434.99(-202.28,1089.28) | 137.81(-55.23,345.3) | 297.18(-144.47,766.56) | 57.52(-8.01,131.18) | 16.94(-2.35,38.21) | 40.57(-5.68,92.93) | 66.25(-58.18,203.06) | 17.86(-14.51,54) | 48.39(-43.64,149.44) | 42.16(-38.01,144.44) | 14.99(-10.73,47.48) | 27.17(-27.63,100.06) | 163.22(-66.78,419.93) | 52.76(-18.66,140.27) | 110.46(-47.89,297.67) | 105.74(-26.05,233.76) | 35.23(-8.14,76.59) | 70.51(-18.66,160.63) |
|  | Rate95%(UI) | 0.14(-0.06,0.34) | 0.08(-0.03,0.21) | 0.19(-0.09,0.49) | 0.08(-0.01,0.19) | 0.05(-0.01,0.11) | 0.12(-0.02,0.28) | 0.09(-0.08,0.28) | 0.05(-0.04,0.14) | 0.14(-0.12,0.43) | 0.21(-0.19,0.73) | 0.15(-0.11,0.48) | 0.28(-0.28,1.03) | 0.29(-0.12,0.73) | 0.18(-0.06,0.48) | 0.4(-0.17,1.07) | 0.1(-0.03,0.23) | 0.07(-0.02,0.15) | 0.14(-0.04,0.32) |
| 65-69 years | Number95%(UI) | 479.72(-192.64,1175.45) | 163.63(-54.68,386.64) | 316.09(-134.26,789.3) | 65.31(-10,148.92) | 19.51(-2.75,44.46) | 45.81(-7.27,104.68) | 66.46(-39.29,184.25) | 20.87(-12.78,58.64) | 45.59(-26.76,127.38) | 47.23(-36.9,150.67) | 17.27(-7.07,47.04) | 29.97(-28.87,107.85) | 180.07(-68.56,470.48) | 62.01(-18.17,161.73) | 118.05(-48.82,316.89) | 120.51(-30.82,269.28) | 43.94(-10.2,99.35) | 76.57(-19.98,178.35) |
|  | Rate95%(UI) | 0.17(-0.07,0.43) | 0.11(-0.04,0.27) | 0.24(-0.1,0.6) | 0.11(-0.02,0.25) | 0.06(-0.01,0.14) | 0.16(-0.03,0.36) | 0.1(-0.06,0.28) | 0.06(-0.04,0.17) | 0.15(-0.09,0.41) | 0.31(-0.24,1) | 0.22(-0.09,0.61) | 0.41(-0.39,1.46) | 0.41(-0.15,1.06) | 0.27(-0.08,0.7) | 0.55(-0.23,1.48) | 0.14(-0.03,0.3) | 0.1(-0.02,0.22) | 0.18(-0.05,0.41) |
| 70-74 years | Number95%(UI) | 491.78(-173.87,1198.77) | 187.41(-60.2,442.43) | 304.37(-111.76,750.53) | 74.19(-12.06,166) | 25.19(-3.96,56.63) | 49(-8.19,109.83) | 63.01(-26.69,163.67) | 23.44(-10.28,63.45) | 39.57(-16.94,101.46) | 55.99(-31.42,166.3) | 22.97(-9.89,64.05) | 33.02(-21.39,104.02) | 183.27(-72.48,469.88) | 70.44(-24.5,183.71) | 112.82(-46.54,294.22) | 115.17(-30.37,250.46) | 45.31(-11.61,101.32) | 69.86(-18.24,156.43) |
|  | Rate95%(UI) | 0.24(-0.08,0.58) | 0.17(-0.06,0.4) | 0.32(-0.12,0.78) | 0.14(-0.02,0.31) | 0.09(-0.01,0.2) | 0.2(-0.03,0.44) | 0.13(-0.05,0.34) | 0.09(-0.04,0.24) | 0.18(-0.08,0.46) | 0.54(-0.3,1.6) | 0.43(-0.19,1.21) | 0.65(-0.42,2.05) | 0.58(-0.23,1.49) | 0.43(-0.15,1.12) | 0.75(-0.31,1.97) | 0.19(-0.05,0.41) | 0.14(-0.04,0.31) | 0.24(-0.06,0.53) |
| 75-79 years | Number95%(UI) | 488.62(-172.68,1179.68) | 202.24(-70.34,497.56) | 286.38(-99.71,722.74) | 71.12(-11.45,162.15) | 26.65(-4.43,61.22) | 44.47(-7.04,102.88) | 64.22(-19.01,156.6) | 26.94(-9.54,70.3) | 37.28(-10.08,88.88) | 50.86(-34.75,161.25) | 22.97(-13.35,74.51) | 27.9(-21.36,92.53) | 181.14(-73.34,462.12) | 75.37(-27.68,196.46) | 105.77(-43.39,284.76) | 121.1(-27.34,269.24) | 50.24(-12.56,117.33) | 70.86(-15.91,162.12) |
|  | Rate95%(UI) | 0.37(-0.13,0.89) | 0.28(-0.1,0.69) | 0.48(-0.17,1.21) | 0.2(-0.03,0.45) | 0.13(-0.02,0.31) | 0.27(-0.04,0.63) | 0.22(-0.06,0.52) | 0.16(-0.06,0.42) | 0.29(-0.08,0.69) | 0.79(-0.54,2.51) | 0.69(-0.4,2.24) | 0.9(-0.69,2.99) | 0.9(-0.37,2.3) | 0.7(-0.26,1.83) | 1.13(-0.47,3.05) | 0.31(-0.07,0.69) | 0.24(-0.06,0.55) | 0.39(-0.09,0.9) |
| 80-84 years | Number95%(UI) | 518.91(-166.68,1248.64) | 239.54(-82.3,578.67) | 279.37(-83.69,700.94) | 76.22(-15.04,173.69) | 31.78(-6.86,74.34) | 44.43(-8.41,102.32) | 87.48(-35.41,236.36) | 46.54(-23.25,133.19) | 40.94(-12.93,103.74) | 46.48(-23.56,133.73) | 21.65(-9.01,61.69) | 24.83(-14.4,81.31) | 171.63(-65.92,434.35) | 73.68(-28.23,197.07) | 97.95(-36.58,265.05) | 136.86(-31.84,317.49) | 65.76(-17.75,158.92) | 71.1(-14.64,162) |
|  | Rate95%(UI) | 0.59(-0.19,1.43) | 0.47(-0.16,1.14) | 0.76(-0.23,1.91) | 0.28(-0.06,0.64) | 0.2(-0.04,0.48) | 0.39(-0.07,0.9) | 0.4(-0.16,1.08) | 0.35(-0.17,0.99) | 0.49(-0.15,1.23) | 1.38(-0.7,3.96) | 1.19(-0.5,3.4) | 1.59(-0.92,5.22) | 1.49(-0.57,3.78) | 1.14(-0.44,3.06) | 1.94(-0.72,5.24) | 0.57(-0.13,1.33) | 0.48(-0.13,1.17) | 0.69(-0.14,1.58) |
| 85-89 years | Number95%(UI) | 454.46(-131.03,1098.86) | 221.43(-66.49,523.03) | 233.03(-68.54,555.44) | 79.86(-16.7,185.86) | 39.41(-8.48,95.05) | 40.45(-8.54,93.66) | 92.03(-28.66,237.56) | 50.58(-20.33,138.07) | 41.46(-8.84,101.29) | 27.52(-17.39,85.36) | 13.1(-4.35,37.89) | 14.41(-12.85,52.27) | 119.87(-44.27,314.88) | 52.04(-19.61,148.15) | 67.83(-24.74,184.44) | 134.95(-27.54,295.59) | 66.17(-16.07,154.44) | 68.79(-12.06,152.06) |
|  | Rate95%(UI) | 0.99(-0.29,2.4) | 0.78(-0.23,1.84) | 1.35(-0.4,3.22) | 0.48(-0.1,1.13) | 0.38(-0.08,0.92) | 0.66(-0.14,1.52) | 0.81(-0.25,2.09) | 0.68(-0.27,1.85) | 1.05(-0.22,2.58) | 2.06(-1.3,6.39) | 1.76(-0.58,5.09) | 2.44(-2.18,8.85) | 2.4(-0.89,6.3) | 1.81(-0.68,5.16) | 3.19(-1.16,8.67) | 1.18(-0.24,2.58) | 0.94(-0.23,2.19) | 1.56(-0.27,3.45) |
| 90-94 years | Number95%(UI) | 274.33(-80.16,671.5) | 150.33(-50.34,379.79) | 124(-29.89,294.51) | 65.37(-13.98,156.28) | 38.07(-7.88,93.06) | 27.3(-6.11,63.94) | 62.92(-17.72,157.67) | 38.1(-14.11,102.64) | 24.82(-3.98,57.56) | 8.49(-7.35,30.22) | 4.12(-3.71,15.65) | 4.36(-3.56,15.38) | 58.25(-23.02,149.86) | 25.25(-13.74,73.57) | 33(-9.87,87.83) | 79.18(-21.16,193.15) | 44.71(-14.52,114.27) | 34.47(-6.64,77.99) |
|  | Rate95%(UI) | 1.53(-0.45,3.75) | 1.25(-0.42,3.15) | 2.13(-0.51,5.05) | 0.85(-0.18,2.04) | 0.72(-0.15,1.77) | 1.13(-0.25,2.65) | 1.44(-0.41,3.61) | 1.23(-0.46,3.31) | 1.95(-0.31,4.52) | 2.37(-2.05,8.43) | 2.02(-1.82,7.67) | 2.82(-2.3,9.95) | 3.6(-1.42,9.26) | 2.67(-1.45,7.77) | 4.92(-1.47,13.09) | 2.05(-0.55,5.01) | 1.76(-0.57,4.49) | 2.63(-0.51,5.96) |
| 95+ years | Number95%(UI) | 122.56(-33.05,300.5) | 83.32(-24.46,206.02) | 39.24(-8.9,95.68) | 38.98(-7.82,96.26) | 26.62(-5.22,66.24) | 12.35(-2.6,29.33) | 26.15(-7.53,67.15) | 19.77(-5.7,51.17) | 6.37(-2.03,16.93) | 2.04(-1.87,7.31) | 1.15(-1.4,4.71) | 0.89(-0.51,2.82) | 20.19(-7.72,52.63) | 8.59(-5.43,28.61) | 11.6(-2.54,31.03) | 35.16(-10.4,91.03) | 27.16(-8.17,70.4) | 8(-2.41,20.19) |
|  | Rate95%(UI) | 2.25(-0.61,5.51) | 2.12(-0.62,5.23) | 2.59(-0.59,6.33) | 1.41(-0.28,3.47) | 1.29(-0.25,3.21) | 1.74(-0.37,4.14) | 2.37(-0.68,6.08) | 2.39(-0.69,6.19) | 2.3(-0.74,6.12) | 2.68(-2.46,9.6) | 2.37(-2.89,9.71) | 3.22(-1.83,10.24) | 4.45(-1.7,11.61) | 2.96(-1.87,9.86) | 7.11(-1.56,19) | 3.38(-1,8.75) | 3.84(-1.16,9.96) | 2.4(-0.72,6.05) |
| ASMR: Age-standardized mortality rates. SDI: Sociodemographic Index. | | | | | | | | | | | | | | | | | | | |

| **Table S8. Age-specific death numbers and ASMR for myocardial disease attributable to low temperature, categorized by 5-year age groups, presented globally and by SDI region in 2021.** | | | | | | | | | | | | | | | | | | | | | | | | | | | | | | | | | | | | | | |  |  |
| --- | --- | --- | --- | --- | --- | --- | --- | --- | --- | --- | --- | --- | --- | --- | --- | --- | --- | --- | --- | --- | --- | --- | --- | --- | --- | --- | --- | --- | --- | --- | --- | --- | --- | --- | --- | --- | --- | --- | --- | --- |
|  | |  | | Global | | | | | | High SDI | | | | | | High-middle SDI | | | | | | Low SDI | | | | | | Low-middle SDI | | | | | Middle SDI | | | | | |  |  |
| age | | metric | | Both | | Female | | Male | | Both | | Female | | Male | | Both | | Female | | Male | | Both | | Female | | Male | | Both | | Female | | Male | Both | | Female | | Male | |  |  |
| <5 years | | Number95%(UI) | | 386.3(213.91,626.5) | | 184.51(94.92,311.52) | | 201.79(112.64,332.04) | | 21.33(16.9,26.22) | | 9.5(7.5,11.38) | | 11.83(9.2,14.87) | | 25.7(16.98,33.64) | | 10.59(6.93,13.85) | | 15.12(9.98,20.35) | | 92.39(43.13,157.87) | | 45.75(18.99,82.05) | | 46.64(20.23,78.99) | | 164.73(53.76,306.81) | | 83.81(25.91,162.99) | | 80.92(28.76,147.06) | 81.95(54.54,118.9) | | 34.75(23.01,50.73) | | 47.19(30.56,69.33) | |  |  |
|  |  | Rate95%(UI) | | 0.06(0.03,0.1) | | 0.06(0.03,0.1) | | 0.06(0.03,0.1) | | 0.04(0.03,0.05) | | 0.04(0.03,0.04) | | 0.04(0.03,0.05) | | 0.04(0.02,0.05) | | 0.03(0.02,0.04) | | 0.04(0.03,0.06) | | 0.06(0.03,0.1) | | 0.06(0.02,0.1) | | 0.06(0.02,0.09) | | 0.09(0.03,0.16) | | 0.09(0.03,0.18) | | 0.08(0.03,0.15) | 0.05(0.03,0.07) | | 0.04(0.03,0.06) | | 0.05(0.03,0.08) | |  |  |
| 5-9 years | | Number95%(UI) | | 57.88(37.52,88.64) | | 25.5(15.5,40.03) | | 32.38(20.92,48.59) | | 4.63(3.7,5.52) | | 2.22(1.73,2.67) | | 2.41(1.91,2.94) | | 6.6(3.99,8.56) | | 2.67(1.72,3.57) | | 3.93(2.29,5.37) | | 9.83(4.58,17.78) | | 3.86(1.61,7.35) | | 5.97(2.42,11.28) | | 18.3(7.48,34.24) | | 8.84(2.92,17.5) | | 9.46(3.69,18.61) | 18.49(13.01,25.85) | | 7.9(5.26,11.38) | | 10.59(7.1,14.95) | |  |  |
|  |  | Rate95%(UI) | | 0.01(0.01,0.01) | | 0.01(0,0.01) | | 0.01(0.01,0.01) | | 0.01(0.01,0.01) | | 0.01(0.01,0.01) | | 0.01(0.01,0.01) | | 0.01(0,0.01) | | 0.01(0,0.01) | | 0.01(0.01,0.01) | | 0.01(0,0.01) | | 0.01(0,0.01) | | 0.01(0,0.01) | | 0.01(0,0.02) | | 0.01(0,0.02) | | 0.01(0,0.02) | 0.01(0.01,0.01) | | 0.01(0.01,0.01) | | 0.01(0.01,0.01) | |  |  |
| 10-14 years | | Number95%(UI) | | 63.27(41.56,91.44) | | 29.3(17.58,45.85) | | 33.97(22.6,49) | | 6.08(4.88,7.15) | | 2.56(2.04,3.07) | | 3.52(2.76,4.23) | | 8.72(5.86,11.04) | | 3.32(2.11,4.32) | | 5.4(3.55,6.96) | | 8.96(4.45,15.53) | | 4.94(2.12,9.63) | | 4.02(1.88,7.37) | | 19.4(7.5,33.83) | | 10.24(3.42,20.39) | | 9.17(3.64,17.06) | 20.07(14.2,27.63) | | 8.23(5.59,11.76) | | 11.84(8.17,16.49) | |  |  |
|  |  | Rate95%(UI) | | 0.01(0.01,0.01) | | 0.01(0.01,0.01) | | 0.01(0.01,0.01) | | 0.01(0.01,0.01) | | 0.01(0.01,0.01) | | 0.01(0.01,0.01) | | 0.01(0.01,0.01) | | 0.01(0.01,0.01) | | 0.01(0.01,0.02) | | 0.01(0,0.01) | | 0.01(0,0.01) | | 0.01(0,0.01) | | 0.01(0,0.02) | | 0.01(0,0.02) | | 0.01(0,0.02) | 0.01(0.01,0.01) | | 0.01(0.01,0.01) | | 0.01(0.01,0.02) | |  |  |
| 15-19 years | | Number95%(UI) | | 98.04(66.82,142.75) | | 32.2(19.95,49.79) | | 65.83(43.52,97) | | 13.54(10.95,15.98) | | 3.61(2.84,4.3) | | 9.93(8.02,11.79) | | 17.44(12.48,21.99) | | 4.92(3.34,6.21) | | 12.52(9,16.06) | | 13.3(6.5,23.99) | | 4.57(1.99,8.13) | | 8.73(3.93,16.36) | | 27.78(11.06,49.87) | | 10.97(3.89,22.2) | | 16.82(6.47,31.26) | 25.92(17.91,35.59) | | 8.13(5.54,11.23) | | 17.79(12.08,24.92) | |  |  |
|  |  | Rate95%(UI) | | 0.02(0.01,0.02) | | 0.01(0.01,0.02) | | 0.02(0.01,0.03) | | 0.02(0.02,0.03) | | 0.01(0.01,0.01) | | 0.03(0.03,0.04) | | 0.02(0.02,0.03) | | 0.01(0.01,0.02) | | 0.03(0.02,0.04) | | 0.01(0.01,0.02) | | 0.01(0,0.01) | | 0.01(0.01,0.03) | | 0.02(0.01,0.03) | | 0.01(0,0.02) | | 0.02(0.01,0.03) | 0.01(0.01,0.02) | | 0.01(0.01,0.01) | | 0.02(0.01,0.03) | |  |  |
| 20-24 years | | Number95%(UI) | | 145.14(101.1,205.26) | | 48.59(30.41,74.07) | | 96.55(68.94,132.94) | | 22.27(17.68,26.5) | | 6.01(4.74,7.27) | | 16.25(12.89,19.27) | | 33.21(23.8,40.92) | | 8.34(5.81,10.66) | | 24.87(17.46,31.32) | | 15.97(7.78,28.61) | | 6.67(2.76,12.53) | | 9.3(4.49,17.47) | | 38.48(13.42,69.57) | | 15.88(5.12,33.57) | | 22.6(8.58,41.74) | 35.13(23.65,49.3) | | 11.66(7.56,16.44) | | 23.47(15.36,33.47) | |  |  |
|  |  | Rate95%(UI) | | 0.02(0.02,0.03) | | 0.02(0.01,0.03) | | 0.03(0.02,0.04) | | 0.03(0.03,0.04) | | 0.02(0.02,0.02) | | 0.05(0.04,0.06) | | 0.04(0.03,0.05) | | 0.02(0.02,0.03) | | 0.06(0.04,0.08) | | 0.02(0.01,0.03) | | 0.01(0.01,0.02) | | 0.02(0.01,0.03) | | 0.02(0.01,0.04) | | 0.02(0.01,0.04) | | 0.03(0.01,0.05) | 0.02(0.01,0.03) | | 0.01(0.01,0.02) | | 0.03(0.02,0.04) | |  |  |
| 25-29 years | | Number95%(UI) | | 230.49(166.53,318.03) | | 67.76(43.44,100.07) | | 162.74(120.29,223.96) | | 32.81(26.23,38.78) | | 8.51(6.67,10.32) | | 24.31(19.5,28.99) | | 79.17(59.36,97.26) | | 16.73(12.33,21.36) | | 62.44(46.03,77.91) | | 21.25(9.83,39.01) | | 7.83(3.13,14.47) | | 13.41(6.36,25.74) | | 50.62(18.13,89.73) | | 19.79(6.04,39.84) | | 30.84(11.33,57.85) | 46.51(31.35,66.09) | | 14.87(9.69,21.23) | | 31.64(20.87,45.47) | |  |  |
|  |  | Rate95%(UI) | | 0.04(0.03,0.05) | | 0.02(0.01,0.03) | | 0.05(0.04,0.08) | | 0.05(0.04,0.05) | | 0.02(0.02,0.03) | | 0.07(0.05,0.08) | | 0.09(0.07,0.11) | | 0.04(0.03,0.05) | | 0.14(0.1,0.18) | | 0.02(0.01,0.05) | | 0.02(0.01,0.03) | | 0.03(0.02,0.06) | | 0.03(0.01,0.06) | | 0.02(0.01,0.05) | | 0.04(0.01,0.07) | 0.03(0.02,0.04) | | 0.02(0.01,0.02) | | 0.03(0.02,0.05) | |  |  |
| 30-34 years | | Number95%(UI) | | 428.44(321.42,556) | | 104.75(73.59,144.91) | | 323.69(241.37,418.65) | | 50.4(40.18,59.2) | | 13.16(10.37,15.95) | | 37.25(29.73,43.84) | | 214.56(158.76,264) | | 44.58(32.64,57.53) | | 169.98(125.19,210.54) | | 26.16(12.76,46.47) | | 7.6(3.06,14.48) | | 18.56(8.71,35.88) | | 68.94(26.6,118.41) | | 20.72(7,40.92) | | 48.21(18.44,84.54) | 68.16(44.83,95.65) | | 18.65(12.05,26.63) | | 49.51(32.28,70.97) | |  |  |
|  |  | Rate95%(UI) | | 0.07(0.05,0.09) | | 0.04(0.02,0.05) | | 0.11(0.08,0.14) | | 0.06(0.05,0.08) | | 0.04(0.03,0.04) | | 0.09(0.07,0.11) | | 0.2(0.15,0.25) | | 0.09(0.06,0.11) | | 0.31(0.23,0.38) | | 0.04(0.02,0.06) | | 0.02(0.01,0.04) | | 0.05(0.02,0.1) | | 0.05(0.02,0.08) | | 0.03(0.01,0.06) | | 0.07(0.02,0.11) | 0.03(0.02,0.05) | | 0.02(0.01,0.03) | | 0.05(0.03,0.07) | |  |  |
| 35-39 years | | Number95%(UI) | | 664.76(487.3,866.78) | | 157.11(113.36,212.07) | | 507.65(376.37,654.09) | | 74.42(59.87,86.47) | | 18.8(14.85,22.64) | | 55.61(44.67,64.17) | | 382.83(285.38,477.8) | | 77.89(56.76,102.32) | | 304.94(229.2,382.99) | | 32.21(16.03,55.98) | | 9.9(4.05,17.6) | | 22.31(11.1,41.11) | | 84.33(32.57,149.13) | | 26.25(8.81,49.95) | | 58.08(22.54,103.16) | 90.56(58.14,125.31) | | 24.2(15.46,34.97) | | 66.36(42.6,92.01) | |  |  |
|  |  | Rate95%(UI) | | 0.12(0.09,0.15) | | 0.06(0.04,0.08) | | 0.18(0.13,0.23) | | 0.09(0.08,0.11) | | 0.05(0.04,0.06) | | 0.14(0.11,0.16) | | 0.38(0.28,0.47) | | 0.16(0.11,0.21) | | 0.59(0.44,0.74) | | 0.05(0.03,0.09) | | 0.03(0.01,0.06) | | 0.07(0.04,0.14) | | 0.06(0.02,0.11) | | 0.04(0.01,0.08) | | 0.09(0.03,0.15) | 0.05(0.03,0.07) | | 0.03(0.02,0.04) | | 0.07(0.05,0.1) | |  |  |
| 40-44 years | | Number95%(UI) | | 916.31(672.91,1196.03) | | 205.63(148.89,279.14) | | 710.67(525.29,924.11) | | 110.68(89.04,128.87) | | 25.05(19.56,30.58) | | 85.63(69.28,99.02) | | 518.04(389.67,647.83) | | 108.89(79.96,138.02) | | 409.15(308.71,518.72) | | 47.5(22.85,87.14) | | 11.11(4.67,20.79) | | 36.39(17.31,70.4) | | 124.18(46.94,214.71) | | 30.6(9.55,57.75) | | 93.57(33.94,169.81) | 115.1(75.78,161.51) | | 29.87(18.99,42.71) | | 85.23(55.39,119.24) | |  |  |
|  |  | Rate95%(UI) | | 0.18(0.13,0.24) | | 0.08(0.06,0.11) | | 0.28(0.21,0.37) | | 0.15(0.12,0.17) | | 0.07(0.05,0.08) | | 0.22(0.18,0.25) | | 0.56(0.42,0.7) | | 0.24(0.18,0.3) | | 0.87(0.66,1.11) | | 0.09(0.04,0.17) | | 0.04(0.02,0.08) | | 0.14(0.07,0.28) | | 0.11(0.04,0.19) | | 0.05(0.02,0.1) | | 0.16(0.06,0.29) | 0.07(0.05,0.1) | | 0.04(0.02,0.05) | | 0.1(0.07,0.14) | |  |  |
| 45-49 years | | Number95%(UI) | | 1107.89(819.68,1418.88) | | 259.03(188.03,351.62) | | 848.86(628.25,1079.03) | | 150.17(121.53,172.89) | | 33.45(26.21,39.4) | | 116.72(94.84,134.44) | | 603.28(451.78,741.59) | | 129.89(96.53,170.14) | | 473.39(353.87,585.41) | | 54.16(26.69,95.55) | | 14.38(5.94,25.76) | | 39.78(18.85,75.66) | | 150.29(56.06,271.58) | | 40.15(13.17,76.74) | | 110.14(40.72,204.07) | 148.72(96.09,209.13) | | 40.99(26.3,59.18) | | 107.73(68.52,152.12) | |  |  |
|  |  | Rate95%(UI) | | 0.23(0.17,0.3) | | 0.11(0.08,0.15) | | 0.36(0.26,0.45) | | 0.2(0.17,0.24) | | 0.09(0.07,0.11) | | 0.31(0.25,0.36) | | 0.62(0.47,0.77) | | 0.27(0.2,0.35) | | 0.97(0.73,1.2) | | 0.13(0.06,0.23) | | 0.07(0.03,0.12) | | 0.19(0.09,0.36) | | 0.15(0.06,0.28) | | 0.08(0.03,0.16) | | 0.22(0.08,0.42) | 0.09(0.06,0.13) | | 0.05(0.03,0.07) | | 0.13(0.08,0.19) | |  |  |
| 50-54 years | | Number95%(UI) | | 1330.72(1005.42,1713.84) | | 307.15(228.73,399.74) | | 1023.56(769.32,1325.21) | | 219.93(172.6,252.09) | | 50.09(39.57,59.81) | | 169.83(133.21,196.54) | | 681.32(507.83,846.61) | | 145.19(108.1,194.13) | | 536.13(396.76,667.37) | | 58.49(28.29,107.97) | | 14.54(6.32,27.08) | | 43.96(20.37,85.26) | | 177.31(68.31,324.25) | | 46.07(14.88,87.03) | | 131.24(49.46,244.22) | 191.68(125.96,271.77) | | 50.98(34.47,71.72) | | 140.7(92.24,201.73) | |  |  |
|  |  | Rate95%(UI) | | 0.3(0.23,0.39) | | 0.14(0.1,0.18) | | 0.46(0.35,0.6) | | 0.3(0.23,0.34) | | 0.14(0.11,0.16) | | 0.45(0.36,0.52) | | 0.7(0.52,0.87) | | 0.3(0.22,0.4) | | 1.11(0.82,1.38) | | 0.18(0.09,0.33) | | 0.09(0.04,0.17) | | 0.27(0.12,0.52) | | 0.21(0.08,0.39) | | 0.11(0.04,0.21) | | 0.32(0.12,0.59) | 0.12(0.08,0.17) | | 0.06(0.04,0.09) | | 0.18(0.12,0.26) | |  |  |
| 55-59 years | | Number95%(UI) | | 1686.97(1283.03,2174.58) | | 455.54(338.92,607.96) | | 1231.44(932.16,1580.45) | | 318.14(250.26,357.51) | | 73.74(57.46,86.15) | | 244.4(192.23,276.23) | | 771.43(588.17,958.98) | | 197.12(148.16,269.15) | | 574.31(434.37,717.81) | | 84.25(37.32,146.26) | | 26.94(9.83,50.55) | | 57.31(25.17,104.01) | | 247.17(86.1,428.42) | | 74.48(24.65,146.13) | | 172.69(60.86,312.6) | 263.45(171.3,370.06) | | 82.83(53.51,118.34) | | 180.62(115.09,258.73) | |  |  |
|  |  | Rate95%(UI) | | 0.43(0.32,0.55) | | 0.23(0.17,0.3) | | 0.63(0.48,0.81) | | 0.44(0.34,0.49) | | 0.2(0.16,0.24) | | 0.67(0.53,0.76) | | 0.86(0.65,1.07) | | 0.43(0.32,0.59) | | 1.3(0.99,1.63) | | 0.33(0.15,0.58) | | 0.21(0.08,0.4) | | 0.45(0.2,0.83) | | 0.36(0.12,0.62) | | 0.21(0.07,0.41) | | 0.51(0.18,0.92) | 0.19(0.12,0.27) | | 0.12(0.08,0.17) | | 0.27(0.17,0.38) | |  |  |
| 60-64 years | | Number95%(UI) | | 1837.18(1406.46,2334.88) | | 513.07(383.3,681.46) | | 1324.1(1010.84,1690.47) | | 407.26(321.26,461.99) | | 102.28(78.93,118.03) | | 304.98(240.53,348.27) | | 805.77(608.3,1009.78) | | 207.21(155.98,282.47) | | 598.56(448.49,755.15) | | 88.79(38.59,165.04) | | 30.8(11.09,59.36) | | 57.99(24.06,114.43) | | 264.24(90.85,469.07) | | 83.78(26.18,156.7) | | 180.46(64.57,323.95) | 267.74(176.02,376.35) | | 88.3(57.41,124.66) | | 179.44(115.74,255.45) | |  |  |
|  |  | Rate95%(UI) | | 0.57(0.44,0.73) | | 0.31(0.23,0.41) | | 0.85(0.65,1.09) | | 0.6(0.47,0.68) | | 0.29(0.23,0.34) | | 0.91(0.72,1.04) | | 1.1(0.83,1.38) | | 0.55(0.41,0.74) | | 1.71(1.28,2.16) | | 0.45(0.2,0.84) | | 0.31(0.11,0.6) | | 0.6(0.25,1.17) | | 0.46(0.16,0.82) | | 0.28(0.09,0.53) | | 0.65(0.23,1.17) | 0.26(0.17,0.37) | | 0.17(0.11,0.24) | | 0.36(0.23,0.51) | |  |  |
| 65-69 years | | Number95%(UI) | | 1859.16(1442.99,2374.85) | | 586.71(445.24,770.68) | | 1272.46(975.96,1606.69) | | 499.33(400.02,558.24) | | 136.58(107.79,155.74) | | 362.75(289.24,407.64) | | 669.77(516.12,811.89) | | 208.67(155.67,262.43) | | 461.1(354.38,554.34) | | 95.08(40.67,178.45) | | 33.45(12.4,63.29) | | 61.63(25.09,121.07) | | 286.42(98.95,512.37) | | 96.19(30.01,181.51) | | 190.23(67.07,345.05) | 304.29(204.41,430.84) | | 110.66(72.85,160.37) | | 193.62(128.98,272.29) | |  |  |
|  |  | Rate95%(UI) | | 0.67(0.52,0.86) | | 0.41(0.31,0.54) | | 0.97(0.74,1.22) | | 0.82(0.66,0.92) | | 0.43(0.34,0.49) | | 1.25(1,1.4) | | 1.01(0.78,1.22) | | 0.59(0.44,0.74) | | 1.49(1.15,1.79) | | 0.63(0.27,1.18) | | 0.44(0.16,0.82) | | 0.83(0.34,1.64) | | 0.65(0.22,1.16) | | 0.42(0.13,0.79) | | 0.89(0.31,1.62) | 0.34(0.23,0.48) | | 0.24(0.16,0.35) | | 0.45(0.3,0.63) | |  |  |
| 70-74 years | | Number95%(UI) | | 1901.35(1480.37,2406.03) | | 685.77(522.43,892.82) | | 1215.59(943.82,1526.49) | | 621.63(486.59,705.28) | | 195.88(152.54,230.36) | | 425.75(326.88,480.74) | | 581.56(428.52,699.88) | | 216.42(154.54,272.59) | | 365.15(269.8,443.25) | | 104.68(40.28,198.12) | | 42.58(14.34,82.73) | | 62.1(23.18,126.5) | | 293.06(93.5,521.01) | | 111.45(34.55,214.2) | | 181.61(60.41,331.92) | 295.66(197.13,412.51) | | 117.75(76.15,167.95) | | 177.91(115.73,252.92) | |  |  |
|  |  | Rate95%(UI) | | 0.92(0.72,1.17) | | 0.63(0.48,0.82) | | 1.26(0.98,1.58) | | 1.16(0.91,1.32) | | 0.69(0.54,0.81) | | 1.7(1.31,1.92) | | 1.19(0.88,1.43) | | 0.81(0.57,1.01) | | 1.66(1.23,2.02) | | 1.01(0.39,1.91) | | 0.8(0.27,1.56) | | 1.23(0.46,2.5) | | 0.93(0.3,1.66) | | 0.68(0.21,1.3) | | 1.21(0.4,2.22) | 0.48(0.32,0.67) | | 0.36(0.24,0.52) | | 0.61(0.4,0.86) | |  |  |
| 75-79 years | | Number95%(UI) | | 1909.28(1496.24,2385.47) | | 794.46(617.14,1009.52) | | 1114.82(871.57,1396.9) | | 665.53(521.88,751.81) | | 246.88(192.04,283.52) | | 418.65(330.43,471.61) | | 547.84(399.75,648.09) | | 248.58(185.16,296.07) | | 299.26(208.57,361.71) | | 96.86(36.35,180.38) | | 43.36(14.31,86.98) | | 53.5(20.9,106.28) | | 290.38(96.02,517.31) | | 122.44(40.19,227.48) | | 167.94(52.31,314.48) | 303.73(195.44,428.08) | | 131.01(81.39,187.8) | | 172.72(110.49,250.06) | |  |  |
|  |  | Rate95%(UI) | | 1.45(1.13,1.81) | | 1.1(0.86,1.4) | | 1.86(1.46,2.34) | | 1.84(1.44,2.08) | | 1.24(0.97,1.43) | | 2.56(2.02,2.89) | | 1.83(1.34,2.17) | | 1.47(1.1,1.75) | | 2.31(1.61,2.79) | | 1.51(0.57,2.81) | | 1.3(0.43,2.62) | | 1.73(0.67,3.43) | | 1.45(0.48,2.58) | | 1.14(0.37,2.12) | | 1.8(0.56,3.37) | 0.78(0.5,1.09) | | 0.62(0.38,0.89) | | 0.96(0.61,1.39) | |  |  |
| 80-84 years | | Number95%(UI) | | 2509.18(1977.54,3046.44) | | 1273.61(981.86,1545.92) | | 1235.57(989.6,1503.72) | | 892.07(675.84,1021.28) | | 402.4(300.43,471.74) | | 489.67(380.72,560.54) | | 906(691.89,1060.15) | | 531.9(404.05,629.88) | | 374.1(276.61,444.72) | | 86.07(32.37,164.2) | | 40.38(14.23,81.75) | | 45.69(16.87,99.6) | | 275.95(94.22,501.26) | | 123.52(40.96,234.04) | | 152.43(52.81,292.47) | 341.96(219.6,482.42) | | 171.44(108.22,253.3) | | 170.52(112.59,243.52) | |  |  |
|  |  | Rate95%(UI) | | 2.86(2.26,3.48) | | 2.5(1.93,3.04) | | 3.37(2.7,4.1) | | 3.31(2.51,3.79) | | 2.57(1.92,3.02) | | 4.32(3.36,4.95) | | 4.15(3.17,4.86) | | 3.96(3.01,4.69) | | 4.45(3.29,5.29) | | 2.55(0.96,4.87) | | 2.22(0.78,4.5) | | 2.93(1.08,6.4) | | 2.4(0.82,4.36) | | 1.92(0.64,3.63) | | 3.01(1.04,5.78) | 1.44(0.92,2.03) | | 1.26(0.8,1.87) | | 1.66(1.1,2.38) | |  |  |
| 85-89 years | | Number95%(UI) | | 2513.79(1909.32,3004.18) | | 1401.61(1052.11,1676.08) | | 1112.17(866.12,1340.13) | | 1060.83(788.78,1232.33) | | 569.04(416.81,674.11) | | 491.78(370.97,565.65) | | 883.01(637.92,1036.2) | | 551.07(401.82,653.43) | | 331.95(235.63,389.67) | | 51.93(20.02,96.57) | | 24.01(8.76,47.66) | | 27.91(10.58,56.56) | | 194.66(66.8,353.5) | | 90.38(30.3,173.11) | | 104.28(35.48,197.87) | 315.88(201.41,448.98) | | 162.4(87.81,240.08) | | 153.48(97.97,216.49) | |  |  |
|  |  | Rate95%(UI) | | 5.5(4.18,6.57) | | 4.92(3.7,5.89) | | 6.45(5.02,7.77) | | 6.43(4.78,7.47) | | 5.51(4.04,6.53) | | 7.97(6.01,9.16) | | 7.76(5.61,9.11) | | 7.4(5.4,8.77) | | 8.45(6,9.92) | | 3.89(1.5,7.23) | | 3.23(1.18,6.4) | | 4.73(1.79,9.57) | | 3.9(1.34,7.07) | | 3.15(1.06,6.03) | | 4.9(1.67,9.3) | 2.75(1.76,3.92) | | 2.3(1.24,3.4) | | 3.48(2.22,4.91) | |  |  |
| 90-94 years | | Number95%(UI) | | 1764.51(1313.19,2093.68) | | 1117.7(808.46,1339.67) | | 646.81(492.27,781.82) | | 876.13(625.53,1031.47) | | 551.17(388.06,656.96) | | 324.95(243.87,376.56) | | 585.81(422.6,700.12) | | 401.77(291.39,490.14) | | 184.04(131.3,219.88) | | 16.26(6.26,28.67) | | 8.29(2.8,16.76) | | 7.97(3.18,15.55) | | 94.71(33.4,173.24) | | 46.71(15.47,87.23) | | 47.99(15.44,91.22) | 186.93(109.63,272.61) | | 106.6(58.53,162.01) | | 80.33(51.32,116) | |  |  |
|  |  | Rate95%(UI) | | 9.86(7.34,11.7) | | 9.27(6.7,11.11) | | 11.1(8.45,13.41) | | 11.43(8.16,13.45) | | 10.49(7.39,12.51) | | 13.47(10.11,15.6) | | 13.4(9.67,16.01) | | 12.97(9.41,15.82) | | 14.45(10.31,17.26) | | 4.54(1.75,8) | | 4.06(1.37,8.22) | | 5.16(2.06,10.06) | | 5.85(2.06,10.71) | | 4.93(1.63,9.21) | | 7.15(2.3,13.6) | 4.85(2.84,7.07) | | 4.19(2.3,6.36) | | 6.13(3.92,8.86) | |  |  |
| 95+ years | | Number95%(UI) | | 837.4(575.22,1001.59) | | 606.54(407.7,735.95) | | 230.86(165.83,271.49) | | 480.44(321.4,571.95) | | 349.68(231.67,421.52) | | 130.76(92.02,154.3) | | 245.03(166.08,303.09) | | 181.43(121.02,225.24) | | 63.6(42.71,78.91) | | 4.03(1.6,7.44) | | 2.52(0.84,4.92) | | 1.51(0.62,2.93) | | 32.78(11.56,59.88) | | 17.39(6.1,34.82) | | 15.39(5.02,30.45) | 73.26(38.05,112.54) | | 54.22(27.28,85.92) | | 19.05(10.81,28.68) | |  |  |
|  |  | Rate95%(UI) | | 15.36(10.55,18.38) | | 15.4(10.35,18.69) | | 15.27(10.97,17.96) | | 17.34(11.6,20.65) | | 16.97(11.24,20.45) | | 18.44(12.98,21.76) | | 22.2(15.05,27.46) | | 21.93(14.63,27.23) | | 23(15.44,28.53) | | 5.29(2.1,9.77) | | 5.19(1.73,10.15) | | 5.48(2.24,10.63) | | 7.23(2.55,13.2) | | 5.99(2.1,12) | | 9.42(3.07,18.64) | 7.04(3.66,10.82) | | 7.67(3.86,12.16) | | 5.71(3.24,8.6) | |  |  |
| ASMR: Age-standardized mortality rates. SDI: Sociodemographic Index. | | | | | | | | | | | | | | | | | | | | | | | | | | | | | | | | | | | | | | |  |  |
| **Table S9. Age-specific death numbers and ASMR for ischemic heart disease attributable to high temperature, categorized by 5-year age groups, presented globally and by SDI region in 2021.** | | | | | | | | | | | | | | | | | | | | | | | | | | | | | | | | | | | | | | |  |  |
|  | |  | | Global | | | | | | High SDI | | | | | | High-middle SDI | | | | | | Low SDI | | | | | | Low-middle SDI | | | | | | | Middle SDI | | | | | |
| age | | metric | | Both | | Female | | Male | | Both | | Female | | Male | | Both | | Female | | Male | | Both | | Female | | Male | | Both | | Female | | Male | | | Both | | Female | | Male | |
| 15-19 years | | Number95%(UI) | | 190.08(38.59,395.47) | | 92.51(20.13,183.46) | | 97.57(16.27,213.61) | | 4.82(0.93,10.08) | | 1.74(0.34,3.47) | | 3.08(0.53,6.56) | | 5.26(-1.07,15.48) | | 1.92(-0.21,5.39) | | 3.34(-0.88,10.23) | | 21.61(1.39,46.85) | | 12.05(0.63,26.56) | | 9.55(0.67,21.45) | | 117.26(23.83,242.62) | | 58.59(13.26,114.17) | | 58.66(10.31,129.1) | | | 41.06(10.02,84.06) | | 18.17(4.5,34.92) | | 22.9(5.21,49.21) | |
|  |  | Rate95%(UI) | | 0.03(0.01,0.06) | | 0.03(0.01,0.06) | | 0.03(0.01,0.07) | | 0.01(0,0.02) | | 0.01(0,0.01) | | 0.01(0,0.02) | | 0.01(0,0.02) | | 0.01(0,0.02) | | 0.01(0,0.03) | | 0.02(0,0.04) | | 0.02(0,0.04) | | 0.02(0,0.03) | | 0.06(0.01,0.13) | | 0.06(0.01,0.13) | | 0.06(0.01,0.14) | | | 0.02(0.01,0.05) | | 0.02(0.01,0.04) | | 0.02(0.01,0.05) | |
| 20-24 years | | Number95%(UI) | | 376.77(78.44,777.09) | | 151(33.03,294.72) | | 225.77(44.36,471.87) | | 13.66(2.78,29.67) | | 4.58(0.98,9.93) | | 9.08(1.81,20.15) | | 12.08(-3.55,37.2) | | 3.53(-0.72,10.19) | | 8.55(-2.74,26.61) | | 38.05(3.56,79.52) | | 18.89(1.72,39.56) | | 19.16(1.57,41.88) | | 231.7(51.27,465.97) | | 93.38(21.62,179.11) | | 138.32(27.87,286.5) | | | 81.18(19.09,163.05) | | 30.58(7.58,59.84) | | 50.6(11.74,103.86) | |
|  |  | Rate95%(UI) | | 0.06(0.01,0.13) | | 0.05(0.01,0.1) | | 0.07(0.01,0.16) | | 0.02(0,0.05) | | 0.01(0,0.03) | | 0.03(0.01,0.06) | | 0.02(0,0.05) | | 0.01(0,0.03) | | 0.02(-0.01,0.07) | | 0.04(0,0.08) | | 0.04(0,0.08) | | 0.04(0,0.08) | | 0.13(0.03,0.27) | | 0.11(0.02,0.21) | | 0.16(0.03,0.33) | | | 0.05(0.01,0.09) | | 0.04(0.01,0.07) | | 0.06(0.01,0.11) | |
| 25-29 years | | Number95%(UI) | | 599.72(123.52,1223.8) | | 210.38(43.13,410.19) | | 389.33(78.68,786.19) | | 37.04(7.65,77.26) | | 10.19(2.26,20.5) | | 26.85(5.16,57.16) | | 23.12(-6.08,68.27) | | 5.3(-1.04,15.59) | | 17.83(-5.38,53.3) | | 45.2(3.54,97.4) | | 18.98(1.22,42.04) | | 26.22(2.25,59.47) | | 347.95(78.63,682.26) | | 128.11(28.94,246.98) | | 219.84(48.26,426.71) | | | 146.23(36.27,293.4) | | 47.75(10.27,93.72) | | 98.48(25.86,197.77) | |
|  |  | Rate95%(UI) | | 0.1(0.02,0.21) | | 0.07(0.01,0.14) | | 0.13(0.03,0.26) | | 0.05(0.01,0.11) | | 0.03(0.01,0.06) | | 0.07(0.01,0.16) | | 0.03(-0.01,0.08) | | 0.01(0,0.04) | | 0.04(-0.01,0.12) | | 0.05(0,0.11) | | 0.04(0,0.1) | | 0.06(0.01,0.14) | | 0.21(0.05,0.42) | | 0.16(0.04,0.3) | | 0.27(0.06,0.53) | | | 0.08(0.02,0.16) | | 0.05(0.01,0.1) | | 0.11(0.03,0.21) | |
| 30-34 years | | Number95%(UI) | | 1164.03(240.23,2354.55) | | 352.54(78.13,692.5) | | 811.49(164.78,1667.65) | | 92.57(19.23,202.17) | | 22.09(4.78,47.12) | | 70.48(14.5,157.26) | | 55.07(-14.91,170.59) | | 11.21(-2.16,33.21) | | 43.86(-13.08,138.58) | | 87.76(8.89,180.17) | | 35.16(4.64,73.72) | | 52.6(4.39,110.29) | | 634.29(153.59,1192.08) | | 202.96(47.78,381.33) | | 431.32(103.19,818.22) | | | 294.01(69.17,587.47) | | 81.02(20.32,163.55) | | 212.99(49.41,432.04) | |
|  |  | Rate95%(UI) | | 0.19(0.04,0.39) | | 0.12(0.03,0.23) | | 0.27(0.05,0.55) | | 0.12(0.02,0.26) | | 0.06(0.01,0.13) | | 0.18(0.04,0.39) | | 0.05(-0.01,0.16) | | 0.02(0,0.06) | | 0.08(-0.02,0.25) | | 0.12(0.01,0.25) | | 0.09(0.01,0.2) | | 0.15(0.01,0.31) | | 0.43(0.1,0.81) | | 0.27(0.06,0.52) | | 0.58(0.14,1.11) | | | 0.15(0.03,0.29) | | 0.08(0.02,0.17) | | 0.21(0.05,0.43) | |
| 35-39 years | | Number95%(UI) | | 1771.89(385.69,3576.03) | | 514.68(113.16,999.13) | | 1257.2(271.9,2532.4) | | 177.81(35.09,366.62) | | 39.41(7.82,83.61) | | 138.39(27.57,293.65) | | 88.86(-23.11,278.88) | | 18.16(-3.58,55.24) | | 70.7(-19.28,220.19) | | 130.47(14.25,268.82) | | 48.17(5.16,98.6) | | 82.3(8.26,170.72) | | 914.61(231.4,1737.81) | | 295.04(71.46,548.95) | | 619.57(159.93,1184) | | | 459.6(108.95,941.37) | | 113.74(30.19,227.9) | | 345.85(80.38,713.62) | |
|  |  | Rate95%(UI) | | 0.32(0.07,0.64) | | 0.19(0.04,0.36) | | 0.44(0.1,0.89) | | 0.23(0.04,0.47) | | 0.1(0.02,0.22) | | 0.34(0.07,0.72) | | 0.09(-0.02,0.27) | | 0.04(-0.01,0.11) | | 0.14(-0.04,0.42) | | 0.21(0.02,0.43) | | 0.15(0.02,0.31) | | 0.27(0.03,0.56) | | 0.69(0.17,1.3) | | 0.44(0.11,0.82) | | 0.93(0.24,1.77) | | | 0.25(0.06,0.51) | | 0.12(0.03,0.25) | | 0.37(0.09,0.77) | |
| 40-44 years | | Number95%(UI) | | 3052.91(700.81,6137.52) | | 852.72(206.04,1658.86) | | 2200.19(491.03,4497.14) | | 333.28(67.34,701.36) | | 71.07(15.23,149.1) | | 262.21(50.4,556.22) | | 151.1(-34.63,479.66) | | 31.51(-5.55,95.86) | | 119.59(-28.5,381.2) | | 223.08(26.06,456.11) | | 74.05(7.95,158.15) | | 149.03(16.27,313.97) | | 1546.39(415.93,2908.36) | | 468.8(130.33,866.4) | | 1077.59(280.13,2045.26) | | | 798.08(194.44,1588.8) | | 207.03(49.69,403.74) | | 591.05(146.84,1189.39) | |
|  |  | Rate95%(UI) | | 0.61(0.14,1.23) | | 0.34(0.08,0.67) | | 0.87(0.19,1.78) | | 0.44(0.09,0.93) | | 0.19(0.04,0.41) | | 0.67(0.13,1.43) | | 0.16(-0.04,0.52) | | 0.07(-0.01,0.21) | | 0.26(-0.06,0.81) | | 0.43(0.05,0.88) | | 0.28(0.03,0.61) | | 0.58(0.06,1.23) | | 1.34(0.36,2.52) | | 0.81(0.23,1.5) | | 1.87(0.49,3.55) | | | 0.48(0.12,0.96) | | 0.25(0.06,0.49) | | 0.71(0.18,1.43) | |
| 45-49 years | | Number95%(UI) | | 4444.7(964.99,9068.21) | | 1325.52(317.71,2578.94) | | 3119.18(649.5,6388.77) | | 464.3(84.53,1011.91) | | 105.07(17.99,228.44) | | 359.23(68.16,780.05) | | 237.38(-55,771.94) | | 52.09(-10.1,166.93) | | 185.29(-44.5,598.45) | | 335.96(41.04,705.11) | | 117.11(14.88,242.37) | | 218.85(23.96,465.92) | | 2108.93(565.43,3969.35) | | 670.08(185.93,1218.47) | | 1438.85(369.51,2747.05) | | | 1296.57(311.02,2646.35) | | 380.76(91.05,763.53) | | 915.82(217.78,1890.39) | |
|  |  | Rate95%(UI) | | 0.94(0.2,1.92) | | 0.56(0.13,1.09) | | 1.31(0.27,2.69) | | 0.63(0.12,1.38) | | 0.29(0.05,0.64) | | 0.96(0.18,2.08) | | 0.24(-0.06,0.8) | | 0.11(-0.02,0.35) | | 0.38(-0.09,1.23) | | 0.81(0.1,1.69) | | 0.56(0.07,1.17) | | 1.05(0.11,2.23) | | 2.14(0.57,4.03) | | 1.36(0.38,2.46) | | 2.93(0.75,5.6) | | | 0.8(0.19,1.63) | | 0.47(0.11,0.94) | | 1.12(0.27,2.32) | |
| 50-54 years | | Number95%(UI) | | 6443.94(1338,13180.14) | | 1857.39(410.53,3725.89) | | 4586.55(922.66,9512.09) | | 556.57(73.13,1296.53) | | 132.87(17.69,302.75) | | 423.7(56.11,992.42) | | 387.94(-85.92,1282.06) | | 87.86(-17.61,290.66) | | 300.07(-71.7,983.04) | | 467.45(55.54,961.66) | | 155.42(17.65,311.55) | | 312.03(35.53,657.88) | | 3088.77(816.1,5795.31) | | 938.17(252.25,1781.53) | | 2150.6(554.42,4084.46) | | | 1940.91(439.52,4030.25) | | 542.44(124.35,1108.55) | | 1398.47(315.97,2900.45) | |
|  |  | Rate95%(UI) | | 1.45(0.3,2.96) | | 0.83(0.18,1.67) | | 2.07(0.42,4.29) | | 0.75(0.1,1.75) | | 0.36(0.05,0.82) | | 1.13(0.15,2.65) | | 0.4(-0.09,1.32) | | 0.18(-0.04,0.6) | | 0.62(-0.15,2.03) | | 1.43(0.17,2.94) | | 0.95(0.11,1.91) | | 1.9(0.22,4) | | 3.68(0.97,6.9) | | 2.22(0.6,4.21) | | 5.16(1.33,9.81) | | | 1.24(0.28,2.57) | | 0.69(0.16,1.4) | | 1.8(0.41,3.73) | |
| 55-59 years | | Number95%(UI) | | 9003.96(1882.22,18684.65) | | 3040.82(728.28,6188.41) | | 5963.14(1164.72,12682.66) | | 659.62(62.46,1613.8) | | 168.44(16.22,399.11) | | 491.18(37.9,1211.91) | | 592.01(-125.02,1949.99) | | 166.58(-30.9,528.9) | | 425.43(-96.41,1425.25) | | 624.26(84.63,1277.33) | | 238.8(30.37,466.2) | | 385.46(48.97,794.36) | | 4320.24(1181.2,8142.14) | | 1493.34(425.48,2696.29) | | 2826.91(731.11,5411.61) | | | 2804.79(627.18,5776.85) | | 972.77(237.14,1985.72) | | 1832.01(392.6,3803.87) | |
|  |  | Rate95%(UI) | | 2.28(0.48,4.72) | | 1.51(0.36,3.08) | | 3.06(0.6,6.51) | | 0.91(0.09,2.22) | | 0.46(0.04,1.09) | | 1.36(0.1,3.35) | | 0.66(-0.14,2.17) | | 0.36(-0.07,1.15) | | 0.97(-0.22,3.23) | | 2.46(0.33,5.04) | | 1.88(0.24,3.66) | | 3.06(0.39,6.31) | | 6.22(1.7,11.72) | | 4.23(1.21,7.64) | | 8.28(2.14,15.84) | | | 2.03(0.45,4.19) | | 1.38(0.34,2.82) | | 2.71(0.58,5.63) | |
| 60-64 years | | Number95%(UI) | | 10861.99(2181.77,23312.92) | | 3835.87(831.82,7874.22) | | 7026.12(1377.37,15309.97) | | 737.5(24.85,2029.37) | | 206.26(8.14,552.82) | | 531.24(15.68,1503.14) | | 833.95(-141.03,2776.28) | | 261.99(-39.01,882.4) | | 571.96(-104.47,1914.8) | | 759.43(107.02,1503.91) | | 300.35(42.68,582.41) | | 459.08(66.58,930.92) | | 4957.32(1317.3,9441.24) | | 1832.4(483.66,3360.68) | | 3124.92(809.99,6007.18) | | | 3569.77(783.26,7416.75) | | 1233.57(288.86,2519.55) | | 2336.2(507.31,4863.32) | |
|  |  | Rate95%(UI) | | 3.39(0.68,7.28) | | 2.33(0.51,4.79) | | 4.52(0.89,9.84) | | 1.08(0.04,2.98) | | 0.59(0.02,1.59) | | 1.59(0.05,4.51) | | 1.14(-0.19,3.81) | | 0.69(-0.1,2.32) | | 1.64(-0.3,5.48) | | 3.85(0.54,7.63) | | 3.01(0.43,5.84) | | 4.71(0.68,9.56) | | 8.67(2.3,16.52) | | 6.23(1.64,11.43) | | 11.26(2.92,21.64) | | | 3.51(0.77,7.29) | | 2.36(0.55,4.83) | | 4.71(1.02,9.8) | |
| 65-69 years | | Number95%(UI) | | 13303.18(2413.22,29060.39) | | 5188.12(1019.82,11028.44) | | 8115.06(1425.56,17905.55) | | 819.53(-20.08,2425.71) | | 244.81(-9.21,727.88) | | 574.72(-13.01,1697.83) | | 1208.77(-240.12,4124.34) | | 449.03(-79.75,1540.49) | | 759.74(-167.95,2586.39) | | 962.86(129.87,1923.44) | | 399.44(60.32,780.88) | | 563.42(88,1132.74) | | 5865.55(1561.48,11067.06) | | 2403.02(649.64,4512.19) | | 3462.53(903.96,6622.06) | | | 4441.39(888.7,9441.64) | | 1689.98(352.4,3513.03) | | 2751.41(537.92,5821.54) | |
|  |  | Rate95%(UI) | | 4.82(0.87,10.54) | | 3.6(0.71,7.66) | | 6.16(1.08,13.58) | | 1.35(-0.03,4) | | 0.78(-0.03,2.31) | | 1.98(-0.04,5.84) | | 1.82(-0.36,6.21) | | 1.27(-0.22,4.34) | | 2.46(-0.54,8.36) | | 6.39(0.86,12.76) | | 5.2(0.79,10.16) | | 7.62(1.19,15.32) | | 13.22(3.52,24.95) | | 10.44(2.82,19.61) | | 16.22(4.23,31.02) | | | 4.98(1,10.59) | | 3.66(0.76,7.61) | | 6.4(1.25,13.54) | |
| 70-74 years | | Number95%(UI) | | 14448.46(2453.32,31917.84) | | 5986.89(1029.86,13213.79) | | 8461.58(1443.56,18863.43) | | 908.42(-66.75,2898.75) | | 324.24(-15.77,1013.27) | | 584.17(-50.75,1856.3) | | 1552.92(-322,5366.72) | | 671.93(-117.27,2356.69) | | 880.98(-213.85,3020.09) | | 1098.33(185.37,2151.49) | | 461.63(63.64,904.21) | | 636.7(117.73,1234.48) | | 6318.67(1770.08,11691.53) | | 2679.87(746.37,4944.77) | | 3638.8(1001.83,6754.26) | | | 4564.38(765.26,10183.33) | | 1846.8(314.91,4106.34) | | 2717.58(417.49,5979.6) | |
|  |  | Rate95%(UI) | | 7.02(1.19,15.51) | | 5.47(0.94,12.07) | | 8.78(1.5,19.57) | | 1.7(-0.13,5.43) | | 1.14(-0.06,3.58) | | 2.33(-0.2,7.42) | | 3.18(-0.66,10.99) | | 2.5(-0.44,8.77) | | 4.01(-0.97,13.75) | | 10.59(1.79,20.75) | | 8.7(1.2,17.03) | | 12.58(2.33,24.39) | | 20.09(5.63,37.18) | | 16.27(4.53,30.01) | | 24.31(6.69,45.12) | | | 7.41(1.24,16.52) | | 5.71(0.97,12.7) | | 9.28(1.43,20.42) | |
| 75-79 years | | Number95%(UI) | | 13987.17(2111.7,32059.89) | | 6306.85(948.13,14444.49) | | 7680.32(1183.96,17526.84) | | 877.47(-118.04,2949.76) | | 339.72(-45.14,1140.92) | | 537.75(-72.79,1794.92) | | 1688.09(-407.21,5914.66) | | 808.52(-161.41,2860.17) | | 879.57(-246.86,3071.12) | | 975.54(162.8,1881.8) | | 457.08(71.91,894.2) | | 518.46(94.07,1036.79) | | 5781.92(1658.94,10764.64) | | 2661.86(732.85,4939.57) | | 3120.06(898.47,5879.63) | | | 4657.97(705.9,10660.61) | | 2036.7(326.52,4619.77) | | 2621.27(362.48,6063.29) | |
|  |  | Rate95%(UI) | | 10.61(1.6,24.31) | | 8.75(1.32,20.03) | | 12.85(1.98,29.32) | | 2.42(-0.33,8.15) | | 1.71(-0.23,5.74) | | 3.29(-0.45,10.99) | | 5.65(-1.36,19.8) | | 4.78(-0.95,16.92) | | 6.78(-1.9,23.69) | | 15.19(2.54,29.31) | | 13.75(2.16,26.89) | | 16.75(3.04,33.49) | | 28.8(8.26,53.61) | | 24.76(6.82,45.94) | | 33.45(9.63,63.03) | | | 11.89(1.8,27.21) | | 9.62(1.54,21.83) | | 14.55(2.01,33.66) | |
| 80-84 years | | Number95%(UI) | | 14098.47(1482.33,34130.57) | | 6945.11(748.96,16989.83) | | 7153.36(698.96,17468.09) | | 1006.56(-181.3,3589.44) | | 444.44(-87.16,1625.01) | | 562.12(-94.73,1971.37) | | 2455.7(-584.94,8570.63) | | 1356.95(-259.95,4831.6) | | 1098.74(-328.94,3896.67) | | 825.44(154.44,1617.88) | | 376.86(58.54,760.84) | | 448.58(91.99,889.44) | | 4977.4(1413.6,9164.05) | | 2426.37(669.8,4452.45) | | 2551.03(740.25,4840.14) | | | 4826.09(528.47,11475.37) | | 2336.48(296.76,5510.62) | | 2489.61(217.89,5859.71) | |
|  |  | Rate95%(UI) | | 16.1(1.69,38.97) | | 13.64(1.47,33.36) | | 19.52(1.91,47.66) | | 3.73(-0.67,13.31) | | 2.84(-0.56,10.39) | | 4.96(-0.84,17.39) | | 11.25(-2.68,39.26) | | 10.11(-1.94,36.01) | | 13.06(-3.91,46.32) | | 24.47(4.58,47.96) | | 20.75(3.22,41.9) | | 28.81(5.91,57.12) | | 43.28(12.29,79.68) | | 37.68(10.4,69.15) | | 50.4(14.62,95.62) | | | 20.26(2.22,48.17) | | 17.22(2.19,40.61) | | 24.28(2.13,57.16) | |
| 85-89 years | | Number95%(UI) | | 10702.78(396.54,28467.95) | | 5267.37(161.2,14397.1) | | 5435.41(235.34,14405.57) | | 1096.93(-224.86,4090.25) | | 543.98(-121.39,2051.05) | | 552.95(-108.07,2015.67) | | 2278.25(-646.92,8307.96) | | 1262.89(-300.94,4723.46) | | 1015.36(-362.01,3652.43) | | 425.13(74.83,831.83) | | 189.39(28.49,380.81) | | 235.74(44.87,472.04) | | 2829.78(795.41,5312.28) | | 1311.71(357.43,2496.45) | | 1518.07(430.58,2876.72) | | | 4065.96(295.88,10144.35) | | 1955.36(154.31,4837.01) | | 2110.6(141.57,5240.93) | |
|  |  | Rate95%(UI) | | 23.41(0.87,62.26) | | 18.5(0.57,50.57) | | 31.5(1.36,83.5) | | 6.65(-1.36,24.79) | | 5.27(-1.18,19.87) | | 8.96(-1.75,32.65) | | 20.02(-5.69,73.02) | | 16.96(-4.04,63.42) | | 25.84(-9.21,92.94) | | 31.85(5.61,62.31) | | 25.45(3.83,51.17) | | 39.9(7.6,79.9) | | 56.63(15.92,106.31) | | 45.73(12.46,87.02) | | 71.32(20.23,135.16) | | | 35.46(2.58,88.46) | | 27.72(2.19,68.56) | | 47.83(3.21,118.76) | |
| 90-94 years | | Number95%(UI) | | 5797.14(-54.05,16473.61) | | 3174.79(-95.73,9279.14) | | 2622.35(34.28,7243.74) | | 899.24(-188.52,3419.05) | | 526.36(-119.12,2039.35) | | 372.88(-72.17,1375.34) | | 1394.47(-400.86,5187.53) | | 892.22(-226.32,3378.03) | | 502.25(-166.95,1898.91) | | 156.31(26.7,319.11) | | 71.66(11.12,148.62) | | 84.64(14.64,169.24) | | 1317.2(350.32,2486.31) | | 607.39(180.05,1122.21) | | 709.81(176.8,1417.64) | | | 2026.23(131.49,5065.19) | | 1074.82(57.7,2712.32) | | 951.41(72.95,2357.93) | |
|  |  | Rate95%(UI) | | 32.41(-0.3,92.09) | | 26.32(-0.79,76.94) | | 44.99(0.59,124.28) | | 11.73(-2.46,44.6) | | 10.02(-2.27,38.82) | | 15.45(-2.99,56.99) | | 31.9(-9.17,118.66) | | 28.8(-7.31,109.05) | | 39.42(-13.1,149.05) | | 43.6(7.45,89.02) | | 35.14(5.45,72.87) | | 54.78(9.47,109.53) | | 81.4(21.65,153.64) | | 64.12(19.01,118.47) | | 105.79(26.35,211.28) | | | 52.56(3.41,131.38) | | 42.22(2.27,106.54) | | 72.65(5.57,180.05) | |
| 95+ years | | Number95%(UI) | | 2142.73(-93.36,6316.41) | | 1407.79(-101.32,4312.68) | | 734.93(10.89,2003.73) | | 500.51(-118.92,1930.08) | | 350.19(-86.93,1371.9) | | 150.32(-33.18,556.79) | | 479.66(-154.27,1823.88) | | 359.64(-115.32,1392.54) | | 120.02(-39.98,446.77) | | 41.1(7.43,85.77) | | 22.88(3.97,47.37) | | 18.22(3.33,37.31) | | 470.03(119.48,926.6) | | 240.11(74.99,446.49) | | 229.93(46.95,467.06) | | | 649.9(45.89,1613.75) | | 433.94(20.94,1134.52) | | 215.96(27.71,492.38) | |
|  |  | Rate95%(UI) | | 39.31(-1.71,115.89) | | 35.75(-2.57,109.51) | | 48.61(0.72,132.52) | | 18.07(-4.29,69.67) | | 16.99(-4.22,66.56) | | 21.2(-4.68,78.51) | | 43.45(-13.98,165.23) | | 43.47(-13.94,168.32) | | 43.4(-14.46,161.55) | | 54.02(9.77,112.74) | | 47.18(8.19,97.67) | | 66.06(12.07,135.29) | | 103.65(26.35,204.33) | | 82.75(25.84,153.88) | | 140.78(28.74,285.97) | | | 62.47(4.41,155.11) | | 61.39(2.96,160.51) | | 64.75(8.31,147.62) | |
| Age-standardized | | Rate95%(UI) | | 1.34(0.2,3.07) | | 1(0.15,2.32) | | 1.74(0.26,4.01) | | 0.45(0,1.31) | | 0.28(-0.01,0.85) | | 0.64(0,1.83) | | 0.7(-0.18,2.45) | | 0.56(-0.12,2.01) | | 0.89(-0.26,3.15) | | 1.71(0.26,3.36) | | 1.39(0.19,2.74) | | 2.06(0.35,4.09) | | 3.49(0.96,6.57) | | 2.71(0.74,5.02) | | 4.37(1.18,8.38) | | | 1.51(0.24,3.47) | | 1.15(0.18,2.61) | | 1.96(0.29,4.43) | |

| **Table S10. Age-specific death numbers and ASMR for ischemic heart disease attributable to low temperature, categorized by 5-year age groups, presented globally and by SDI region in 2021.** | | | | | | | | | | | | | | | | | | | | |
| --- | --- | --- | --- | --- | --- | --- | --- | --- | --- | --- | --- | --- | --- | --- | --- | --- | --- | --- | --- | --- |
|  | |  | Global | | | High SDI | | | High-middle SDI | | | Low SDI | | | Low-middle SDI | | | Middle SDI | | |
| age | | metric | Both | Female | Male | Both | Female | Male | Both | Female | Male | Both | Female | Male | Both | Female | Male | Both | Female | Male |
| 15-19 years | | Number95%(UI) | 436.65(351.69,549.88) | 194.27(148.86,250.37) | 242.38(199.1,310.35) | 10.65(8.87,13.29) | 3.19(2.53,4.06) | 7.46(6.27,9.39) | 36.14(30,45.63) | 11.04(8.93,14.08) | 25.1(20.52,31.67) | 72.59(54.99,91.65) | 39.09(28.79,50.31) | 33.51(26.18,43.08) | 191.14(142.65,256.28) | 92.89(64.89,125.94) | 98.25(73.3,135.81) | 125.93(105.9,155.05) | 47.98(38.23,60.61) | 77.95(66.07,96.9) |
|  |  | Rate95%(UI) | 0.07(0.06,0.09) | 0.06(0.05,0.08) | 0.08(0.06,0.1) | 0.02(0.01,0.02) | 0.01(0.01,0.01) | 0.02(0.02,0.03) | 0.05(0.04,0.06) | 0.03(0.03,0.04) | 0.07(0.05,0.08) | 0.06(0.04,0.07) | 0.06(0.05,0.08) | 0.05(0.04,0.07) | 0.1(0.08,0.14) | 0.1(0.07,0.14) | 0.1(0.08,0.14) | 0.07(0.06,0.09) | 0.05(0.04,0.07) | 0.08(0.07,0.1) |
| 20-24 years | | Number95%(UI) | 867.82(690.21,1101.49) | 319.7(242.96,412.06) | 548.12(440.04,698.85) | 30.09(24.86,37.42) | 8.2(6.42,10.61) | 21.89(18.18,27.25) | 83.43(70.19,104.81) | 21.7(17.21,28.02) | 61.73(50.76,77.12) | 115.66(88.06,147.09) | 55.54(39.87,73.76) | 60.12(45.82,79.22) | 377.87(272.34,504.73) | 148.14(100.59,209.58) | 229.73(169.23,312.94) | 260.43(218.86,320.22) | 85.99(68.7,108.11) | 174.45(145.79,214.25) |
|  |  | Rate95%(UI) | 0.15(0.12,0.18) | 0.11(0.08,0.14) | 0.18(0.15,0.23) | 0.05(0.04,0.06) | 0.03(0.02,0.03) | 0.06(0.05,0.08) | 0.11(0.09,0.14) | 0.06(0.05,0.08) | 0.16(0.13,0.2) | 0.11(0.08,0.14) | 0.11(0.08,0.14) | 0.12(0.09,0.15) | 0.22(0.16,0.29) | 0.17(0.12,0.24) | 0.26(0.19,0.36) | 0.15(0.12,0.18) | 0.1(0.08,0.12) | 0.19(0.16,0.24) |
| 25-29 years | | Number95%(UI) | 1408.64(1127.71,1780.65) | 456.23(346.6,586.73) | 952.4(770.43,1205.61) | 71.57(56.88,91.18) | 17.04(13.12,21.89) | 54.54(43.32,69.39) | 164.26(141.51,199.37) | 34.18(27.62,43.95) | 130.08(111.72,159.14) | 151.73(116.63,193.7) | 61.89(44.24,81.24) | 89.83(67.78,119.8) | 568.24(413.44,753.92) | 207.52(142.02,287.19) | 360.72(262.89,486.1) | 452.23(379.05,555.97) | 135.41(106.19,170.84) | 316.82(266.41,382.65) |
|  |  | Rate95%(UI) | 0.24(0.19,0.3) | 0.16(0.12,0.2) | 0.32(0.26,0.41) | 0.1(0.08,0.13) | 0.05(0.04,0.06) | 0.15(0.12,0.19) | 0.19(0.17,0.24) | 0.08(0.07,0.11) | 0.29(0.25,0.36) | 0.18(0.14,0.22) | 0.14(0.1,0.18) | 0.21(0.16,0.28) | 0.35(0.26,0.47) | 0.26(0.17,0.35) | 0.45(0.33,0.6) | 0.25(0.21,0.3) | 0.15(0.12,0.19) | 0.34(0.29,0.41) |
| 30-34 years | | Number95%(UI) | 2894.22(2353.49,3641.98) | 800.13(621.78,1016.78) | 2094.1(1711.13,2635.57) | 182.77(148.39,238.44) | 40.74(32.02,52.79) | 142.02(114.89,186.21) | 449.05(388.05,548.74) | 85.33(69.12,108.71) | 363.71(314.15,445.56) | 264.89(206.41,334.83) | 98.27(69.52,129.33) | 166.61(125.44,216.33) | 1043.99(741.28,1392.17) | 332.03(233.28,450.1) | 711.96(514.49,943.03) | 952.33(804.81,1162.04) | 243.39(194.41,303.73) | 708.93(600.39,860.23) |
|  |  | Rate95%(UI) | 0.48(0.39,0.6) | 0.27(0.21,0.34) | 0.69(0.56,0.86) | 0.24(0.19,0.31) | 0.11(0.09,0.14) | 0.35(0.29,0.46) | 0.42(0.36,0.51) | 0.17(0.13,0.21) | 0.66(0.57,0.81) | 0.37(0.29,0.46) | 0.26(0.19,0.35) | 0.47(0.36,0.61) | 0.71(0.5,0.94) | 0.45(0.32,0.61) | 0.96(0.7,1.28) | 0.48(0.4,0.58) | 0.25(0.2,0.31) | 0.7(0.6,0.85) |
| 35-39 years | | Number95%(UI) | 4553.48(3723.18,5648.49) | 1205.74(946.13,1506.14) | 3347.74(2751.98,4197.06) | 372.86(309.57,472.3) | 78.63(63.03,99.76) | 294.23(242.64,371.05) | 794.83(688.18,970.74) | 146.89(120.92,183.55) | 647.94(550.8,798.28) | 393.34(306.41,496.64) | 137.7(100.68,180.16) | 255.64(196.37,327.79) | 1507.11(1084.8,2008.57) | 488.01(339.45,659.22) | 1019.1(741.59,1371.04) | 1483.15(1259.5,1814.31) | 353.95(291.45,438.46) | 1129.2(944.33,1384.49) |
|  |  | Rate95%(UI) | 0.81(0.66,1.01) | 0.43(0.34,0.54) | 1.18(0.97,1.48) | 0.47(0.39,0.6) | 0.21(0.17,0.26) | 0.72(0.6,0.91) | 0.78(0.68,0.96) | 0.3(0.24,0.37) | 1.25(1.06,1.54) | 0.63(0.49,0.8) | 0.43(0.32,0.57) | 0.84(0.65,1.08) | 1.13(0.81,1.51) | 0.73(0.51,0.99) | 1.52(1.11,2.05) | 0.8(0.68,0.98) | 0.39(0.32,0.48) | 1.21(1.02,1.49) |
| 40-44 years | | Number95%(UI) | 7848.19(6448.83,9818.81) | 2041.87(1636.94,2578.28) | 5806.32(4765.93,7322.81) | 730.25(603.89,908.29) | 153(126.54,193.08) | 577.25(475.48,723.96) | 1401.97(1212.3,1724.2) | 269.19(224.37,335.7) | 1132.78(971.25,1420.97) | 683.63(534.87,860.55) | 220.02(162.27,283.67) | 463.61(357.14,595.16) | 2591.19(1914.85,3426.73) | 775.76(547.69,1046.33) | 1815.43(1334.54,2438.44) | 2436.91(2033.18,3009.68) | 622.86(505.54,766.04) | 1814.05(1517.21,2249.75) |
|  |  | Rate95%(UI) | 1.57(1.29,1.96) | 0.82(0.66,1.04) | 2.3(1.89,2.9) | 0.97(0.8,1.2) | 0.42(0.34,0.53) | 1.48(1.22,1.86) | 1.52(1.31,1.87) | 0.59(0.49,0.74) | 2.42(2.07,3.03) | 1.32(1.04,1.67) | 0.84(0.62,1.09) | 1.82(1.4,2.33) | 2.25(1.66,2.97) | 1.34(0.95,1.81) | 3.15(2.32,4.23) | 1.48(1.23,1.82) | 0.76(0.62,0.94) | 2.18(1.83,2.71) |
| 45-49 years | | Number95%(UI) | 12210.17(10094.25,15155.57) | 3337.74(2652.64,4184.27) | 8872.43(7347.59,11056.71) | 1263.25(1094.16,1551.59) | 267.46(227.3,331.07) | 995.79(861.31,1216.75) | 2390.53(2062.6,2936.77) | 493.64(413.91,625.46) | 1896.89(1611.71,2348.81) | 1017.82(798.46,1293.87) | 332.58(247.28,430.37) | 685.24(526.48,880.01) | 3614.5(2631.56,4731.55) | 1127.44(818.17,1495.86) | 2487.06(1822.05,3275.85) | 3916.54(3294.35,4802.46) | 1114.82(900.15,1385.13) | 2801.72(2356.75,3430.92) |
|  |  | Rate95%(UI) | 2.58(2.13,3.2) | 1.42(1.13,1.78) | 3.73(3.09,4.65) | 1.72(1.49,2.11) | 0.74(0.63,0.92) | 2.66(2.3,3.25) | 2.47(2.13,3.03) | 1.02(0.86,1.3) | 3.9(3.31,4.83) | 2.44(1.91,3.1) | 1.6(1.19,2.07) | 3.27(2.52,4.2) | 3.67(2.67,4.8) | 2.28(1.65,3.03) | 5.07(3.71,6.68) | 2.41(2.03,2.95) | 1.37(1.11,1.71) | 3.44(2.89,4.21) |
| 50-54 years | | Number95%(UI) | 19295.88(16273.58,23778.26) | 5217.99(4287.19,6440.83) | 14077.89(11754.39,17230.33) | 2162.46(1929.35,2601.61) | 462.26(407.31,561.14) | 1700.2(1517.28,2044.58) | 4052.65(3490.24,4947.33) | 895.95(742.91,1114.61) | 3156.7(2695,3900.57) | 1377.27(1074.33,1745.08) | 442.26(331.17,568.69) | 935.01(718.52,1202.87) | 5324.6(3930.9,6977.87) | 1631.91(1202.72,2167.67) | 3692.68(2723.64,4879.13) | 6366.66(5379.83,7662.45) | 1782.69(1469.88,2159.44) | 4583.97(3853.55,5536.62) |
|  |  | Rate95%(UI) | 4.34(3.66,5.34) | 2.34(1.92,2.89) | 6.34(5.3,7.76) | 2.91(2.6,3.5) | 1.26(1.11,1.52) | 4.54(4.05,5.46) | 4.18(3.6,5.11) | 1.85(1.53,2.3) | 6.52(5.57,8.06) | 4.21(3.28,5.33) | 2.72(2.03,3.49) | 5.69(4.37,7.32) | 6.34(4.68,8.31) | 3.86(2.84,5.13) | 8.87(6.54,11.72) | 4.06(3.43,4.89) | 2.26(1.86,2.74) | 5.89(4.95,7.11) |
| 55-59 years | | Number95%(UI) | 28639.26(24111.61,35399.31) | 8802.61(7165.83,10890.22) | 19836.65(16781.83,24468.09) | 3440.59(3102.42,4084.19) | 782.82(693.75,937.81) | 2657.77(2404.16,3142.86) | 6608.35(5698.28,8122.14) | 1745.36(1454.73,2206.08) | 4862.98(4177.94,6071.69) | 1803.79(1398.23,2280.65) | 668.62(495.23,845.81) | 1135.17(877.78,1469.03) | 7537.61(5638.13,9938.33) | 2578.97(1901.63,3394.49) | 4958.64(3684.69,6515.96) | 9230.9(7809.77,11174.16) | 3022.22(2472.17,3752.85) | 6208.68(5180.15,7502.07) |
|  |  | Rate95%(UI) | 7.24(6.09,8.95) | 4.38(3.57,5.42) | 10.19(8.62,12.57) | 4.73(4.27,5.62) | 2.14(1.9,2.57) | 7.34(6.64,8.68) | 7.35(6.34,9.03) | 3.8(3.17,4.81) | 11.04(9.48,13.78) | 7.12(5.52,9) | 5.25(3.89,6.64) | 9.01(6.97,11.66) | 10.85(8.12,14.31) | 7.3(5.39,9.61) | 14.52(10.79,19.08) | 6.69(5.66,8.1) | 4.29(3.51,5.33) | 9.19(7.67,11.11) |
| 60-64 years | | Number95%(UI) | 37689.77(32058.81,46713.61) | 12354.34(10361.84,15210.02) | 25335.43(21476.61,31234.83) | 5052.88(4572.63,6001.77) | 1276.24(1139.73,1530.22) | 3776.64(3429.21,4473.87) | 10179.59(8908,12489.82) | 3107.33(2644.09,3916.28) | 7072.27(6100.6,8816.56) | 2141.14(1674.77,2682.63) | 783.99(585.23,996.46) | 1357.15(1036.84,1717.14) | 8807.3(6716.89,11364.53) | 3216.22(2400.34,4112.62) | 5591.08(4204.99,7344.32) | 11480.59(9708.27,14038.65) | 3962.48(3325.27,4828.18) | 7518.11(6335.81,9155.73) |
|  |  | Rate95%(UI) | 11.78(10.02,14.6) | 7.51(6.3,9.25) | 16.29(13.81,20.08) | 7.41(6.71,8.8) | 3.66(3.27,4.39) | 11.33(10.29,13.42) | 13.96(12.21,17.12) | 8.18(6.96,10.31) | 20.24(17.46,25.23) | 10.86(8.49,13.61) | 7.86(5.87,9.99) | 13.93(10.64,17.63) | 15.41(11.75,19.88) | 10.94(8.16,13.99) | 20.14(15.15,26.46) | 11.28(9.54,13.79) | 7.6(6.37,9.26) | 15.15(12.77,18.45) |
| 65-69 years | | Number95%(UI) | 50481.39(43389.28,61992.07) | 18768.54(15784.29,23014.99) | 31712.85(27080.83,38409.88) | 6680.1(6016.74,7962.74) | 1901.45(1658.86,2292.4) | 4778.65(4327.11,5684.73) | 14689.99(12734.28,17829.14) | 5481.39(4636.65,6834.99) | 9208.6(7898.98,11369.21) | 2681.86(2097.49,3390.86) | 1091.64(825.67,1370.13) | 1590.23(1230.41,2027.22) | 10417.02(7875.51,13435.46) | 4191.48(3159.39,5369.81) | 6225.54(4653.42,8214.81) | 15972.44(13590.3,19211.04) | 6089.52(5086.27,7399.03) | 9882.92(8279.88,11852.27) |
|  |  | Rate95%(UI) | 18.3(15.73,22.47) | 13.03(10.96,15.98) | 24.06(20.54,29.14) | 11.02(9.92,13.13) | 6.02(5.25,7.26) | 16.45(14.89,19.57) | 22.12(19.18,26.85) | 15.46(13.07,19.27) | 29.76(25.53,36.75) | 17.79(13.91,22.49) | 14.21(10.75,17.83) | 21.51(16.64,27.42) | 23.48(17.75,30.29) | 18.21(13.73,23.33) | 29.16(21.8,38.48) | 17.92(15.25,21.55) | 13.19(11.02,16.03) | 22.99(19.26,27.57) |
| 70-74 years | | Number95%(UI) | 60471.9(52368.61,73679.27) | 24749.26(21159.2,30497.07) | 35722.64(30577,43099.86) | 8938.81(8013.72,10594.1) | 2950.53(2548.97,3536.46) | 5988.28(5420.91,7056.17) | 18786.88(16323.91,23097.83) | 8312.25(7016.32,10415.21) | 10474.63(9013.16,12991.27) | 3080.31(2358.5,3863.21) | 1263.9(960.58,1611.25) | 1816.41(1381.45,2286.31) | 11031.31(8343.11,14358.29) | 4674.12(3486.4,6060.2) | 6357.19(4756.08,8264.13) | 18584.62(15864.8,22461.05) | 7528.59(6308.72,9038.88) | 11056.03(9211.65,13430.18) |
|  |  | Rate95%(UI) | 29.38(25.44,35.79) | 22.61(19.33,27.86) | 37.06(31.72,44.71) | 16.75(15.02,19.85) | 10.41(9,12.48) | 23.93(21.66,28.19) | 38.46(33.42,47.28) | 30.92(26.1,38.74) | 47.69(41.03,59.14) | 29.7(22.74,37.25) | 23.81(18.1,30.35) | 35.88(27.29,45.16) | 35.08(26.53,45.66) | 28.37(21.16,36.78) | 42.46(31.77,55.2) | 30.16(25.74,36.45) | 23.28(19.5,27.94) | 37.76(31.46,45.86) |
| 75-79 years | | Number95%(UI) | 63103.26(54592.88,76430.71) | 28596.21(24336.88,34876.44) | 34507.05(29510.02,41239.11) | 10322.94(8984.55,12292.34) | 3948.15(3308.66,4753.19) | 6374.79(5685.35,7552.6) | 19600.95(17053.12,24044.66) | 9823.78(8346.4,12323.11) | 9777.17(8327.96,11829.57) | 2692.6(2071.65,3335.59) | 1215.62(929.27,1518.59) | 1476.97(1116.49,1904.88) | 10268.11(7850.24,13298.37) | 4775.6(3588.32,6139.8) | 5492.51(4130.53,7202.64) | 20161.91(17315.23,23711.66) | 8806.01(7247.78,10492.21) | 11355.9(9462.46,13652.03) |
|  |  | Rate95%(UI) | 47.85(41.39,57.95) | 39.66(33.76,48.37) | 57.72(49.36,68.98) | 28.51(24.81,33.94) | 19.86(16.64,23.91) | 39.03(34.81,46.25) | 65.63(57.09,80.5) | 58.12(49.38,72.9) | 75.42(64.24,91.25) | 41.94(32.26,51.95) | 36.56(27.95,45.67) | 47.71(36.07,61.53) | 51.14(39.1,66.23) | 44.42(33.38,57.11) | 58.88(44.28,77.22) | 51.46(44.19,60.52) | 41.6(34.24,49.57) | 63.03(52.52,75.78) |
| 80-84 years | | Number95%(UI) | 79463.28(67173.07,96348.17) | 40989.27(33453.81,49973.41) | 38474.01(32734.56,46066.64) | 13823.63(11179.5,16620.21) | 6227.6(4790.65,7672.05) | 7596.03(6403.8,9022.56) | 30906.37(26036.76,37713.6) | 18008.66(14703.23,22427.18) | 12897.71(11029.22,15788.13) | 2263.58(1708.31,2860.72) | 1020.39(777.61,1309.64) | 1243.19(926.9,1623.93) | 9151.17(7006.85,11808.25) | 4556.75(3457.37,5861.67) | 4594.42(3478.08,6084.62) | 23241(19389.33,27274.54) | 11132.36(9022.11,13204.94) | 12108.65(10206.1,14403.3) |
|  |  | Rate95%(UI) | 90.73(76.7,110.01) | 80.48(65.68,98.12) | 104.97(89.31,125.69) | 51.25(41.45,61.62) | 39.82(30.63,49.06) | 67.02(56.5,79.61) | 141.56(119.26,172.74) | 134.2(109.57,167.13) | 153.3(131.09,187.65) | 67.11(50.64,84.81) | 56.19(42.82,72.12) | 79.84(59.53,104.29) | 79.57(60.93,102.68) | 70.77(53.7,91.04) | 90.77(68.71,120.21) | 97.57(81.4,114.5) | 82.05(66.49,97.32) | 118.11(99.55,140.49) |
| 85-89 years | | Number95%(UI) | 72018.68(59008.82,86182.43) | 38338.54(29773.85,47147.42) | 33680.14(28762.03,40033.35) | 16462.34(12772.4,19981.73) | 8526.51(6250.68,10467.1) | 7935.84(6521.6,9473.83) | 27868.67(22590.58,34144.93) | 16477.23(13075.42,20728.38) | 11391.44(9687.93,13559.71) | 1174.7(893.59,1476.11) | 515.4(378.66,650.1) | 659.3(507.52,843.06) | 5349.54(4007.47,6891.13) | 2611.08(1950.21,3296.07) | 2738.46(2066.34,3593.78) | 21081.42(17353.19,24785.16) | 10157.25(7853.39,12288.77) | 10924.17(9284.56,13067.98) |
|  |  | Rate95%(UI) | 157.52(129.06,188.49) | 134.67(104.58,165.61) | 195.22(166.71,232.04) | 99.78(77.42,121.12) | 82.59(60.54,101.38) | 128.54(105.63,153.45) | 244.95(198.56,300.12) | 221.25(175.57,278.33) | 289.87(246.52,345.04) | 87.99(66.94,110.57) | 69.26(50.88,87.36) | 111.6(85.91,142.7) | 107.05(80.2,137.9) | 91.02(67.98,114.9) | 128.66(97.08,168.85) | 183.83(151.32,216.13) | 143.97(111.32,174.19) | 247.55(210.39,296.13) |
| 90-94 years | | Number95%(UI) | 45546.75(35660.07,55577.81) | 27731.42(20935.22,34493.5) | 17815.33(14686.92,21277.78) | 13943.26(10455.48,17088.83) | 8562.99(6135.26,10577.4) | 5380.27(4344.95,6470.48) | 17726.84(14130.11,21818.92) | 11802.69(9179.39,14835.13) | 5924.15(4951.26,7150.13) | 436.84(327.76,550.79) | 194.34(143.16,248.91) | 242.5(181.33,309.1) | 2513.13(1849.65,3262.55) | 1201.04(857.59,1557.87) | 1312.09(983.32,1752.17) | 10877.12(8697.06,12792.07) | 5936.87(4475.26,7174.77) | 4940.25(4097.5,5860.53) |
|  |  | Rate95%(UI) | 254.6(199.34,310.68) | 229.93(173.58,286) | 305.66(251.98,365.06) | 181.87(136.38,222.9) | 163(116.79,201.34) | 222.95(180.05,268.13) | 405.48(323.21,499.09) | 381(296.32,478.89) | 465.01(388.64,561.24) | 121.87(91.44,153.65) | 95.29(70.2,122.05) | 156.94(117.35,200.05) | 155.3(114.3,201.61) | 126.79(90.53,164.46) | 195.55(146.55,261.14) | 282.13(225.58,331.8) | 233.21(175.79,281.83) | 377.22(312.87,447.49) |
| 95+ years | | Number95%(UI) | 18368.18(13191.93,22577.15) | 13281.02(9386.81,16374.56) | 5087.17(3874.03,6182.24) | 7846.66(5469.77,9737.92) | 5704.32(3890.4,7103.16) | 2142.34(1572.33,2627.41) | 6035.92(4422.8,7488.33) | 4591.06(3332.94,5714.39) | 1444.86(1133.21,1756.11) | 110.49(81.15,142.34) | 59.65(41.81,76.59) | 50.84(37.09,65.53) | 865(604.48,1139.27) | 453.76(311.61,601.95) | 411.24(293.77,548.32) | 3489.43(2578.53,4239.6) | 2456.93(1779.46,3048.26) | 1032.5(796.77,1239.16) |
|  |  | Rate95%(UI) | 337.01(242.04,414.24) | 337.23(238.35,415.78) | 336.44(256.21,408.87) | 283.25(197.45,351.52) | 276.77(188.76,344.64) | 302.09(221.71,370.48) | 546.8(400.66,678.37) | 554.94(402.86,690.72) | 522.45(409.76,635) | 145.23(106.66,187.09) | 122.98(86.2,157.9) | 184.37(134.52,237.62) | 190.74(133.3,251.23) | 156.38(107.39,207.45) | 251.79(179.87,335.72) | 335.4(247.85,407.51) | 347.6(251.75,431.25) | 309.56(238.89,371.53) |
| Age-standardized | | Rate95%(UI) | 6.14(5.23,7.53) | 4.85(4.05,5.97) | 7.71(6.6,9.29) | 3.84(3.21,4.6) | 2.67(2.12,3.25) | 5.25(4.56,6.24) | 8.42(7.17,10.32) | 6.97(5.7,8.68) | 10.25(8.85,12.46) | 4.79(3.74,5.92) | 3.78(2.91,4.73) | 5.9(4.6,7.4) | 6.24(4.79,8.06) | 4.9(3.68,6.29) | 7.75(5.86,10.25) | 6.44(5.45,7.62) | 4.97(4.1,5.94) | 8.27(6.97,9.88) |
| ASMR: Age-standardized mortality rates. SDI: Sociodemographic Index. | | | | | | | | | | | | | | | | | | | | |

| **Table S11. Changes in DALYs and deaths for myocardial disease, and ischemic heart disease attributable to high and low temperatures from 1990 to 2021, categorized globally and by SDI regions.** | | | | | | | | | | | |
| --- | --- | --- | --- | --- | --- | --- | --- | --- | --- | --- | --- |
| Location | Sex | Cause | Risk | Measure | Overll difference | Aging | Population | Epidemiological change | Change of Aging (%) | Change of Population (%) | Change of Epidemiological change (%) |
| Global | Both | Myocardial disease | High temperature | DALYs | 79905.07 | 13855.84 | 43780.42 | 22268.81 | 17.34 | 54.79 | 27.87 |
| Global | Both | Myocardial disease | Low temperature | DALYs | 84356.35 | 141389.22 | 217113.42 | -274146.29 | 167.61 | 257.38 | -324.99 |
| Global | Both | Ischemic heart disease | High temperature | DALYs | 1809955.95 | 555589.42 | 606917.01 | 647449.52 | 30.70 | 33.53 | 35.77 |
| Global | Both | Ischemic heart disease | Low temperature | DALYs | 2993895.01 | 3533271.72 | 3389312.63 | -3928689.34 | 118.02 | 113.21 | -131.22 |
| High-middle SDI | Both | Myocardial disease | High temperature | DALYs | 13101.35 | 3468.31 | 2805.78 | 6827.27 | 26.47 | 21.42 | 52.11 |
| High-middle SDI | Both | Myocardial disease | Low temperature | DALYs | 67101.52 | 75976.77 | 41680.60 | -50555.85 | 113.23 | 62.12 | -75.34 |
| High-middle SDI | Both | Ischemic heart disease | High temperature | DALYs | 162531.22 | 74219.03 | 30444.23 | 57867.95 | 45.66 | 18.73 | 35.60 |
| High-middle SDI | Both | Ischemic heart disease | Low temperature | DALYs | 621260.41 | 1375930.62 | 532465.08 | -1287135.29 | 221.47 | 85.71 | -207.18 |
| High SDI | Both | Myocardial disease | High temperature | DALYs | 2848.63 | 3657.21 | 3762.44 | -4571.03 | 128.39 | 132.08 | -160.46 |
| High SDI | Both | Myocardial disease | Low temperature | DALYs | -31229.03 | 47849.05 | 32519.38 | -111597.46 | -153.22 | -104.13 | 357.35 |
| High SDI | Both | Ischemic heart disease | High temperature | DALYs | 95321.54 | 55283.88 | 34415.44 | 5622.21 | 58.00 | 36.10 | 5.90 |
| High SDI | Both | Ischemic heart disease | Low temperature | DALYs | -556951.47 | 866218.56 | 425570.64 | -1848740.67 | -155.53 | -76.41 | 331.94 |
| Low-middle SDI | Both | Myocardial disease | High temperature | DALYs | 38005.99 | 2156.78 | 21022.54 | 14826.67 | 5.67 | 55.31 | 39.01 |
| Low-middle SDI | Both | Myocardial disease | Low temperature | DALYs | 32635.31 | 7698.99 | 37882.36 | -12946.05 | 23.59 | 116.08 | -39.67 |
| Low-middle SDI | Both | Ischemic heart disease | High temperature | DALYs | 810164.41 | 233800.45 | 342509.74 | 233854.22 | 28.86 | 42.28 | 28.87 |
| Low-middle SDI | Both | Ischemic heart disease | Low temperature | DALYs | 1080913.73 | 493179.73 | 720999.64 | -133265.64 | 45.63 | 66.70 | -12.33 |
| Low SDI | Both | Myocardial disease | High temperature | DALYs | 11666.88 | -98.07 | 7050.14 | 4714.81 | -0.84 | 60.43 | 40.41 |
| Low SDI | Both | Myocardial disease | Low temperature | DALYs | 11727.30 | -1051.50 | 24938.25 | -12159.46 | -8.97 | 212.65 | -103.69 |
| Low SDI | Both | Ischemic heart disease | High temperature | DALYs | 124286.78 | 4031.33 | 85677.17 | 34578.28 | 3.24 | 68.94 | 27.82 |
| Low SDI | Both | Ischemic heart disease | Low temperature | DALYs | 244730.76 | 14212.22 | 317416.52 | -86897.98 | 5.81 | 129.70 | -35.51 |
| Middle SDI | Both | Myocardial disease | Low temperature | DALYs | 4037.28 | 17805.46 | 31203.64 | -44971.82 | 441.03 | 772.89 | -1113.91 |
| Middle SDI | Both | Ischemic heart disease | High temperature | DALYs | 616922.24 | 255874.31 | 158161.10 | 202886.82 | 41.48 | 25.64 | 32.89 |
| Middle SDI | Both | Ischemic heart disease | Low temperature | DALYs | 1605416.81 | 1319621.74 | 773725.83 | -487930.76 | 82.20 | 48.19 | -30.39 |
| Global | Both | Myocardial disease | High temperature | Deaths | 3217.29 | 1131.74 | 1309.21 | 776.34 | 35.18 | 40.69 | 24.13 |
| Global | Both | Myocardial disease | Low temperature | Deaths | 4642.07 | 9521.19 | 8256.99 | -13136.11 | 205.11 | 177.87 | -282.98 |
| Global | Both | Ischemic heart disease | High temperature | Deaths | 79820.77 | 28685.26 | 25352.30 | 25783.20 | 35.94 | 31.76 | 32.30 |
| Global | Both | Ischemic heart disease | Low temperature | Deaths | 179709.76 | 214444.96 | 166362.43 | -201097.64 | 119.33 | 92.57 | -111.90 |
| High-middle SDI | Both | Myocardial disease | High temperature | Deaths | 542.76 | 255.08 | 102.13 | 185.55 | 47.00 | 18.82 | 34.19 |
| High-middle SDI | Both | Myocardial disease | Low temperature | Deaths | 1917.63 | 4881.30 | 1674.01 | -4637.67 | 254.55 | 87.30 | -241.84 |
| High-middle SDI | Both | Ischemic heart disease | High temperature | Deaths | 9756.69 | 4696.55 | 1578.42 | 3481.73 | 48.14 | 16.18 | 35.69 |
| High-middle SDI | Both | Ischemic heart disease | Low temperature | Deaths | 54132.40 | 88618.07 | 28320.51 | -62806.19 | 163.71 | 52.32 | -116.02 |
| High SDI | Both | Myocardial disease | High temperature | Deaths | 172.15 | 277.37 | 147.19 | -252.40 | 161.12 | 85.50 | -146.62 |
| High SDI | Both | Myocardial disease | Low temperature | Deaths | -372.16 | 3628.95 | 1577.68 | -5578.79 | -975.11 | -423.93 | 1499.04 |
| High SDI | Both | Ischemic heart disease | High temperature | Deaths | 3475.48 | 3773.23 | 1659.67 | -1957.42 | 108.57 | 47.75 | -56.32 |
| High SDI | Both | Ischemic heart disease | Low temperature | Deaths | -21445.73 | 64044.39 | 24824.34 | -110314.46 | -298.63 | -115.75 | 514.39 |
| Low SDI | Both | Myocardial disease | High temperature | Deaths | 364.15 | 8.38 | 199.74 | 156.02 | 2.30 | 54.85 | 42.85 |
| Low SDI | Both | Myocardial disease | Low temperature | Deaths | 440.05 | 12.59 | 624.97 | -197.51 | 2.86 | 142.02 | -44.88 |
| Low SDI | Both | Ischemic heart disease | High temperature | Deaths | 5060.40 | 206.49 | 3303.47 | 1550.44 | 4.08 | 65.28 | 30.64 |
| Low SDI | Both | Ischemic heart disease | Low temperature | Deaths | 10143.12 | 685.60 | 11976.95 | -2519.43 | 6.76 | 118.08 | -24.84 |
| Low-middle SDI | Both | Myocardial disease | High temperature | Deaths | 1302.50 | 283.00 | 548.23 | 471.28 | 21.73 | 42.09 | 36.18 |
| Low-middle SDI | Both | Myocardial disease | Low temperature | Deaths | 1446.92 | 604.66 | 1049.25 | -206.99 | 41.79 | 72.52 | -14.31 |
| Low-middle SDI | Both | Ischemic heart disease | High temperature | Deaths | 32671.56 | 10401.22 | 13064.30 | 9206.05 | 31.84 | 39.99 | 28.18 |
| Low-middle SDI | Both | Ischemic heart disease | Low temperature | Deaths | 45466.40 | 22362.29 | 27865.12 | -4761.00 | 49.18 | 61.29 | -10.47 |
| Middle SDI | Both | Myocardial disease | High temperature | Deaths | 834.38 | 375.03 | 284.44 | 174.91 | 44.95 | 34.09 | 20.96 |
| Middle SDI | Both | Myocardial disease | Low temperature | Deaths | 1203.29 | 1340.23 | 913.18 | -1050.12 | 111.38 | 75.89 | -87.27 |
| Middle SDI | Both | Ischemic heart disease | High temperature | Deaths | 28821.95 | 12993.06 | 6723.38 | 9105.50 | 45.08 | 23.33 | 31.59 |
| Middle SDI | Both | Ischemic heart disease | Low temperature | Deaths | 91426.02 | 71860.75 | 35077.45 | -15512.18 | 78.60 | 38.37 | -16.97 |
| Overall Difference: Net change in DALYs or deaths over the study period (1990–2021). Aging: Contribution of population aging to the changes in DALYs or deaths. Population Growth: Contribution of population growth to the changes in DALYs or deaths. Epidemiological Change: Contribution of healthcare improvements, public health interventions, or other epidemiological factors to the changes in DALYs or deaths. Percent Change of Aging, Population Growth, and Epidemiological Change: Proportional contribution of each factor (aging, population growth, and epidemiological change) to the overall change in DALYs or deaths. | | | | | | | | | | | |

| **Table S12. Age-standardized DALYs and mortality rates (ASDR, ASMR) for cardiomyopathy, myocarditis, and ischemic heart disease related to high and low temperatures, from 1992 to projected estimates for 2040.** | | | | | | | | | | | | |
| --- | --- | --- | --- | --- | --- | --- | --- | --- | --- | --- | --- | --- |
| ASDR | Myocardial Disease attributable to High temperature | | | Myocardial Disease attributable to  Low temperature | | | Ischemic Heart Disease attributable to  High temperature | | | Ischemic Heart Disease attributable to  Low temperature | | |
| Year | Both | Female | Male | Both | Female | Male | Both | Female | Male | Both | Female | Male |
| 1992 | 1.525984 | 1.258193 | 1.827059 | 12.634261 | 9.535201 | 15.842882 | 28.436123 | 21.471478 | 36.252852 | 278.009915 | 214.33812 | 347.343753 |
| 1993 | 1.677757 | 1.374852 | 2.020519 | 12.754745 | 9.510844 | 16.081844 | 30.635166 | 22.925058 | 39.328598 | 279.733933 | 215.074677 | 349.492783 |
| 1994 | 1.886049 | 1.531747 | 2.281284 | 12.168515 | 8.93785 | 15.478138 | 32.513089 | 24.405217 | 41.614537 | 265.068065 | 203.150422 | 331.523941 |
| 1995 | 2.071958 | 1.675999 | 2.513478 | 12.381534 | 9.008554 | 15.844085 | 35.886829 | 26.823196 | 45.998802 | 263.869695 | 201.656249 | 330.845742 |
| 1996 | 1.717501 | 1.390362 | 2.081393 | 12.493411 | 9.08121 | 16.003418 | 30.9898 | 23.07627 | 39.81516 | 270.361082 | 206.82022 | 338.631122 |
| 1997 | 1.377708 | 1.125114 | 1.66081 | 11.90052 | 8.634647 | 15.275967 | 29.21679 | 21.798183 | 37.540183 | 256.240568 | 196.263406 | 320.747565 |
| 1998 | 2.372914 | 1.904788 | 2.891063 | 10.934024 | 7.917302 | 14.052247 | 41.76213 | 30.998416 | 53.765235 | 236.617777 | 181.006049 | 296.414277 |
| 1999 | 1.912577 | 1.546073 | 2.319526 | 10.922162 | 7.8906 | 14.055154 | 34.182505 | 25.486888 | 43.805554 | 236.780077 | 181.144407 | 296.443755 |
| 2000 | 1.833452 | 1.477171 | 2.228782 | 11.158535 | 7.935375 | 14.493242 | 33.402954 | 24.641483 | 43.154915 | 238.947448 | 181.452131 | 300.801544 |
| 2001 | 1.692319 | 1.351885 | 2.06598 | 10.713339 | 7.541994 | 13.995422 | 33.182889 | 24.521202 | 42.80986 | 231.610126 | 175.721141 | 291.71494 |
| 2002 | 2.159468 | 1.720435 | 2.642471 | 10.713838 | 7.42224 | 14.112924 | 40.09939 | 29.226763 | 52.158628 | 226.753096 | 171.794821 | 285.882762 |
| 2003 | 2.033632 | 1.628957 | 2.479724 | 11.384165 | 7.834348 | 15.04011 | 37.689015 | 27.331208 | 49.243332 | 236.011853 | 178.118636 | 298.588472 |
| 2004 | 1.849227 | 1.476176 | 2.260772 | 11.311801 | 7.622247 | 15.103835 | 34.103884 | 24.373615 | 44.996978 | 223.083427 | 167.311195 | 283.602991 |
| 2005 | 2.227186 | 1.744124 | 2.751812 | 12.215189 | 8.048414 | 16.483534 | 38.982337 | 27.96964 | 51.230018 | 225.61082 | 168.340965 | 287.855232 |
| 2006 | 2.14208 | 1.669658 | 2.653042 | 11.429811 | 7.448851 | 15.502339 | 37.789493 | 26.994871 | 49.773774 | 207.997923 | 155.44545 | 264.980331 |
| 2007 | 2.282401 | 1.750233 | 2.856507 | 11.330358 | 7.385034 | 15.37882 | 39.595451 | 28.031602 | 52.382008 | 206.315486 | 152.868344 | 264.577969 |
| 2008 | 1.738206 | 1.356465 | 2.15129 | 11.358793 | 7.438497 | 15.384031 | 32.363302 | 22.387032 | 43.406015 | 206.744442 | 152.068432 | 266.395437 |
| 2009 | 2.31139 | 1.809377 | 2.85753 | 10.170341 | 6.694694 | 13.742157 | 42.63564 | 29.636953 | 56.982842 | 196.279652 | 144.696462 | 252.645548 |
| 2010 | 2.850617 | 2.120919 | 3.632187 | 9.861538 | 6.562878 | 13.276433 | 49.019506 | 33.885739 | 65.651882 | 200.744159 | 146.853579 | 259.845622 |
| 2011 | 1.942631 | 1.499919 | 2.423781 | 9.453858 | 6.405266 | 12.62014 | 37.01095 | 25.805041 | 49.37136 | 204.960991 | 149.715242 | 265.531124 |
| 2012 | 2.370688 | 1.845025 | 2.943571 | 8.802649 | 6.021425 | 11.703573 | 45.431401 | 32.023388 | 60.168372 | 201.50728 | 146.917766 | 261.555796 |
| 2013 | 2.016835 | 1.579946 | 2.493196 | 8.795701 | 5.961935 | 11.748847 | 40.952563 | 29.066598 | 54.180191 | 196.554274 | 143.243673 | 255.485484 |
| 2014 | 2.239439 | 1.758306 | 2.763374 | 8.568006 | 5.802652 | 11.454604 | 44.955928 | 31.754654 | 59.70078 | 191.151411 | 139.314464 | 248.579112 |
| 2015 | 2.255779 | 1.748301 | 2.805961 | 8.072433 | 5.409036 | 10.853663 | 44.995974 | 32.042222 | 59.323546 | 178.567703 | 130.175897 | 232.181719 |
| 2016 | 2.592304 | 2.01205 | 3.222284 | 7.606418 | 5.044504 | 10.283131 | 50.390807 | 36.199687 | 66.012461 | 171.737368 | 125.17615 | 223.273989 |
| 2017 | 2.438731 | 1.87379 | 3.048918 | 7.545351 | 4.972449 | 10.231664 | 48.119526 | 34.344064 | 63.209048 | 171.699049 | 124.590282 | 223.720442 |
| 2018 | 2.303561 | 1.75317 | 2.894903 | 7.351637 | 4.846516 | 9.971366 | 46.87623 | 33.351005 | 61.666389 | 170.929446 | 124.021498 | 222.682911 |
| 2019 | 2.636163 | 1.97649 | 3.344957 | 7.373845 | 4.838125 | 10.027597 | 51.452718 | 36.414405 | 67.869514 | 170.370083 | 122.845147 | 222.838517 |
| 2020 | 2.108699 | 1.539884 | 2.719482 | 7.221418 | 4.652184 | 9.912181 | 44.853162 | 31.650843 | 59.276021 | 166.834857 | 120.213443 | 218.438068 |
| 2021 | 1.95564 | 1.419246 | 2.529837 | 6.983848 | 4.469669 | 9.614676 | 42.878127 | 30.359756 | 56.576739 | 164.634904 | 118.891405 | 215.29974 |
| 2022 | 2.305917 | 1.691622 | 2.964628 | 6.764168 | 4.33438 | 9.313783 | 47.604308 | 33.704098 | 62.794376 | 159.014962 | 114.61169 | 208.088997 |
| 2023 | 2.312563 | 1.685237 | 2.984707 | 6.588856 | 4.194757 | 9.102561 | 47.881553 | 33.880395 | 63.180306 | 155.746988 | 112.134997 | 203.957017 |
| 2024 | 2.319277 | 1.678852 | 3.004786 | 6.413699 | 4.055134 | 8.891338 | 48.160917 | 34.056692 | 63.566235 | 152.485477 | 109.658304 | 199.825037 |
| 2025 | 2.324158 | 1.673732 | 3.019857 | 6.304916 | 3.969954 | 8.75783 | 48.343298 | 34.174903 | 63.809295 | 150.454368 | 108.185746 | 197.143081 |
| 2026 | 2.329031 | 1.668612 | 3.034927 | 6.196185 | 3.884774 | 8.624322 | 48.525023 | 34.293114 | 64.052354 | 148.421254 | 106.713189 | 194.461125 |
| 2027 | 2.333874 | 1.663492 | 3.049998 | 6.087444 | 3.799594 | 8.490814 | 48.705525 | 34.411325 | 64.295414 | 146.384001 | 105.240632 | 191.77917 |
| 2028 | 2.338732 | 1.658372 | 3.065069 | 5.978766 | 3.714414 | 8.357305 | 48.886279 | 34.529536 | 64.538473 | 144.347131 | 103.768075 | 189.097214 |
| 2029 | 2.343639 | 1.653252 | 3.080139 | 5.870214 | 3.629234 | 8.223797 | 49.068209 | 34.647746 | 64.781533 | 142.313664 | 102.295518 | 186.415258 |
| 2030 | 2.346818 | 1.649787 | 3.090047 | 5.81635 | 3.587798 | 8.155476 | 49.152465 | 34.706772 | 64.885737 | 141.345843 | 101.688327 | 184.998691 |
| 2031 | 2.349998 | 1.646322 | 3.099954 | 5.762539 | 3.546362 | 8.087156 | 49.236352 | 34.765798 | 64.98994 | 140.376299 | 101.081136 | 183.582124 |
| 2032 | 2.353163 | 1.642858 | 3.109861 | 5.708733 | 3.504926 | 8.018835 | 49.319419 | 34.824823 | 65.094144 | 139.403465 | 100.473945 | 182.165557 |
| 2033 | 2.356338 | 1.639393 | 3.119769 | 5.654976 | 3.463489 | 7.950514 | 49.402544 | 34.883849 | 65.198348 | 138.429833 | 99.866755 | 180.74899 |
| 2034 | 2.359554 | 1.635928 | 3.129676 | 5.601328 | 3.422053 | 7.882194 | 49.48647 | 34.942874 | 65.302551 | 137.457738 | 99.259564 | 179.332422 |
| 2035 | 2.360753 | 1.633903 | 3.133974 | 5.593521 | 3.415687 | 7.870179 | 49.471717 | 34.93757 | 65.270151 | 137.411614 | 99.391098 | 179.035487 |
| 2036 | 2.361965 | 1.631877 | 3.138272 | 5.58579 | 3.40932 | 7.858165 | 49.456986 | 34.932265 | 65.237751 | 137.365233 | 99.522633 | 178.738551 |
| 2037 | 2.363174 | 1.629852 | 3.14257 | 5.578095 | 3.402954 | 7.84615 | 49.442022 | 34.92696 | 65.20535 | 137.317996 | 99.654167 | 178.441615 |
| 2038 | 2.364404 | 1.627826 | 3.146868 | 5.570468 | 3.396587 | 7.834135 | 49.427374 | 34.921656 | 65.17295 | 137.27146 | 99.785702 | 178.144679 |
| 2039 | 2.365675 | 1.625801 | 3.151166 | 5.562954 | 3.390221 | 7.822121 | 49.413557 | 34.916351 | 65.140549 | 137.226924 | 99.917236 | 177.847743 |
| 2040 | 2.366977 | 1.623775 | 3.155465 | 5.555522 | 3.383854 | 7.810106 | 49.400334 | 34.911047 | 65.108149 | 137.183826 | 100.048771 | 177.550808 |
| ASMR | Myocardial Disease attributable to High temperature | | | Myocardial Disease attributable to  Low temperature | | | Ischemic Heart Disease attributable to  High temperature | | | Ischemic Heart Disease attributable to  Low temperature | | |
| Year | Both | Female | Male | Both | Female | Male | Both | Female | Male | Both | Female | Male |
| 1992 | 0.047928 | 0.037713 | 0.060676 | 0.545404 | 0.452268 | 0.643956 | 1.231799 | 0.97126 | 1.560566 | 14.621647 | 12.374499 | 17.244541 |
| 1993 | 0.053062 | 0.041287 | 0.0679 | 0.549836 | 0.454848 | 0.648069 | 1.329672 | 1.037094 | 1.701984 | 14.76419 | 12.51874 | 17.33633 |
| 1994 | 0.061675 | 0.048479 | 0.078048 | 0.51193 | 0.417819 | 0.609488 | 1.447002 | 1.140418 | 1.833386 | 13.857749 | 11.724019 | 16.279126 |
| 1995 | 0.065368 | 0.050548 | 0.083741 | 0.518954 | 0.420179 | 0.622178 | 1.578035 | 1.238131 | 2.000866 | 13.830839 | 11.660004 | 16.305711 |
| 1996 | 0.053028 | 0.040863 | 0.068108 | 0.528006 | 0.427245 | 0.633637 | 1.343371 | 1.046341 | 1.714081 | 14.237779 | 12.021691 | 16.751078 |
| 1997 | 0.042706 | 0.033189 | 0.054703 | 0.497003 | 0.399276 | 0.601088 | 1.274902 | 0.992346 | 1.631993 | 13.399738 | 11.300747 | 15.79045 |
| 1998 | 0.075587 | 0.058458 | 0.096728 | 0.464429 | 0.373911 | 0.560591 | 1.81915 | 1.414877 | 2.325761 | 12.421955 | 10.47125 | 14.641529 |
| 1999 | 0.059738 | 0.046204 | 0.07655 | 0.45637 | 0.365651 | 0.553013 | 1.486237 | 1.164858 | 1.883174 | 12.390657 | 10.447271 | 14.591095 |
| 2000 | 0.058078 | 0.045032 | 0.074346 | 0.449995 | 0.353925 | 0.553357 | 1.458015 | 1.129311 | 1.868571 | 12.402289 | 10.375076 | 14.717107 |
| 2001 | 0.053398 | 0.041304 | 0.068206 | 0.431947 | 0.33739 | 0.533824 | 1.460821 | 1.13586 | 1.864767 | 12.087733 | 10.11303 | 14.337889 |
| 2002 | 0.066511 | 0.050681 | 0.085924 | 0.424189 | 0.32707 | 0.528523 | 1.73094 | 1.326222 | 2.234079 | 11.833261 | 9.88916 | 14.052175 |
| 2003 | 0.066647 | 0.051927 | 0.084681 | 0.443309 | 0.339824 | 0.553831 | 1.654029 | 1.261199 | 2.146476 | 12.316057 | 10.243706 | 14.710096 |
| 2004 | 0.058303 | 0.04467 | 0.075021 | 0.433651 | 0.327641 | 0.546669 | 1.479225 | 1.107071 | 1.948829 | 11.630317 | 9.612296 | 13.983743 |
| 2005 | 0.070733 | 0.053936 | 0.090816 | 0.452436 | 0.335151 | 0.576875 | 1.715899 | 1.300671 | 2.233613 | 11.726326 | 9.646753 | 14.16475 |
| 2006 | 0.068664 | 0.052437 | 0.087937 | 0.421237 | 0.310087 | 0.538612 | 1.669367 | 1.265221 | 2.173703 | 10.827676 | 8.930907 | 13.043625 |
| 2007 | 0.072711 | 0.054603 | 0.094162 | 0.41721 | 0.305732 | 0.535708 | 1.74336 | 1.314523 | 2.275188 | 10.694121 | 8.736662 | 13.009033 |
| 2008 | 0.055135 | 0.041968 | 0.070797 | 0.420005 | 0.308741 | 0.538218 | 1.423652 | 1.051874 | 1.885436 | 10.766617 | 8.753961 | 13.148801 |
| 2009 | 0.072973 | 0.055262 | 0.094204 | 0.385062 | 0.28579 | 0.490474 | 1.836204 | 1.354816 | 2.431496 | 10.319422 | 8.405786 | 12.589899 |
| 2010 | 0.091483 | 0.067272 | 0.119857 | 0.382156 | 0.284976 | 0.487099 | 2.145903 | 1.588877 | 2.826846 | 10.591278 | 8.565032 | 13.00906 |
| 2011 | 0.062908 | 0.047383 | 0.081544 | 0.364118 | 0.273179 | 0.462991 | 1.634012 | 1.216684 | 2.147341 | 10.67303 | 8.600754 | 13.149024 |
| 2012 | 0.077279 | 0.058489 | 0.099809 | 0.342029 | 0.257412 | 0.434731 | 1.977388 | 1.483504 | 2.581758 | 10.448667 | 8.380752 | 12.93704 |
| 2013 | 0.065373 | 0.049704 | 0.084178 | 0.342215 | 0.256363 | 0.436055 | 1.825513 | 1.36989 | 2.395016 | 10.272038 | 8.217647 | 12.76523 |
| 2014 | 0.071262 | 0.054066 | 0.091842 | 0.327338 | 0.243218 | 0.419824 | 1.965018 | 1.454662 | 2.608918 | 9.943287 | 7.931152 | 12.396763 |
| 2015 | 0.07268 | 0.05482 | 0.093941 | 0.310595 | 0.2293 | 0.400073 | 1.965048 | 1.475863 | 2.570261 | 9.342941 | 7.45541 | 11.641121 |
| 2016 | 0.083071 | 0.062456 | 0.107652 | 0.296782 | 0.218192 | 0.383387 | 2.190914 | 1.659028 | 2.841474 | 9.053983 | 7.230674 | 11.264912 |
| 2017 | 0.078389 | 0.058613 | 0.101749 | 0.290895 | 0.212143 | 0.377662 | 2.09259 | 1.580635 | 2.711559 | 9.013853 | 7.172355 | 11.234965 |
| 2018 | 0.073978 | 0.054969 | 0.096168 | 0.282422 | 0.205007 | 0.368085 | 2.049631 | 1.546286 | 2.654621 | 8.939253 | 7.100884 | 11.150217 |
| 2019 | 0.085631 | 0.062737 | 0.112349 | 0.280599 | 0.201824 | 0.368016 | 2.230873 | 1.671708 | 2.900112 | 8.832465 | 6.961681 | 11.08686 |
| 2020 | 0.06894 | 0.049426 | 0.091873 | 0.276011 | 0.196013 | 0.365033 | 1.953837 | 1.462351 | 2.542909 | 8.646538 | 6.796684 | 10.885086 |
| 2021 | 0.063533 | 0.04545 | 0.084687 | 0.270766 | 0.19311 | 0.357042 | 1.874246 | 1.40752 | 2.435222 | 8.613737 | 6.800866 | 10.807656 |
| 2022 | 0.074765 | 0.053677 | 0.099201 | 0.260619 | 0.184511 | 0.345276 | 2.074571 | 1.55594 | 2.693757 | 8.340695 | 6.597318 | 10.434993 |
| 2023 | 0.075025 | 0.053531 | 0.099874 | 0.254176 | 0.178882 | 0.338056 | 2.08662 | 1.564158 | 2.710039 | 8.184676 | 6.476087 | 10.237844 |
| 2024 | 0.07529 | 0.053386 | 0.100547 | 0.247746 | 0.173254 | 0.330836 | 2.098877 | 1.572376 | 2.726321 | 8.029237 | 6.354857 | 10.040695 |
| 2025 | 0.075459 | 0.053219 | 0.101061 | 0.243705 | 0.169792 | 0.326177 | 2.105822 | 1.577093 | 2.734619 | 7.929046 | 6.281056 | 9.905446 |
| 2026 | 0.075626 | 0.053053 | 0.101575 | 0.23966 | 0.166329 | 0.321518 | 2.112661 | 1.581811 | 2.742917 | 7.82855 | 6.207256 | 9.770197 |
| 2027 | 0.075788 | 0.052887 | 0.102089 | 0.235608 | 0.162867 | 0.316859 | 2.119347 | 1.586529 | 2.751215 | 7.727569 | 6.133455 | 9.634948 |
| 2028 | 0.075951 | 0.052721 | 0.102603 | 0.231557 | 0.159404 | 0.3122 | 2.126045 | 1.591247 | 2.759513 | 7.626573 | 6.059655 | 9.499699 |
| 2029 | 0.076117 | 0.052554 | 0.103118 | 0.227513 | 0.155942 | 0.307542 | 2.132845 | 1.595965 | 2.767811 | 7.525851 | 5.985854 | 9.364451 |
| 2030 | 0.076234 | 0.052425 | 0.103496 | 0.225609 | 0.154341 | 0.305264 | 2.135614 | 1.598105 | 2.770506 | 7.478381 | 5.957858 | 9.290114 |
| 2031 | 0.076348 | 0.052296 | 0.103874 | 0.223703 | 0.15274 | 0.302987 | 2.138312 | 1.600245 | 2.7732 | 7.430669 | 5.929861 | 9.215777 |
| 2032 | 0.07646 | 0.052168 | 0.104252 | 0.22179 | 0.151138 | 0.300709 | 2.140896 | 1.602385 | 2.775895 | 7.382567 | 5.901865 | 9.14144 |
| 2033 | 0.076571 | 0.052039 | 0.10463 | 0.219877 | 0.149537 | 0.298432 | 2.143456 | 1.604525 | 2.77859 | 7.334312 | 5.873868 | 9.067103 |
| 2034 | 0.076685 | 0.05191 | 0.105008 | 0.217968 | 0.147936 | 0.296155 | 2.146063 | 1.606665 | 2.781285 | 7.286115 | 5.845872 | 8.992766 |
| 2035 | 0.076758 | 0.051837 | 0.105243 | 0.217796 | 0.147789 | 0.295877 | 2.145122 | 1.606585 | 2.778917 | 7.28492 | 5.857681 | 8.973495 |
| 2036 | 0.076831 | 0.051765 | 0.105477 | 0.217625 | 0.147641 | 0.2956 | 2.144148 | 1.606505 | 2.776549 | 7.283621 | 5.86949 | 8.954225 |
| 2037 | 0.076903 | 0.051692 | 0.105712 | 0.217452 | 0.147494 | 0.295322 | 2.143122 | 1.606424 | 2.774181 | 7.282176 | 5.881299 | 8.934954 |
| 2038 | 0.076976 | 0.05162 | 0.105946 | 0.217282 | 0.147346 | 0.295045 | 2.142103 | 1.606344 | 2.771812 | 7.280728 | 5.893108 | 8.915684 |
| 2039 | 0.077051 | 0.051548 | 0.106181 | 0.217116 | 0.147199 | 0.294767 | 2.141134 | 1.606264 | 2.769444 | 7.279381 | 5.904917 | 8.896414 |
| 2040 | 0.077127 | 0.051475 | 0.106416 | 0.216955 | 0.147051 | 0.29449 | 2.140191 | 1.606183 | 2.767076 | 7.278088 | 5.916726 | 8.877143 |
